# Triple the fun: tris(ferrocenyl)arene-based gold(i) complexes for redox-switchable catalysis†

**DOI:** 10.1039/d0sc03604h

**Published:** 2020-08-03

**Authors:** Axel Straube, Peter Coburger, Luis Dütsch, Evamarie Hey-Hawkins

**Affiliations:** Institute of Inorganic Chemistry, Universität Leipzig Johannisallee 29 D-04103 Leipzig Germany hey@uni-leipzig.de https://anorganik.chemie.unileipzig.de/de/anorganik/ak-hey-hawkins/; Institute of Inorganic Chemistry, Universität Regensburg Universitätsstr. 31 D-93053 Regensburg Germany

## Abstract

The modular syntheses of *C*_3_-symmetric tris(ferrocenyl)arene-based tris-phosphanes and their homotrinuclear gold(i) complexes are reported. Choosing the arene core allows fine-tuning of the exact oxidation potentials and thus tailoring of the electrochemical response. The tris[chloridogold(i)] complexes were investigated in the catalytic ring-closing isomerisation of *N*-(2-propyn-1-yl)benzamide, showing cooperative behaviour *vs.* a mononuclear chloridogold(i) complex. Adding one, two, or three equivalents of 1,1′-diacetylferrocenium[tetrakis(perfluoro-*tert*-butoxy)aluminate] as an oxidant during the catalytic reaction (*in situ*) resulted in a distinct, stepwise influence on the resulting catalytic rates. Isolation of the oxidised species is possible, and using them as (pre-)catalysts (*ex situ* oxidation) confirmed the activity trend. Proving the intactness of the P–Au–Cl motif during oxidation, the tri-oxidised benzene-based complex has been structurally characterised.

## Abstract 2

Trinuclear gold(i) complexes of *C*_3_-symmetric tris(ferrocenyl)arene-based tris-phosphanes with four accessible oxidation states catalyse the ring-closing isomerisation of *N*-(2-propyn-1-yl)benzamide with different rates depending on their redox state.

## Graphical abstract

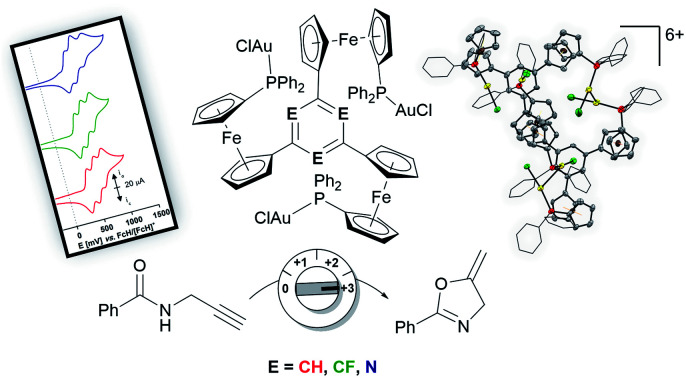

## Introduction

**Chart 1:**
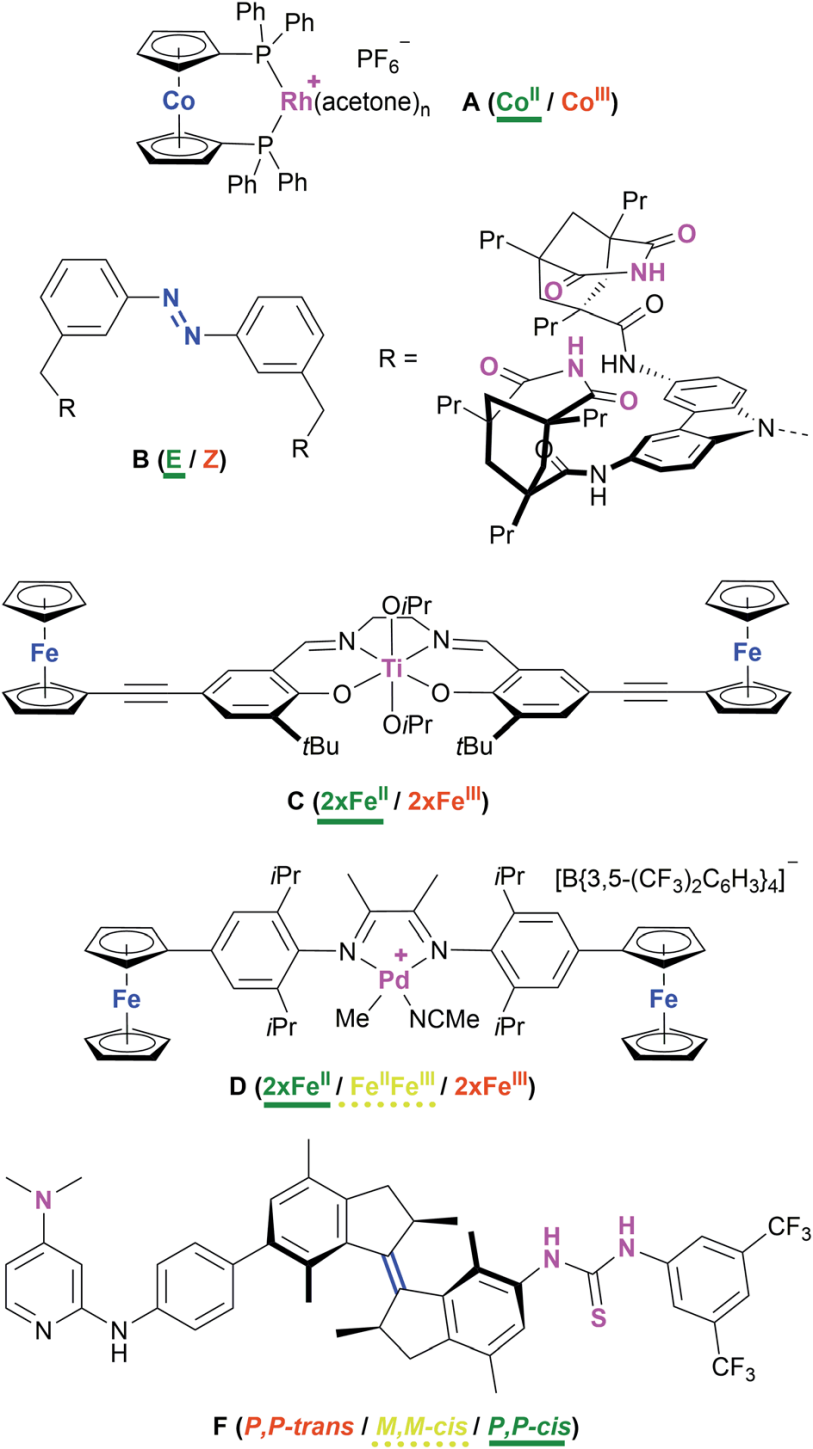
Examples of redox- (**A** [activity indicated for hydrogenation of alkenes],^4^**C**,^21^**D**^29^) and light-/thermo-switchable (**B**,^5^**F**^42^) catalyst systems. The switching moiety is highlighted in blue and the catalytic sites are highlighted in purple. The most active (ON) state is labelled in green and underlined (bold), the intermediate state in yellow and underlined (dashed), and the least active state (OFF) is labelled in red.

Attaining control over catalytic reactions by external stimuli, preferably with as tight a grip as demonstrated in Nature's complex regulation pathways, has long been at the centre of interest for the chemical community.^1–3^ It has been 25 years since the seminal work by the groups of Wrighton and Rebek, the former demonstrating a cobaltocene-based rhodium complex **A** (Chart 1) to function as either a good hydrogenation (Co^II^) or hydrosilylation (Co^III^) catalyst, the activity for the corresponding reaction being significantly lower in the respective other oxidation state.^4^ Rebek and co-workers used light to reversibly isomerise an azobenzene-derived organo-catalyst **B** and thus tuning it towards an amide-forming reaction.^5^ A vast body of work concerning artificially switchable catalysis has since been assembled,^6–9^ with a major focus on redox-switchable catalysis (RSC).^10–13^ Even though switching between oxidation states is usually achieved through the addition of chemical redox agents, RSC holds great potential for applications using electrodes and thus greatly reducing chemical waste.^14–18^ Conceptually, both ligand and metal can form the centres of (reversible) electron transfer for changing the activity state of the catalyst.^11^ For ligand-based switching, ferrocene has proven and remains a cornerstone for ligand design owing to its amenability to synthetic modification and favourable, while modifiable, redox properties.^19,20^

Even though one of the first redox-switchable catalysts by Long and co-workers used for the ring-opening polymerisation of *rac*-lactide, **C** (Chart 1),^21^ already contained two pendant ferrocenyl groups, and a plethora of compounds featuring multiple ferrocenyl groups is available,^22–28^ only one report by Zhao and Chen deals with exploiting the possibility of addressing more than just two catalytic states. Employing an α-diimine palladium catalyst **D** with two pendant ferrocenyl groups, the three activity states resulting from sequential two-step oxidation were found to differ with respect to polymerisation activity, resulting in tuneable polymer molecular weight, topology, and polydispersity.^29^ The scarcity of this concept in RSC is surprising given how multi-state switchable molecules feature prominently in molecular machines^30–33^ and molecular electronics and logic.^34–41^ Combining molecular machines and catalysis, Wang and Feringa have impressively demonstrated a unidirectionally light- and thermo-switchable rotor **F** to display three different activity states in an organo-catalysed asymmetric Michael addition, in turn also leading to different enantioselectivities (*P*,*P-trans*: racemic; *M*,*M-cis*: *S* enantiomer; *M*,*M-trans*: *R* enantiomer).^42^

**Scheme 1:**
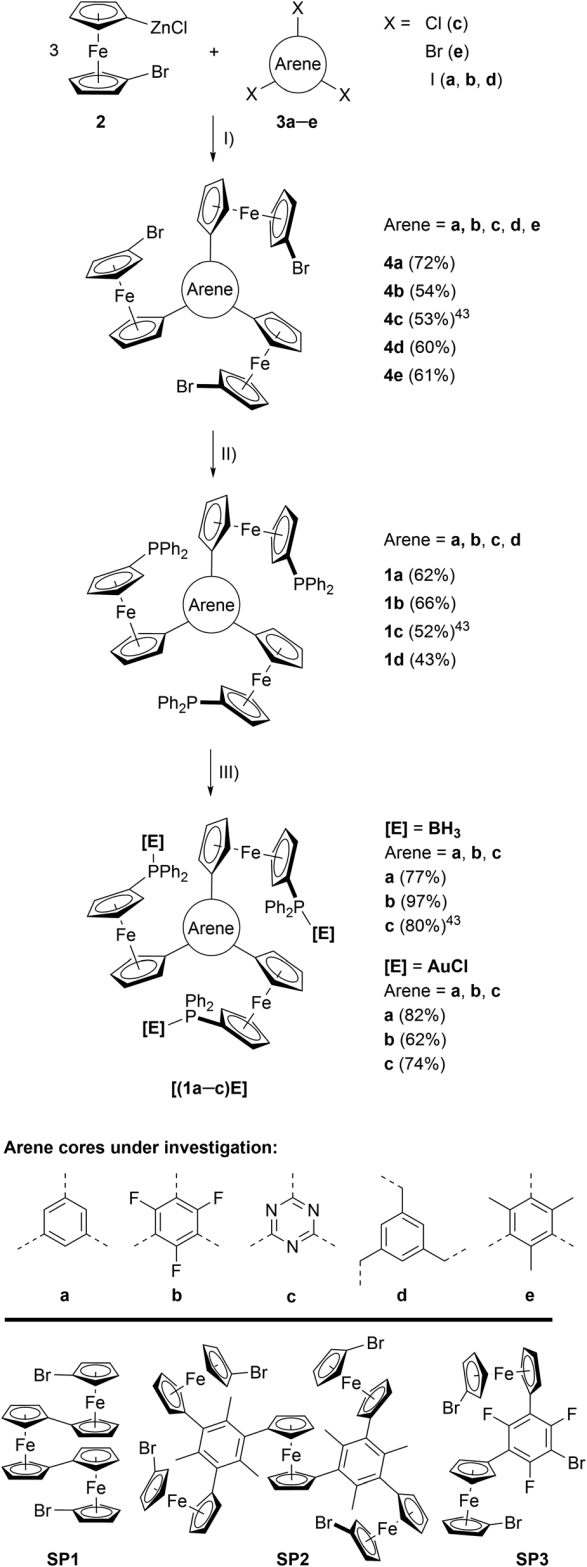
(Top) Preparation of tris-phosphanes **1a–d** and complexes **[(1a–c)E]**. (I) Pd precatalyst, THF, r. t.; (II) *n*BuLi, Ph_2_PCl, THF, −80 °C to r. t.; (III) **[E]**·X (**[E]**·X = BH_3_·SMe_2_ or [AuCl(tht)]), CH_2_Cl_2_, r. t.; (Bottom) crystallographically characterised side products obtained during the syntheses of **4d** (**SP1**), **4e** (**SP2**), and **4b** (**SP3**). For their solid-state structures, see Fig. S3.†

Expanding on this idea, we sought to prepare a system with four accessible oxidation states by making use of the *C*_3_-symmetric *s*-tris(ferrocenyl)arene motif recently first exploited for ligand design.^43^ So far, we have focused on the monotopic use of these tris-phosphanes **1** (Scheme 1); however, put to use as tritopic ligands renders them miniaturised and thus easier-to-study analogues of the ferrocenyl-based dendrimers we could recently show to function as redox-switchable ruthenium(ii) catalysts for the isomerisation of an allylic alcohol^44,45^ and for transfer hydrogenation of a prochiral ketone with two distinct catalytic activity states (neutral and fully oxidised).^46^

*C*_3_ symmetry in general has been recognised as a promising ligand design principle,^47,48^ and adorning one ligand fragment with multiple metal centres can furthermore allow for cooperative effects to occur.^49–54^ The underlying key feature of the catalytically active sites in close proximity has been also found crucial in the design of single-molecule magnets^55,56^ and for molecular recognition and supramolecular assemblies.^57–60^

## Results and discussion

### Preparation and electrochemical characterisation of tris-phosphanes and trinuclear gold(i) complexes

The preparation of tris-phosphanes **1** (Scheme 1, top), as described for **1c**,^43^ starts with a triple Negishi coupling of the *in situ*-generated 1-bromo-1′-ferrocenylene zinc halide **2**, prepared from 1,1′-dibromoferrocene,^61^*n*-butyllithium and anhydrous zinc chloride in THF,^25,62^ with *C*_3_-symmetric arenes **3a–e**. Introducing electron-withdrawing (**b**, **c**) and electron-donating (**e**) arenes as well as a tris-benzylic arene core (**d**), preventing conjugation between the three ferrocenylene groups, allows for modularly fine-tuning the system's electrochemical response (*vide infra*). Tris(1-bromo-1′-ferrocenylene)arenes **4a–e** are obtained in moderate to good yields as crystalline solids and the potential to incorporate more and highly functionalised cores is only limited by the functional group tolerance of the Negishi protocol.^63^**4a**, **b**, **d**, **e** were analysed by single crystal X-ray diffraction (XRD) analysis (Fig. S1 and Table S3†), their structural parameters falling within the expected standard ranges for *C*_3_-symmetric tris(ferrocenyl)arenes.^25,64–68^ In solution, **4a–e** are characterised by unhindered rotation about the C_arene_–C_Cp_ bonds on the NMR timescale, thus displaying *C*_3v_ symmetry in their ^1^H and ^13^C{^1^H} NMR spectra.

Depending on the choice of Pd^II^ precatalyst, formation of bi- and triferrocenes has also been observed. Among them, triferrocene **SP1** (Scheme 1, bottom) had not been crystallographically characterised until now^69^ and is most likely formed from hindered reductive elimination followed by a second transmetallation step of **2** onto the Pd catalyst.^70^ The formation of pentanuclear **SP2** during the preparation of **4e** can be similarly rationalised. Together with the isolation of only di-ferrocenylated bromotrifluorobenzene **SP3**, these findings point towards the use of our synthetic protocol to access ever more complex and functionalisable redox-active structures.

**Fig. 1:**
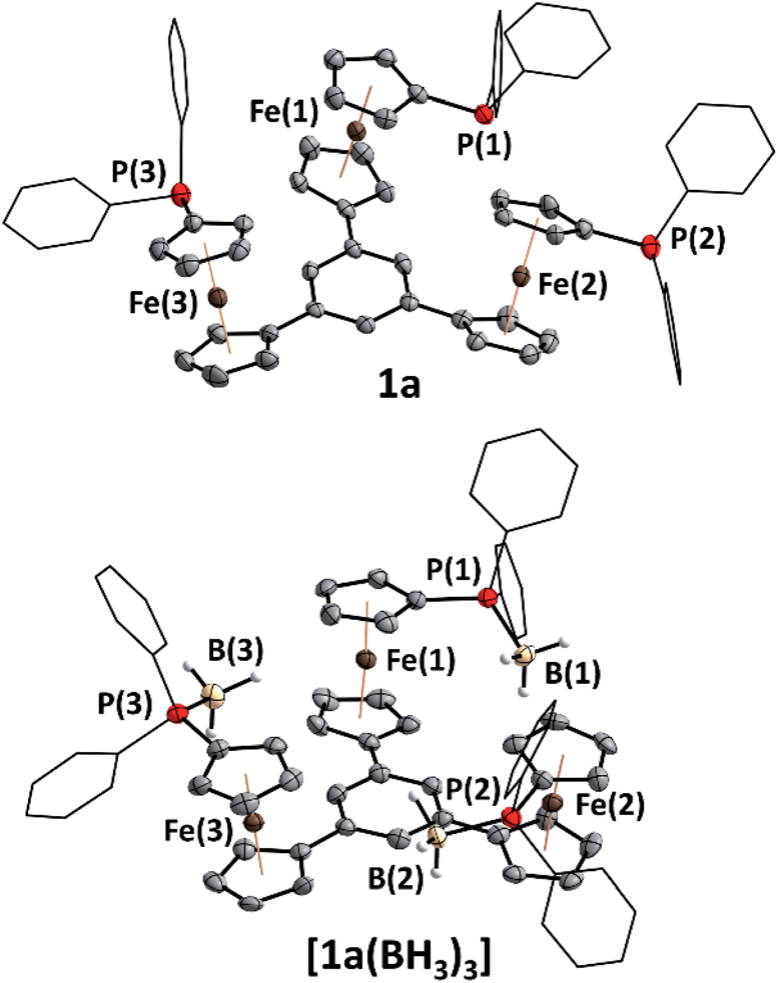
Molecular structures of tris-phosphane **1a** and borane adduct **[1a(BH3)3]**. Thermal ellipsoids are set at the 50% probability level. For clarity, the phenyl rings are depicted in wireframe style, disorder in the phenyl rings of **1a** has been omitted, and hydrogen atoms except for those of the BH_3_ groups in **[1a(BH3)3]** are not depicted.

Tris-phosphanes **1a–d** are obtained following the established protocol and purified by column chromatography.^43^ Attempting the synthesis of a mesitylene-based tris-phosphane from **4e** has only resulted in the isolation of impure trace amounts, potentially due to side reactions involving the methyl protons of **4e**. Crystals suitable for XRD analysis have been obtained for **1a** and **1d** (Fig. 1 and S4†). Their structural parameters are in agreement with those previously reported for **1c**^43^ and other diphenylphosphanyl ferrocenes.^71–74^ Similar to their precursors **4**, no rotamers are observed in solution, while small changes in the ^31^P{^1^H} NMR chemical shifts of **1a–d** reflect the electronic nature of the arene core (Table 1).

**Table 1:** ^31^P{^1^H} NMR chemical shifts (ppm) of tris-phosphanes **1a–d**, their corresponding adducts with BH_3_, and gold(i) chloride complexes, determined in CD_2_Cl_2_

	**a**	**b**	**c**	**d**
**1**	−17.5	−17.6	−18.4 (ref. 43)	−17.1
**[1(BH3)3]**	15.7	15.5	15.6 (ref. 43)	—
**[1(Au)3]**	28.3	28.1	27.9^a^	—

a In CDCl_3_.

Borane adducts **[1a–c(BH3)3]** have been prepared (**[1c(BH3)3]** has been reported before)^43^ to study the electrochemistry of the phosphanes (*vide infra*), since direct cyclopentadienyl–phosphorus bonds usually render the oxidations irreversible due to the lone pair of electrons on phosphorus.^75–77^ Adduct **[1a(BH3)3]** was found to crystallise with crystallographic *C*_3_ symmetry (space group *R*3̄, Fig. 1) and compares well (Table S5†) to structural parameters of similar ferrocenyl-phosphane boranes described by us^78^ as well as by Štěpnička and co-workers.^79^

Recently we reported the capability of **1c** to bind coinage metal ions in a tridentate trigonal planar coordination mode;^43^ now we have turned our focus to potential trinuclear gold(i) complexes of **1a–c**. Indeed, reacting the tris-phosphanes with the common gold(i) precursor [AuCl(tht)] (tht = tetrahydrothiophene) in slight stoichiometric excess afforded, after simple precipitation, homotrinuclear metal complexes **[1a–c(Au)3]** in good yields. Their trinuclear composition is confirmed by CHN analyses, multinuclear NMR spectroscopy, and they remain homotrinuclear in the gas phase as assessed by high-resolution electrospray-ionisation mass spectrometry (HR-ESI MS).

**Fig. 2:**
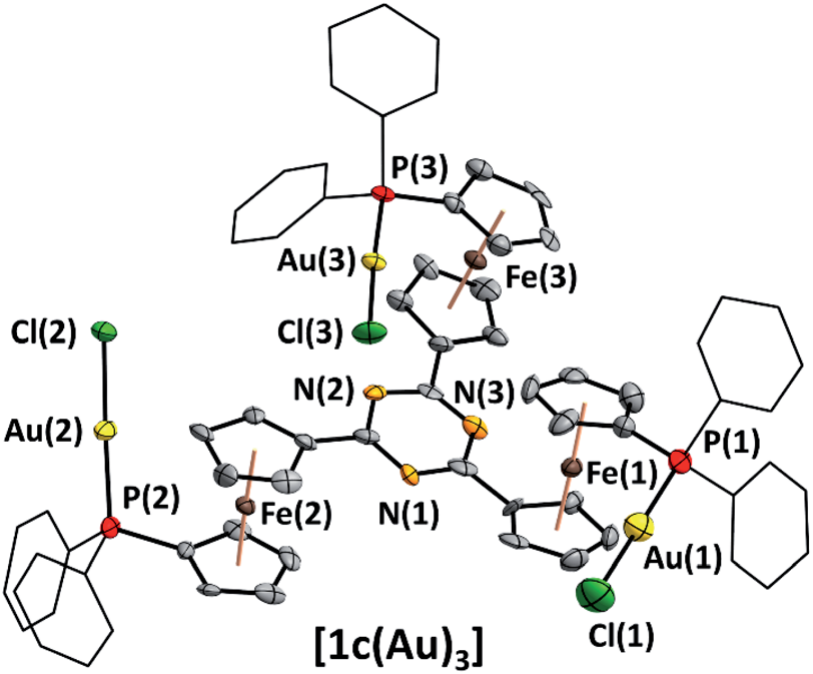
Molecular structure of homotrinuclear **[1c(Au)3]** with partial atom numbering scheme. Thermal ellipsoids are set at the 50% probability level. For clarity, the phenyl rings are depicted in wireframe style, and co-crystallised solvent, disorder in the P(2)–Au(2)–Cl(2) fragment, and hydrogen atoms have been omitted.

Representative for all three gold(i) chloride complexes, the molecular structure of **[1c(Au)3]** was determined from suitable crystals (Fig. 2) and confirms the trinuclearity in the solid state as well. In line with the experimentally determined non-centrosymmetric space group *P*1, **[1c(Au)3]** crystallises as an enantiopure compound (*x*_Flack_ = −0.008(7)); however, in solution, there is no indication of a corresponding hindered rotation as assessed from NMR spectroscopy.

The P–Au bond lengths (2.225(5)–2.231(6) Å, Tables 2 and S6†) are similar to mononuclear analogues featuring an N-heterocyclic substituent at the 1′-position of the 1-diphenylphosphanyl-ferrocenylene moiety described by the groups of Siemeling (*d*(P–Au) = 2.242 Å [2-pyridyl], 2.234 Å [3-pyridyl])^80^ and Lang (*d*(P–Au) = 2.215 Å [2,2′:6′,2′′-terpyridin-4′-yl]),^81^ yet **[1c(Au)3]** is the first such complex containing ferrocene in the tris-phosphane backbone and the second to incorporate three ferrocene moieties into a trinuclear gold complex.^82^

The spectral data of both **[1a(Au)3]** and **[1b(Au)3]** are in line with that of **[1c(Au)3]** and all complexes are thus presumed to have similar structures. Notably, **[1c(Au)3]** does not show aurophilic interactions^83–85^ or close ferrocene⋯metal contacts^86^ in the solid state (for M⋯M distances see Table 2), setting it apart from related structures regularly displaying aurophilic interactions (Table S7†). In the context of cooperative effects operating in multimetallic catalysis,^49,52,87–90^**[1c(Au)3]** thus falls short of the proximity criterion formulated by Feringa as the gold⋯gold separation exceeds 6 Å.^49^ Yet, owing to the flexibility of the ligand backbone, gold⋯gold distances in solution might well become much closer.

**Table 2:** Selected bond lengths, metal⋯metal distances [Å], and angles [°] of **[1c(Au)3]**, numbered according to Fig. 2 (*m* = 1–3). Full parameters in Table S6

Au(*m*)–P(*m*)	2.231(6)/2.286(7)/2.225(5)
Au(*m*)–Cl(*m*)	2.288(6)/2.229(4)/2.281(5)
P(*m*)–M(*m*)–Cl(*m*)	179.0(2)/177.0(2)/176.9(2)
Au(1,2,3)⋯Au(2,3,1)^a^	14.187(1)/8.012(1)/9.100(1)/*6.4204(9)*
Au(*m*)⋯Fe(*m*)	4.302(3)/4.080(3)/4.544(2)

a Intramolecular distances; the shortest intermolecular distance is shown italicised.

Regarding our vision of using **1**-derived complexes as “rotary” or “dimmable” switches for multi-redox-state applications, electrochemical characterisation by cyclic voltammetry (CV) was of utmost relevance. 1,3,5-Tris(ferrocenyl)arenes have previously been shown^91–93^ to display reversible redox activity and, in supporting electrolytes (SE) containing weakly coordinating anions such as [B(C_6_F_5_)_4_]^−^ or [B{3,5-(CF_3_)_2_C_6_H_3_}_4_]^−^ (BAr^F^_4_^−^),^94,95^ to furthermore be oxidisable in three separate, resolved steps.^25^

**Fig. 3:**
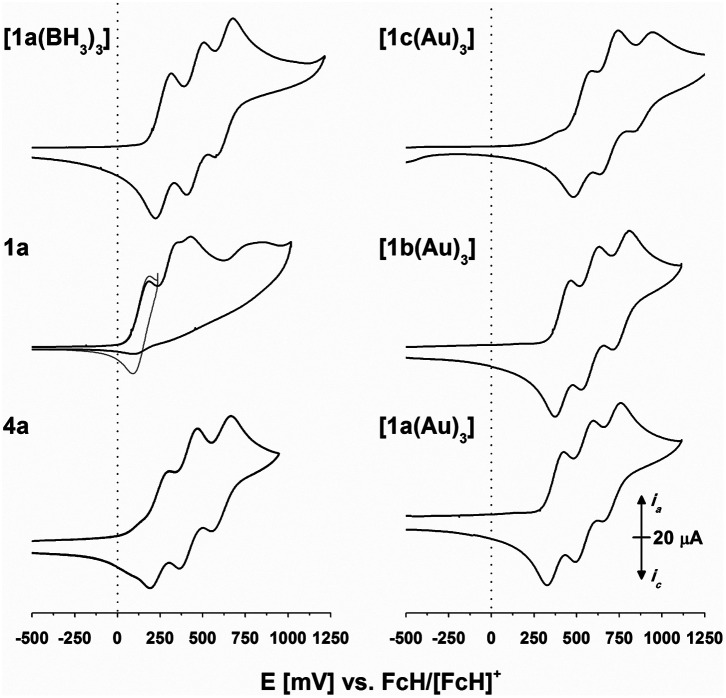
Cyclic voltammograms of **4a**, **1a** (the grey trace was recorded with a vertex potential of 245 mV *vs.* FcH/[FcH]^+^), **[1a(BH3)3]**, and **[1a–c(Au)3]** at 1 mmol L^−1^ in 0.1 mol L^−1^ (*n*Bu_4_N)BAr^F^_4_/CH_2_Cl_2_ (scan rate: 100 mV s^−1^, working electrode: glassy carbon, counter electrode: platinum wire). The 2^nd^ of 3 cycles is shown for all compounds (for full voltammograms, s. Fig. S8–S12†).

Triazine-based **4c** and **[1c(BH3)3]** displayed a similar behaviour,^43^ while **1c**, as expected, showed an irreversible electrochemical oxidation due to the direct cyclopentadienyl–phosphorus linkage.^75–77,96^ Their analogues reported herein share these redox features (s. Fig. 3, left, for **4a**, **1a**, and **[1a(BH3)]** and Fig. S8–S11†). Gratifyingly, the arene core determines the exact oxidation potentials (Tables 3 and S9–S12†), in line with substitution by electron-donating or -withdrawing groups. The choice of arene thus allows for electrochemically fine-tuning the whole system. The arene trend also holds true for **[1a–c(Au)3]** (Fig. 3, right). In a BF_4_^−^-based SE, the three gold(i) complexes show partly irreversible behaviour (Fig. S12†), but in all cases, their tris(ferrocenyl)arene cores are oxidised in one (quasi)reversible event. In a BAr^F^_4_^−^-based SE, the tris(ferrocenyl)arene core is again oxidised in three resolved steps. In our setup (0.1 mol L^−1^ (*n*Bu_4_N)BF_4_ or (*n*Bu_4_N)BAr^F^_4_ in CH_2_Cl_2_, −1.75 to 1.2 V *vs.* FcH/[FcH]^+^),^97^ no oxidation of gold(i) in **[1a–c(Au)3]** was observed.

**Table 3:** Redox potentials for the first iron-centred oxidation *E*^0^_1_ (*vs.* FcH/[FcH]^+^) of **4a–e**, **1a–d**, **[1a–c(BH3)3]**, and **[1a–c(Au)3]**, arranged according to ascending *E*^0^_1_

*E* ^0^_1_ (Δ*E*_p_)^a^ [mV]					
**4d**	195 (85)	**1d**	113^b^	**[1a(BH3)3]**	270 (91)
**4e**	212 (87)	**1a**	138 (98)	**[1b(BH3)3]**	353 (87)
**4a**	247 (100)	**1b**	206 (116)	**[1c(BH3)3]** ^43^	421 (100)
**4b**	293 (96)	**1c** ^43^	275 (160)	**[1a(Au)3]**	376 (101)
**4c** ^43^	386 (127)			**[1b(Au)3]**	419 (93)
				**[1c(Au)3]**	535 (108)

a Determined on 1 mmol L^−1^ samples in anhydrous 0.1 mol L^−1^ (*n*Bu_4_N)BAr^F^_4_/CH_2_Cl_2_ (working electrode: glassy carbon) at 100 mV s^−1^. The difference between oxidation and reduction potential, Δ*E*_p_, is given in brackets.

b Determined from square-wave voltammetry due to close peak-to-peak separation, leaving Δ*E*_p_ inaccessible.

This is in line with results from DFT calculations locating the HOMO at the iron centres; in the mono-oxidised model complex **[1a(Au)3]+**, the spin density solely resides at the three iron centres, too (Fig. S14–S19†). Among the three tris-phosphanes, **1c** yields the most anodically shifted redox potentials in its complexes (Fig. 3, right, and Table 3). In line with our previous findings, complexes of **1c** were also found to display the least straightforward electrochemistry such as cathodically shifted reductions connected to electron transfer-induced chemical transformations (EC mechanism; *cf.* Fig. S13†).^43^

### Redox-switchable gold(i) catalysis

**Scheme 2:**
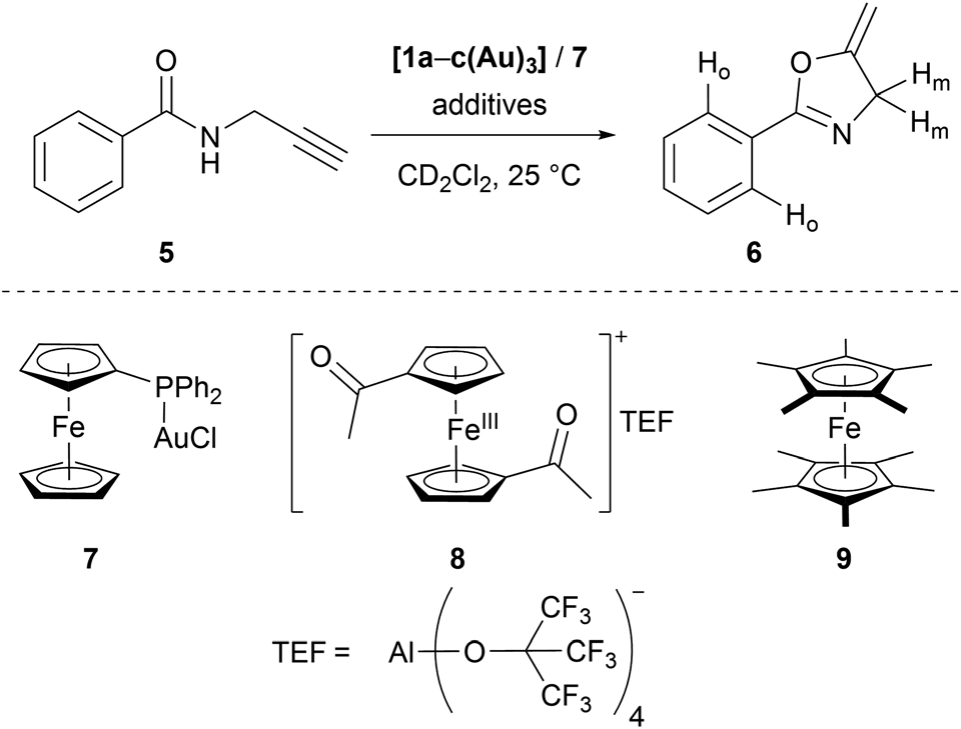
Gold(i)-catalysed ring-closing isomerisation of *N*-(2-propyn-1-yl)benzamide **5** to oxazoline **6** (top, labelled protons used for following the reaction), including alternative mononuclear (pre-)catalyst **7**, oxidant **8**, and reductant **9**.

Seeking to demonstrate the applicability of the stepwise oxidation of the *s*-tris(ferrocenyl)arene core, our choice fell on the gold-catalysed 5-*exo-dig* ring-closing isomerisation of *N*-(2-propyn-1-yl)benzamide (**5**) to 5-methylene-2-phenyl-4,5-dihydrooxazole (**6**) (Scheme 2) as a read-out. Uncovered by Hashmi and co-workers in 2004,^98^ the catalytic synthesis of oxazolines has quickly developed into a standard reaction for gold(i) complexes.^99–104^ The groups of Sarkar and Heinze, among others, have established the transformation of **5** to **6** as a platform to perform and study RSC using gold(i) catalysts.^105–109^ Rendering the gold(i) centres reversibly more Lewis-acidic and hence more catalytically active^110^ through oxidation of a connected ferrocenyl moiety is one way of obviating the sometimes problematic use of silver salts for activation of gold(i) pre-catalysts by halide abstraction and enables temporal control over the activity of the catalyst.^111–115^ Since we aimed for a detailed understanding of the switching process, the reaction was performed on the NMR scale in CD_2_Cl_2_, allowing for a time-resolved study of the reaction through protons H_o_ (reaction involving oxidised species) and H_m_ (reactions not involving oxidised species) of oxazoline **6** (Fig. S21 and S22†) *vs.* 1,3,5-trimethoxybenzene as an internal standard.^116–118^

**Fig. 4:**
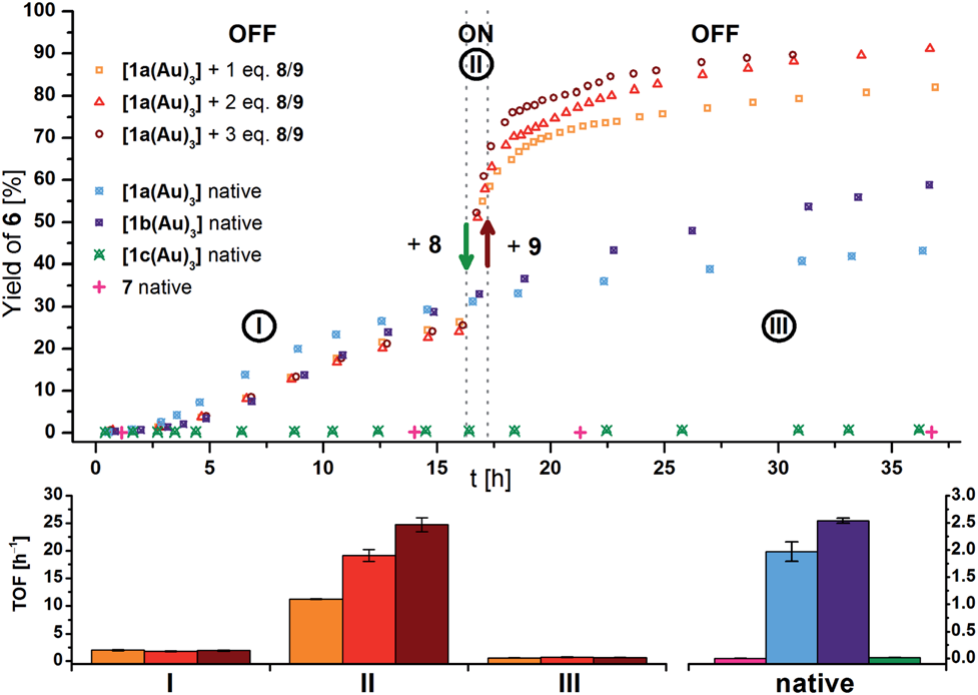
(Top) Yield-over-time graphs for native (crossed symbols) and redox-switchable (hollow symbols) gold(i)-catalysed cyclisation of **5** to **6** (3 mol% Au as **[1(Au)3]** and **7**, [**5**]_0_ = 60 mmol L^−1^, CD_2_Cl_2_, 25 °C). Arrows indicate the addition of additives. **I**: initial OFF phase with little catalytic activity; **II**: ON phase after addition of 1.0–3.0 eq. oxidant **8**; **III**: 2^nd^ OFF phase of little catalytic activity after addition of 1.1–3.3 eq. reductant **9**. (Bottom) Comparison of turnover frequencies (TOF), determined from linear fits of fixed time frames (s. Fig. S39–S45†) for the switched reaction phases shown above (left) and for the native complexes (right).

Native **[1a(Au)3]** (crossed light blue circles; Fig. 4), employed in a 3 mol% Au loading (referring to substrate **5**), performed with low activity (TOF = 2.0 ± 0.2 h^−1^; TOF fits, s. Fig. S39–S53†). A control experiment without any added gold(i) complex yielded no product. While **[1b(Au)3]** (crossed navy squares, TOF = 2.5 ± 0.1 h^−1^) performed slightly better than **[1a(Au)3]**, potentially due to the slightly more electron-withdrawing 1,3,5-trifluorobenzene core, **[1c(Au)3]** (crossed turquoise triangles) did, reproducibly, not show any catalytic activity under the same conditions. Given that the only difference is in the *s*-triazine core, these nitrogen atoms might interact with the amide protons of substrate **5**, in turn preventing the completion of the catalytic cycle by protodeauration.^98^ This hypothesis is supported comparing the ^1^H NMR spectra of **[1a(Au)3]** and **[1c(Au)3]** with 3 equivalents of **5**, respectively, at −60 °C in CD_2_Cl_2_, resulting in a signal splitting for the amide proton signal of **5** in presence of **[1c(Au)3]** (Fig. S23†).

In order to test for potential cooperative effects due to its three gold centres, [AuCl(FcPPh_2_)] (**7**) was prepared as a mononuclear gold complex analogue of **[1a(Au)3]**.^119^ Quite surprisingly, a catalytic evaluation of **7** has not yet been reported. As judged from ^31^P{^1^H} NMR spectroscopy (**[1a(Au)3]**: *δ*(CD_2_Cl_2_) = 28.3 ppm; **7**: *δ*(CDCl_3_) = 28.4 ppm) and CV (**[1a(Au)3]**: *E*^0^((*n*Bu_4_N)BF_4_) = 340 mV; **7**: *E*^0^((*n*Bu_4_N)PF_6_) = 320 mV),^119^**7** is a well-suited model compound with respect to the electronic properties of both gold(i) and the ferrocene unit.

At the same concentration of gold(i), **[1a(Au)3]** clearly outperformed the catalytically silent **7** (pink pluses, Fig. 4). This mirrors results from Mendoza-Espinosa and co-workers who have observed a similar effect in comparing tetranuclear mesoionic carbene gold(i) halide complexes and their mononuclear analogues in the hydroamination and hydrohydrazination of terminal alkynes.^90^ Peris and co-workers found a less prominent cooperative effect for the gold(i)-catalysed hydroamination of phenylacetylene using a trinuclear gold(i) chloride complex with a triphenylene-based tris(N-heterocyclic carbene) ligand.^53^

Contrastingly, an anti-cooperative effect was found when **[1a(Au)3]** and **7** were activated by halide abstraction (Fig. S24†) using NaBAr^F^_4_.^117,120^ At a 1 mol% gold(i) loading, the catalytic activity of **7/NaBArF4** (TOF = 20.1 ± 0.6 h^−1^) surpassed that of **[1a(Au)3]/NaBArF4** (TOF = 3.2 ± 0.1 h^−1^) greatly. Following the chloride abstraction of **[1a(Au)3]** by ^1^H and ^31^P{^1^H} NMR spectroscopy proved inconclusive but hinted at the slow formation of a *P*,*P′*-dicoordinated gold(i) complex (*δ*_P_ = 42.9 ppm, Fig. S25†).^121–123^ An HR-ESI mass spectrum showed signals corresponding to [M–Au–2Cl]^+^, [M–Au–3Cl]^2+^, and [M–2Cl]^2+^ species. The inferior performance of **[1a(Au)3]/NaBArF4***vs.***7/NaBArF4** might thus relate to the formation of chelated and therefore less substrate-accessible gold(i) species.

Given its favourable redox properties – the lowest oxidation potentials and three fully reversible and well-separated oxidation events in the BAr^F^_4_^−^-based SE (*cf.*Fig. 3) – **[1a(Au)3]** was chosen as a model (pre-)catalyst for initial tests concerning RSC. For the oxidation, 1,1′-diacetylferrocenium tetrakis(perfluoro-*tert*-butoxy)aluminate (= teflonate) (**8**) was chosen. Next to the ready synthetic availability from silver(i) teflonate^124^ and 1,1′-diacetylferrocene and the sufficiently high oxidation potential (*E*^0^ = 490 mV *vs.* FcH/[FcH]^+^ in CH_2_Cl_2_),^125^ the highly inert non-coordinating anion informed this choice. Smaller anions such as BF_4_^−^ and SbCl_6_^−^ have been shown to form tight ion pairs with ferrocenium,^105,126^ and anion effects in general have been found crucial for the understanding and tailoring of gold catalysts.^127^

After the reaction shown in Fig. 4 had reached a 25% yield (phase **I**), 1.0 (orange squares), 2.0 (red triangles), or 3.0 (maroon circles) equivalents of **8** were added. The catalytic activity increased considerably (phase **II**; *cf.*Fig. 4, bottom left), in line with findings for similar systems.^105–109^ Gratifyingly, the activity differed according to the amount of oxidant added: one equivalent of oxidant resulted in a 5.5-fold, two in a 10-fold, and three in a 13-fold increase of the experimentally determined TOF (Fig. 4, bottom). Upon addition of decamethylferrocene (**9**) as reductant (phase **III**), less-than-initial activity (TOF_Ø_ = 0.67 ± 0.06 h^−1^) was restored after some delay. In control experiments, employing only **8** or **8** + **9** did not trigger product formation (Fig. S20 and S21†).

The amplified activity is usually explained by an increase in Lewis acidity at the gold(i) site,^105–109^ and DFT calculations for mono-oxidised **[1a(Au)3]+** showed a decrease of energy of unoccupied gold-centred orbitals by over 1.9 eV (Fig. S14–S19†). Such a decrease in LUMO energy can cautiously be understood in terms of increased electrophilicity.^128^

**Fig. 5:**
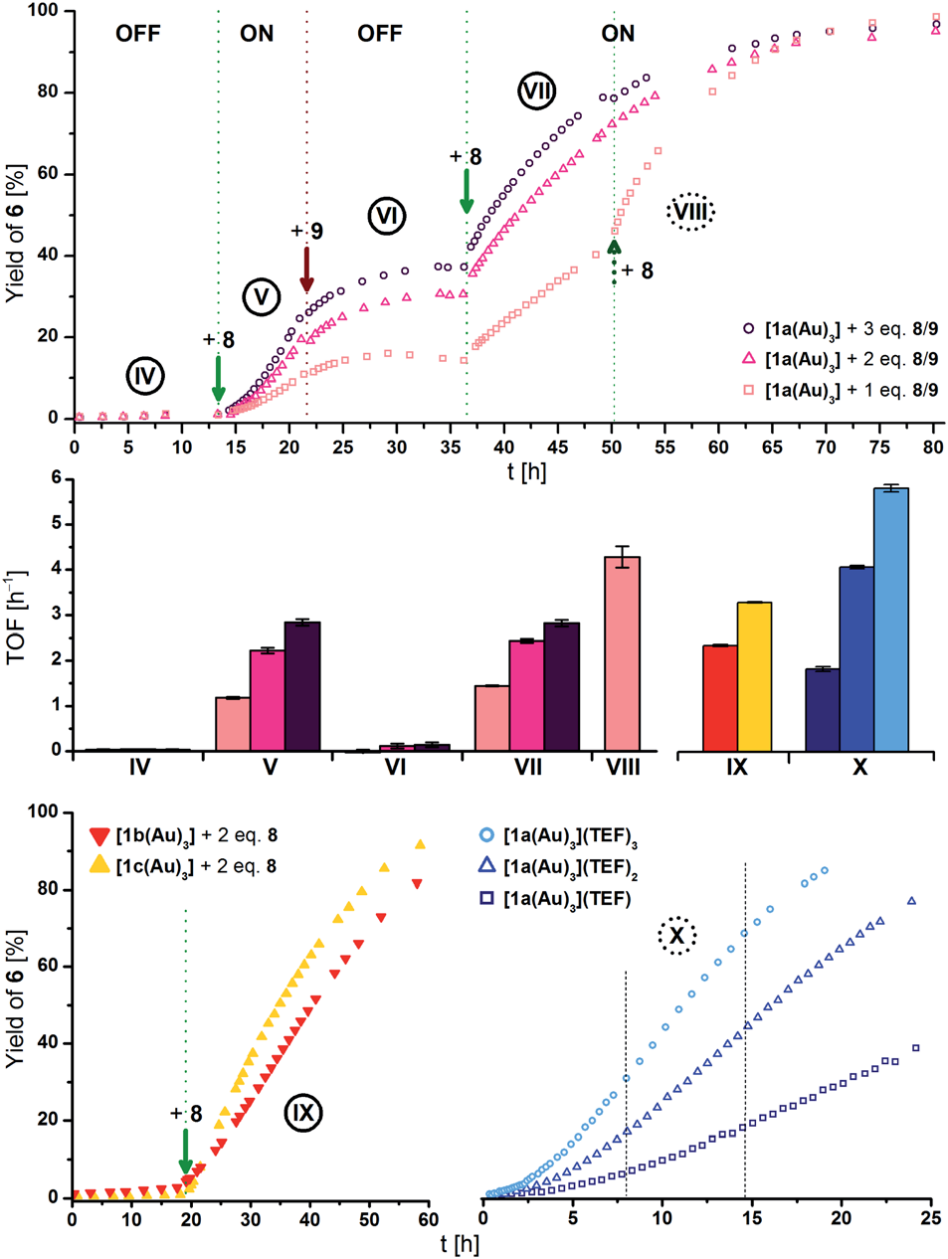
(Top) Yield-over-time graphs for redox-switchable gold(i)-catalysed cyclisation of **5** to **6** using **[1a(Au)3]** (1 mol% Au, [**5**]_0_ = 60 mmol L^−1^, CD_2_Cl_2_, 25 °C). Arrows indicate the addition of additives. **IV**: initial OFF phase; **V**: 1^st^ ON phase after addition of 1.0–3.0 eq. oxidant **8**; **VI**: 2^nd^ OFF phase after addition of 1.1–3.3 eq. reductant **9**; **VII**: 2^nd^ ON phase after addition of 1.1–3.3 eq. oxidant **8**; **VIII**: addition of 2.2 eq. **8** to 1 eq.-switched reaction (rose squares). (Centre) Comparison of turn-over frequencies (TOF) determined from linear fits (*cf.* Fig. S40–S48† for regression plots) of reaction phases **IV** to **VIII** (top), **IX** (bottom left), and **X** (bottom right). (Bottom) Left: yield-over-time graphs for gold(i)-catalysed cyclisation of **5** to **6** using **[1b(Au)3]** (red) and **[1c(Au)3]** (yellow; conditions as above), oxidised *in situ* with 2 eq. of **8**. Right: yield-over-time graphs for gold(i)-catalysed cyclisation of **5** to **6** using isolated oxidised complexes **[1a(Au)3](TEF)n** (*n* = 1–3, conditions as above). Dashed lines represent the chosen timeframe for linear fitting.

The different activities, which might correspond to distinct activity states in line with findings of Zhao and Chen,^29^ were found to be easier to distinguish at 1 mol% Au catalyst loading (Fig. 5, top), decelerating the reaction in so much as to allow for an **OFF**–**ON**–**OFF**–**ON** switching sequence. Moreover, adding two equivalents of **8** to a catalytic run previously oxidised by one equivalent of **8** (**VII** to **VIII**, rose squares), a distinct increase of activity was found. Due to the negligible initial activity (TOF < 0.05 h^−1^), the great increase in activity is difficult to quantify by numbers (Fig. 5, centre left). It is, however, evident that the TOF effected by the re-oxidised species (**VI** to **VII**) are slightly larger than those originating from the first oxidation for one (rose squares) and two (pink triangles), yet not for the second oxidation with three equivalents of **8** (purple circles). Oxidising what we assume to be the mono-oxidised species **[1a(Au)3]+** to presumably tri-oxidised **[1a(Au)3]3+** (**VII** to **VIII**) leads to an even larger reaction rate (TOF = 4.3 ± 0.2 h^−1^). The **ON**-switching can also be used on a macroscopic level, to which end two 5 mL-scale reactions (57 mg **5**, 2.2 mg **[1a(Au)3]**, *i.e.*, 1 mol% Au, [**5**]_0_ = 60 mmol L^−1^) were compared. One run was oxidised using 3.0 eq. **8** at 12.5 h after the start and led to a quantitative yield *vs.* only 20% conversion for the non-switched case after 42.0 h (29.5 h after the **ON**-switch).^129^

These findings are invariant to the order of addition, that is, the presence of substrate during oxidation;^130^ when **[1a(Au)3]** is oxidised with 1–3 equivalents of **8** before the addition of **5**, similar reaction profiles and TOF result (Fig. S26, Table S13†). In all cases, the first **ON**-switch is accompanied by an induction period which is absent for the re-oxidation (*e.g*., **IV** to **V***vs.***VI** to **VII** in Fig. 5). When **[1b(Au)3]** (red triangles) is used in the cyclisation (Fig. 5, bottom left) and oxidised using two equivalents of **8**, the same behaviour, including a similar TOF (2.3 ± 0.1 h^−1^) as for **[1a(Au)3]**, is found. Oxidising **[1c(Au)3]** (yellow triangles) with two equivalents of **8** converted the otherwise silent complex (*vide supra*) into an active catalyst with higher TOF (3.3 ± 0.1 h^−1^). Even though other redox-switchable gold(i) catalysts perform with sometimes significantly higher activity, complexes **[1a–c(Au)3]** were intended as models demonstrating the feasibility of tailorable multi-oxidation-state applications. Lower observed activities for **[1a–c(Au)3]** are hence an acceptable trade-off prior to further optimisation.^105,109^ In the same way, the significantly higher reaction rate of using oxidised **7** (1 mol% Au, TOF = 10.6 ± 0.4 h^−1^, Fig. S24†) comes at the expense of not being principally able to address separate states with differing catalytic activity.

In order to gain more insight into the switching process, the oxidation of **[1a(Au)3]** in the absence of substrate was first followed by ^1^H and ^31^P{^1^H} NMR spectroscopy (Fig. S27 and S28†). The ^31^P{^1^H} NMR resonance shifted upfield and broadened with each added equivalent of **8**, while the subsequent addition of **9** reversed this trend. In line with the findings from the catalytic runs, the reduction took quite long to take its full effect.

**Chart 2:**
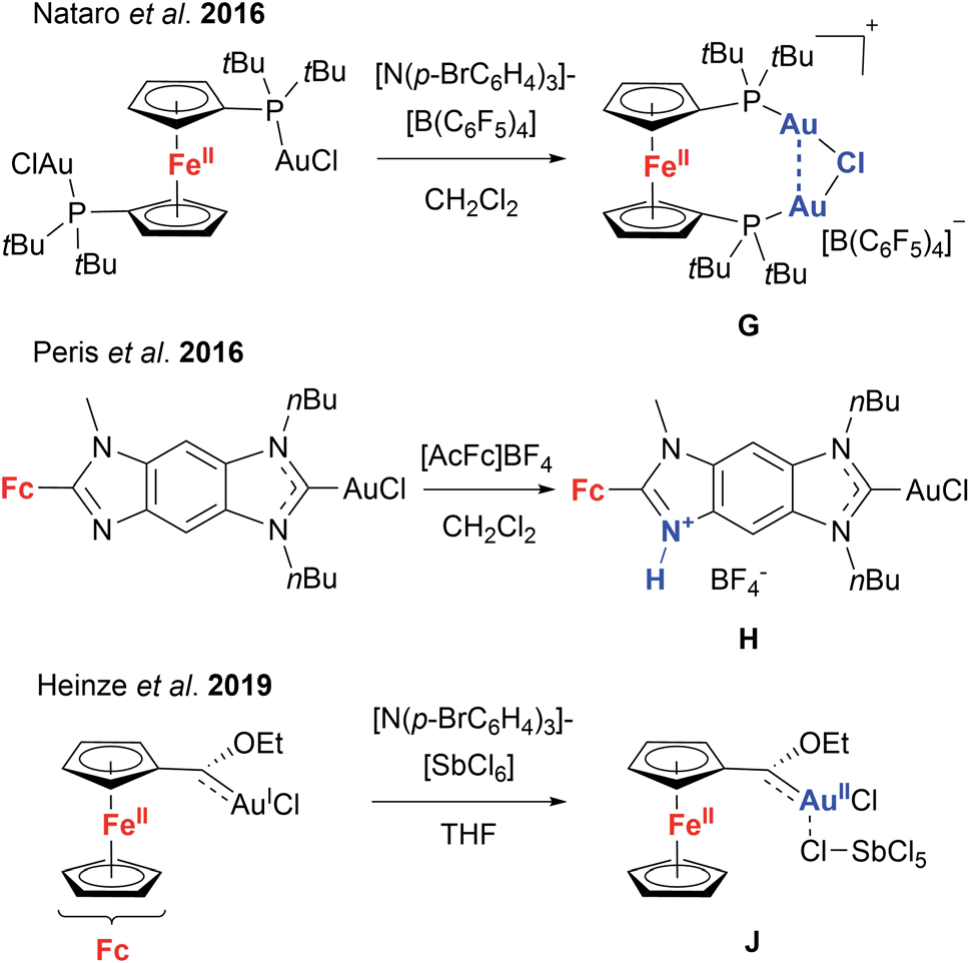
Behaviour of ferrocene-derived chloridogold(i) complexes upon chemical oxidation (compounds **G** and **H** have been characterised by XRD in the solid state, while information about the structure of **J** has been gathered from DFT calculations and various analytical techniques).

Given these encouraging results, we sought to isolate the individually switched complexes **[1a(Au)3](TEF)n** (*n* = 1–3), attempting to dismiss the possibility that oxidising **[1a(Au)3]** during the catalytic reaction (*in situ*) might lead to mixtures of different oxidation states (*e.g.* by disproportionation). Furthermore, we wanted to ascertain the integrity of the oxidised species, since Nataro and co-workers observed loss of a chlorido ligand (**G**, Chart 2) from dinuclear gold(i) complexes based on 1,1′-bis(phosphanyl)ferrocenes after chemical oxidation using tris(*p*-bromophenyl)ammoniumyl tetrakis(pentafluorophenyl)-borate (“Magic Blue”).^131^ While the group of Peris found oxidation-induced protonation (**H**, Chart 2) of the ferrocenyl-imidazolylidene backbone in their gold(i) chloride complexes upon addition of acetylferrocenium tetrafluoroborate,^126^ Heinze and co-workers described valence isomerisation from, initially, Fe^III^/Au^I^ to Fe^II^/Au^II^ (**J**, Chart 2) assisted by both the SbCl_6_^−^ anion of their oxidant and propargylic amide **5**.^105^ In the last two instances, the respective Au–Cl fragment was found to remain intact.

**Fig. 6:**
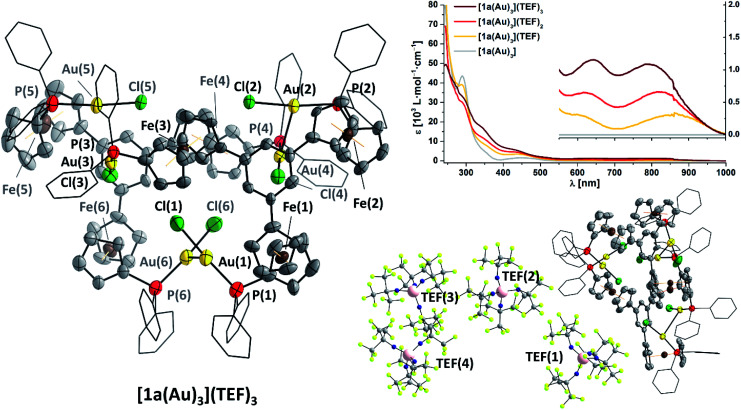
(Left) Molecular structure of **{[1a(Au)3]}2(TEF)6** with partial atom numbering scheme. Thermal ellipsoids are set at the 50% probability level. For clarity, *P*-bound phenyl rings are depicted in wireframe style, and co-crystallised solvent, hydrogen atoms, and anions have been omitted. (Bottom right) Asymmetric unit of **{[1a(Au)3]}2(TEF)6** with the four localisable teflonate anions in ball-and-stick style. The presence of severely disordered teflonate anions TEF(5) and TEF(6) has been confirmed through their electron density and the unoccupied volume. (Top right) Stacked UV/Vis spectra of **[1a(Au)3](TEF)n** (*n* = 0–3) in CH_2_Cl_2_. The inset shows a magnification for the absorptions at long wavelength.

Gratifyingly, **[1a(Au)3](TEF)n** can indeed by obtained as analytically pure compounds from the reaction of **[1a(Au)3]** with 1, 2, or 3 equivalents of **8** as evidenced by CHN analyses. While **[1a(Au)3](TEF)** is a pale green powder, both **[1a(Au)3](TEF)2** and **[1a(Au)3](TEF)3** are dark-green microcrystalline solids. In contrast to **[1a(Au)3]**, they are readily soluble in diethyl ether which allows for their purification by precipitation with pentanes, as 1,1′-diacetylferrocene is soluble under these conditions and can hence be extracted. The effective magnetic moments *μ*_eff_ of **[1a(Au)3](TEF)n** in solution were determined using Evans' method (Fig. S99, S103 and S107†) and match the expected spin-only values for one (2.03 *μ*_B_, expected: 1.73 *μ*_B_), two (2.98 *μ*_B_, expected: 2.83 *μ*_B_), and three (3.52 *μ*_B_, expected: 3.82 *μ*_B_) unpaired electrons reasonably well.^132,133^ The ^1^H and ^31^P{^1^H} NMR-spectroscopic features (Fig. S98–S109†) match those of the previously mentioned stepwise oxidation (Fig. S27 and S28†). HR-ESI mass spectra show peaks for the three different cations respectively, the tri-cation **[1a(Au)3]3+** apparently stabilised by a tight contact to the teflonate anion as **{[1a(Au)3](TEF)}2+** under these conditions; similarly, the ^19^F resonance of **[1a(Au)3](TEF)3** (*ω*_1/2_ = 17 Hz) is slightly broadened with respect to that of the mono- and dioxidised species (*ω*_1/2_ = 4 Hz). IR spectra (Fig. S29†) of **[1a(Au)3](TEF)n** display both the signature of the teflonate anion and a band at 860 cm^−1^, characteristic for the *δ*(C–H) vibration of ferrocenium.^134,135^ In line with previous reports,^136^ the ferrocenylene *ν*(C–H) stretches shift to higher wavenumbers upon increasing degree of oxidation and do not indicate the presence of native or lower oxidation states as expected for disproportionation. Neither can this be inferred from UV/Vis spectra of **[1a(Au)3](TEF)n** (Fig. 6 top right, Fig. S30†), as they differ in the position of their bands at long wavelength, most likely related to LMCT, inner ferrocenyl, and potentially even to Au^I^–Fe^III^ MMCT transitions,^106,137,138^ between 500 and 1000 nm.

As final proof for the site of oxidation, we were able to isolate single crystals of the tri-oxidised complex as **{[1a(Au)3]}2(TEF)6** (Fig. 6, left and bottom right), thus unambiguously confirming the oxidation state of three times Fe^III^ through the presence of six, albeit only partly crystallographically describable, teflonate anions per asymmetric unit. Notably, all three P–Au–Cl fragments are still intact and form dimers of an overall sixfold positive charge through a set of two aurophilic interactions (*d*(Au(1)–Au(6)) = 2.989(1) Å, *d*(Au(2)–Au(4)) = 3.188(1) Å) and a less-close Au⋯Au contact (*d*(Au(3)⋯Au(5)) = 3.397(1) Å).^139^ While the poor crystal quality precludes detailed metric analyses, generally larger C_5_H_4_(centroid)–Fe distances of over 1.68 Å and, accordingly, Fe–C bond lengths of up to 2.15(2) Å, typical for ferrocenium species,^136,140^ are discernible (Table S8†). Other structural parameters, particularly with respect to the P–Au–Cl moieties are, within error, comparable to the molecular structure of **[1c(Au)3]**. While ferrocenium cations are frequently encountered as counter cations for metallate anions and several homo- and heteromultinuclear bridged metallocenes featuring ferrocenium units have been listed in the CSD, **{[1a(Au)3]}2(TEF)6** is a very rare example of a crystallographically characterised metal complex containing ferrocenium in its ligand backbone. To the best of our knowledge, a rhenium(0) carbonyl complex by Nataro and co-workers is the only other example to contain both ferrocenium and a phosphane-bound metal complex fragment,^141^ while the +III oxidation state of iron in reported bis[1,1′-bis(diphenylphosphanyl)ferrocenium]hexadecachlorotetraantimonate(iii) by the group of Kasim^142^ has been questioned by both Nataro and Connick.^143^ Not backed up by a solid-state molecular structure, Grandberg and co-workers also reported a phosphanyl ferrocenium-based gold(iii) bromide complex in 1977.^144^ Last but not least, a very recent report by the Lapinte group details a related tetranuclear iron(ii/iii) half-sandwich array with potential use in molecular electronics.^145^

**[1a(Au)3](TEF)n** appear to be air- and moisture-insensitive in the solid state for short periods. They can be weighed out in ambient conditions but quickly turn from green to yellow solutions in wet solvents. Their stability in dry CD_2_Cl_2_ was monitored by ^1^H and ^31^P{^1^H} NMR spectroscopy (Fig. S31–S33†). Surprisingly, a solution of **[1a(Au)3](TEF)3** did show little change over three days while **[1a(Au)3](TEF)** and **[1a(Au)3](TEF)2** slowly decomposed under the same conditions (room temperature, protected from light). ^31^P{^1^H} resonances attributable to *P*,*P*′-dicoordinate species at around 40 ppm appeared, and **[1a(Au)3](TEF)2** produced a metallic mirror of presumably elemental gold on the wall of the NMR tube. Furthermore, the ^1^H NMR spectral evolution of **[1a(Au)3](TEF)2** (Fig. S32†) indicates at least some degree of disproportionation to the mono- and trioxidised species, making it the least solution-stable of the three isolated oxidation products. Although a mass spectrum of the crystals of **[1a(Au)3](TEF)3** does not contain signals for dimeric species, aurophilic interactions in solution might play a role in stabilising the trication. When fresh solutions of **[1a(Au)3](TEF)n** (*n* = 1–3) are reacted with one, two, or three equivalents of reductant **9**, native **[1a(Au)3]** is re-obtained respectively (Fig. S34–S36†).

The isolated oxidised species **[1a(Au)3](TEF)n** have finally also been tested for their catalytic performance (*ex situ*; Fig. 5, bottom right). Their TOF show the previously noted distinct dependence on the degree of oxidation, with tri-oxidised **[1a(Au)3](TEF)3** (light blue circles, 5.8 ± 0.1 h^−1^) performing with about threefold activity than mono-oxidised **[1a(Au)3](TEF)** (navy squares, 1.8 ± 0.1 h^−1^). All of them outperform the *in situ*-oxidised species, most notably **[1a(Au)3](TEF)3**. This points towards redox equilibria between **8** and the complexes in higher oxidation states which might be overcome using stronger oxidants, thus ensuring full conversion upon addition. Even though we cannot fully exclude potential disproportionation or the presence of mixtures under these catalytic conditions, the distinctly different activities in the read-out catalytic conversion strongly suggest that **[1a(Au)3]** and its analogues can function as molecular switches with four addressable states.

Similar to the *in situ*-generated species, induction periods are observed for **[1a(Au)3](TEF)n**. Notably, the addition of a fresh batch of **5** to an almost-completed reaction after 24 h (Fig. S37†) using **[1a(Au)3](TEF)3** did not result in another induction period but led to a slight loss of activity (3.2 ± 0.1 *vs.* 5.0 ± 0.1 h^−1^). Mixing isolated **[1a(Au)3](TEF)3** with **5** (Fig. S38†) in CH_2_Cl_2_ led to an appreciably slow colour change which was followed by time-resolved UV/Vis spectroscopy. We thus speculate that the catalytically active species are, in general, formed from a chemical process involving **5**. Bearing in mind the aforementioned results from Heinze and co-workers and in accordance with loss of the Fe^III^-associated absorptions of **[1a(Au)3](TEF)3** at long wavelength,^105^ a coordination-assisted valence isomerisation from Fe^III^/Au^I^ to Fe^II^/Au^II^ might be at the heart of this behaviour.

## Conclusions

In summary, we have demonstrated the modular syntheses of a new class of tris(ferrocenyl)arene-based tris-phosphanes **1** which can be used to form well-defined, *C*_3_-symmetric homotrinuclear gold(i) complexes. Four oxidation states relating to the tris(ferrocenyl)arene backbone – non-, mono-, di-, and tri-oxidised – have been identified by cyclic voltammetry. Stoichiometric oxidation of **[1a(Au)3]** produces isolable products **[1a(Au)3](TEF)n**. This redox behaviour can be advantageously used in redox-switchable catalysis *ex* and *in situ*, as we were able to show for the proof-of-principle ring-closing isomerisation of *N*-(2-propyn-1-yl)benzamide (**5**) forming oxazoline **6**. The arene cores determine the exact redox potential and were also found to influence the catalytic performance of the native and oxidised species, a feature which we are currently investigating in more detail. Metal complexes of **1** and its analogues thus hold great promise for applications in molecular electronics and logic, possibly extending binary to quaternary signal processing, as four different oxidation states can be addressed and isolated.

## Conflicts of interest

There are no conflicts to declare.

## Supplementary Material

SC-011-D0SC03604H-s001

SC-011-D0SC03604H-s002
