# Supplementary material for: Triple the fun: tris(ferrocenyl)arene-based gold(i) complexes for redox-switchable catalysis
Source: Chem Sci. 2020 Aug 3;11(39):10657–68. doi: 10.1039/d0sc03604h (PMC8162263; doi:10.1039/d0sc03604h)
Supplement: SC-011-D0SC03604H-s001 [file SC-011-D0SC03604H-s001.pdf]

Electronic Supplementary Material (ESI) for Chemical Science.
This journal is © The Royal Society of Chemistry 2020

SUPPORTING INFORMATION

Supporting Information

Triple the fun: Tris(ferrocenyl)arene-based gold(I) complexes for redox-switchable catalysis

Axel Straube,[a] Peter Coburger,[a] Luis Dütsch,[b] and Evamarie Hey-Hawkins*[a]

Abstract: The modular syntheses of C_3-symmetric tris(ferrocenyl)arene-based tris-phosphanes and their homotrinuclear gold(I) complexes are reported. Choosing the arene core allows to fine-tune the exact oxidation potentials and thus to tailor the electrochemical response. The tris[chloridogold(I)] complexes were investigated in the catalytic ring-closing isomerisation of *N*-(2-propyn-1-yl)benzamide, showing cooperative behaviour *vs.* a mononuclear chloridogold(I) complex. Adding one, two, or three equivalents of 1,1'-diacetylferrocenium[tetrakis(perfluoro-*tert*-butoxy)aluminate] as an oxidant during the catalytic reaction (*in situ*) resulted in a distinct, stepwise influence on the resulting catalytic rates. Isolation of the oxidised species is possible and using them as (pre-)catalysts (*ex situ* oxidation) confirmed the activity trend. Proving the intactness of the P–Au–Cl motif during oxidation, the tri-oxidised benzene-based complex has been structurally characterised.

DOI:

SUPPORTING INFORMATION

Table of Contents

1. Syntheses and characterisation 3
    1.1. General information 3
    1.2. Synthetic procedures 4
        1.2.1. 1,3,5-Tris(1-bromo-1'-ferrocenylene)benzene (**4a**) – Typical procedure for the synthesis of tris(1-bromo-1'-ferrocenylene)arenes (**4a**–**e**) 4
        1.2.2. 2,4,6-Tris(1-bromo-1'-ferrocenylene)-1,3,5-trifluorobenzene (**4b**) and 1-bromo-3,5-bis(1-bromo-1'-ferrocenylene)-2,4,6-trifluorobenzene (**SP3**) 4
        1.2.3. 1,3,5-tris{(1-bromo-1'-ferrocenylene)methyl}benzene (**4d**) and 1',1''''-dibromo-1,1'':1''',1''''-terferrocene (**SP1**) 5
        1.2.5. 1,3,5-Tris(1-diphenylphosphanyl-1'-ferrocenylene)benzene (1a) – General procedure for the synthesis of 1,3,5-(1-diphenylphosphanyl-1'-ferrocenylene)arenes (**1a**–**d)** 6
        1.2.6. 2,4,6-Tris(1-diphenylphosphanyl-1'-ferrocenylene)-1,3,5-trifluorobenzene (**1b**) 7
        1.2.7. 1,3,5-Tris[(1-diphenylphosphanyl-1'-ferrocenylene)methyl}benzene (**1d**) 7
        1.2.8. 1,3,5-Tris[1-(diphenylphosphanyl borane)-1'-ferrocenylene]benzene (**[1a(BH$_3$)$_3$]**) 8
        1.2.9. 2,4,6-Tris[1-(diphenylphosphanyl borane)-1'-ferrocenylene]-1,3,5-trifluorobenzene (**[1b(BH$_3$)$_3$]**) 8
        1.2.10. μ$_3$-[2,4,6-Tris(1-diphenylphosphanyl-1'-ferrocenylene)-1,3,5-triazine-1κ^1*P*,2κ^1*P*,3κ^1*P*}-tris[chloridogold(I)] (**[1c(Au)$_3$]**) 9
        1.2.11. μ$_3$-[1,3,5-Tris(1-diphenylphosphanyl-1'-ferrocenylene)benzene-1κ^1*P*,2κ^1*P*,3κ^1*P*]-tris[chloridogold(I)] (**[1a(Au)$_3$]**) 9
        1.2.12. μ$_3$-[2,4,6-Tris(1-diphenylphosphanyl-1'-ferrocenylene)-1,3,5-trifluorobenzene-1κ^1*P*,2κ^1*P*,3κ^1*P*]-tris[chloridogold(I)] (**[1b(Au)$_3$]**) 10
        1.2.13. 1,1'-Diacetylferrocinium teflonate (**8**) 10
        1.2.14. Monooxidised complex **[1a(Au)$_3$](TEF)** 10
        1.2.15. Dioxidised complex **[1a(Au)$_3$](TEF)$_2$** 11
        1.2.16. Trioxidised complex **[1a(Au)$_3$](TEF)$_3$** 11
    1.3. Catalytic tests 12
2. Single Crystal X-Ray Diffraction Analyses 13
    2.1. Crystallographic data 13
    2.2. Structural parameters 14
3. Electrochemical data 23
4. DFT Calculations 28
5. Catalysis 30

Author Contributions 48
References 48
NMR Spectra 50
DFT Coordinates for **[1a(Au)$_3$]**/**[1a(Au)$_3$]$^+$** 78

SUPPORTING INFORMATION

1. Syntheses and characterisation

1.1. General information

Materials and methods

All reactions and manipulations were carried out under an atmosphere of either nitrogen or argon using standard Schlenk line techniques unless stated otherwise. Thin-layer chromatography (TLC) with silica gel 60 F254 on glass or aluminium sheets available from Merck KGaA was used for monitoring the tris-phosphane synthesis reactions. Column chromatography was performed using a Biotage Isolera 1 automatic purification system with SNAP (silica, particle diameter: 0.040 to 0.065 mm) and SNAP Ultra (silica, spherical particle, diameter: 0.025 mm) cartridges using solvents purged with nitrogen prior to use. The fractions were detected by an integrated UV/Vis detector.

Molecular sieves (4 Å) were activated at 300 °C in vacuo for a minimum of 3 h. Dry, oxygen-free solvents (THF, CH_2Cl_2, Et_2O, hexanes, and toluene) were obtained from an MBraun Solvent Purification System MB SPS-800 and directly stored over 4 Å molecular sieves, except for THF, which was further distilled from potassium/benzophenone and stored over 4 Å molecular sieves.

For the use in NMR measurements, CD_2Cl_2 and $CDCl_3$ were dried by stirring over P_2O_5 at room temperature for several days, followed by vacuum transfer into a storage flask and degassing by the freeze-pump-thaw method. The solvents were stored over 4 Å molecular sieves.

[P(*t*Bu)$_2$C(CH$_3$)$_2$CH$_2$Pd(μ-Cl)]$_2$,[1] tetrakis(triphenylphosphane)palladium(0),[2] 1,3,5-triiodobenzene,[3] 1,3,5-triiodo-2,4,6-mesitylene,[4] 1,3,5-tribromo-2,4,6-trifluorobenzene,[5] 1,3,5-tris(bromomethyl)benzene,[6] 1,1'-dibromoferrocene,[7] Na[B{3,5-(CF$_3$)$_2$C$_6$H$_3$}$_4$] (NaBAr$^F_4$),[8] (*n*Bu$_4$N)[B{3,5-(CF$_3$)$_2$C$_6$H$_3$}$_4$] ((*n*Bu$_4$N)BAr$^F_4$),[9] [AuCl(tht)] (tht = tetrahydrothiophene),[10] Ag[Al{OC(CF$_3$)$_4$}] (Ag[teflonate]),[11] and *N*-prop-2-ynylbenzamide (**5**)[12] were synthesised as described in the literature. Ph_2PCl was distilled and stored under nitrogen prior to use. Tris-phosphane **1c** was prepared according to our previously published procedure.[13] All other chemicals were used as purchased.

NMR spectra were recorded with a BRUKER Avance III HD 400 MHz NMR spectrometer at 25 °C (frequencies of ^{1}H: 400.13 MHz; ^{11}B 128.38 MHz; ^{13}C 100.63 MHz; ^{19}F: 376.53 MHz; ^{27}Al: 104.26; ^{31}P: 161.99 MHz) unless indicated otherwise. Pseudo-triplets and -quadruplets (due to additional coupling to heteronuclei like ^{19}F and ^{31}P) of ferrocenyl protons are abbreviated as pt or pq and their observable coupling constants *J* are given. Assignment of ^{1}H and ^{13}C signals to the respective chemical entities are based on ^{1}H,^{1}H COSY, phase-sensitive ^{1}H,^{13}C HSQC and ^{1}H,^{13}C HMBC NMR experiments. TMS was used as the internal standard in the ^{1}H and ^{13}C NMR spectra, and spectra of all other nuclei were referenced to TMS using the Ξ scale.[14] For the determination of the effective magnetic moments by Evans' NMR experiment,[15] a defined amount of sample was weighed in and dissolved in 0.6 mL of CD_2Cl_2/CH_2Cl_2 (50:1 v/v), a sealed capillary with the same solvent mixture but without sample also present in the NMR tube. The diamagnetic contribution of parent complex **[1a(Au)$_3$]** was determined through the same experiment. Diamagnetic contributions for the anions were approximated as described by Bain and Berry.[16]

Electrospray ionisation (ESI) mass spectrometry was performed with an ESI ESQUIRE 3000 PLUS spectrometer with an IonTrap analyser from Bruker Daltonics, or a MicroTOF spectrometer from Bruker Daltonics with a ToF analyser in positive mode. As solvents for the measurements, pure CH_2Cl_2 or mixtures of CH_2Cl_2 and CH_3CN were used. Dry, oxygen-free solvents were used for air-sensitive species. Elemental analyses were performed with a VARIO EL elemental analyser from Heraeus. Melting points were determined with a Gallenkamp MPD350·BM2.5 melting point device and are reported uncorrected. FTIR spectra were obtained with a PerkinElmer FT-IR spectrometer Spectrum 2000 as KBr pellets and with a Thermo Scientific Nicolet iS5 with an ATR unit in the range from 4000 to 400 cm^{-1}. UV/Vis spectra were recorded on a PerkinElmer UV/VIS-NIR Lambda 900 spectrometer in quartz cuvettes (d = 10 mm) or double-chamber cuvettes (d = 2 x 4.37 mm). Sample concentration was in the range of $3 \cdot 10^{-5}$ mol·L^{-1}.

Crystallography

The data were collected on a Gemini-CCD diffractometer (RIGAKU INC.) using Mo-K$_\alpha$ radiation (λ = 0.71073 Å), ω-scan rotation. Data reduction was performed with CrysAlis Pro[17] including the program SCALE3 ABSPACK[18] for empirical absorption correction. The structure solution and anisotropic full-matrix least-squares refinement on F^2 of all non-hydrogen atoms for **4a**, **4b**, **4d**, **4e**, **SP1**, **SP2**, **SP3**, **1a**, **1d**, **[1a(BH$_3$)$_3$]**, and **[1c(Au)$_3$]** was performed with SHELXS-97 (direct methods).[19] The structure solution and anisotropic full-matrix least-squares refinement on F^2 of all non-hydrogen atoms for **[1a(Au)$_3$](TEF)$_3$** was performed with SHELXT-2018 (dual-space method).[20] Except for disordered solvent molecules, all non-hydrogen atoms were refined with anisotropic thermal parameters and the HFIX command was used to locate all hydrogen atoms for non-disordered regions of the structure. Structure figures were generated with Mercury (versions 3.8 and 3.10).[21] CCDC 1990272 (**4a**), 1990274 (**4b**), 1990276 (**4d**), 1990282 (**4e**), 1990277 (**SP1**), 1990275 (**SP2**), 1990281 (**SP3**), 1990273 (**1a**), 1990278 (**1d**), 1990283 (**[1a(BH$_3$)$_3$]**), 1990279 (**[1c(Au)$_3$]**), 2012019 (**{[1a(Au)$_3$]}$_2$(TEF)$_6$**) contain the supplementary crystallographic data for this paper. These data are provided free of charge by The Cambridge Crystallographic Data Centre.

SUPPORTING INFORMATION

Electrochemistry

Cyclic voltammetry (CV) measurements on 1.0 mmol·L^{-1} analyte solutions in dry, oxygen-free dichloromethane containing 0.1 mol·L^{-1} (nBu$_4$N)BF$_4$ or (nBu$_4$N)BAr$^F_4$ as supporting electrolyte have been conducted in a three-electrode setup (GAMRY Instruments, SP-50 potentiostat by BioLogic Science Instruments) under a blanket of nitrogen at room temperature. The glassy-carbon working electrode (ALS; surface area 0.07 cm^2) and the counter electrode (neoLab; platinum wire, 99.9%) were immersed in the analyte solution, while the reference electrode (ALS; Ag/AgNO$_3$ (0.01 mol·L^{-1}) in 0.1 mol·L^{-1} tetrabutylammonium hexafluorophosphate in dry, oxygen-free CH_3CN) was connected to the cell via a bridge tube (filled with the supporting electrolyte) through Vycor tips. The reference electrode was calibrated against decamethylferrocene as an internal standard at the end of the CV experiment,[22] and the results were converted to the FcH/[FcH]$^+$ scale in accordance with the IUPAC requirements.[23]

Computational methods

All calculations were carried out with the ORCA program package.[24,25] All geometry optimisations were performed at the BP86-D3BJ/def2-TZVP[26–30] level of theory in the gas phase. For gold, the respective effective core potentials, namely def2-ECP,[31] were used. Frequency calculations were carried out to confirm the nature of stationary points found by geometry optimisations.

1.2. Synthetic procedures

NMR spectra for all compounds described in the following, including spectra for Evans' NMR experiment, can be found at the end of this document.

1.2.1. 1,3,5-Tris(1-bromo-1'-ferrocenylene)benzene (4a) – Typical procedure for the synthesis of tris(1-bromo-1'-ferrocenylene)arenes (4a–e)

A stirred solution of 4.00 g (11.6 mmol, 3.30 eq.) 1,1'-dibromoferrocene (**2**) in 35 mL THF was cooled to −80 °C (ethyl acetate/$N_{2(l)}$), and 7.3 mL nBuLi in n-hexane (1.53 mol·L^{-1}, 11 mmol, 3.15 eq.) were added dropwise over the course of 10 min. A dark-orange precipitate formed, and the solution was kept stirring at this temperature for a further 30 min. A freshly prepared solution of 1.66 g (12.2 mmol, 3.45 eq.) zinc chloride in 12 mL THF was added, the clear solution was warmed to room temperature and stirred for a further 2 hours. In a separate flask, 1.61 mg (3.53 mmol, 1.00 eq.) 1,3,5-triiodobenzene (**3a**) and 36 mg (53 µmol, 1.5 mol%) [P(tBu)$_2$C(CH$_3$)$_2$CH$_2$Pd(µ-Cl)]$_2$ were dissolved in 15 mL THF and transferred to the reaction mixture (immersed in a water bath due to the exothermic reaction upon addition) *via* cannula, immediately followed by a darkening of the red colour. The reaction mixture was left stirring at room temperature overnight. TLC (CH_2Cl_2/hexanes, 1:1) then indicated the complete conversion of the aryl halide; all further work-up was carried out under ambient conditions.

For quenching, 60 mL of brine were added, and the phases were separated. The aqueous phase was extracted with ethyl acetate (2x 40 mL), the combined organic phases were dried over Na_2SO_4, followed by filtration over neutral alumina using ethyl acetate as the eluent. Column chromatography (CH_2Cl_2/ hexanes, gradient 23:1 to 2:1) and subsequent evaporation of all volatiles gave **4a** (2.24 g, 72%) as an orange microcrystalline solid. Crystals suitable for XRD were obtained by recrystallisation from CH_2Cl_2/hexanes 1:1 v/v.

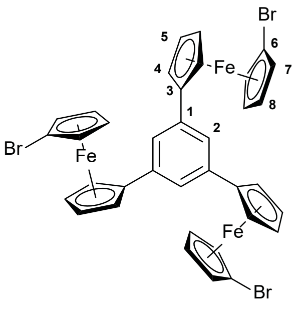

Mp: 166–167 °C (from CH_2Cl_2/hexanes). R$_f$: 0.71 (CH_2Cl_2/ hexanes 1:1). Anal. calcd. for $C_{36}H_{27}Br_3Fe_3$: C 49.88, H 3.14, found: C 50.68, 3.10%. IR (KBr, ṽ): 3095 (m), 2954 (m), 2928 (m), 2860 (w), 2835 (w, all ν(C–H)), 1656 (w), 1639 (w), 1631 (w), 1593 (s, ν(C–C)), 1558 (w), 1550 (w), 1507 (w), 1498 (w), 1457 (w), 1408 (m), 1383 (m), 1350 (m), 1317 (w), 1205 (w), 1150 (m), 1105 (m), 1095 (m), 1053 (w), 1036 (s, δ(C$_5$H$_4$–Br)), 922 (m), 868 (s), 811 (vs), 728 (m), 691 (m, δ(C–H)), 493 (s), 473 (m), 436 (m), 418 (w) cm^{-1}. ^{1}H NMR (CD_2Cl_2, δ): 7.57 (3H, s, H2), 4.78 (6H, pt, J_{HH} = 1.9 Hz, H4/5), 4.46 (6H, pt, J_{HH} = 1.9 Hz, H4/5), 4.26 (6H, pt, J_{HH} = 1.9 Hz, H7/8), 4.10 (6H, pt, J_{HH} = 1.9 Hz, H7/8) ppm. ^{13}C{^{1}H} NMR (CD_2Cl_2, δ): 138.1 (C1), 122.6 (C2), 87.4 (C3), 78.8 (C6), 72.2 (C7/8), 71.5 (C4/5), 69.5 (C4/5), 68.8 (C7/8) ppm. HRMS (ESI-TOF, m/z): Calcd. for $C_{36}H_{27}Br_3Fe_3$ 865.7693; found 865.7683 [M]$^+$.

1.2.2. 2,4,6-Tris(1-bromo-1'-ferrocenylene)-1,3,5-trifluorobenzene (4b) and 1-bromo-3,5-bis(1-bromo-1'-ferrocenylene)-2,4,6-trifluorobenzene (SP3)

Compound **4b** was prepared following the standard procedure, using 650 mg 1,3,5-tribromo-2,4,6-trifluorobenzene (1.76 mmol, 1.00 eq.) and substituting the pre-catalyst for tetrakis(triphenylphosphane)palladium(0) (123 mg, 106 µmol, 6 mol%). Column chromatography (CH_2Cl_2/hexanes, gradient 24:1 to 2:1) gave the **4b** as a bright-orange microcrystalline material (870 mg, 54%). Crystals suitable for XRD were obtained by slow evaporation of a CH_2Cl_2/hexane (1:5 v/v) solution of **4b**.

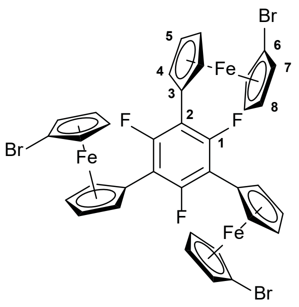

Mp: 124 °C (from CH_2Cl_2/hexanes). R_f: 0.18 (hexanes/CH_2Cl_2 5:1). Anal. calcd. for $C_{36}H_{24}Br_3F_3Fe_3$: C 46.96, H 2.63, found: C 47.25, H 2.33%. IR (KBr, ṽ): 3091 (m), 2954 (w), 2928 (w), 2860 (w), 2835 (w, all ν(C–H)), 1610 (m, ν(C–C)), 1487 (m), 1423 (m), 1410 (m), 1387 (m), 1350 (m), 1313 (m), 1230 (w), 1205 (m), 1150 (m), 1105 (m), 1060 (m), 1021 (vs, δ(C_5H_4–Br)), 914 (w), 870 (s), 810 (s), 760 (w), 697 (m), 681 (w, δ(C–H)), 497 (vs), 481 (vs), 450 (m), 420 (w) cm^{-1}. ^1H NMR (CD_2Cl_2, δ): 4.90 (6H, m, H4/5), 4.47 (6H, pt, J_{HH} = 1.9 Hz, H4/5), 4.41 (6H, pt, J_{HH} = 1.9 Hz, H7/8), 4.15 (6H, pt, J_{HH} = 1.9 Hz, H7/8) ppm. $^{13}C\{^1H\}$ NMR (CD_2Cl_2, δ): 155.8 (dt, $^1J_{CF}$ = 252.9 Hz, $^3J_{CF}$ = 11.1 Hz, C1), 111.2 (m, C2), 78.8 (s, C6), 76.0 (s, C3), 72.0 (m, C4), 71.5 (s, C7/8), 71.4 (*s,* C5), 68.8 (s, C7/8) ppm. $^{19}F\{^1H\}$ NMR (CD_2Cl_2, δ): −107.2 (s) ppm. HRMS (ESI-TOF, m/z): Calcd. for $C_{36}H_{24}Br_3F_3Fe_3$ 919.7411; found 919.7410 $[M]^+$.

Side product **SP3** was isolated as a red microcrystalline material (290 mg, 22% based on 1,3,5-tribromo-2,4,6-trifluorobenzene). Crystals suitable for XRD were obtained from a partially evaporated CH_2Cl_2/hexane solution at 7 °C overnight. Only limited analytical data (*vide infra*) has been acquired, since NMR spectra of **SP3** showed impurities.

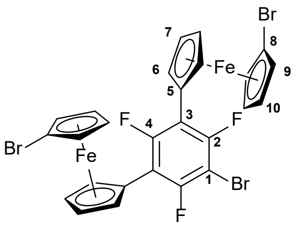

R_f: 0.30 (hexanes/CH_2Cl_2 5:1). ^1H NMR (CD_2Cl_2, δ): 4.90–4.81 (6H, m, H6/7), 4.47 (6H, pt, J_{HH} = 1.9 Hz, H6/7), 4.40 (6H, pt, J_{HH} = 1.9 Hz, H9/10), 4.12 (6H, pt, J_{HH} = 1.9 Hz, H9/10) ppm. $^{13}C\{^1H\}$ NMR (CD_2Cl_2, δ): 156.7 (dt, $^1J_{CF}$ = 253.3 Hz, $^3J_{CF}$ = 9.6 Hz, C4), 154.8 (ddd, $^1J_{CF}$ = 248.8 Hz, $^3J_{CF}$ = 11.3, 6.4 Hz, C2), 112.4 (td, $^2J_{CF}$ = 19.1, $^4J_{CF}$ = 3.7 Hz, C3), 94.8 (td, $^2J_{CF}$ = 26.3, $^4J_{CF}$ = 4.5 Hz, C1), 78.7 (s, C8), 75.0(s, C5), 71.7 (m, 2x C_4H_4), 71.5 (*s,* C6/7), 68.8 (s, C9/10) ppm. $^{19}F\{^1H\}$ NMR (CD_2Cl_2, δ): −103.7 (2F, d, $^4J_{FF}$ = 5.5 Hz), −105.5 (1F, t, J $^4J_{FF}$ = 5.5 Hz) ppm. HRMS (ESI-TOF, m/z): Calcd. for $C_{26}H_{16}Br_3F_3Fe_2$ 735.7432; found 735.7369 $[M]^+$.

1.2.3. 1,3,5-tris{(1-bromo-1'-ferrocenylene)methyl}benzene (4d) and 1',1'''''-dibromo-1,1'':1''',1''''-terferrocene (SP1)

Compound **4d** was prepared following the standard procedure, using 724 mg 1,3,5-tris(bromomethyl)benzene (2.03 mmol, 1.00 eq.) and substituting $[P(tBu)_2C(CH_3)_2CH_2Pd(\mu\text{-Cl})]_2$ for dichloridobis(triphenylphosphane)palladium(II) (41 mg, 58 µmol, 3 mol%). Column chromatography (hexanes/$CHCl_3$, gradient 9:2 to 3:1) gave **4d** as a yellow microcrystalline material (1.09 g, 60%). Crystals suitable for XRD were obtained by slow evaporation of a solution of **4d** in CH_2Cl_2/hexanes (1:1 v/v).

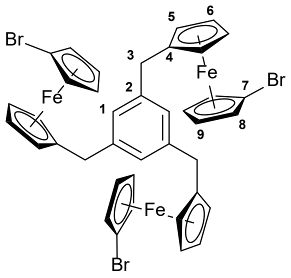

Mp: 101–102 °C (from $CHCl_3$/hexanes). R_f: 0.27 (hexanes/CH_2Cl_2 2:1). Anal. calcd. for $C_{39}H_{33}Br_3Fe_3$: C 51.54, H 3.66, found: C 51.59, H 3.62%. IR (KBr, ṽ): 3096 (w), 3085 (w), 3011 (w), 2943 (w), 2921 (w, all ν(C–H)), 1599 (m), 1457 (w), 1449 (w), 1439 (w), 1420 (w), 1405 (m), 1394 (w), 1377 (w), 1345 (m), 1308 (w), 1294 (w), 1243 (m), 1228 (m), 1203 (w), 1183 (w), 1147 (m), 1053 (w), 1038 (m), 1024 (s, δ(C_5H_4–Br)), 1007 (m), 984 (m), 928 (m), 910 (w), 895 (w), 864 (s), 846 (m), 833 (m), 812 (s), 800 (s), 764 (w), 738 (m), 675 (m), 624 (w), 586 (w) cm^{-1}. ^1H NMR (CD_2Cl_2, δ): 6.83 (3H, s, H1) 4.31 (6H, pt, J_{HH} = 1.9 Hz, H8/9), 4.13 (6H, pt, J_{HH} = 1.9 Hz, H5/6), 4.09 (6H, pt, J_{HH} = 1.9 Hz, H5/6), 4.03 (6H, pt, J_{HH} = 1.9 Hz, H8/9), 3.61 (6H, s, H3) ppm. $^{13}C\{^1H\}$ NMR (CD_2Cl_2, δ): 141.5 (C2), 126.2 (C1), 89.9 (C4), 78.1 (C7), 71.1 (C5/6), 70.7 (C8/9), 70.2 (C5/6), 67.8 (C8/9), 34.9 (C3) ppm. HRMS (ESI-TOF, m/z): Calcd. for $C_{39}H_{33}Br_3F_3Fe_3$ 907.8163; found 907.8161 $[M]^+$.

Side product **SP1** was obtained as one of many impure fractions of column-chromatographic purification when the synthesis of **4d** was attempted with $[P(tBu)_2C(CH_3)_2CH_2Pd(\mu\text{-Cl})]_2$ as the pre-catalyst. A few crystals of **SP1** suitable for XRD were obtained by evaporation of a CD_2Cl_2 solution. As the sample was found to be very impure, only a ^1H NMR spectrum was recorded (s. annex). The synthesis of **SP1** has been reported before, yet no spectroscopic data are available for comparison.[32]

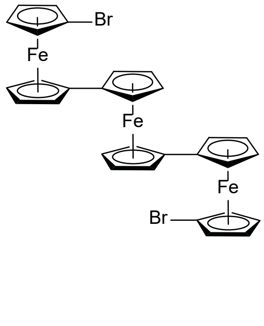

^1H NMR (CD_2Cl_2, δ): 4.26 (4H, pt, J_{HH} = 1.9 Hz), 4.23 (4H, pt, J_{HH} = 1.9 Hz), 4.20 (4H, pt, J_{HH} = 1.9 Hz), 4.12 (4H, pt, J_{HH} = 1.9 Hz), 4.09 (4H, pt, J_{HH} = 1.9 Hz), 3.91 (4H, pt, J_{HH} = 1.9 Hz) ppm.

SUPPORTING INFORMATION

1.2.4. 1,3,5-Tris(1-bromo-1'-ferrocenylene)-2,4,6-mesitylene (4e) and 1,1'-Bis{3,5-bis(1-bromo-1'-ferrocenylene)-2,4,6-trimethylphenyl}ferrocene (SP2)

Compound **4e** was prepared following the standard procedure, using 210 mg 1,3,5-triiodo-2,4,6-mesitylene (421 µmol, 1.00 eq.) and 9.0 mg of $[P(tBu)_2C(CH_3)_2CH_2Pd(\mu\text{-}Cl)]_2$ (13 µmol, 3 mol%). Column chromatography (CH_2Cl_2/hexanes, gradient 24:1 to 2:1) gave **4e** as an orange microcrystalline material (235 mg, 61%). Crystals suitable for XRD were obtained by slow evaporation of a solution of **4e** in CH_2Cl_2/hexanes (1:1 v/v).

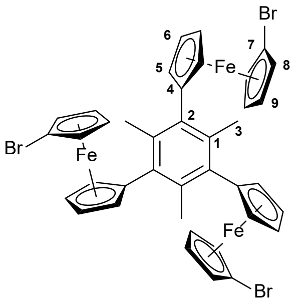

Mp: 82 °C (decomp.; from CH_2Cl_2/hexanes). R_f: 0.15 (hexanes/ CH_2Cl_2 5:1). Anal. calcd. for $C_{39}H_{33}Br_3Fe_3$: C 51.54, H 3.66, found: C 51.54, H 3.66%. IR (KBr, $\tilde{\nu}$): 3082 (w), 2963 (w), 2927 (w), 2873 (w, all ν(C–H)), 1446 (m), 1408 (m), 1382 (m), 1349 (m), 1314 (w), 1262 (w), 1215 (w), 1151 (m), 1105 (w), 1090 (w), 1046 (m), 1023 (s (br), δ(C_5H_4–Br)), 892 (m), 870 (s), 853 (m), 830 (s), 815 (s), 720 (w), 675 (w), 625 (w) cm^{-1}. ^1H NMR (CD_2Cl_2, δ): 4.44 (6H, pt, J_{HH} = 1.9 Hz, H8/9), 4.42 (6H, pt, J_{HH} = 1.9 Hz, H5/6), 4.35 (6H, pt, J_{HH} = 1.9 Hz, H5/6), 4.16 (6H, pt, J_{HH} = 1.9 Hz, H8/9), 2.64 (9H, s, H3) ppm. $^{13}C\{^1H\}$ NMR (CD_2Cl_2, δ): 135.9 (C1), 133.4 (C2), 90.1 (C4), 78.2 (C7), 74.1 (C5/6), 71.1 (C8/9), 70.8 (C5/6), 69.5 (C8/9), 22.8 (C3) ppm. HRMS (ESI-TOF, m/z): Calcd. for $C_{39}H_{33}Br_3F_3Fe_3$ 907.8163; found 907.8168 $[M]^+$.

Side product **SP2** was obtained as a minor fraction in column-chromatographic purification of **4e**, and no yield was determined. Crystals suitable for XRD were obtained by evaporation of a solution of **4e** in CH_2Cl_2/hexanes (1:1 v/v). Only limited analytical data has been recorded, as **SP2** was found to be impure by NMR spectroscopy despite crystallisation.

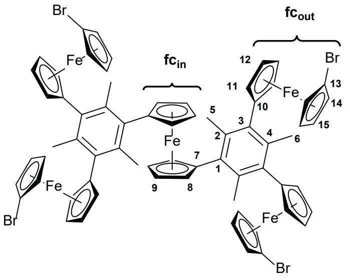

^1H NMR (CD_2Cl_2, δ): 4.48–4.50 (4H, m, fc_{in}), 4.39 (8H, pt, J_{HH} = 1.9 Hz, fc_{out}), 4.38 (8H, pt, J_{HH} = 1.9 Hz, fc_{out}), 4.32–4.34 (4H, m, fc_{in}), 4.27 (8H, pt, J_{HH} = 1.9 Hz, fc_{in}), 4.08 (8H, pt, J_{HH} = 1.9 Hz, fc_{in}), 2.69 (12H, s, H5), 2.46 (6H, s, H6) ppm. $^{13}C\{^1H\}$ NMR (CD_2Cl_2, δ): 136.5 (C1–4), 136.4 (C1–4), 134.1 (C1–4), 133.8 (C1–4), 90.8 (C10), 78.7 (C13), 74.7 (C8/9), 74.4 (fc_{out}), 71.5 (fc_{out}), 71.3 (fc_{out}), 70.0 (fc_{out}), 68.4 (C8/9), 23.6 (C6), 23.1 (C5) ppm. HRMS (ESI-TOF, m/z): Calcd. for $C_{68}H_{58}Br_4Fe_5$ 1473.7998; found 1473.8002 $[M]^+$.

1.2.5. 1,3,5-Tris(1-diphenylphosphanyl-1'-ferrocenylene)benzene (1a) – General procedure for the synthesis of 1,3,5-(1-diphenylphosphanyl-1'-ferrocenylene)arenes (1a–d)

A stirred solution of 2.36 g (2.72 mmol, 1.00 eq.) **4a** in 60 mL THF was cooled to −80 °C (ethyl acetate/$N_{2(l)}$) and 5.9 mL (1.53 mol·L^{-1}, 9.0 mmol, 3.3 eq.) *n*BuLi in *n*-hexane were added dropwise over the course of 30 min, resulting in a strong intensification of the orange colour. The mixture was kept stirring for 1.5 hours at −80 °C, followed by the dropwise addition of 2.07 g (9.39 mmol, 3.45 eq.) chlorodiphenylphosphane dissolved in 40 mL THF at the same temperature. After slowly warming to room temperature overnight and stirring at 60 °C for 1 hour, TLC (hexanes/ethyl acetate, 10:1) indicated full conversion of **4a**. For quenching, a degassed saturated aqueous solution of NH_4Cl (40 mL) was added to the fervently stirred mixture *via* cannula. The phases were separated, and the aqueous phase was extracted with diethyl ether (2x 20 mL). The combined organic phases were dried over degassed $MgSO_4$, filtered, and then the product mixture was adsorbed on Celite® and subjected to column chromatography (hexanes/ethyl acetate, gradient 99:1 to 6:1). Crystals suitable for XRD were obtained from a so-obtained fraction at 7 °C. The fractions were combined and the solvent removed under reduced pressure, **1a** was filtered over degassed silica using CH_2Cl_2, and the solvent was removed *in vacuo*, yielding **1a** (2.01 g, 62% yield) as an orange foam.

SUPPORTING INFORMATION

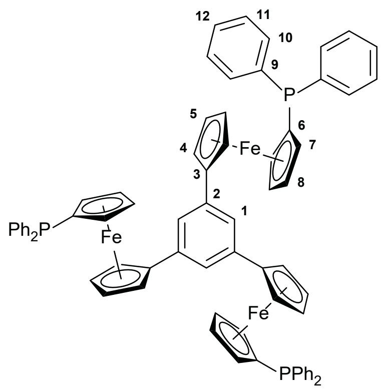

Mp: 95–96 °C (from CH_2Cl_2). R_f: 0.19 (hexanes/ethyl acetate 10:1). Anal. calcd. for $C_{72}H_{57}Fe_3P_3$: C 73.12, H 4.86, found: C 72.52, H 4.30%.[1] IR (KBr, ṽ): 3068 (m), 3050 (m), 3027 (w), 3013 (w), 3000 (w), 2963 (m), 2929 (w), 2907 (w), 2861 (w) 2860 (w, all ν(C–H)), 1952 (w), 1887 (w), 1817 (w, all aromatic overtones), 1594 (m), 1477 (m), 1433 (m, ν(C–P)), 1384 (w), 1306 (w), 1261 (m), 1192 (w), 1182 (w), 1159 (m), 1093 (m), 1068 (w), 1025 (s), 998 (m), 922 (m), 886 (w), 871 (m), 825 (s), 808 (s), 741 (s), 732 (m), 695 (vs), 631 (m), 498 (s), 456 (m), 409 (w) cm^{-1}. ^1H NMR (CD_2Cl_2, δ): 7.43 (3H, s, H1), 7.40–7.34 (12H, m, H10), 7.33–7.27 (18H, m, H11+12), 4.69 (6H, pt, J_{HH} = 1.9 Hz, H4/5), 4.20 (12H, m, H4/5+ H8), 3.92 (6H, pq, J = 1.9 Hz, H7) ppm. $^{13}C\{^1H\}$ NMR (CD_2Cl_2, δ): 139.2 (d, $^1J_{CP}$ = 10.8 Hz, C9), 138.5 (s, C2), 133.4 (d, $^2J_{CP}$ = 19.5 Hz, C10), 128.4 (s, C12), 128.1 (d, $^3J_{CP}$ = 6.8 Hz, C11), 122.0 (s, C1), 86.0 (s, C3), 76.6 (d, $^1J_{CP}$ = 7.3 Hz, C6), 74.5 (d, $^2J_{CP}$ = 14.6 Hz, C7), 72.9 (d, $^3J_{CP}$ = 3.9 Hz, C8), 70.2 (s, C4/5), 67.8 (s, C4/5) ppm. $^{31}P\{^1H\}$ NMR (CD_2Cl_2, δ): −17.5 (s) ppm. HRMS (ESI-TOF, m/z): Calcd. for $C_{72}H_{57}Fe_3P_3$ 1182.1723; found 1182.1736 $[M]^+$.

1.2.6. 2,4,6-Tris(1-diphenylphosphanyl-1'-ferrocenylene)-1,3,5-trifluorobenzene (1b)

Compound **1b** was prepared analogously to **1a**, using 540 mg **4b** (586 µmol, 1.00 eq.). Purification by column chromatography (hexanes/ethyl acetate, gradient 24:1 to 13:1), combining the fractions, removal of all volatiles, and subsequent inert filtration over silica using CH_2Cl_2 yielded **1b** (480 mg, 66%) as an orange foam.

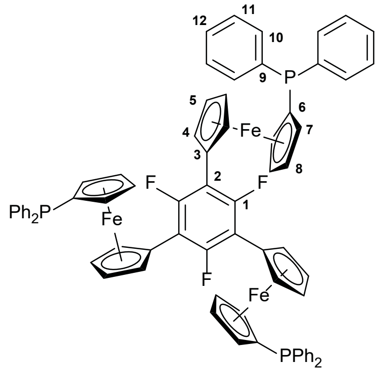

Mp: 95–97 °C (from CH_2Cl_2). R_f: 0.33 (hexanes/ethyl acetate 12:1). Anal. calcd. for $C_{72}H_{54}F_3Fe_3P_3$: C 69.93, H 4.40, found: C 69.53, H 4.04%. IR (KBr, ṽ): 3067 (m), 3049 (m), 3000 (w, all ν(C–H)), 1952 (w), 1893 (w), 1818 (w, all aromatic overtones), 1637 (m), 1605 (m), 1585 (m), 1479 (s), 1431 (s, ν(C–P)), 1420 (m), 1386 (m), 1308 (m), 1263 (w), 1231 (w), 1204 (w), 1159 (m), 1093 (m), 1066 (m), 1024 (vs), 913 (w), 888 (w), 863 (w), 828 (m), 816 (m), 743 (s), 697 (vs), 632 (w), 490 (s), 456 (m) cm^{-1}. ^1H NMR (CD_2Cl_2, δ): 7.39–7.31 (12H, m, H10), 7.30–7.26 (18H, m, H11+H12), 4.73 (6H, m, H4), 4.39 (6H, m, H8), 4.34 (6H, m, H5), 4.10 (6H, m, H7) ppm. $^{13}C\{^1H\}$ NMR (CD_2Cl_2, δ): 155.9 (dt, $^1J_{CF}$ = 252.7 Hz, $^3J_{CF}$ = 10.6 Hz, C1), 139.7 (d, $^1J_{CP}$ = 10.8 Hz, C9), 133.9 (d, $^2J_{CP}$ = 19.6 Hz, C10), 129.0 (s, C12), 128.7 (d, $^3J_{CP}$ = 6.8 Hz, C11), 112.5 (m, C2), 77.5 (d, $^1J_{CP}$ = 7.9 Hz, C6), 75.1 (s, C3), 74.6 (d, $^2J_{CP}$ = 14.5 Hz, C7), 73.6 (d, $^3J_{CP}$ = 3.8 Hz, C8), 71.4 (m, C4), 70.6 (s, C5) ppm. $^{19}F\{^1H\}$ NMR (CD_2Cl_2, δ): −107.8 (s) ppm. $^{31}P\{^1H\}$ NMR (CD_2Cl_2, δ): −17.6 (s) ppm. HRMS (ESI-TOF, m/z): Calcd. for $C_{72}H_{54}F_3Fe_3P_3$ 1236.1440, calcd. for $C_{72}H_{54}F_3Fe_3P_3+H^+$ 1237.1518; found 1236.1482 $[M]^+$, 1237.1515 $[M+H]^+$.

1.2.7. 1,3,5-Tris[(1-diphenylphosphanyl-1'-ferrocenylene)methyl}benzene (1d)

Compound **1d** was prepared analogously to **1a**, using 1.28 g **4d** (1.41 mmol, 1.00 eq.). Purification by column chromatography (hexanes/ethyl acetate, gradient 24:1 to 13:1), combining the fractions, removal of all volatiles, and subsequent inert filtration over silica using CH_2Cl_2 yielded **1d** (740 mg, 43%) as an orange foam.

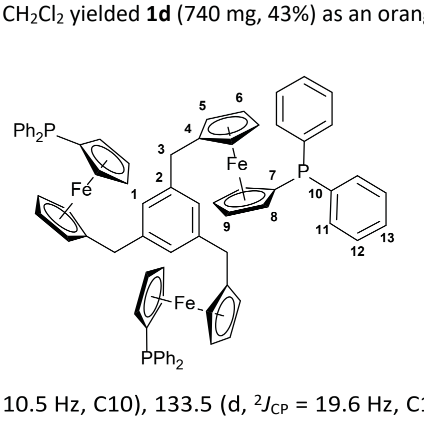

Mp: 95–96 °C (from CH_2Cl_2). R_f: 0.34 (hexanes/ethyl acetate 9:1). Anal. calcd. for $C_{75}H_{63}Fe_3P_3$: C 73.55, H 5.18, found: C 73.08, H 5.14%. IR (KBr, ṽ): 3068 (m), 3049 (m), 3014 (m), 3000 (m), 2913 (m), 2836 (w, all ν(C–H)), 1953 (w), 1888 (w), 1813 (w), 1748 (w), 1662 (w, all aromatic overtones), 1599 (m), 1585 (m), 1569 (w), 1477 (m), 1456 (m), 1433 (s, ν(C–P)), 1383 (m), 1324 (w), 1307 (m), 1226 (w), 1192 (m), 1181 (m), 1159 (s), 1093 (m), 1068 (m), 1025 (s), 998 (m), 969 (w), 924 (m), 887 (w), 826 (s), 811 (s), 741 (vs), 696 (vs), 632 (m), 490 (vs), 455 (s) cm^{-1}. ^1H NMR (CD_2Cl_2, δ): 7.40–7.33 (12H, m, H11), 7.33–7.27 (18H, m, H12+H13), 6.58 (3H, s, H1), 4.29 (6H, pt, J_{HH} = 1.8 Hz, H8/9), 4.03 (6H, pq, J = 1.9 Hz, H8/9), 3.97 (6H, pt, J_{HH} = 1.8 Hz, H5/6), 3.94 (6H, pt, J_{HH} = 1.8 Hz, H5/6), 3.29 (6H, s, H3) ppm. $^{13}C\{^1H\}$ NMR (CD_2Cl_2, δ): 141.5 (s, C2), 139.6 (d, $^1J_{CP}$ = 10.5 Hz, C10), 133.5 (d, $^2J_{CP}$ = 19.6 Hz, C11), 128.4 (s, C13), 128.1 (d, $^3J_{CP}$ = 6.8 Hz, C12), 125.9 (s, C1), 88.9 (s, C4), 75.8 (d, $^1J_{CP}$ =

[1] The results of the CHN analysis for **1a** are just outside the exceptionally allowed range for analytical purity (±0.5%) and are only provided to illustrate the best value obtained to date.

6.8 Hz, C7), 73.6 (d, $^{2}J_{CP}$ = 15.2 Hz, C8), 71.7 (d, $^{3}J_{CP}$ = 4.0 Hz, C9), 69.7 (s, C5/6), 68.8 (s, C5/6), 35.4 (s, C3) ppm. $^{31}P\{^{1}H\}$ NMR (CD_2Cl_2, δ): −17.1 (s) ppm. HRMS (ESI-TOF, m/z): Calcd. for $C_{75}H_{63}Fe_3P_3$ 1224.2193; found 1224.2225 $[M]^{+}$.

1.2.8. 1,3,5-Tris[1-(diphenylphosphanyl borane)-1'-ferrocenylene]benzene ([1a(BH₃)₃])

A stirred solution of 100 mg (84.5 µmol, 1.00 eq.) **1a** in 10 mL CH_2Cl_2 was cooled to 0 °C (ice/water) and 0.15 mL (2.0 mol·L^{-1}, 300 µmol, 3.5 eq.) $BH_3 \cdot SMe_2$ in THF were carefully added. After the gas evolution had ceased, the reaction mixture was kept stirring at room temperature for 45 min after which TLC (CH_2Cl_2/hexanes 1:1) indicated full conversion of **1a**. Further work-up was carried out under ambient conditions. The volatiles were removed under reduced pressure and the residue was filtered over a plug of silica (hexanes/CH_2Cl_2, gradient 1:1 to 1:2), yielding pure **[1a(BH$_3$)$_3$]** (80 mg, 77%) as microcrystalline material after solvent removal. Crystals suitable for XRD were obtained by slow evaporation of a solution of **[1a(BH$_3$)$_3$]** in CH_2Cl_2/hexanes 1:1 v/v.

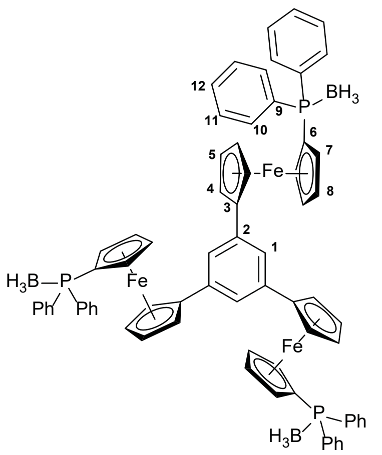

Mp: 220 °C (decomposition; from CH_2Cl_2/hexanes). R_f: 0.27 (hexanes/CH_2Cl_2 1:1). Anal. calcd. for $C_{72}H_{66}B_3Fe_3P_3$: C 70.64, H 5.43, found: C 68.42, 5.37%.[2] IR (KBr, ṽ): 3084 (w), 3052 (w), 2958 (w) 2922 (m), 2852 (m, all ν(C–H)), 2391 (s (br)), 2252 (m, both ν(B–H)), 1961 (w), 1891 (w, both aromatic overtones), 1636 (w), 1593 (m), 1484 (m), 1435 (s, ν(C–P)), 1385 (m), 1362 (w), 1309 (m), 1264 (w), 1173 (s), 1132 (w), 1108 (m), 1063 (s), 1041 (m), 1025 (m), 999 (w), 970 (w), 921 (w), 873 (w), 830 (m), 815 (m), 738 (vs), 693 (s), 671 (m), 637 (m), 609 (m), 526 (w), 498 (s), 478 (s), 450 (m), 409 (w) cm^{-1}. ^{1}H NMR (CD_2Cl_2, δ): 7.64–7.53 (12H, m, H10), 7.51–7.44 (6H, m, H12), 7.44–7.37 (12H, m, H11), 7.31 (3H, s, H1), 4.71 (6H, pt, J_{HH} =1.9 Hz, H4), 4.38 (6H, pq, J = 1.6 Hz, H7), 4.31 (6H, pq, J = 1.9 Hz, H8), 4.21 (6H, pt, J_{HH} = 1.9 Hz, H5), 1.72–0.77 (9H, m (br), BH_3) ppm. $^{1}H\{^{11}B\}$ NMR (CD_2Cl_2, δ): 1.37–1.19 (m (br), BH_3) ppm. $^{11}B\{^{1}H\}$ NMR (CD_2Cl_2, δ): −38.0 (s (br)) ppm. $^{13}C\{^{1}H\}$ NMR (CD_2Cl_2, δ): 137.9 (s, C2), 132.5 (d, $^{2}J_{CP}$ = 9.7 Hz, C10), 131.2 (d, $^{1}J_{CP}$ = 61.8 Hz, C9), 131.0 (s, C12), 128.4 (d, $^{3}J_{CP}$ = 10.1 Hz, C11), 122.1 (s, C1), 86.5 (s, C3), 74.6 (d, $^{2}J_{CP}$ = 7.6 Hz, C7), 74.0 (d, $^{3}J_{CP}$ = 10.0 Hz, C8), 71.2 (s, C4/5), 69.4 (d, $^{1}J_{CP}$ = 68.1 Hz, C6), 68.0 (s, C4/5) ppm. $^{31}P\{^{1}H\}$ NMR (CD_2Cl_2, δ): 15.7 (s (br)) ppm. HRMS (ESI-TOF, m/z): Calcd. for $C_{72}H_{66}B_3Fe_3P_3$+Na: 1247.2632; found 1247.2651 $[M+Na]^{+}$.

1.2.9. 2,4,6-Tris[1-(diphenylphosphanyl borane)-1'-ferrocenylene]-1,3,5-trifluorobenzene ([1b(BH₃)₃])

[1b(BH$_3$)$_3$] was prepared analogously to **[1a(BH$_3$)$_3$]** using 100 mg **1b** (80.9 µmol, 1.00 eq.). Filtration over silica (hexanes/CH_2Cl_2, gradient 1:1 to pure CH_2Cl_2) afforded **[1b(BH$_3$)$_3$]** (100 mg, 97%) as an orange foam.

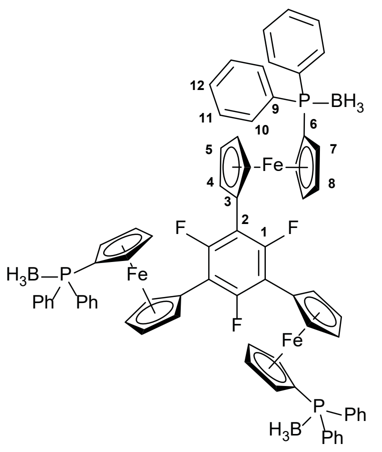

Mp: 90 °C (decomposition; from CH_2Cl_2). R_f: 0.70 (CH_2Cl_2). Anal. calcd. for $C_{72}H_{63}B_3F_3Fe_3P_3$: C 67.66, H 4.97, found: C 67.94, H 4.90%. IR (neat, ṽ): 3076 (w), 3053 (w), 2959 (w), 2927 (w), 2855 (w, all all ν(C–H)), 2374 (m (br)), 2346 (m), 2240 (w, all ν(B–H)), 1604 (w), 1483 (m), 1435 (m, ν(C–P)), 1421 (m), 1388 (m), 1362 (w), 1311 (w), 1233 (w), 1206 (w), 1172 (m), 1133 (w), 1105 (m), 1057 (m), 1024 (s), 999 (w), 913 (w), 893 (w), 871 (w), 830 (m), 735 (s), 693 (s), 637 (m), 609 (m), 527 (m), 495 (s), 474 (vs), 463 (s), 447 (s), 436 (m), 420 (m), 407 (m) cm^{-1}. ^{1}H NMR (CD_2Cl_2, δ): 7.60–7.49 (12H, m, H10), 7.47–7.40 (6H, m, H12), 7.39–7.34 (12H, m, H11), 4.69 (6H, m, H4), 4.53 (6H, m, H7), 4.39 (6H, pq, J = 1.9 Hz, H8), 4.35 (6H, pt, J_{HH} = 1.9 Hz, H5), 1.72–0.74 (9H, BH_3) ppm. $^{1}H\{^{11}B\}$ NMR (CD_2Cl_2, δ): 1.34–1.06 (m (br), BH_3) ppm. $^{11}B\{^{1}H\}$ NMR (CD_2Cl_2, δ): −38.2 (s (br)) ppm. $^{13}C\{^{1}H\}$ NMR (CD_2Cl_2, δ): 153.5 (dt, $^{1}J_{CF}$ = 252.7 Hz, $^{3}J_{CF}$ = 10.4 Hz, C1), 130.5 (d, $^{2}J_{CP}$ = 9.6 Hz, C10), 129.3 (d, $^{1}J_{CP}$ = 55.8 Hz, C9), 129.0 (s, C12), 126.4 (d, $^{3}J_{CP}$ = 10.1 Hz, C11), 109.5 (m, C2), 73.1 (s, C3), 72.4 (d, $^{2}J_{CP}$ = 7.6 Hz, C7), 72.1 (d, $^{3}J_{CP}$ = 9.9 Hz, C8), 69.4 (m, C4), 68.8 (s, C5), 67.5 (d, $^{1}J_{CP}$ = 68.0 Hz, C6) ppm. $^{19}F\{^{1}H\}$ NMR (CD_2Cl_2, δ): −107.5 (s) ppm. $^{31}P\{^{1}H\}$ NMR (CD_2Cl_2, δ): 15.5 (s (br)) ppm. HRMS (ESI-TOF, m/z): Calcd. for $C_{72}H_{63}B_3F_3Fe_3P_3$: 1278.2452; found 1278.2442 $[M]^{+}$.

[2] The results of the CHN analysis for **[1a(BH$_3$)$_3$]** regarding its carbon content are outside the range viewed as establishing analytical purity and are only provided to illustrate the best value obtained to date.

SUPPORTING INFORMATION

1.2.10. μ3-[2,4,6-Tris(1-diphenylphosphanyl-1'-ferrocenylene)-1,3,5-triazine-1κ1*P*,2κ1*P*,3κ1*P*}-tris[chloridogold(I)] ([1c(Au)3])

Under stirring and protected from light, a solution of 59 mg (50 μmol, 1.0 eq.) **1c** in 2 mL CH_2Cl_2 was added to 50 mg (156 μmol, 3.15 eq.) [AuCl(tht)] in 1 mL CH_2Cl_2 and kept stirring at room temperature overnight after which the protection from light was discontinued. The reaction mixture was filtered, its volume was reduced to approx. one third and complex **[1c(Au)3]** was precipitated from the dark red solution using 5 mL diethyl ether, yielding a red powder which was dried *in vacuo* (69 mg, 74%). Crystals suitable for XRD were obtained by layering a solution of **[1c(Au)3]** in $CHCl_3$ with diethyl ether and keeping it at 7 °C for two days.

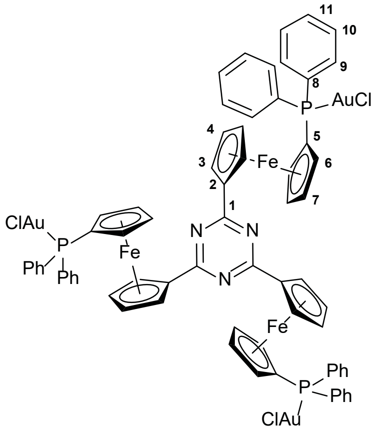

Mp: 240 °C (decomposition; from CH_2Cl_2/diethyl ether). Anal. calcd. for $C_{69}H_{54}Au_3Cl_3Fe_3N_3P_3$: C 44.01, H 2.89, N 2.23, found: C 43.76, H 2.55, N 2.29%. IR (KBr, ṽ): 3086 (w), 3050 (w), 2961 (w), 2922 (w), 2855 (w, all ν(C–H)), 1509 (s), 1482 (m), 1435 (m), 1395 (w), 1380 (m), 1359 (m), 1319 (m), 1310 (m), 1195 (w), 1171 (m), 1102 (m), 1071 (w), 1055 (w), 1028 (m), 998 (w), 928 (w), 833 (m), 745 (m), 691 (s), 627 (m), 555 (m), 528 (m), 503 (s), 478 (s), 455 (m), 433 (m) cm^{-1}. ^1H NMR ($CDCl_3$, δ): 7.61–7.48 (18H, m, H10+H11), 7.47–7.41 (12H, m, H9), 5.23 (6H, pt, J_{HH} = 2.0 Hz, H3), 4.63 (6H, pt, J_{HH} = 2.0 Hz, H4), 4.35 (6H, m, H6/7), 4.34 (6H, m, H6/7) ppm. $^{13}C\{^1H\}$ NMR ($CDCl_3$, δ): 174.9 (s, C1), 133.6 (d, $^3J_{CP}$ = 13.9 Hz, C10), 131.9 (d, $^4J_{CP}$ = 2.2 Hz, C11), 130.4 (d, $^1J_{CP}$ = 63.9 Hz, C8), 129.0 (d, $^2J_{CP}$ = 11.8 Hz, C3), 81.3 (s, C2), 75.4 (d, $^2J_{CP}$ = 9.0 Hz, C6), 74.6 (d, $^3J_{CP}$ = 13.7 Hz, C7), 74.4 (s, C3/4), 71.6 (s, C3/4), 70.6 (d, $^1J_{CP}$ = 72.5 Hz, C5) ppm. $^{31}P\{^1H\}$ NMR ($CDCl_3$, δ): 27.9 (s) ppm. HRMS (ESI-TOF, m/z): Calcd. for $C_{69}H_{54}Au_3Cl_3Fe_3N_3P_3$+Na 1905.9531; found 1905.9540 $[M+Na]^+$.

1.2.11. μ3-[1,3,5-Tris(1-diphenylphosphanyl-1'-ferrocenylene)benzene-1κ1*P*,2κ1*P*,3κ1*P*]-tris[chloridogold(I)] ([1a(Au)3])

[1a(Au)3] was prepared analogously to **[1c(Au)3]** using 100 mg **1a** (84.6 μmol, 1.00 eq.) and obtained as a yellow-orange amorphous solid (130 mg, 82%).

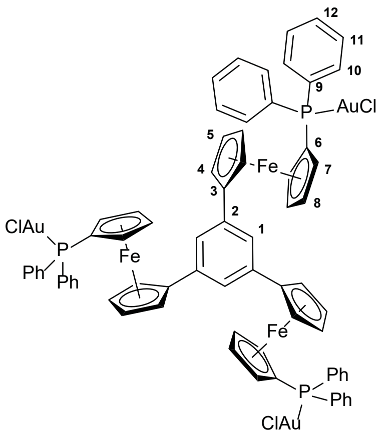

Mp: 185 °C (decomposition; from CH_2Cl_2/diethyl ether). Anal. calcd. for $C_{72}H_{57}Au_3Cl_3Fe_3P_3$: C 46.00, H 3.06, found: C 45.66, H 3.11%. UV/Vis (CH_2Cl_2), λ_{max} (ε): 449 (1670), 290 (43600) nm ($dm^3 \cdot mol^{-1} \cdot cm^{-1}$). IR (ATR, ṽ): 3075 (m), 3054 (m), 2971 (w), 2859 (w, all ν(C–H)), 1595 (m), 1480 (m), 1435 (s, ν(C–P)), 1386 (m), 1362 (w), 1309 (m), 1252 (w), 1196 (m), 1171 (s), 1101 (s), 1071 (m), 1058 (m), 1026 (s), 998 (m), 922 (w), 871 (w), 827 (s), 744 (s), 691 (vs), 625 (m), 555 (s), 527 (s), 492 (vs), 477 (vs), 424 (s) cm^{-1}. ^1H NMR (CD_2Cl_2, δ): 7.62–7.53 (12H, m, H11), 7.51–7.46 (6H, m, H12), 7.46–7.39 (12H, m, H10), 7.22 (3H, s, H1), 4.79 (6H, pt, J_{HH} = 1.9 Hz, H4), 4.47 (6H, pt, J_{HH} = 1.9 Hz, H5), 4.38 (6H, pq, *J* = 1.5 Hz, H7), 4.30 (6H, pdt, *J* = 3.6, 1.7 Hz, H8) ppm. $^{13}C\{^1H\}$ NMR (CD_2Cl_2, δ): 137.8 (s, C2), 133.5 (d, $^3J_{CP}$ = 13.8 Hz, C11), 131.7 (d, $^4J_{CP}$ = 2.6 Hz, C12), 130.7 (d, $^1J_{CP}$ = 63.6 Hz, C9), 128.9 (d, $^2J_{CP}$ = 11.8 Hz, C10), 122.1 (s, C1), 86.6 (s, C3), 75.5 (d, $^2J_{CP}$ = 9.0 Hz, C7), 74.4 (d, $^3J_{CP}$ = 14.0 Hz, C8), 71.9 (s, C4/5), 69.7 (d, $^1J_{CP}$ = 73.4 Hz, C6), 68.3 (s, C4/5) ppm. $^{31}P\{^1H\}$ NMR (CD_2Cl_2, δ): 28.3 (s) ppm. HRMS (ESI-TOF, m/z): Calcd. for $C_{72}H_{57}Au_3Cl_2Fe_3P_3$ 1843.0098; found 1843.0075 $[M-Cl]^+$.

SUPPORTING INFORMATION

1.2.12. μ3-[2,4,6-Tris(1-diphenylphosphanyl-1'-ferrocenylene)-1,3,5-trifluorobenzene-1κ[1]*P*,2κ[1]*P*,3κ[1]*P*]-tris[chloridogold(I)] ([1b(Au)3])

[1b(Au)3] was prepared analogously to **[1c(Au)3]** using 100 mg **1b** (80.9 µmol, 1.00 eq.) and obtained as a yellow amorphous solid (97 mg, 62%).

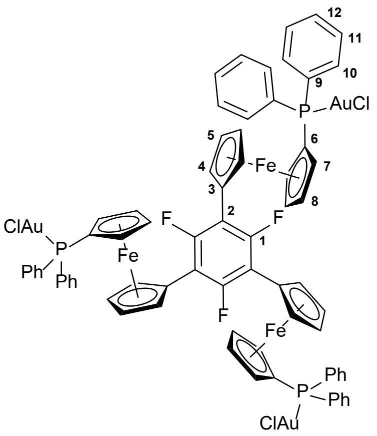

Mp: 180 °C (from CH_2Cl_2/diethyl ether). Anal. calcd. for $C_{72}H_{54}Au_3Cl_3F_3Fe_3P_3$: C 44.72, H 2.81, found: C 44.69, H 2.45%. IR (KBr, ṽ): 3074 (w), 3050 (w), 2969 (w), 2926 (w), 2855 (w, all ν(C–H)), 1606 (w), 1481 (m), 1435 (m, ν(C–P)), 1434 (m), 1421 (m), 1387 (m), 1309 (m), 1232 (w), 1205 (m), 1172 (m), 1101 (m), 1069 (w), 1024 (m), 998 (m), 914 (w), 891 (w), 830 (m), 745 (m), 691 (s), 627 (m), 556 (m), 528 (m), 475 (s), 458 (m), 438 (m), 419 (m) cm^{-1}. ^{1}H NMR (CD_2Cl_2, δ): 7.61–7.53 (12 H, m, H11), 7.51–7.44 (6 H, m, H12), 7.44–7.33 (12 H, m, H10), 4.79 (6 H, m, H4), 4.58 (6 H, m, H7/8), 4.51 (6 H, pt, J_{HH} = 1.8 Hz, H5), 4.36 (6 H, m, H7/8) ppm. $^{13}C\{^{1}H\}$ NMR (CD_2Cl_2, δ): 156.8 (m, C1), 133.4 (d, $^{3}J_{CP}$ = 13.8 Hz, C11), 131.6 (d, $^{4}J_{CP}$ = 2.7 Hz, C12), 130.7 (d, $^{1}J_{CP}$ = 63.7 Hz, C9), 128.8 (d, $^{2}J_{CP}$ = 11.8 Hz, C10), 111.2 (m, C2), 75.4 (s, C3), 75.0 (d, $^{2}J_{CP}$ = 9.0 Hz, C7), 74.6 (d, $^{3}J_{CP}$ = 13.9 Hz, C8), 71.6 (m, C4), 71.2 (s, C5), 69.7 (d, $^{1}J_{CP}$ = 73.3 Hz, C6) ppm. $^{19}F\{^{1}H\}$ NMR (CD_2Cl_2, δ): −107.0 (s) ppm. $^{31}P\{^{1}H\}$ NMR (CD_2Cl_2, δ): 28.1 (s) ppm. HRMS (ESI-TOF, m/z): Calcd. for $C_{72}H_{54}Au_3Cl_3F_3Fe_3P_3$ 1933.9495; found 1933.9466 $[M]^{+}$.

1.2.13. 1,1'-Diacetylferrocinium teflonate (8)

A colourless solution of 1.07 g (1.00 mmol, 1.00 eq.) silver teflonate in 10 mL CH_2Cl_2 was transferred to an orange solution of 270 mg (1.00 mmol, 1.00 eq.) 1,1'-diacetylferrocene in 10 mL CH_2Cl_2 leading to an colour change to dark teal and precipitation of a grey metallic solid (elemental silver). The solution was stirred for 18 h, filtered over dry Celite®, and the filter residue was extracted with 10 mL CH_2Cl_2. The crude product was precipitated by adding 150 mL *n*-pentane. After decanting off the mother liqueur, the residue was re-dissolved in 10 mL CH_2Cl_2 and layered with 50 mL *n*-hexane. After a few days at 4 °C, crystalline **8** was obtained as dark blue needles. The crystals were washed twice with 10 mL *n*-pentane and dried *in vacuo* (950 mg, 77%).

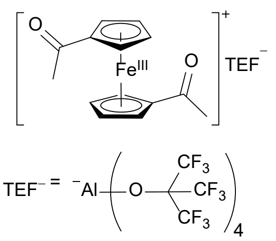

Mp: 90–91 °C (decomp.; from CH_2Cl_2/hexanes). Anal. calcd. for $C_{30}H_{14}AlF_{36}O_6$: C 29.12, H 1.14, found: C 29.38, H 1.23 %. IR (KBr, ṽ): 3125 (w), 3090 (w), 3015 (vw), 2935 (vw), 1702 (m, ν(C=O)), 1357 (m), 1302 (s), 1279 (s), 1243 (s), 1221 (s), 1169 (m), 974 (s), 867 (w) 833 (w), 728 (s), 661 (vw), 620 (w), 564 (w), 538 (w), 446 (w) cm^{-1}. ^{1}H NMR (CD_2Cl_2, δ)[3]: 32.5 (br s, $\omega_{1/2}$ = 1400 Hz), 4.0 (br s, $\omega_{1/2}$ = 361 Hz), −10.1 (br s, $\omega_{1/2}$ = 321 Hz) ppm. $^{13}C\{^{1}H\}$ NMR (CD_2Cl_2): no signals found. $^{19}F\{^{1}H\}$ NMR (CD_2Cl_2, δ): −76.2 (s, CF_3) ppm. $^{27}Al\{^{1}H\}$ NMR (CD_2Cl_2, δ): 34.7 (s) ppm. MS (ESI-TOF, m/z): Cation: Calcd. for $C_{14}H_{14}FeO_2^{+}$ 271.04; found 271.1 $[M_{cat}]^{+}$; Anion: Calcd. for $AlO_4C_{16}F_{36}^{-}$ 966.90, found 967.1 $[M_{an}]^{-}$.

1.2.14. Monooxidised complex [1a(Au)3](TEF)

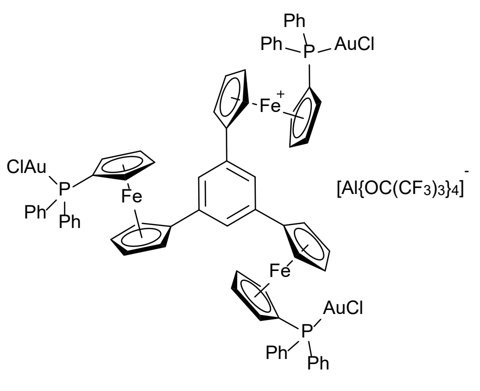

To a yellow solution of 30.0 mg (16.0 µmol, 1.00 eq.) **[1a(Au)3]** in 3 mL CH_2Cl_2 cooled to 0 °C (ice/water), a similarly cooled, dark-brown solution of 21.0 mg (16.8 µmol, 1.05 eq.) **8** in 2 mL CH_2Cl_2 was added in small portions *via* cannula under stirring. The colour of the oxidant solution vanished immediately upon mixing with the solution of **[1a(Au)3]**, which in turn slightly darkened in colour. Stirring at 0 °C was continued for 30 min after which the volatiles were removed *in vacuo* at 0 °C. The brown residue was taken up in 2 mL diethyl ether, to which 4 mL pentanes were added under vigorous stirring. The orange supernatant, containing 1,1'-diacetylferrocene as confirmed by ^{1}H NMR spectroscopy, was filtered off the resulting light-green precipitate, and this procedure was repeated twice, until the washing solution was colourless. The light-green solid was dried *in vacuo* at room temperature (45 mg, quant.).

[3] Integrals cannot reliably be determined and are thus excluded for all paramagnetic species.

Mp: 145 °C (decomp.; from Et_2O/pentanes). Anal. calcd. for $C_{88}H_{57}AlAu_3Cl_3F_{36}Fe_3O_4P_3$: C 37.13, H 2.02, found: C 37.60, H 1.72%. UV/Vis (CH_2Cl_2), λ_{max} (ε): 860 (350), 602 (320), 439 (3490), 354sh (8000) 288 (39200) nm ($dm^3{\cdot}mol^{-1}{\cdot}cm^{-1}$). IR (KBr, $\tilde{\nu}$): 3086 (w), 3058 (w, both ν(C–H)), 1665 (vw), 1596 (w), 1483 (w), 1438 (m, ν(C–P)), 1388 (w), 1351 (m), 1298 (m), 1275 (s), 1239 (s), 1215 (vs), 1169 (s), 1102 (m), 1028 (m), 972 (vs), 923 (w), 829 (m), 747 (s), 727 (s), 713 (m), 691 (s), 627 (m), 557 (m), 536 (m), 526 (m), 478 (s), 445 (s) cm^{-1}. ^1H NMR (CD_2Cl_2, δ): 13.8 (br, $\omega_{1/2}$ = 300 Hz), 12.9 (br, $\omega_{1/2}$ = 430 Hz), 11.0 (br, $\omega_{1/2}$ = 430 Hz), 8.5 (br, $\omega_{1/2}$ = 480 Hz), 7.9 (br s, $\omega_{1/2}$ = 32 Hz, $P(C_6H_5)_2$), 7.6–7.3 (m, $P(C_6H_5)_2$) −7.8 (br, $\omega_{1/2}$ = 490 Hz) ppm. ^{19}F NMR (CD_2Cl_2, δ): −75.8 (s, $\omega_{1/2}$ = 4 Hz, CF_3) ppm. $^{31}P\{^1H\}$ NMR (CD_2Cl_2, δ): 12.0 (br s, $\omega_{1/2}$ = 130 Hz) ppm. HRMS (ESI-TOF, m/z): Calcd. for $C_{72}H_{57}Au_3Cl_3Fe_3P_3^+$ 1879.9778, found 1879.9822 [Cation]$^+$. μ_{eff} = 2.03 μ_B ($\mu_{S.O.}$ = 1.73 μ_B for S = ½).

1.2.15. Dioxidised complex [1a(Au)$_3$](TEF)$_2$

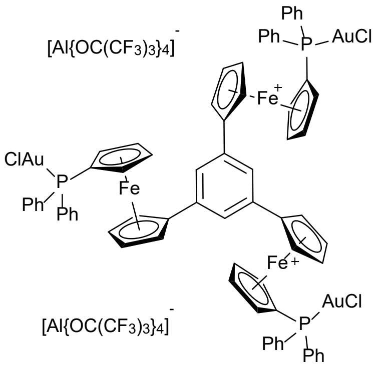

[1a(Au)$_3$](TEF)$_2$ was prepared analogously to **[1a(Au)$_3$](TEF)** using 30.0 mg **[1a(Au)$_3$]** (16.0 µmol, 1.00 eq.) in 2 mL CH_2Cl_2 and 41.5 mg **8** (33.5 µmol, 2.10 eq.) in 4.5 mL CH_2Cl_2. The dark-green solid was dried *in vacuo* at room temperature (60 mg, quant.).

Mp: 135 °C (decomp.; from Et_2O/pentanes). Anal. calcd. for $C_{104}H_{57}Al_2Au_3Cl_3F_{72}Fe_3O_8P_3$: C 32.75, H 1.51, found: C 33.11, H 1.38%. UV/Vis (CH_2Cl_2), λ_{max} (ε): 825 (700), 622 (700), 430 (4580), 356sh (11700) 287sh (30400) nm ($dm^3{\cdot}mol^{-1}{\cdot}cm^{-1}$). IR (KBr, $\tilde{\nu}$): 3088 (w, ν(C–H)), 1670 (vw), 1597 (w), 1484 (w), 1439 (w), 1388 (vw), 1351 (m), 1297 (m), 1274 (s), 1239 (s), 1212 (vs), 1168 (s), 1103 (m), 1029 (m), 970 (vs), 859 (m), 829 (m), 748 (m), 726 (s), 715 (m), 691 (s), 627 (m), 558 (m), 536 (m), 529 (m), 485 (s), 442 (s) cm^{-1}. ^1H NMR (CD_2Cl_2, δ): 21.2 (br, $\omega_{1/2}$ = 1600 Hz), 19.0 (br, $\omega_{1/2}$ = 1360 Hz), 12.7 (br, $\omega_{1/2}$ = 1430 Hz), 10.3 (br, $\omega_{1/2}$ = 63 Hz), 8.8 (br s, $\omega_{1/2}$ = 70 Hz, $P(C_6H_5)_2$), 7.7 (br s, $\omega_{1/2}$ = 19 Hz, $P(C_6H_5)_2$), 7.5 (br s, $\omega_{1/2}$ = 18 Hz, $P(C_6H_5)_2$), 7.7–7.2 (m, CH_{arom}), 7.1 (br s, $\omega_{1/2}$ = 23 Hz, CH_{arom}), 1.8 (br, $\omega_{1/2}$ = 351 Hz), −12.4 (br s, $\omega_{1/2}$ = 90 Hz), −17.5 (br, $\omega_{1/2}$ = 824 Hz), −19.6 (br, $\omega_{1/2}$ = 1480 Hz) ppm. ^{19}F NMR (CD_2Cl_2, δ): −75.8 (s, $\omega_{1/2}$ = 4 Hz, CF_3) ppm. $^{31}P\{^1H\}$ NMR (CD_2Cl_2, δ): 5.8 (br s, $\omega_{1/2}$ = 626 Hz) ppm. HRMS (ESI-TOF, m/z): Calcd. for $C_{72}H_{57}Au_3Cl_3Fe_3P_3^{2+}$ 939.9886, found 939.9855 [Cation]$^{2+}$. μ_{eff} = 2.98 μ_B ($\mu_{S.O.}$ = 2.83 μ_B for S = 1).

1.2.16. Trioxidised complex [1a(Au)$_3$](TEF)$_3$

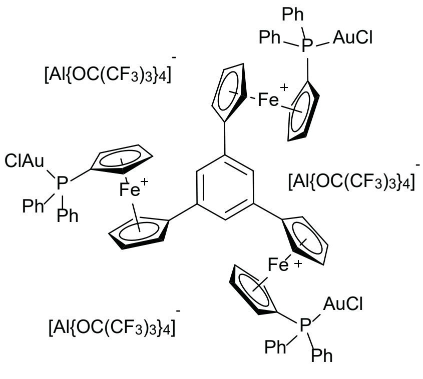

[1a(Au)$_3$](TEF)$_3$ was prepared analogously to **[1a(Au)$_3$](TEF)** using 30.0 mg **[1a(Au)$_3$]** (16.0 µmol, 1.00 eq.) in 2 mL CH_2Cl_2 and 62.0 mg **8** (50.3 µmol, 3.15 eq.) in 7 mL CH_2Cl_2. Repeated extraction cycles with diethyl ether/pentane ensured full conversion to the triply oxidised complex as monitored by IR spectroscopy (disappearance of the ν(C=O) vibration of **8**). The black solid was dried *in vacuo* at room temperature (76 mg, quant.). Crystals suitable for XRD analysis were obtained by letting pentanes vapour-diffuse into a concentrated solution of **[1a(Au)$_3$](TEF)$_3$** in 1,2-dichloroethane at 7 °C for several days.

Mp: 150 °C (decomp.; from Et_2O/pentanes). Anal. calcd. for $C_{120}H_{57}Al_3Au_3Cl_3F_{108}Fe_3O_{12}P_3$: C 30.15, H 1.20, found: C 30.41, H 1.10%. UV/Vis (CH_2Cl_2), λ_{max} (ε): 790 (1350), 639 (1370), 424sh (6000), 339sh (17800) 289sh (33200), 246 (49500) nm ($dm^3{\cdot}mol^{-1}{\cdot}cm^{-1}$). IR (KBr, $\tilde{\nu}$): 3130 (sh, w), 3080 (w, both ν(C–H)), 1620 (br, vw), 1484 (w), 1440 (w), 1388 (vw), 1351 (m), 1296 (s), 1273 (s), 1239 (s), 1207 (vs), 1168 (s), 1103 (m), 1040 (w), 968 (vs), 859 (m), 831 (m), 747 (m), 727 (vs), 690 (m), 628 (w), 559 (m), 536 (m), 519 (m), 479 (w), 441 (m) cm^{-1}. ^1H NMR (CD_2Cl_2, δ)[4]: 9.3 (br s, $\omega_{1/2}$ = 177 Hz, $P(C_6H_5)_2$), 7.9 (br s, $\omega_{1/2}$ = 35 Hz, $P(C_6H_5)_2$), 7.4 (br s, $\omega_{1/2}$ = 48 Hz, $P(C_6H_5)_2$) ppm. ^{19}F NMR (CD_2Cl_2, δ): −75.3 (s, $\omega_{1/2}$ = 17 Hz, CF_3) ppm. $^{31}P\{^1H\}$ NMR (CD_2Cl_2, δ): 5.5 (br s, $\omega_{1/2}$ = 1080 Hz) ppm. HRMS (ESI-TOF, m/z): Calcd. for $C_{72}H_{57}Au_3Cl_3Fe_3P_3^{3+}$ 626.6589, found 626.6608 [Cation]$^{3+}$; cald. for $C_{88}H_{57}AlAu_3Cl_3F_{36}Fe_3O_4P_3^{2+}$ 1423.4409, found 1423.4452 [Cation+TEF]$^{2+}$. μ_{eff} = 3.52 μ_B ($\mu_{S.O.}$ = 3.87 μ_B for S = $^3/_2$).

---

[4] No other resonances than the three phenyl-ring proton resonances have been found between 50 and −45 ppm; they might be too broad to be identified.

1.3. Catalytic tests

Catalytic experiments were run on the NMR scale under argon in inert-gas NMR tubes, using 0.5 mL of a stock solution of *N*-prop-2-ynylbenzamide (**5**) (72.0 mmol·L^{-1}) and 1,3,5-trimethoxybenzene (7.2 mmol·L^{-1}) in dry CD_2Cl_2 (containing TMS as an internal chemical shift standard) and 0.1 mL of a stock solution of the respective (pre-)catalyst **[1a–c(Au)$_3$](TEF)$_n$** (**n = 0**: 3 mol% Au: 3.6 mmol·L^{-1}; **n = 0–3**: 1 mol% Au: 1.2 mmol·L^{-1}) or **7** (3 mol% Au: 10.8 mmol·L^{-1}; 1 mol% Au: 3.6 mmol·L^{-1}) in CD_2Cl_2, leading to an effective substrate concentration of [**5**]$_0$ = 60 mmol·L^{-1} and [1,3,5-trimethoxybenzene] = 6.0 mmol·L^{-1}. The substrate stock solution was stored at 7 °C and protected from light. Catalyst stock solutions were freshly prepared.

For activation of the pre-catalysts by halide abstraction, 67 µL of a dried (stirred over 3 Å molecular sieves overnight, then filtered) stock solution of $NaBAr^F_4$ in acetonitrile (5 mg·mL^{-1}) were added to an inert-gas NMR tube. The solvent was removed *in vacuo* and the colourless residue was carefully dried using a heat gun at low power. Next, 0.1 mL of the respective pre-catalyst stock solution were added to the NMR tube, which was then put in an ultra-sonicating bath for 5 min. The catalytic reaction was started (t = 0) upon addition of 0.5 mL of the substrate stock solution.

For redox-switching experiments, stock solutions of oxidant **8** (3 mol% Au: 18.0 mmol·L^{-1}; 1 mol% Au: 6.0 mmol·L^{-1}) and reductant **9** (3 mol% Au: 19.8 mmol·L^{-1}; 1 mol% Au: 6.6 mmol·L^{-1}) were freshly prepared for each set of runs and added to the inert-gas NMR tubes at the chosen points in time as 20 µL (1.0 eq. **8**, 1.1 eq. **9**), 40 µL (2.0 eq. **8**, 2.2 eq. **9**), or 60 µL (3.0 eq. **8**, 3.3 eq. **9**) aliquots for the first oxidation and reduction, respectively, *via* microliter syringes. For re-oxidation, 24 µL (1.2 eq. **8**) 48 µL (2.4 eq. **8**), or 72 µL (3.6 eq. **8**) were added. After each addition of **8** or **9**, the NMR tube was closed again and shaken vigorously.

All reactions were followed by time-resolved ^1H NMR spectroscopy at 25 °C, using 12 scans and d = 10 s. For evaluation of the conversion/yield, spectra were stacked and jointly subjected to auto phase correction and baseline correction (polynomial fit) by MestReNova (version 12.0.0-20080). Spectra, which had not been properly shimmed, were removed from further analyses. Using the “Concentration Graph” function of MestReNova’s built-in data analysis suite, signals of interest were integrated against the signal from the internal standard’s methoxy protons (9H, 3.71–3.79 ppm). As best suited for determining the yield, the *ortho*-phenyl protons (H_O) of **6** (2H, 7.93–7.99) were chosen (*vide infra*, section 5). Cross-checking against other ^1H NMR signals of both **5** and **6** gave similar yields, conversions, and turn-over frequencies (TOF) than those reported.

SUPPORTING INFORMATION

2. Single Crystal X-Ray Diffraction Analyses

2.1. Crystallographic data

Table S1 Crystallographic data of tris(1-bromo-1'-ferrocenylene)arenes **4a**,**b**,**d**,**e** and side products **SP1–3**.

	$C_6H_3(fcBr)_3$ **4a**	$C_6F_3(fcBr)_3$ **4b**	$C_6H_3(CH_2fcBr)_3$ **4d**	$C_6Me_3(fcBr)_3$ **4e**	**Brfc-fc-fcBr SP1**	$Fc\{C_6Me_3(fcBr)_2\}_2$ **SP2**	$C_6BrF_3(fcBr)_2$ **SP3**
Empirical formula[a]	$C_{36}H_{27}Br_3Fe_3$	$C_{36}H_{24}Br_3F_3Fe_3$	$C_{39}H_{33}Br_3Fe_3$	$C_{39}H_{33}Br_3Fe_3$	$C_{30}H_{24}Br_2Fe_3$	$C_{68}H_{58}Br_4Fe_5 \cdot 1.1\ CH_2Cl_2$	$C_{26}H_{16}Br_3F_3Fe_2$
Formula weight [g·mol⁻¹]	866.86	920.83	908.93	908.93	711.84	1569.27	736.82
T [K]	130(2)	130(2)	130(2)	130(2)	130(2)	130(2)	130(2)
Crystal system / Space group	Monoclinic / $P2_1/c$	Monoclinic / $P2_1/c$	Monoclinic / $P2_1/c$	Monoclinic / $P2_1/c$	Monoclinic / $P2_1/c$	Triclinic / $P\bar{1}$	Monoclinic / $P2_1/c$
a,	12.562(5),	14.0078(4),	7.7184(2),	15.6190(5),	15.7205(3),	11.5217(3),	10.8725(2),
b,	10.663(5),	19.3202(5),	32.9287(7),	14.0809(5),	7.4429(2),	11.6762(3),	7.3697(1),
c [Å]	21.857(5)	12.2848(4)	12.6304(3)	14.7566(6)	20.7347(5)	23.9327(7)	57.208(1)
α,	90,	90,	90,	90,	90,	76.369(2),	90,
β,	93.762(5),	115.926(4),	93.703(2),	98.420(3),	100.647(2),	88.688(2),	94.432(2),
γ [°]	90	90	90	90	90	69.101(3)	90
V [Å³]	2921.4(2)	2990.1(2)	3203.4(1)	3210.4(2)	2384.3(1)	2916.5(2)	4570.2(2)
Z	4	4	4	4	4	2	8
ρ_{calc} [g·cm⁻³]	1.971	2.046	1.885	1.881	1.983	1.787	2.142
Θ_{max} [°]	25.35	30.321	29.953	28.281	30.415	26.373	27.122
F(000)	1704	1800	1800	1800	1408	1566	2848
Reflns collected	16249	15838	25894	16679	12280	48496	39366
Independent reflns	5339	7453	8609	7967	6431	11917	9745
R_1 / wR_2 {$I > 2\sigma(I)$}	0.0550 / 0.1371	0.0327 / 0.0612	0.0460 / 0.0666	0.0405 / 0.0912	0.0410 / 0.0775	0.0404 / 0.0776	0.0640 / 0.1130
R_1 / wR_2 (all data)	0.0737 / 0.1544	0.0508 / 0.0683	0.0834 / 0.0762	0.0610 / 0.1021	0.0649 / 0.0869	0.0791 / 0.0824	0.0735 / 0.1161
Largest diff. peak / hole [e·Å⁻³]	1.529 / −2.446	0.524 / −0.754	0.598 / −0.736	1.556 / −1.365	0.783 / −0.993	1.741 / −1.959	1.296 / −1.580

[a] Given as Compound·Solvent(s) for clarity.

SUPPORTING INFORMATION

Table S2 Crystallographic data of tris-phosphanes **1a**,**d**, borane adduct **[1a(BH$_3$)$_3$]**, gold complex **[1c(Au)$_3$]**, and trioxidised gold complex **{[1a(Au)$_3$]}$_2$(TEF)$_6$**.

	**$C_6H_3(fcPPh_2)_3$ 1a**	**$C_6H_3(CH_2fcPPh_2)_3$ 1d**	**$C_6H_3(fcPPh_2BH_3)_3$ [1a(BH$_3$)$_3$]**	**[C_3N_3{fcPPh$_2$(AuCl)}$_3$] [1c(Au)$_3$]**	**[C_6H_3{fcPPh$_2$(AuCl)}$_3$)]$_2$ [Al{OC(CF$_3$)$_3$}$_4$]$_6$ {[1a(Au)$_3$]}$_2$(TEF)$_6$**
Empirical formula[a]	$C_{72}H_{57}Fe_3P_3$	$C_{75}H_{63}Fe_3P_3 \cdot 0.25\ C_5H_{12} \cdot 0.5\ CH_2Cl_2$	$C_{72}H_{66}B_3Fe_3P_3$	$C_{69}H_{54}Au_3Cl_3N_3P_3 \cdot CHCl_3$	$(C_{72}H_{57}Au_3Cl_3Fe_3P_3)_2 \cdot (C_{16}AlF_{36}O_4)_6 \cdot 3.5\ C_2H_4Cl_2$
Formula weight [g·mol^{-1}]	1182.63	1284.13	1224.13	2002.23	9908.94
T [K]	130(2)	130(2)	130(2)	130(2)	130(2)
Crystal system / Space group	Triclinic / $P\bar{1}$	Triclinic / $P\bar{1}$	Trigonal / $R\bar{3}$	Triclinic / $P1$ Flack parameter: −0.008(7)	Triclinic / $P\bar{1}$
a,	12.1916(6),	10.2357(5),	17.2440(3),	9.8526(4),	19.4400(3),
b,	13.1108(6),	16.1124(6),	17.2440(3),	10.9313(4),	25.6696(5),
c [Å]	18.3274(7)	19.2961(7)	33.591(1)	18.774(1)	35.2564(7)
α,	83.468(4),	102.741(3),	90,	92.519(4),	103.677(2),
β,	78.135(4),	96.159(3),	90,	103.594(5),	96.218(2),
γ [°]	80.466(4)	93.096(3)	120	95.610(3)	108.488(2)
V [Å^3]	2817.6(2)	3076.2(2)	8650.2(4)	1951.3(2)	15888.5(6)
Z	2	2	6	1	2
ρ_{calc} [g·cm^{-3}]	1.388	1.386	1.410	1.704	2.071
Θ_{max} [°]	26.371	29.127	26.368	30.702	27.093
F(000)	1214	1334	3816	958	9530
Reflns collected	16017	27216	24383	26496	108958
Independent reflns	10986	13989	3638	19012	61811
R_1 / wR_2 {$I > 2\sigma(I)$}	0.0555 / 0.0982	0.0406 / 0.0829	0.0432 / 0.0890	0.0675 / 0.1551	0.0912 / 0.2241
R_1 / wR_2 (all data)	0.1070 / 0.1152	0.0624 / 0.0911	0.0518 / 0.0931	0.0843 / 0.1667	0.1705 / 0.2770
Largest diff. peak / hole [e·Å^{-3}]	0.875 / −0.616	0.687 / −0.441	0.449 / −0.243	5.197 / −2.386	2.555 / −1.826

[a] Given as Compound·Solvent(s) / Cation·Anion(s)·Solvent(s) for clarity.

2.2. Structural parameters

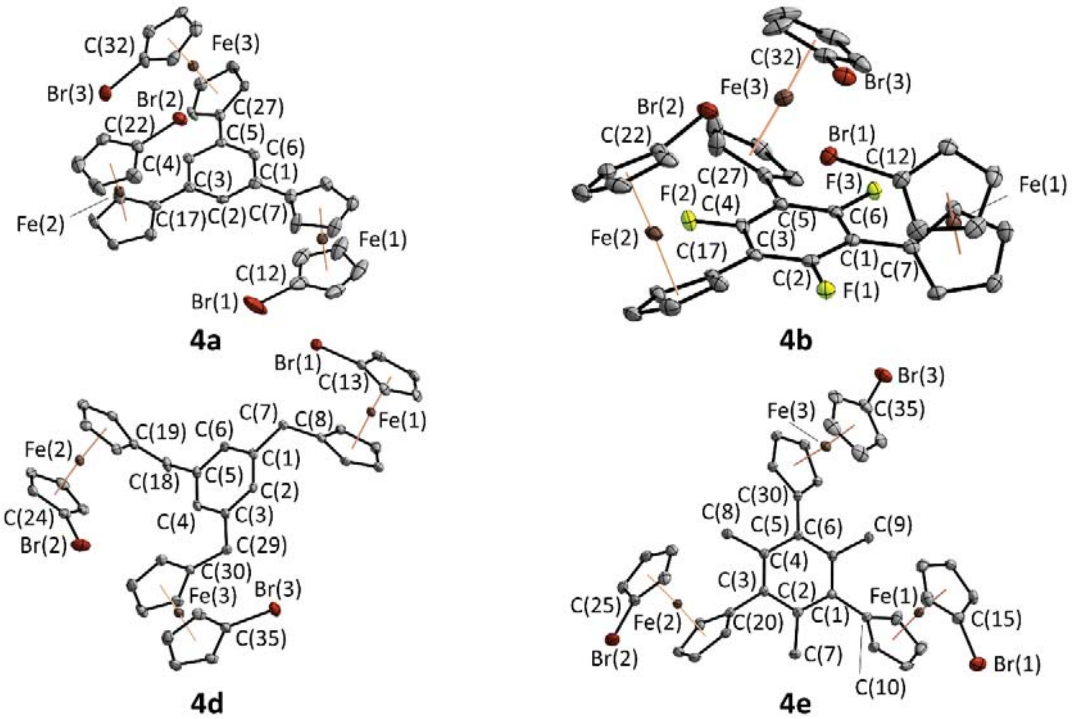

Fig. S1 Molecular structures of tris(1-bromo-1'-ferrocenylene)arenes **4a**,**b**,**d**,**e** including part of their atom-numbering scheme. Thermal ellipsoids are set at the 50% probability level and hydrogen atoms have been omitted for clarity.

SUPPORTING INFORMATION

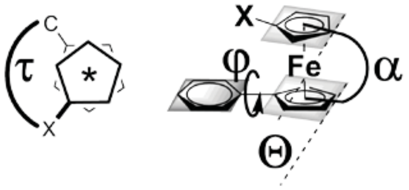

Fig. S2 Schematic representation of key structural parameters for the compounds under investigation (τ: torsion about the Cp^X(centroid)···Fe···Cp^C(centroid) axes; φ: twist angle between the central arene and the directly bound cyclopentadienyl rings; Θ: angle between the mean planes through the substituted cyclopentadienyl rings; α: angle between Ct^C–Fe–Ct^X). Parameters chosen and named following reference [33].

Table S3 Selected bond lengths [Å] and angles [°] of tris(1-bromo-1'-ferrocenylene)arenes **4a**,**b**,**d**,**e**; numbering scheme according to Fig. S1, ferrocenylene geometric parameters according to Fig. S2.

	4a	4b	4d	4e		4a	4b	4d	4e
**C(12)–Br(1) /**	1.89(1)	1.887(4)	1.888(4)	1.901(4)	**α(fc(1)) /**	177.57	179.68	177.88	175.44
**C(22)–Br(2) /**	1.877(6)	1.882(3)	1.874(4)	1.880(4)	**α(fc(2)) /**	176.80	177.40	176.90	175.46
**C(32)–Br(3)**[a]	1.891(6)	1.881(4)	1.894(3)	1.884(4)	**α(fc(3)) /**	179.16	178.04	177.82	175.30
**C(1)–C(7) /**	1.474(9)	1.474(5)	1.502(5)	1.498(5)	**Θ(fc(1)) /**	2.83	0.72	2.42	8.18
**C(3)–C(17) /**	1.482(9)	1.481(4)	1.515(5)	1.499(5)	**Θ(fc(2)) /**	3.95	2.99	4.22	7.26
**C(5)–C(27)**[b]	1.487(9)	1.463(5)	1.510(5)	1.496(6)	**Θ(fc(3))**	0.57	2.71	2.45	6.38
**Ct^C(1)–Fe(1) /**	1.637	1.656	1.644	1.664	**τ(fc(1)) /**	60.19	9.11	1.56	146.10
**Ct^C(2)–Fe(2) /**	1.641	1.651	1.650	1.663	**τ(fc(2)) /**	0.59	0.39	74.92	155.72
**Ct^C(3)–Fe(3)**[c]	1.651	1.654	1.653	1.660	**τ(fc(3))**	64.84	7.20	4.27	143.17
**Ct^{Br}(1)–Fe(1) /**	1.632	1.653	1.638	1.649	**φ(fc(1)) /**	3.40	32.53		40.39
**Ct^{Br}(2)–Fe(2) /**	1.637	1.650	1.642	1.653	**φ(fc(2)) /**	4.93	28.64	-	60.65
**Ct^{Br}(3)–Fe(3)**[d]	1.643	1.654	1.641	1.650	**φ(fc(3))**	30.63	34.13		41.93
**C(2)–X(1) /**		1.349(4)	1.512(5)	1.512(6)					
**C(4)–X(2) /**	-	1.353(3)	1.501(4)	1.519(5)					
**C(6)–X(3)**[e]		1.352(4)	1.515(4)	1.501(5)					

[a] **4d**: C(13)–Br(1), C(24)–Br(2), C(35)–Br(3); **4e:** C(15)–Br(1), C(25)–Br(2), C(35)–Br(3); [b] **4d**: C(7)–C(8), C(18)–C(19), C(29)–C(30); **4e:** C(1)–C(10), C(3)–C(20), C(5)–C(30); [c] Ct^C denotes the calculated centre of gravity of the carbon-substituted cyclopentadienyl ring; [d] Ct^{Br} denotes the calculated centre of gravity of the bromine-substituted cyclopentadienyl ring; [e] **4b**: X = F; **4d**: C(1)–C(7); **4e**: X = C(7), C(8), C(9).

The four crystallographically characterised tris(1-bromo-1'-ferrocenylene)arenes **4a**,**b**,**d**,**e** (Fig. S1) exhibit different conformations, even if trimethylbenzene-based **4d** is not taken into consideration. These differences are most strongly represented by the greatly varying torsion angles τ and φ (Table S3), pointing to little similarity. Just as for **4a**, the closely related compounds 1,3,5-tris(ferrocenyl)benzene[34] and 2,4,6-tris(ferrocenyl)pyridine[35] – their bond lengths and bond angles matching those for **4a**,**b**,**d**,**e** well – crystallise with *syn*,*anti,anti* conformations, two ferrocenyl groups on one side of the arene core and the third on the other.

In contrast, 2,4,6-tris(ferrocenyl)-1,3,5,2,4,6-triselenatriborinane[36] (the B_3Se_3 core also being aromatic) and RuCp{η^6-1,3,5-tris(ferrocenyl)benzene}[37] crystallise in the all-*syn* conformation, resembling the situation in **4b** and **4e** but for different reasons. The conformation of **4b** is most likely connected to weak interactions between the Br atoms and the C–F bonds (their centroids denoted as Ct(CF)), as close contacts of the arene core (d(Ct(CF)···Br) = 3.272–3.315 Å, ∡(Br···Ct(CF)–C_{arene}) = 86–93°), concomitant with Br···C_{arene} distances (3.296–3.416 Å) below the sum of the van-der-Waals radii (3.55 Å),[38] are present. They might be explained in terms of interactions between the lone pairs of electrons of the bromine atoms and the antibonding σ*(C–F) orbitals.[39] Weak interactions involving halogens have recently attained considerable interest for their importance in crystal engineering, pharmaceutical chemistry and materials science.[40] However, this particular interaction, although detected in a search of the Cambridge Structural Database (CSD),[41] for example, in halogenated corroles and porphyrins,[42–47] seems to not yet have been recognised as such.

Regarding the C–Br bond lengths, a slight elongation for sterically encumbered **4e** with respect to the other compounds is apparent, in line with slightly longer C_{arene}–C_{Cp} bonds. While no significant differences among the centroid–Fe distances are found, the steric strain put on **4e** through the three methyl groups becomes noticeable in the strongest deviations of α and Θ from ideal co-planarity of the cyclopentadienyl substituents (180° and 0°, respectively). Two short Fe···H distances of 2.86(5) Å (Fe(3)···$H9_a$) and 2.85(5) Å (Fe(1)···$H9_b$) and one slightly longer one of 3.05(4) Å (Fe(2)···$H8_c$) are all well below the sum of the van der Waals radii (3.61 Å),[38] yet very much longer than the experimentally determined bond lengths of a ferrocenyl hydride (1.34–1.62 Å).[48] If steric constraints or energy gain from attractive interactions are at the heart of these contacts cannot be decided here.

SUPPORTING INFORMATION

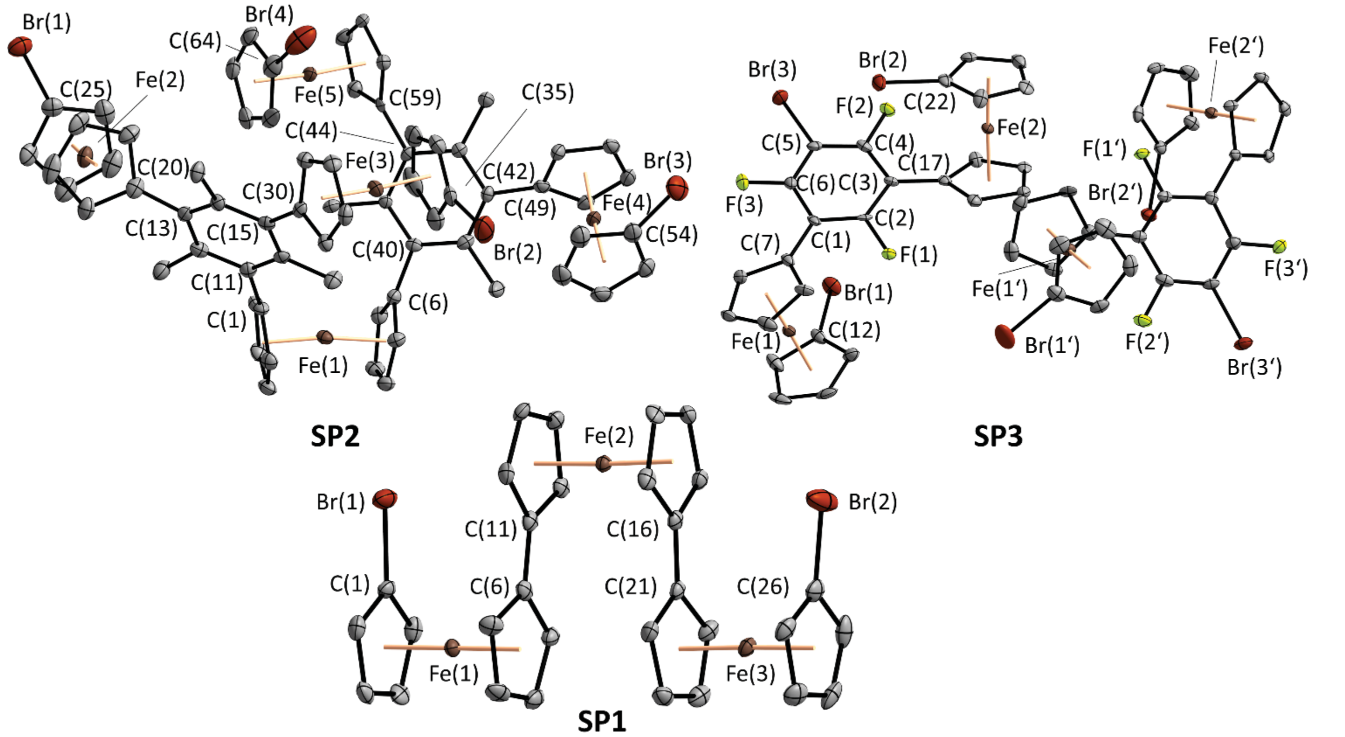

Fig. S3 Molecular structures of bromoferrocenylene-containing side products **SP1**, **SP2**, and **SP3** including part of their atom numbering scheme. As apparent from the depiction, **SP3** crystallises with two independent molecules in the asymmetric unit. Thermal ellipsoids are set at the 50% probability level, and co-crystallised solvent for **SP2** as well as hydrogen atoms have been omitted for clarity.

Crystallographically characterised side products **SP1**–**3** (Fig. S3) demonstrate the utility of the Negishi protocol for constructing complex molecular architectures from comparatively simple building blocks. Utilising the bromine substituents for further, step-wise functionalisation opens pathways for ferrocene-rich systems which might be of interest for molecular electronics and redox-switchable catalysis.

The structural parameters (Table S4) of **SP1**–**3** do not differ significantly from their intended counterparts **4b**,**d** regarding crucial bond lengths and angles. **SP1** is furthermore characterised by two almost perfectly coplanar fulvalenide bridges, likewise found for other, still very rarely prepared tris-/terferrocenes,[49,50] and the synperiplanar arrangement of the two bromoferrocenylene substituents. Pentaferrocenylene derivative **SP2** shows a significant bending of the internal ferrocenylene unit (α = 170.68°, Θ = 14.90°), illustrating the flexibility of ferrocene for accommodating sterically demanding substituents.

Table S4 Selected bond lengths [Å] and angles [°] of side products **SP1–3**; numbering scheme according to Fig. S3, ferrocenylene geometric parameters according to Fig. S2.

SP1	
C(1)–Br(1) / C(26)–Br(2)	1.879(3) 1.882(3)
C(6)–C(11) / C(16)–C(21)	1.457(4) 1.456(4)
Ct^{C}(1)–Fe(1) / Ct^{Br}(1)–Fe(1) / Ct^{C}(3)–Fe(3) / Ct^{Br}(3)–Fe(3)[a]	1.656 1.648 1.657 1.651
Ct^{Cp}(1)–Fe(2) / Ct^{Cp}(3)–Fe(2)[b]	1.650 1.650
α(fc(1)) / α(fc(2)) / α(fc(3))	178.48 177.57 177.89
Θ(fc(1)) / Θ(fc(2)) / Θ(fc(3))	1.96 3.69 2.55
τ(fc(1)) / τ(fc(2)) / τ(fc(3))	6.88 −5.35 −0.85
φ(fc(1)–fc(2)) φ(fc(2)–fc(3))[c]	2.62 3.58

SP2	
C(25)–Br(1) / C(35)–Br(2) / C(54)–Br(3) / C(64)–Br(4)	1.883(4) / 1.895(5) / 1.884(6) / 1.888(5)
C(11)–C(1) / C(6)–C(40) / C(13)–C(20) / C(15)–C(20) / C(42)–C(49) / C(44)–C(59) /	1.496(5) / 1.505(6) / 1.495(6) / 1.508(6) / 1.500(7) / 1.507(5)
Ct^{C}(1)–Fe(1) / Ct^{C}(2)–Fe(1) / Ct^{C}(3)–Fe(2) / Ct^{C}(4)–Fe(3) / Ct^{C}(5)–Fe(4) / Ct^{C}(6)–Fe(5)[a]	1.679 / 1.675 / 1.646 / 1.653 / 1.650 / 1.663
Ct^{Br}(3)–Fe(2) / Ct^{Br}(4)–Fe(3) / Ct^{Br}(5)–Fe(4) / Ct^{Br}(6)–Fe(5)[a]	1.642 / 1.644 / 1.640/ 1.649
α(fc(1)) / α(fc(2)) / α(fc(3)) / α(fc(4)) / α(fc(5))	170.68 / 176.42 / 174.96 / 175.73 / 175.77
Θ(fc(1)) / Θ(fc(2)) / Θ(fc(3)) / Θ(fc(4)) / Θ(fc(5))	14.90 / 6.94 / 8.06 / 6.97 / 7.51
τ(fc(1)) / τ(fc(2)) / τ(fc(3)) / τ(fc(4)) / τ(fc(5))	26.79 / 147.68 / 139.03 147.40 / 90.54
$φ_1$(fc(1)) / $φ_2$(fc(1)) / φ(fc(2)) / φ(fc(3)) / φ(fc(2)) / φ(fc(3))[d]	60.43 / 62.13 / 57.00 / 47.33 / 54.02 / 52.03

SP3 [e]		
C(12)–Br(1) / C(22)–Br(2) / C(5)–Br(3)	1.880(7) 1.895(8) 1.870(7)	1.894(7) 1.882(7) 1.879(7)
C(1)–C(7) / C(3)–C(17) /	1.483(9) 1.472(9)	1.476(9) 1.479(9)
Ct^{C}(1)–Fe(1) / Ct^{C}(2)–Fe(2)[a]	1.643 1.641	1.650 1.644
Ct^{Br}(1)–Fe(1) / Ct^{Br}(2)–Fe(2)[a]	1.644 1.639	1.644 1.636
C(2)–F(1) / C(4)–F(2) / C(6)–F(3)	1.343(8) 1.347(7) 1.342(7)	1.342(8) 1.347(8) 1.348(8)
α(fc(1)) / α(fc(2)) /	178.14 178.46	177.55 179.34
Θ(fc(1)) / Θ(fc(2)) /	2.08 1.22	2.81 0.91
τ(fc(1)) / τ(fc(2)) /	-21.15 10.45	-136.7 -30.45
φ(fc(1)) / φ(fc(2)) /	24.97 28.38	29.25 32.88

[a] Ct^{C} denotes the calculated centre of gravity of the carbon-substituted cyclopentadienyl ring, and Ct^{Br} denotes the calculated centre of gravity of the bromine-substituted cyclopentadienyl ring; [b] Ct^{Cp} denotes the calculated centre of gravity of the cyclopentadienyl-substituted cyclopentadienyl ring; [c] Twist about the cyclopentadienyl-cyclopentadienyl axis; [d] $φ_{1/2}$ differentiates between the two cyclopentadienyl rings of the central ferrocenylene unit (Fe(1)); [e] Values for the second molecule in the asymmetric unit (same numbering scheme) in grey.

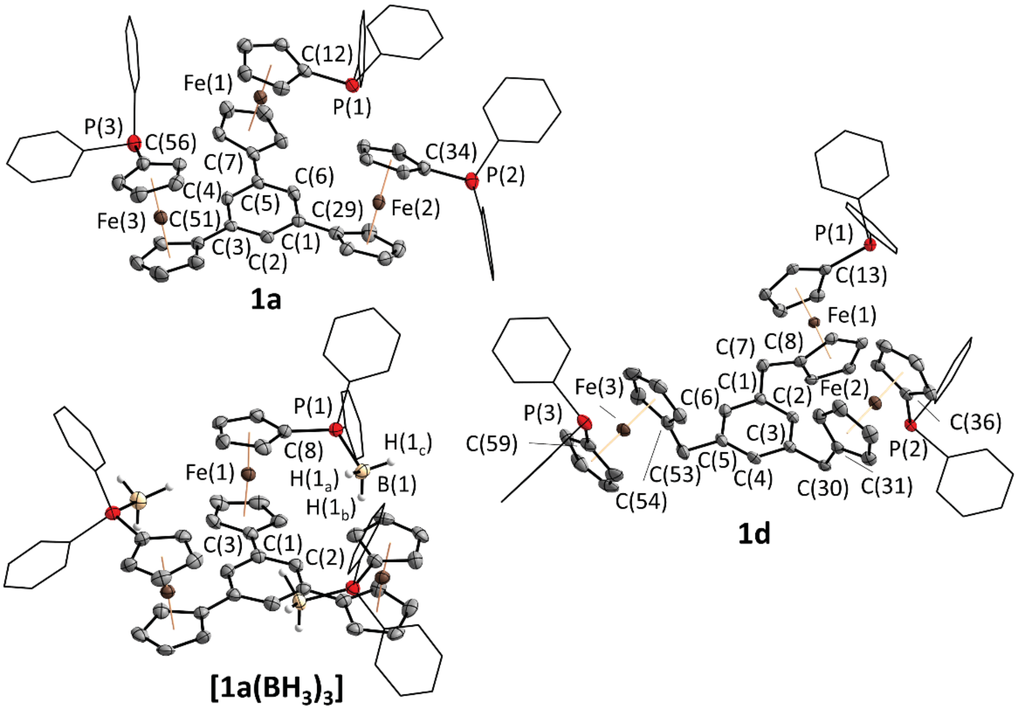

Fig. S4 Molecular structures of tris-phosphanes **1a** and **1d** and the borane adduct **[1a(BH$_3$)$_3$]** including part of their atom numbering scheme. Thermal ellipsoids are set at the 50% probability level, disorder in one phenyl ring of **1a**, co-crystallised solvent in **1d** and hydrogen atoms except for the BH$_3$ moieties of **[1a(BH$_3$)$_3$]** have been omitted for clarity.

SUPPORTING INFORMATION

Table S5 Selected bond lengths [Å] and angles [°] of tris-phosphanes **1a** and **1d** and the borane adduct **[1a(BH$_3$)$_3$]**; numbering scheme according to Fig. S4, ferrocenylene geometric parameters according to Fig. S2.

	1a	1d	[1a(BH$_3$)$_3$][a]		1a	1d	[1a(BH$_3$)$_3$][a]
**$C^{P}_{ips}(fc_1)$–P(1) /**	1.835(4)	1.810(2)		**α(fc(1)) /**	179.33	177.78	
**$C^{P}_{ips}(fc_2)$–P(2) /**	1.817(4)	1.817(2)	1.783(4)	**α(fc(2)) /**	177.74	178.26	176.54
**$C^{P}_{ips}(fc_3)$–P(3)**[b]	1.822(3)	1.811(3)		**α(fc(3)) /**	179.77	177.44	
**$C^{C}_{ips}(fc_1)$–C^{Link}(1) /**	1.483(5)	1.500(3)		**Θ(fc(1)) /**	1.28	2.81	
**$C^{C}_{ips}(fc_2)$–C^{Link}(2) /**	1.477(5)	1.507(3)	1.477(9)	**Θ(fc(2)) /**	3.86	1.99	3.42
**$C^{C}_{ips}(fc_3)$–C^{Link}(3)**[c]	1.471(5)	1.493(3)		**Θ(fc(3))**	0.51	2.70	
**Ct^{C}(1)–Fe(1) /**	1.650	1.636		**τ(fc(1)) /**	82.03	139.64	
**Ct^{C}(2)–Fe(2) /**	1.655	1.656	1.647	**τ(fc(2)) /**	153.96	−24.81	84.30
**Ct^{C}(3)–Fe(3)**[d]	1.645	1.644		**τ(fc(3))**	68.09	154.61	
**Ct^{P}(1)–Fe(1) /**	1.642	1.628		**φ(fc(1)) /**	14.64		
**Ct^{P}(2)–Fe(2) /**	1.655	1.651	1.642	**φ(fc(2)) /**	27.66	-	8.81
**Ct^{P}(3)–Fe(3)**[e]	1.642	1.643		**φ(fc(3))**	19.92		
**Other relevant bond lengths**	-	C(1)–C(7): 1.517(3)<br>C(3)–C(30): 1.500(3)<br>C(5)–C(53): 1.516(3)	P(1)–B(1): 1.925(9)				

[a] Due to crystallographic C_3 symmetry, only one value is shown; [b] $C^{P}_{ips}(fc_n)$ corresponds to the respective *ipso* carbon atom of the P-substituted cyclopentadienyl ring (**1a**: C(12), C(34), C(56); **1d**: C(13), C(36), C(59); **[1a(BH$_3$)$_3$]**: C(8)); [c] C^{Link}(n) refers to the C atom to which the C-substituted cyclopentadienyl is bound (**1a**: C(1), C(3), C(5); **1d**: C(7), C(30), C(57); **[1a(BH$_3$)$_3$]**: C(1)); [d] Ct^{C} denotes the calculated centre of gravity of the carbon-substituted cyclopentadienyl ring; [e] Ct^{P} denotes the calculated centre of gravity of the P-substituted cyclopentadienyl ring.

The solid-state molecular structures of **1a** and **1d** (Table S5) show virtually identical parameters relevant for their use as ligands in coordination chemistry, indicating that the change in the arene backbone does not influence the diphenylphosphanyl moiety. The all-*syn* conformation and other structural parameters of **1a** are similar to **1c**.[13]
Converting **1a** to **[1a(BH$_3$)$_3$]** leads to a significant shortening of the C^{P}_{ips}–P bond, while the other bond lengths and distances of the tris(ferrocenylene)benzene core remain very similar. The twist between the C_6H_3 core and the cyclopentadienyl rings of **[1a(BH$_3$)$_3$]** is notably smaller (8.81° *vs.* 14.64–27.66°); however, as all compounds display free rotation about the C^{C}_{ips}(fc)–C^{Link} bonds, these solid-state parameters are of limited practical value. It is interesting to note that, just as **1a** and **1c** are very similar in most structural aspects, so are their BH$_3$ analogues, including the crystallographic C_3 symmetry represented by space group $R\bar{3}$.[13]

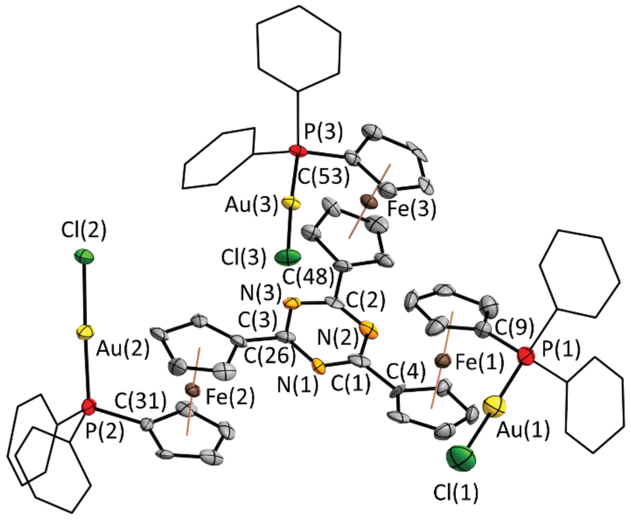

[1c(Au)$_3$]

Fig. S5 Molecular structure of homotrinuclear complex **[1c(Au)$_3$]** including part of the atom numbering scheme. Thermal ellipsoids are set at the 50% probability level. *P*-bound phenyl rings are depicted in wireframe style. Disorder in gold(I) chloride fragment Au(2)–Cl(2) of **[1c(Au)$_3$]**, co-crystallised solvent and hydrogen atoms have been omitted for clarity.

SUPPORTING INFORMATION

Table S6 Selected bond lengths [Å], distances [Å], and angles [°] of homotrinuclear complex **[1c(Au)$_3$]**; numbering scheme according to Fig. S5, ferrocenylene geometric parameters according to Fig. S2.

	[1c(Au)$_3$]		[1c(Au)$_3$]
**P(1)–Au(1) / Au(1)–Cl(1) /**	2.231(6) / 2.286(7) /	**C(1)–N(1) /**	1.30(2) /
**P(2)–Au(2) / Au(2)–Cl(2) /**	2.225(5) / 2.288(6) /	**N(1)–C(2) /**	1.34(3) /
**P(3)–Au(3) / Au(3)–Cl(3)**	2.229(4) / 2.281(5)	**C(2)–N(2) /**	1.34(2) /
**Au(1)···Au(2) /**	14.187(1) /	**N(2)–C(3) /**	1.35(2) /
**Au(2)···Au(3) /**	8.012(1) /	**C(3)–N(3) /**	1.31(3) /
**Au(3)···Au(1)**[a]	9.100(1)	**N(3)–C(1)**	1.32(2)
**Au(1)···Fe(1) /**	4.302(3) /	**∡(P(1)–Au(1)–Cl(1)) /**	179.0(2) /
**Au(2)···Fe(2) /**	4.080(3) /	**∡(P(2)–Au(2)–Cl(2)) /**	177.0(2) /
**Au(3)···Fe(3)**[b]	4.544(2)	**∡(P(3)–Au(3)–Cl(3))**	176.9(2)
**C(9)–P(1) /**	1.80(2) /	**α(fc(1)) /**	175.9 /
**C(31)–P(2) /**	1.80(2) /	**α(fc(2)) /**	176.5 /
**C(53)–P(3)**	1.80(2)	**α(fc(3))**	176.0
**C(4)–C(1) /**	1.54(3) /	**Θ(fc(1)) /**	5.1 /
**C(26)–C(2) /**	1.46(2) /	**Θ(fc(2)) /**	3.1 /
**C(48)–C(3)**	1.47(2)	**Θ(fc(3))**	3.9
**Ct^C(1)–Fe(1) /**	1.65 /	**τ(fc(1)) /**	169.2 /
**Ct^C(2)–Fe(2) /**	1.65 /	**τ(fc(2)) /**	−141.5 /
**Ct^C(3)–Fe(3)**[c]	1.65	**τ(fc(3))**	−69.2
**Ct^P(1)–Fe(1) /**	1.65 /	**φ(fc(1)) /**	15.2 /
**Ct^P(2)–Fe(2) /**	1.65 /	**φ(fc(2)) /**	10.5 /
**Ct^P(3)–Fe(3)**[d]	1.66	**φ(fc(3))**	4.4

[a] Intramolecular metal-metal distances; the shortest intermolecular Au-Au distance 6.4204(9) Å; [b] Intramolecular separation between gold and the corresponding iron centre; [c] Ct^C denotes the calculated centre of gravity of the carbon-substituted cyclopentadienyl ring; [d] Ct^P denotes the calculated centre of gravity of the P-substituted cyclopentadienyl ring.

Due to poor quality of the only available single crystals of **[1c(Au)$_3$]**, bond lengths and angles are less accurate than for the tris-phosphanes and bromoferrocenylene precursors. The large residual electron densities around the gold atoms are presumably caused by this low crystal quality, as employing other absorption correction methods did not yield better results, and no disorder can unambiguously be assigned to the structure model. Despite numerous attempts, we were not able to grow crystals of better quality. However, the data are still sufficient to unambiguously prove the chemical structure of the given compound.

SUPPORTING INFORMATION

Table S7 Comparison of solid-state molecular structures of trinuclear gold complexes containing three metal-bound phosphorus atoms (excl. phosphides) listed in the CSD. Entries are sorted alphabetically. Only non-polymeric entries have been considered and analysed in terms of tris-phosphane ligand, approximate C_3 (or higher) symmetry (given in brackets if potentially so in solution) and for the presence of aurophilic interactions (given in brackets if not recognised as such in the corresponding CSD entry but matching the distance criterion of d(Au···Au) < 3.5 Å).[51]

CSD Identifier Code	Oxidation State of Gold	Tris-Phosphane Ligand	C_3 symmetry	Aurophilic Interactions
**ACUZIH**	+I	X		X
**BEPGUY**	+I	X		(X)[a]
**BOKDIO**	+I	X	(X)	X
**CIFWIY**	+I		X	
**DENDUW**	+III	X		
**DENFOS**	+III	X		
**DODQOC**	+I	X	(X)	(X)
**DODQUI**	+I	X		(X)
**FISPOO**	+I	X		X
**FISQIJ**	+I	X		X
**FONJOJ**	+I	X	X	X
**GATSOK**	+I		X	(X)[a]
**GATSUQ**	+I		X	(X)[a]
**IDOZUY**[b]	+I	X	X	X
**KORBUO / KORCAV**[c]	+I	X	X	X
**KUGYUH**	+I	X	(X)	X
**LIGZOQ**	+I		X	
**LUWHER**	+I	X	X	
**MAFQAM**	+I		X	
**NEDFOS**	+I		X	
**NEVXER**	+I	X	X	
**NIMPEE**	+I		X	
**NIXSUI**	+I	X	X	(X)[a]
**NIXTAP**	+I	X	X	
**NIXTET**	+I	X	X	
**NOFMIF**	+I	X	X	(X)
**OBATUG**	+I	X	X	(X)
**OBAVAO**	+I	X	X	(X)
**OCILOD**	+I	X	X	(X)
**POWSAV**	+I	X		X
**POYCUB**	+I		X	(X)[a]
**RAPDUH**	+I	X	X	(X)[a]
**REVTAN**	+I	X	X	X
**SASWEQ**	+I	X	X	(X)[a]
**SASWAM**	+I	X	X	(X)
**UBIPOK**	+III	X		
**UYUVOB**	+I	X	X	X
**VIVMAQ / VIVMEU**[c]	+I	X	X	
**WAQJEF**	+I	X	X	X
**WAQJIJ**	+I	X	X	X
**WAQJOP**	+I	X	X	X
**XADZIL**	+I	X		X
**YOSWAG**	+I	X	X	
**ZIHVUJ**	+I	X	X	X
**ZIHWAQ**	+I	X	X	X
**ZIHWEU / ZIHWIY**[c]	+I	X	X	X
**ZIHWOE**	+I	X	(X)	X
**ZIHWUK**	+I	X	(X)	X

[a] Close intermolecular Au···Au contact; [b] Contains three ferrocenyl groups, yet not as part of the tris-phosphane but as three individual alkynylferrocenes; [c] Identical complexes only differing in co-crystallised solvent.

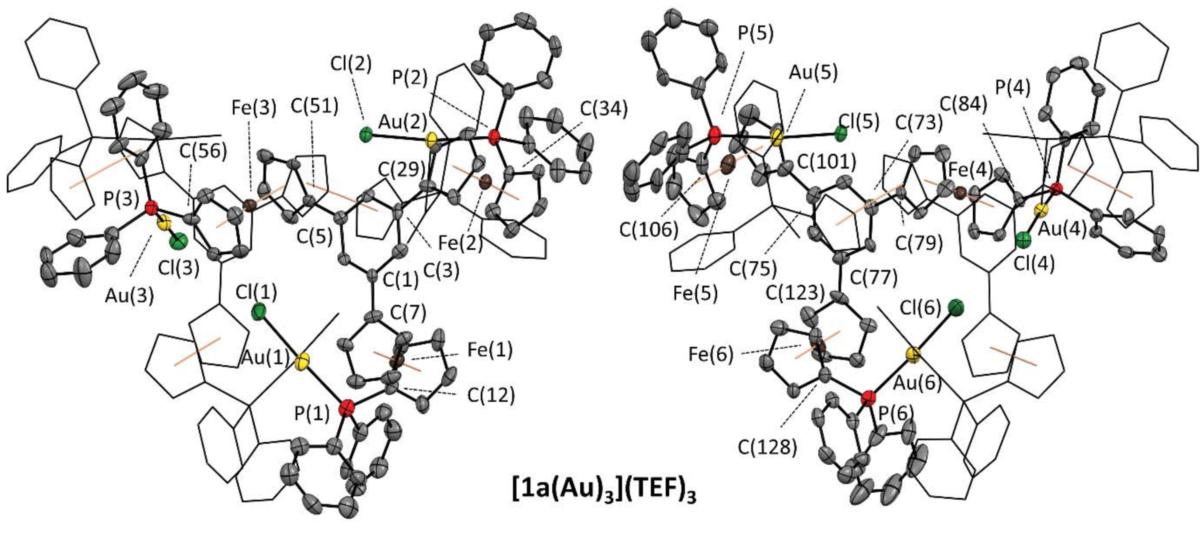

Fig. S6 Molecular structure of trioxidised complex {**[1a(Au)$_3$]**}$_2$**(TEF)$_6$** including part of the atom numbering scheme. For better visibility, the two fragments making up the hexa-cationic dimer have been labelled individually, the respective other half depicted in wireframe style in roughly the same orientation. Thermal ellipsoids are set at the 50% probability level. Co-crystallised solvent, the four localised teflonate anions, and hydrogen atoms have been omitted for clarity. The left fragment is in front of the fragment depicted on the right side.

Table S8 Selected bond lengths [Å], distances [Å], and angles [°] of triply oxidised complex **[1a(Au)$_3$](TEF)$_3$**; numbering scheme according to Fig. S6, ferrocenylene geometric parameters according to Fig. S2.

		[1a(Au)$_3$](TEF)$_3$				[1a(Au)$_3$](TEF)$_3$	
**P(1)–Au(1) /**	**P(4)–Au(4) /**	2.240(5) /	2.220(4) /	**∡(P(1)–Au(1)–Cl(1)) /**		175.7(2) /	
**P(2)–Au(2) /**	**P(5)–Au(5) /**	2.217(4) /	2.230(4) /	**∡(P(2)–Au(2)–Cl(2)) /**		170.8(1) /	
**P(3)–Au(3)**	**P(6)–Au(6)**	2.221(5)	2.215(3)	**∡(P(3)–Au(3)–Cl(3)) /**		176.4(2)	
**Au(1)–Cl(1) /**	**Au(4)–Cl(4) /**	2.215(3) /	2.278(4) /	**∡(P(4)–Au(4)–Cl(4)) /**		171.8(1) /	
**Au(2)–Cl(2) /**	**Au(5)–Cl(5) /**	2.285(4) /	2.278(4) /	**∡(P(5)–Au(5)–Cl(5)) /**		171.8(1) /	
**Au(3)–Cl(3)**	**Au(6)–Cl(6)**	2.286(5)	2.255(4)	**∡(P(6)–Au(6)–Cl(6))**		171.4(2)	
**Au(1)–Au(6) /**		2.989(1) /		**∡(P(1)–Au(1)–Au(6)–P(6)) /**		88.9(2) /	
**Au(2)–Au(4) /**		3.188(1) /		**∡(P(2)–Au(2)–Au(4)–P(4)) /**		−82.2(2) /	
**Au(3)···Au(5)**[a]		3.397(1)		**∡(P(3)–Au(3)···Au(5)–P(5))**		−83.7(2)	
**Au(1)···Fe(1) /**	**Au(4)···Fe(4) /**	4.163(2) /	4.097(2) /	**α(fc(1)) /**	**α(fc(4)) /**	178.0 /	177.2 /
**Au(2)···Fe(2) /**	**Au(5)···Fe(5) /**	4.269(2) /	4.176(2) /	**α(fc(2)) /**	**α(fc(5)) /**	178.8 /	176.8 /
**Au(3)···Fe(3)**	**Au(6)···Fe(6)**[b]	4.202(2)	4.136(2)	**α(fc(3))**	**α(fc(6))**	175.9	178.4
**Ct^C(1)–Fe(1) /**	**Ct^C(4)–Fe(4) /**	1.71 /	1.72 /	**Θ(fc(1)) /**	**Θ(fc(4)) /**	2.1 /	1.3 /
**Ct^C(2)–Fe(2) /**	**Ct^C(5)–Fe(5) /**	1.70 /	1.71 /	**Θ(fc(2)) /**	**Θ(fc(5)) /**	2.6 /	2.4 /
**Ct^C(3)–Fe(3)**	**Ct^C(6)–Fe(6)**[c]	1.71	1.72	**Θ(fc(3))**	**Θ(fc(6))**	3.3	2.6
**Ct^P(1)–Fe(1) /**	**Ct^P(4)–Fe(4) /**	1.66 /	1.72 /	**τ(fc(1)) /**	**τ(fc(4)) /**	−110.4 /	−171.4 /
**Ct^P(2)–Fe(2) /**	**Ct^P(5)–Fe(5) /**	1.71 /	1.71 /	**τ(fc(2)) /**	**τ(fc(5)) /**	87.5 /	85.3 /
**Ct^P(3)–Fe(3)**	**Ct^P(6)–Fe(6)**[d]	1.72	1.70	**τ(fc(3))**	**τ(fc(6))**	−168.5	−110.6
**C(1)–C(7) /**	**C(73)–C(79) /**	1.44(2) /	1.48(2) /	**φ(fc(1)) /**	**φ(fc(4)) /**	8.5 /	28.8 /
**C(3)–C(29) /**	**C(75)–C(101) /**	1.50(1) /	1.47(3) /	**φ(fc(2)) /**	**φ(fc(5)) /**	29.8 /	19.9 /
**C(5)–C(51)**	**C(77)–C(123)**	1.47(2)	1.46(2)	**φ(fc(3))**	**φ(fc(6))**	18.0	8.1
**C(12)–P(1) /**	**C(84)–P(4) /**	1.82(1) /	1.82(1) /				
**C(34)–P(2) /**	**C(106)–P(5) /**	1.83(2) /	1.78(2) /				
**C(56)–P(3)**	**C(128)–P(6)**	1.80(2)	1.83(2)				

[a] The contacts between Au(1) and Au(6) as well as between Au(2) and Au(4) are in the range commonly accepted for true aurophilic interactions, while the distance between Au(3) and Au(5), though well below the sum of the van-der-Waals radii for two gold atoms, is thought of as too large for significant bonding interactions[51]; [b] Intramolecular separation between gold and the corresponding iron centre; [c] Ct^C denotes the calculated centre of gravity of the carbon-substituted cyclopentadienyl ring; [d] Ct^P denotes the calculated centre of gravity of the P-substituted cyclopentadienyl ring.

Due to quality of the only available single crystals of **{[1a(Au)$_3$]}$_2$(TEF)$_6$**, bond lengths and angles are less accurate than for the tris-phosphanes and bromoferrocenylene precursors. Weak residual electron density is found close to the gold atoms; potentially, a part of the dimeric hexa-cation suffers from disorder, which however could not be properly modelled. Due to relatively weak scattering and strong disorder, only four of the six teflonate anions necessary to balance the hexa-cationic charge of the

{[1a(Au)$_3$]$_2$}$^{6+}$ dimer could be crystallographically localised and refined accordingly. The presence of the other two teflonate anions was deduced from the electron count, as 1992 electrons per unit cell have been removed using the SQUEEZE routine implemented in PLATON.[19] Four teflonate anions (465 electrons per anion) require 1860 electrons; the missing 132 electrons are most likely contributed from the solvents of crystallisation, 1,2-dichloroethane (50 electrons per molecule) and pentane (40 electrons per molecule). In the same way, a volume of 955 Å^3 per teflonate anion (as judged from the unoccupied volume, assuming dense packing in the asymmetric unit) compares well to the literature value of 17 Å^3 / non-hydrogen atom (one teflonate anion consists of 57 atoms, thus a value of 16.8 Å^3 / atom results).[52] As shown in Fig. S7, void space is clearly visible in the final structure solution, leaving room for four additional teflonate anions per unit cell. In the space-filling model (Fig. S7, right), the separation of the hexa-cationic dimers by co-crystallised solvent and the teflonate anions is illustrated. The closest separation between two of such dimers has been determined to about 4.65 Å (hydrogen-to-hydrogen distance between H120 (phenyl ring at P5) and H98 (ferrocenyl moiety at Fe4 of a neighbouring dimer).

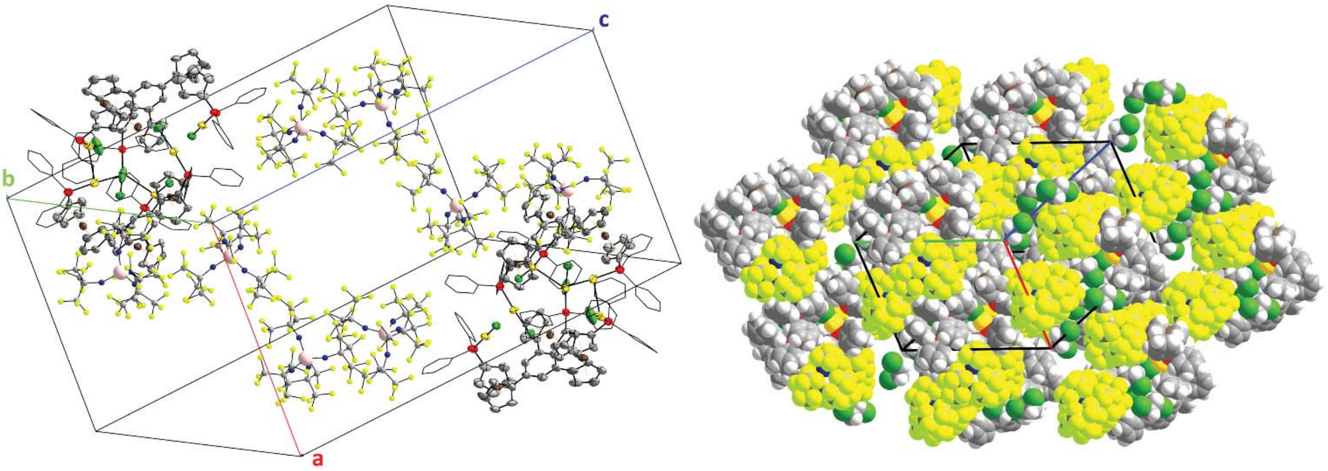

Fig. S7 Left: Unit cell of trioxidised **{[1a(Au)$_3$]}$_2$(TEF)$_6$** with labelled cell axes. Anions / *P*-bound phenyl rings are depicted in ball-and-stick / wireframe style, respectively. Thermal ellipsoids are set at the 50% probability level. Co-crystallised solvent, disordered CF_3 groups in the anions, and hydrogen atoms are omitted for clarity. Right: Space-filling model of trioxidised **{[1a(Au)$_3$]}$_2$(TEF)$_6$**, unit cell axes highlighted.

3. Electrochemical data

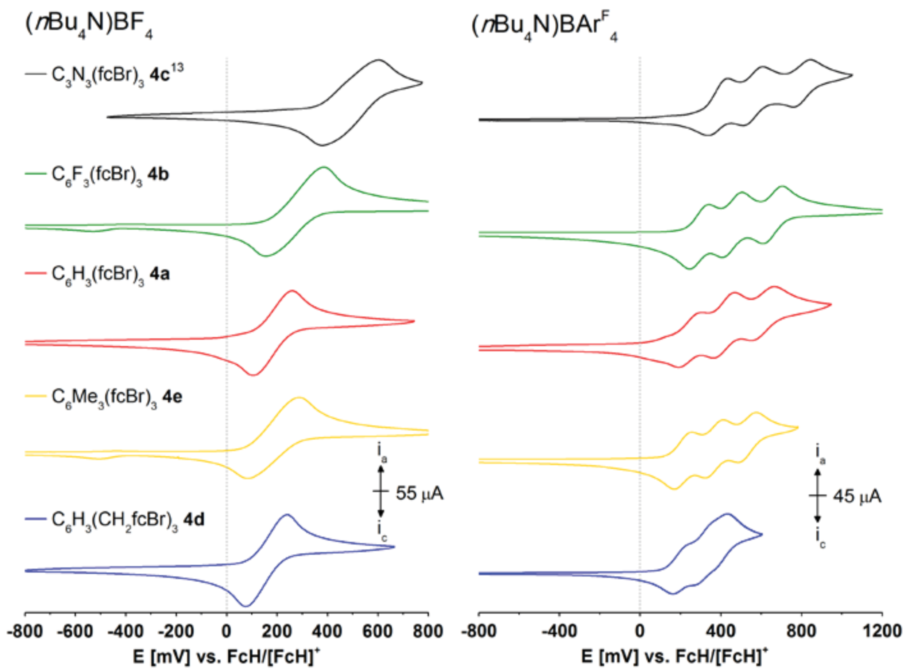

Fig. S8 Cyclic voltammograms of tris(1-bromo-1'-ferrocenylene)arenes **4a–e** in the BF_4^-- (left) and $BAr^F{}_4^-$-based (right) supporting electrolytes, ordered from lowest to highest oxidation potential. For all voltammograms, the second of three consecutively measured cycles is shown, the scan rate was set to 100 mV·s^{-1}, and scans have been carried out in anodic direction. The cyclic voltammograms of **4c** have been reported before and are included here for completeness.[13]

Table S9 Redox potentials of the tris(1-bromo-1'-ferrocenylene)arenes **4a–e** in two different supporting electrolytes determined by cyclic voltammetry, sorted from lowest to highest formal oxidation potential.[a] Electrochemical data for **4c** have been reported before and are included here for completeness.[13]

**Electrolyte**	**(nBu$_4$N)BF$_4$/CH$_2$Cl$_2$**	**(nBu$_4$N)BAr$^F{}_4$/CH$_2$Cl$_2$**			
**Potentials [mV]**	**E° (ΔE$_p$)^b**	**E°$_1$ (ΔE$_p$)^c**	**E°$_2$ (ΔE$_p$)^c**	**E°$_3$ (ΔE$_p$)^c**	**ΔE°$_{1/2}$ / ΔE°$_{2/3}$^{d}**
$C_6H_3(CH_2fcBr)_3$ **4d**	142 (179)	195 (85)^e	320 (95)^e	400 (75)^e	125 / 80
$C_6Me_3(fcBr)_3$ **4e**	180 (214)^f	212 (87)	369 (94)	530 (92)	157 / 161
$C_6H_3(fcBr)_3$ **4a**	182 (159)	247 (100)	416 (94)	605 (99)	169 / 189
$C_6F_3(fcBr)_3$ **4b**	271 (231)^g	293 (96)	456 (98)	656 (96)	163 / 200
$C_3N_3(fcBr)_3$ **4c**[13]	339 (207)	386 (127)	560 (110)	808 (101)	174 / 248

^{a} Redox potential *vs*. the FcH/[FcH]$^+$ couple at a glassy carbon working electrode (scan rate 100 mV·s^{-1}), the splitting between the anodic (oxidation) and cathodic (reduction) peaks given in brackets; ^{b} Determined in an anhydrous 0.1 mol·L^{-1} (nBu$_4$N)BF$_4$/CH$_2$Cl$_2$ solution; ^{c} Determined in an anhydrous 0.1 mol·L^{-1} (nBu$_4$N)BAr$^F{}_4$/CH$_2$Cl$_2$ (BAr$^F{}_4$ = [B{3,5-(CF$_3$)$_2$C$_6$H$_3$}$_4$]) solution; ^{d} Peak-to-peak separation of the first and second/second and third redox process in (nBu$_4$N)BAr$^F{}_4$/CH$_2$Cl$_2$; ^{e} Due to the close spacing of the peaks, an exact peak analysis is difficult and values have been determined to the nearest 5 mV; ^{f} Not fully reversible; additional, scan rate-dependent reduction peak at −437 mV (cf. Fig. S9); ^{g} Not fully reversible; additional, scan rate-dependent reduction peak at −534 mV (cf. Fig. S9).

All tris(1-bromo-1'-ferrocenylene)arenes show one broad, quasi-reversible redox event in the BF_4^--based SE (Fig. S8, left). For the mesitylene (**4e**) and trifluorobenzene (**4b**) cores, a noticeable deviation from reversibility ($i_a/i_c \neq 1$) is linked to a cathodically shifted reduction event which depends on prior oxidation (cf. Fig. S9). This behaviour points towards a fast chemical reaction following the initially Fe-centred oxidation (EC mechanism),[13] generating a species which is harder to reduce (and which can be re-oxidised, too) appearing after the first scan. As this behaviour is linked to 1,3,5-trisubstitued arene cores, this phenomenon is most likely linked to the substituents themselves. Fluorinated (hetero)aromatics are known to generate radical cations and anions on the electrochemical timescale, supporting this hypothesis.[53–55]

In contrast, **4a–e** yield well-defined cyclic voltammograms when the $BAr^F{}_4^-$-based SE is employed (Fig. S8, left), and without indications for follow-up chemistry. With the exception of **4d**, the three consecutive oxidation steps are separated by more than

150 mV each and the generation of the trication ($\Delta E^0_{2/3}$ in Table S9) is more difficult than that of the dication ($\Delta E^0_{1/2}$ in Table S9). Given the disconjugated nature of **4d** due to its methylene spacers, the individual ferrocenylene moieties behave more independently and no mixed-valency behaviour is to be expected. It is thus most likely that the peak separations $\Delta E^0_{1/2}$(**4d**) and $\Delta E^0_{2/3}$(**4d**) reflect purely electrostatic effects,[56] while the other compounds might experience a certain stabilisation in their mono- and dicationic states.

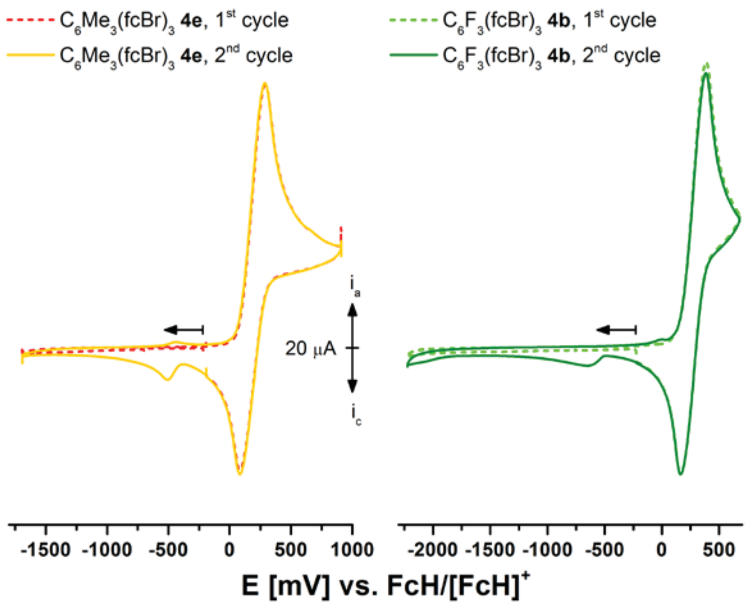

Fig. S9 Cyclic voltammograms of tris(1-bromo-1'-ferrocenylene)arenes **4e** (left, orange/red) and **4b** (right, light green/dark green) in the BF_4^--based supporting electrolyte. The first (dashed lines) and second (solid line) of three consecutively measured cycles are shown. Arrows represent the starting potential and initial scan direction (100 mV·s^{-1}).

Table S10 Oxidation and reduction peak potentials of the tris(1-diphenylphosphanyl-1'-ferrocenylene)arenes **1a**–**d** in two different supporting electrolytes determined by cyclic voltammetry, sorted from lowest to highest first oxidation potential. [a] Electrochemical data for **1c** have been reported before and are included here for completeness.[13]

**Electrolyte**	**(nBu$_4$N)BF$_4$/CH$_2$Cl$_2$**		**(nBu$_4$N)BAr$^F_4$/CH$_2$Cl$_2$**		
**Potentials [mV]**	**E^{ox}** [b]	**E^{red}** [b]	**E$^0_1$ (ΔE$_p$)**[c]	**E^{ox}** [d]	**E^{red}** [d]
$C_6H_3(CH_2fcPPh_2)_3$ **1d**	146 / 644	122 / 302	113 / 228 [e]	345 / 858	-
$C_6H_3(fcPPh_2)_3$ **1a**	193 / 635	209 / 337	138 (98)	351 / 430 / 853	104
$C_6F_3(fcPPh_2)_3$ **1b**	233 / 733	52 / 248 / 413 / 611 (sh)	206 (116)	420 / 558	174 / −1348
$C_3N_3(fcPPh_2)_3$ **1c**[13]	339 / 431 (sh) / 826	267 / 451 / −1387	275 (160)	512 / 865	188 / −640 (br) / −1472

[a] Peak potential *vs.* the FcH/[FcH]$^+$ couple at a glassy carbon working electrode (scan rate 100 mV·s^{-1}), individual processes separated by the "/" sign; [b] Peak potentials, determined in an anhydrous 0.1 mol·L^{-1} (nBu$_4$N)BF$_4$/CH$_2$Cl$_2$ solution; [c] Redox potential of reversible first oxidation with the splitting between the anodic (oxidation) and cathodic (reduction) peaks given in brackets, determined in an anhydrous 0.1 mol·L^{-1} (nBu$_4$N)BAr$^F_4$/CH$_2$Cl$_2$ (BAr$^F_4$ = [B{3,5-(CF$_3$)$_2$C$_6$H$_3$}$_4$]) solution; [d] Peak potentials, determined in an anhydrous 0.1 mol·L^{-1} (nBu$_4$N)BAr$^F_4$/CH$_2$Cl$_2$ solution; [e] Due to the close spacing of the first three oxidation events, square-wave voltammetry was employed to determine the formal potentials of the first two reversible oxidations, entailing the non-determination of the anodic-cathodic peak-to-peak separation.

The redox behaviour of the non-coordinated or unprotected tris-phosphanes **1a**–**d** is dominated by the well-known irreversibility of the first oxidation, which has been attributed to either an involvement of the lone pair of electrons at the phosphorus atom in the HOMO (highest occupied molecular orbital) or by electron transfer from said lone pair of electrons onto the generated iron(III) ion immediately after oxidation.[57–59] While the first oxidation on its own is irreversible in the BF_4^--based SE (Fig. S10, left), it appears more (quasi-)reversible in the $BAr^F_4{}^-$-based SE (Fig. S10, right). In the case of the benzylic arene core of **1d** (blue), even the first two oxidations (dotted lines) are reversible, while for the other arene cores the second oxidation results in a loss of reversibility of the first. In both SE, further oxidation events, potentially *P*-centred, are recorded at higher potentials. The *s*-triazine core of **1c** leads to the appearance of cathodically shifted reduction events upon former oxidation in both supporting electrolytes as we have previously noted.[13]

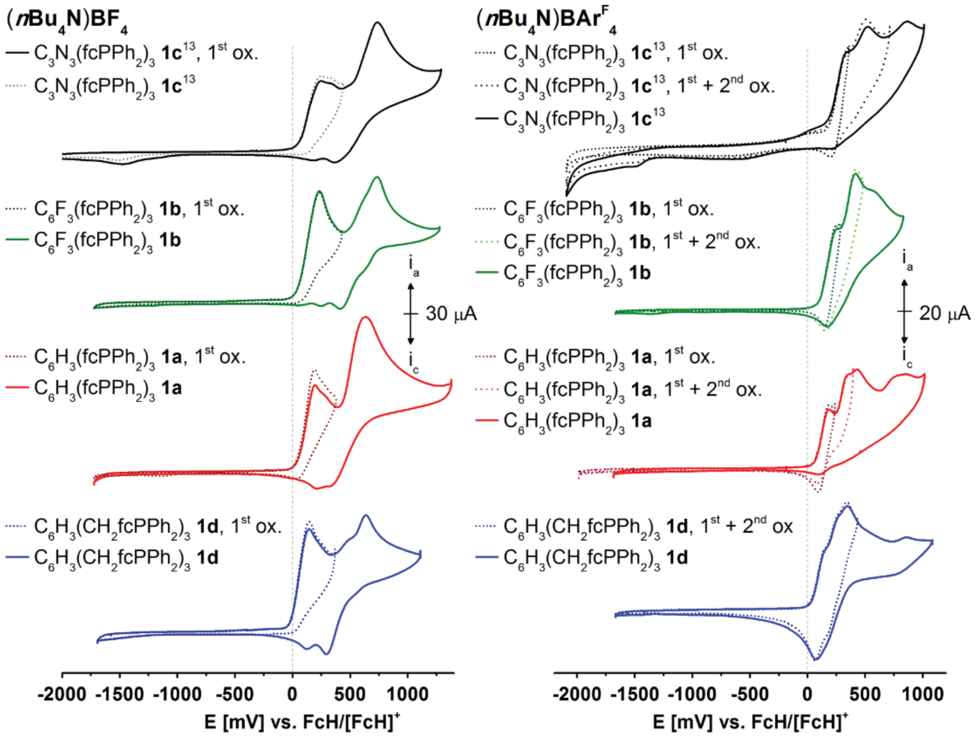

Fig. S10 Cyclic voltammograms of tris(1-diphenylphosphanyl-1'-ferrocenylene)arenes **1a–d** in the BF_4^-- (left) and $BAr^F{}_4^-$-based (right) supporting electrolytes, ordered from lowest to highest oxidation potential. For all voltammograms, the second of three consecutively measured cycles is shown, the scan rate was set to 100 mV·s⁻¹, and scans have been carried out in anodic direction. Dashed and dotted lines represent scans with lower anodic vertex potentials, seeking to explore the electrochemical behaviour of the tris(ferrocenyl)arene core only. The cyclic voltammograms of **1c** have been reported before and are included here for completeness.[13]

Table S11 Formal redox potentials of the tris(1-diphenylphosphanylborane-1'-ferrocenylene)arenes **[1(BH$_3$)$_3$]** in the two different supporting electrolytes determined by cyclic voltammetry, sorted from lowest to highest first oxidation potential. [a] Electrochemical data for [**1c(BH$_3$)$_3$**] have been reported before and are included here for completeness.[13]

Electrolyte	(nBu$_4$N)BF$_4$/CH$_2$Cl$_2$	(nBu$_4$N)BAr$^F{}_4$/CH$_2$Cl$_2$			
**Potentials [mV]**	**E° (ΔE$_p$)[b]**	**E°$_1$ (ΔE$_p$)[c]**	**E°$_2$ (ΔE$_p$)[c]**	**E°$_3$ (ΔE$_p$)[c]**	**ΔE°$_{1/2}$ / ΔE°$_{2/3}$[d]**
**[1a(BH$_3$)$_3$]**	279 (201)	270 (91)	457 (103)	626 (101)	187 / 169
**[1b(BH$_3$)$_3$]**	346 (168)[e]	353 (87)	523 (109)	676 (130)	170 / 153
**[1c(BH$_3$)$_3$]**[13]	431 (204)	421 (100)	600 (104)	792 (95)	179 / 192

[a] Redox potential *vs.* the FcH/[FcH]$^+$ couple at a glassy carbon working electrode (scan rate 100 mV·s^{-1}), the splitting between the anodic (oxidation) and cathodic (reduction) peaks given in brackets; [b] Determined in an anhydrous 0.1 mol·L^{-1} (nBu$_4$N)BF$_4$/CH$_2$Cl$_2$ solution; [c] Determined in an anhydrous 0.1 mol·L^{-1} (nBu$_4$N)BAr$^F{}_4$/CH$_2$Cl$_2$ (BAr$^F{}_4$ = [B{3,5-(CF$_3$)$_2$C$_6$H$_3$}$_4$]) solution; [d] Peak-to-peak separation of the first and second/second and third redox process in (nBu$_4$N)BAr$^F{}_4$/CH$_2$Cl$_2$; [e] Not fully reversible; additional, scan rate-dependent reduction peak – as a result of prior oxidation – at 98 mV.

In engaging the lone pairs of electrones of the phosphorus atoms in **1a**–**c** in a bond to BH_3, the electrochemistry of the tris(ferrocenyl)arene core becomes more recognisable again (Fig. S11). In the BF_4^--based SE (Fig. S11, left), much like for the bromine-substituted precursors **4a**–**c**, all ferrocenyl groups are oxidised (and reduced) at the same potential with no sign of the mono- or dicationic intermediates. Again, the trifluorobenzene core of **[1b(BH$_3$)$_3$]** (green) gives rise to a cathodically shifted reduction tied to the previous oxidation (dashed *vs.* solid line), speaking for an EC mechanism to occur. In the $BAr^F{}_4^-$-based SE (Fig. S11, left), three well-separated redox events without apparent chemical interference can be recorded. The peak-to-peak separations $\Delta E°_{1/2}$ and $\Delta E°_{2/3}$ (Table S11) follow a different trend than those for **4a**–**c** in that for **[1a(BH$_3$)$_3$]** and **[1b(BH$_3$)$_3$]**, the generation of the dication is more difficult than that of the trication ($\Delta E°_{1/2} > \Delta E°_{2/3}$), while the opposite is true for **[1c(BH$_3$)$_3$]**.

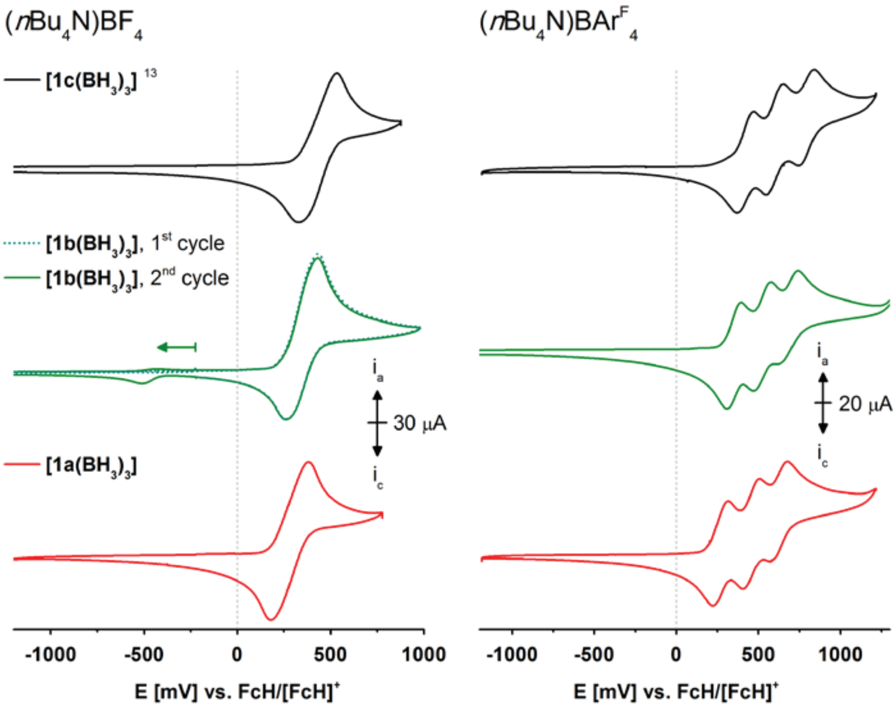

Fig. S11 Cyclic voltammograms of tris(1-diphenylphosphanylborane-1'-ferrocenylene)arenes **[1(BH$_3$)$_3$]** in the BF_4^-- (left) and $BAr^F{}_4^-$-based (right) supporting electrolytes, ordered from lowest to highest oxidation potential. For all voltammograms, the second of three consecutively measured cycles is shown if not indicated otherwise. The scan rate was set to 100 mV·s^{-1}, and scans have been carried out in anodic direction if not indicated otherwise by the arrow. The cyclic voltammograms of **[1c(BH$_3$)$_3$]** have been reported before and are included here for completeness.[13]

Table S12 Formal redox potentials of the tris(1-diphenylphosphanyl-1'-ferrocenylene)arene gold(I) complexes **[1(Au)$_3$]** in two different supporting electrolytes determined by cyclic voltammetry, sorted from lowest to highest redox potential.[a]

Electrolyte	(nBu$_4$N)BF$_4$/CH$_2$Cl$_2$		(nBu$_4$N)BAr$^F{}_4$/CH$_2$Cl$_2$			
**Potentials [mV]**	**E° (ΔE$_p$)**[b]	**E$^{ox}_{fl}$ / E$^{red}_{fl}$**[c]	**E°$_1$ (ΔE$_p$)**[d]	**E°$_2$ (ΔE$_p$)**[d]	**E°$_3$ (ΔE$_p$)**[d]	**ΔE°$_{1/2}$ / ΔE°$_{2/3}$**[e]
[C$_6$H$_3${fcPPh$_2$(AuCl)}$_3$] **[1a(Au)$_3$]**	339 (176)	−313 (br) / −529	376 (101)	545 (106)	704 (110)	170 / 159
[C$_6$F$_3${fcPPh$_2$(AuCl)}$_3$] **[1b(Au)$_3$]**	405 (186)	−433 (br) / −528	419 (93)	580 (106)	760 (98)	160 / 180
[C$_3$N$_3${fcPPh$_2$(AuCl)}$_3$] **[1c(Au)$_3$]**	498 (179)	−408 (br) / −520	535 (108)	688 (111)	888 (116)[f]	153 / 200

[a] Redox potential *vs.* the FcH/[FcH]$^+$ couple at a glassy carbon working electrode (scan rate 100 mV·s^{-1}), the splitting between the anodic (oxidation) and cathodic (reduction) peaks given in brackets; [b] Determined in an anhydrous 0.1 mol·L^{-1} (nBu$_4$N)BF$_4$/CH$_2$Cl$_2$ solution; [c] Peak potentials of a second, cathodically shifted reduction and a concomitant oxidation event observed after a first redox cycle of the analyte in anhydrous 0.1 mol·L^{-1} (nBu$_4$N)BF$_4$/CH$_2$Cl$_2$ solution; [d] Determined in an anhydrous 0.1 mol·L^{-1} (nBu$_4$N)BAr$^F{}_4$/CH$_2$Cl$_2$ (BAr$^F{}_4$ = [B{3,5-(CF$_3$)$_2$C$_6$H$_3$}$_4$]) solution; [e] Peak-to-peak separation of the first and second/second and third redox process in (nBu$_4$N)BAr$^F{}_4$/CH$_2$Cl$_2$; [f] Not fully reversible; measurements with different scan speed reveal this oxidation event to be less reversible at lower (20 mV·s^{-1}) and more reversible at higher (200 mV·s^{-1}) scan speeds.

Comparing **[1a(Au)$_3$]** to mono-gold complex **7**, their redox potentials E^0 (Table S12) are very similar (E^0(**7**) = 320 mV (*vs.* FcH/[FcH]$^+$, recorded in (nBu$_4$N)PF$_6$/CH$_2$Cl$_2$)),[60] although care has to be taken given the broad waves of **[1a(Au)$_3$]** and the slight difference in the supporting electrolyte.

For the C$_3$N$_3$-based tris-chloridogold(I) complex **[1c(Au)$_3$]** in the BAr$^F{}_4^-$-based SE (Fig. S12, right), broad, unstructured reduction events appear after the third oxidation (black solid line) has been cycled through. The first (grey dotted line) and second oxidation (dark grey dashed line) processes, when cycled through individually, do not lead to such behaviour as markedly, even though some weak cathodic currents can be detected starting at about −380 mV *vs.* the FcH/[FcH]$^+$ couple. It can thus be speculated that chemical reactions as part of an EC process transfer a part of the oxidised complex into species which are considerably more difficult to reduce. Such behaviour has already been documented for a mono-gold(I) complex of **1c** and might thus relate to the

s-triazine core itself,[13] as the C_6H_3- and C_6F_3-based complexes do not feature these reduction events under these conditions, while in the BF_4^--based SE (Fig. S12, left), all three compounds show very similar behaviour.

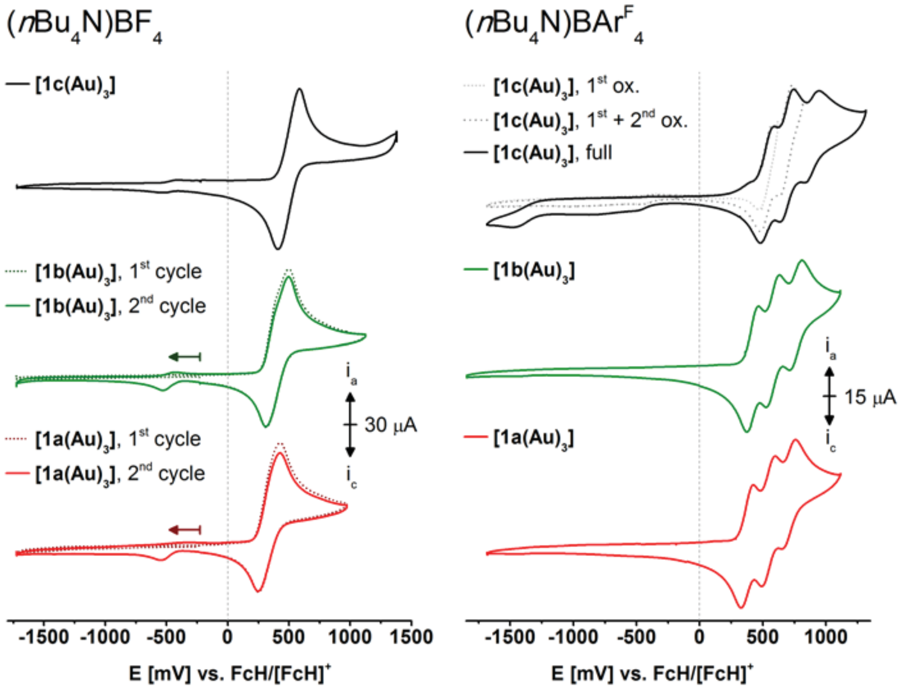

Fig. S12 Cyclic voltammograms of tris(1-diphenylphosphanyl-1'-ferrocenylene)arene gold(I) complexes **[1(Au)₃]** in the BF_4^-- (left) and $BAr^F{}_4^-$-based (right) supporting electrolytes, ordered from lowest to highest oxidation potential. For all voltammograms, the second of three consecutively measured cycles is shown if not indicated otherwise. The scan rate was set to 100 mV·s⁻¹, and scans have been carried out in anodic direction if not indicated otherwise by the arrows, signifying starting potential and initial cathodic scan direction.

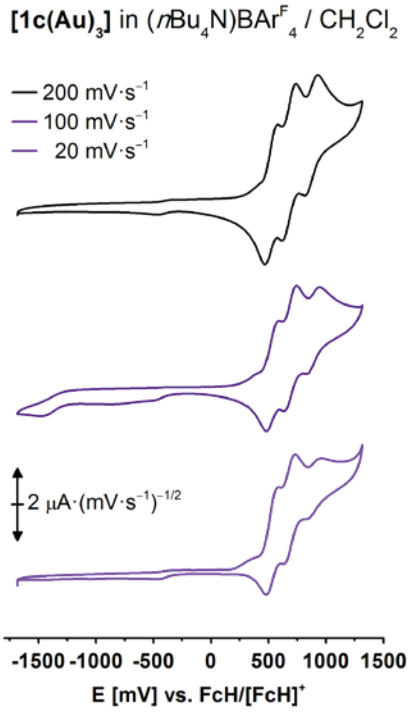

Fig. S13 Oxidation of **[1c(Au)₃]** in (nBu$_4$N)BAr$^F{}_4$/CH_2Cl_2 at and normalised for different scanning speeds (Randles-Sevčik plot), showing the decreasing degree of reversibility of the third oxidation at slower scanning. In all cases, the second of three consecutively measured cycles are shown

Aiming to study this behaviour in more detail, the cyclic voltammograms for **[1c(Au)₃]** have been recorded at three different scan speeds (20, 100, and 200 mV·s⁻¹). As can be seen from the corresponding Randles-Sevčik plot (Fig. S13),[61] the third oxidation becomes more reversible at higher and less reversible at lower scan speeds, supporting the notion of an EC mechanism being the underlying reason for the non-reversibility of the third oxidation. Further proof can be seen in the intensity reduction of the cathodically shifted reduction events for both 20 and 200 mV·s⁻¹. In the former case, the chemically generated species will either diffuse away or might be too unstable to be available for reduction, while in the latter case no sufficient time for its formation is available.

SUPPORTING INFORMATION

4. DFT Calculations

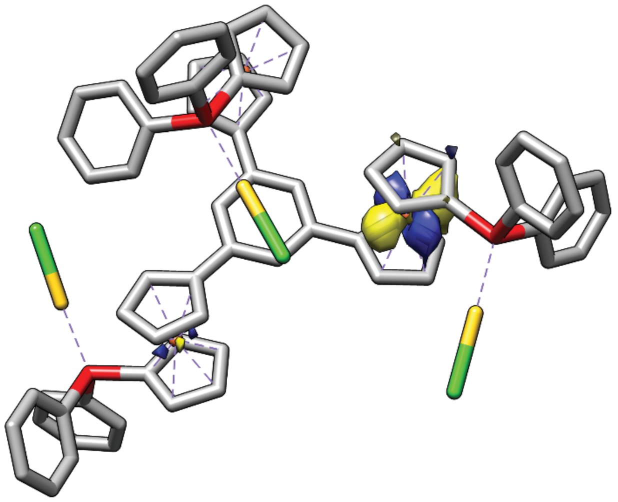

Fig. S14 Fe-centred HOMO of **[1a(Au)$_3$]** at −4.7 eV (isosurface value set to 0.02).

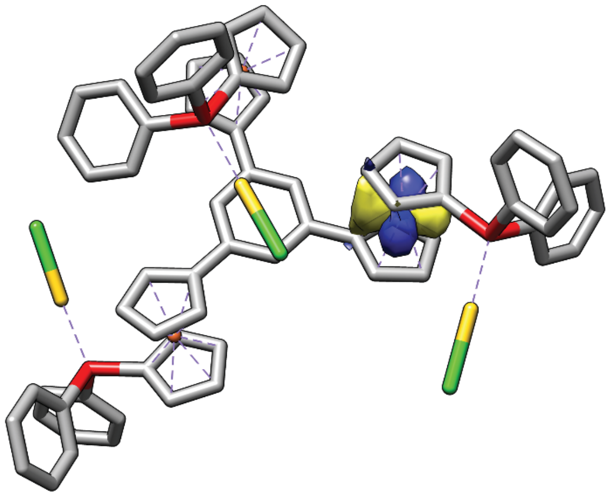

Fig. S15 Fe-centred HOMO−1 of **[1a(Au)$_3$]** at −4.6 eV (isosurface value set to 0.02).

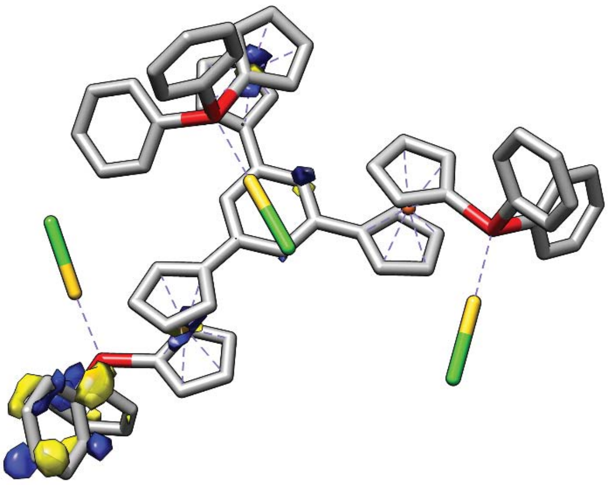

Fig. S16 Fe- and phenyl-centred LUMO of **[1a(Au)$_3$]** at −2.3 eV (isosurface value set to 0.02).

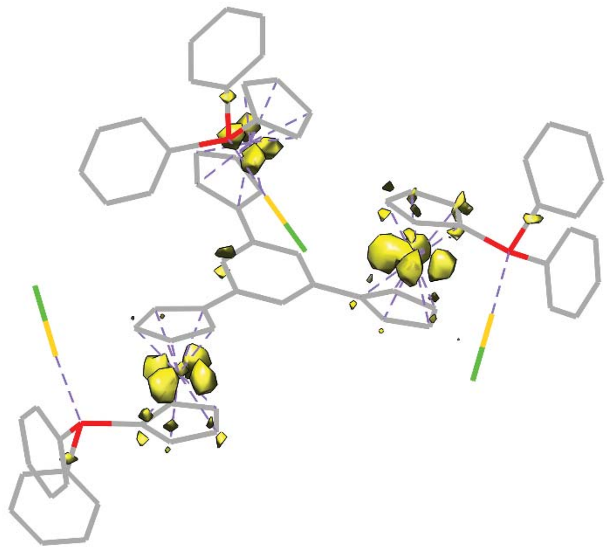

Fig. S17 Difference-density plot highlighting the mostly Fe-centred orbitals involved in the monooxidation of **[1a(Au)$_3$]** to yield **[1a(Au)$_3$]$^+$** (isosurface value set to 0.02).

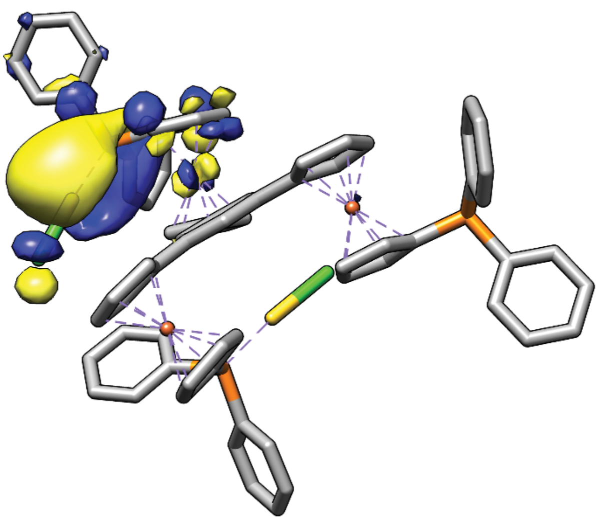

Fig. S18 Unoccupied orbital of native **[1a(Au)$_3$]** with significant Au contribution of about 20% (#380, LUMO+21) at −0.9 eV (isosurface value set to 0.02).

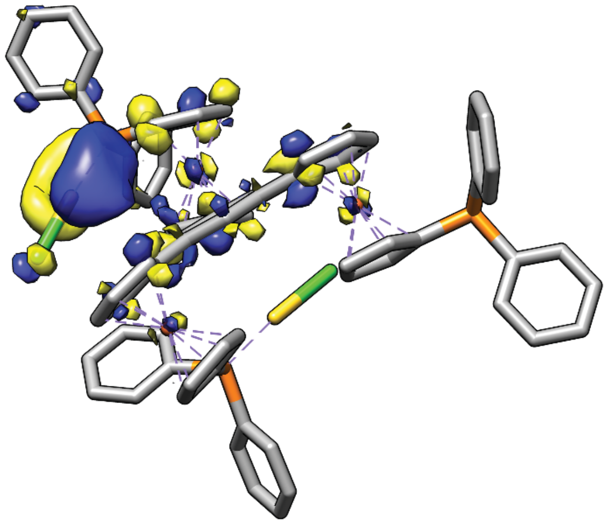

Fig. S19 Unoccupied orbital of mono-oxidised **[1a(Au)$_3$]$^+$** with significant Au contribution of about 20% (#383, LUMO+24) at −2.7 eV (isosurface value set to 0.02).

DFT calculations have been carried out to provide a better understanding of the electrochemical behaviour of **[1a(Au)$_3$]**. The highest occupied molecular orbitals (HOMO, HOMO−1) are mostly Fe-centred (Fig. S14 and S15), while the lowest unoccupied molecular orbital (LUMO) encompasses contributions from iron and the *P*-bound phenyl rings (Fig. S16). This is in line with the (first) oxidation taking place at the ferrocenylene moieties as found by cyclic voltammetry (cf. section 4). Further supporting this notion, a difference-density plot between native **[1a(Au)$_3$]** and a monooxidised species **[1a(Au)$_3$]$^+$** (removing one electron from **[1a(Au)$_3$]**; no changes in geometry, solvent or anion influences have been considered) shows the iron atoms to be involved most strongly (Fig. S17).

As to the presumed increase in electrophilicity of the gold(I) centres upon oxidation of the ferrocenylene groups, unoccupied orbitals with significant gold contribution (Fig. S18 and S19) were found to decrease in energy going from **[1a(Au)$_3$]** to **[1a(Au)$_3$]$^+$**. In the neutral parent complex, these orbitals are located between −0.9 eV and −0.4 eV, while similar orbitals in **[1a(Au)$_3$]$^+$** are found with energies between −2.7 eV and −2.3 eV for both the alpha and beta orbitals of the unrestricted DFT wavefunction.[62]

5. Catalysis

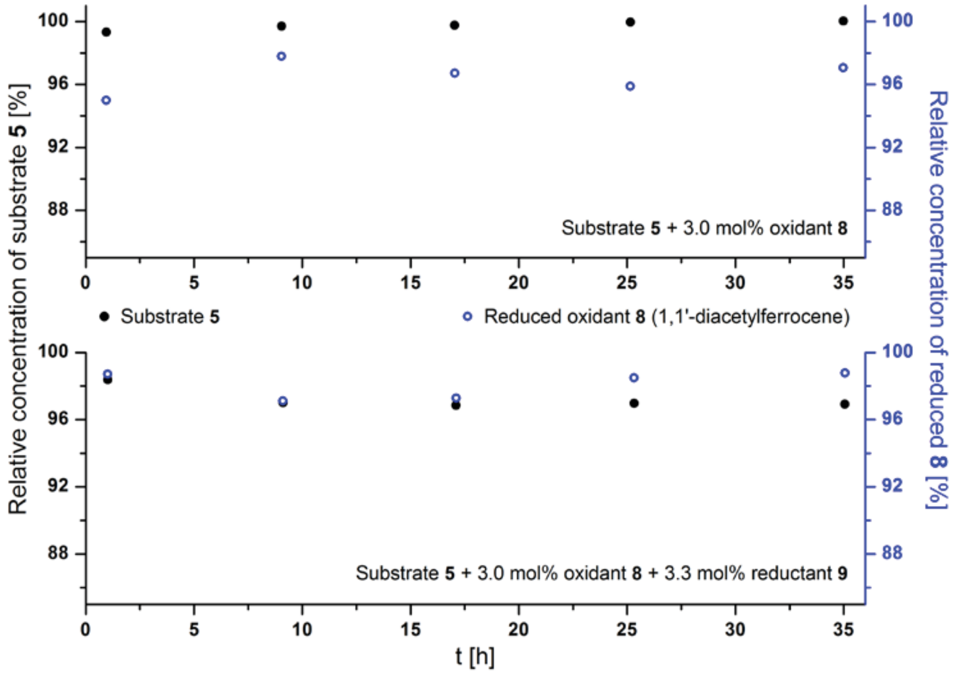

Fig. S20 Relative concentration-over-time graphs for the addition of 3.0 mol% oxidant **8** (top) and for the addition of 3.0 mol% oxidant **8** and 3.3 mol% reductant **9** (bottom) to the stock solution of substrate **5** ([**5**]$_0$ = 60 mmol·L^{-1}, CD_2Cl_2, r.t.; concentration determined *vs.* internal standard 1,3,5-trimethoxybenzene for protons H$_o$ of **5** [black dots] and for ferrocenylene protons of reduced oxidant **8** [hollow blue circles]).

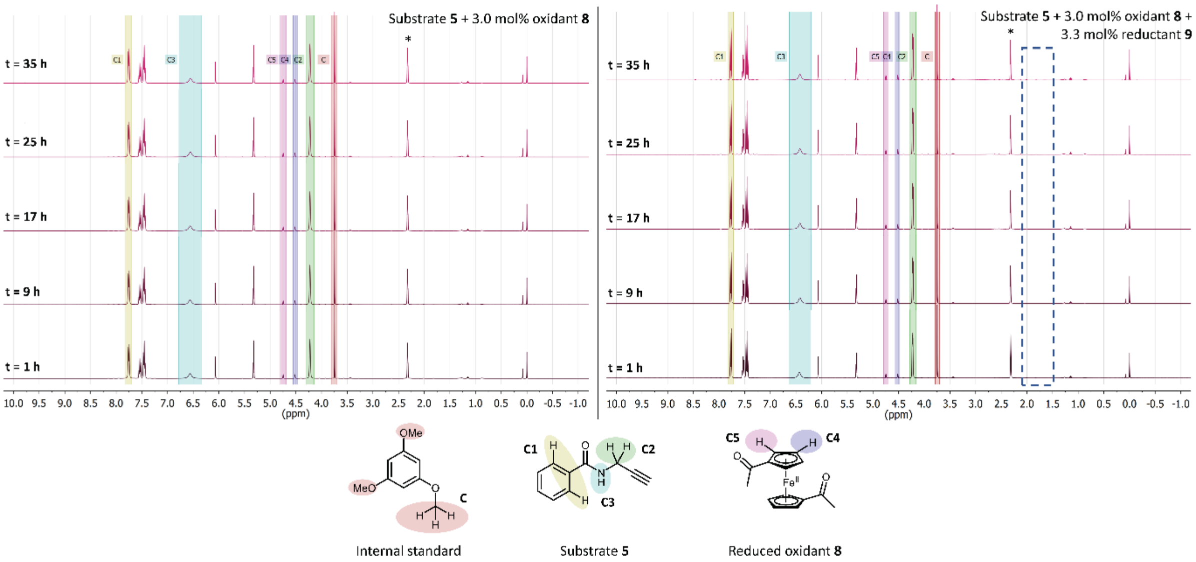

Fig. S21 Stacked ^{1}H NMR spectra, forming the basis for the concentration-over-time graphs in Fig. S20 ([**5**]$_0$ = 60 mmol·L^{-1}, CD_2Cl_2, r.t). The colour-coded areas represent the integrals for determining the reactant concentrations for starting material **5** (yellow, light blue, green) and reduced oxidant **8** (1,1'-diacetylferrocene; pink, dark blue) with respect to internal standard 1,3,5-trimethoxybenzene (red). Time t = 0 has been set as the addition of 3.0 mol% of **8** (left) / 3.0 mol% of **8** and 3.3 mol% of **9** (right) to the substrate stock solution. The asterisk denotes the overlapping resonance of the methine protons of **5** and the methyl protons of reduced **8**. The dashed box marks the spectral region where the methyl protons of unreacted reductant **9** were to be seen.

In order to test for catalytic activity of oxidant **8** and the reaction mixture of **8** and reductant **9** themselves, 3.0 mol% of **8** and both 3.0 mol% of **8** and 3.3 mol% of **9** (with respect to substrate **5**, representing the maximum amounts used throughout this study) were separately added to an appropriately diluted portion (0.6 mL, diluted with pure CD_2Cl_2) of the substrate stock solution (0.5 mL) as 60 µL solutions under argon. Upon introduction of just **8**, a discolouration from green to pink took place; correspondingly, peaks for reduced **8**, *i.e.* 1,1'-diacetylferrocene, are present in the ^{1}H NMR spectra (Figure S21, left), amounting to almost 100% of the maximum concentration of 1.8 mmol·L^{-1}. While it is unclear what caused the reduction of **8**, neither **8** nor 1,1'-diacetylferrocene or any other reduction side- or byproducts catalyse the ring-closing isomerisation of **5** to **6** as it becomes

apparent from the constant concentration of **5** (Fig. S20, top). When both **8** and **9** are introduced to the substrate stock solution, no product formation or appreciable conversion of substrate **5** takes place, either. Again, the relative concentration of reduced **8** is close to 100% (Fig. S20, bottom). It is worth pointing out that no signal for the methyl protons of native **9** is detectable (expected spectral region represented by dashed blue box in Fig. S21) and that the position of the amide proton (light blue spectral region C3 in Fig. S21) is slightly different for both experiments. Both pure reductant **9**[63] and NaBAr$^F_4$ [64] have been previously tested for their catalytic inactivity in this type of catalysis.

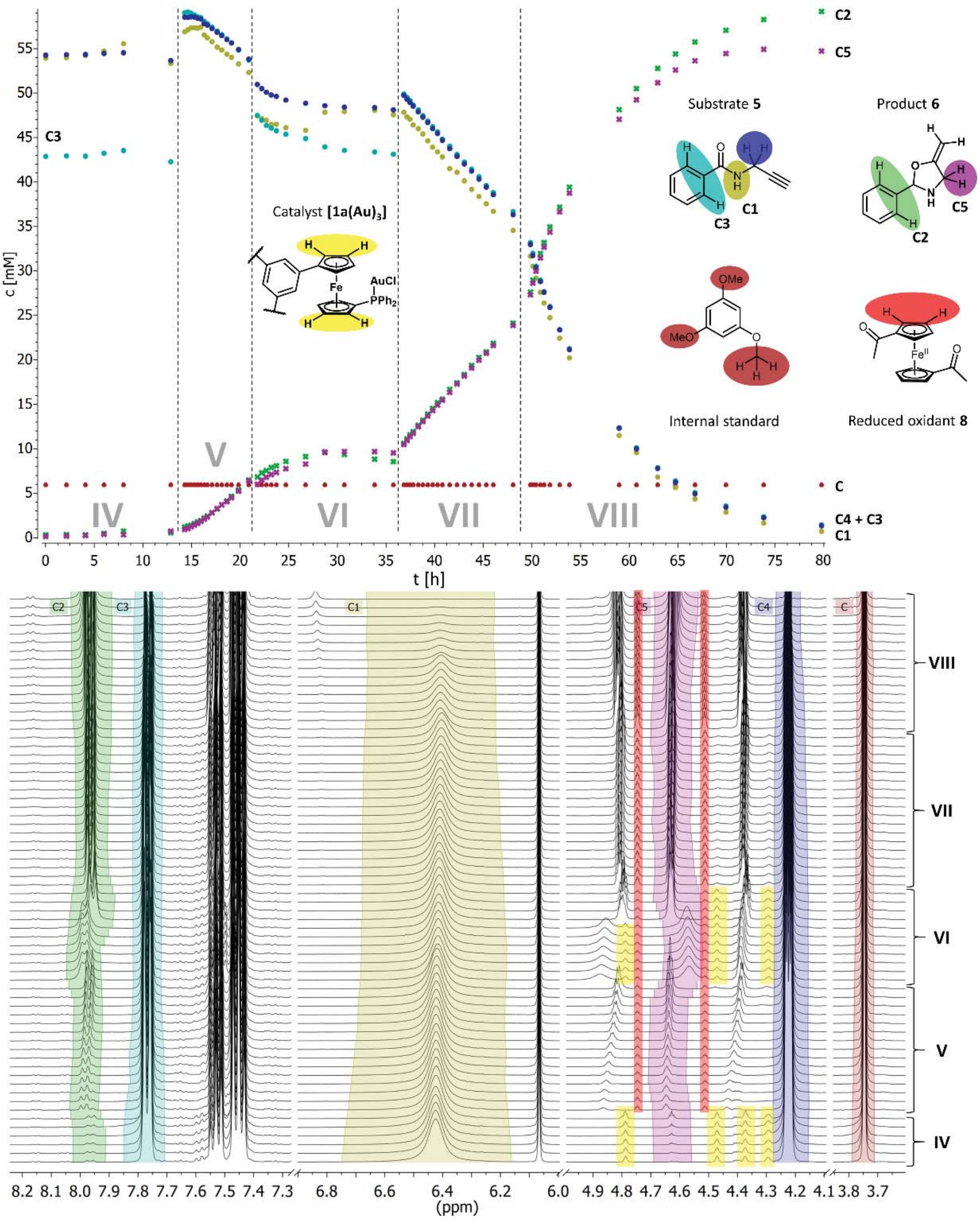

Fig. S22 Top: Concentration-over-time profiles determined for different ^{1}H resonances of substrate **5** (navy, light blue, and golden circles) and product **6** (dark green and lilac crosses) for redox-switched gold(I)-catalysed cyclisation of **5** to **6** (3 mol% Au as **[1a(Au)$_3$]**, [**5**]$_0$ = 60 mmol·L^{-1}, CD_2Cl_2, r.t.), concentrations being determined *vs*. internal standard 1,3,5-trimethoxybenzene (dark red triangles). Phases **IV**–**VIII** refer to Fig. 5, main article, and correspond to native catalyst (**IV**), oxidation with one equivalent of **8** (**V**), reduction with one equivalent of **9** (**VI**), re-oxidation with one equivalent of **8** (**VII**), and subsequent further oxidation with two more equivalents of **8** (**VIII**).

Bottom: Stacked ^{1}H NMR spectra (only regions of interest are shown) forming the basis for the concentration profiles. Spectral regions C1–C5 which have been integrated *vs*. the internal standard C are highlighted in the corresponding colour. ^{1}H resonances attributable to the catalyst during phases **IV** and **VI** are highlighted in yellow, resonances attributable to 1,1'-diacetylferrocene (reduced oxidant **8**) are highlighted in bright red starting from phase **V**.

SUPPORTING INFORMATION

Illustrating, exemplarily, how the catalytic runs were evaluated using ^{1}H NMR spectroscopy and the built-in "data analysis" module of MestReNova, Fig. S22 depicts both the stack of individual ^{1}H NMR spectra (bottom) and the resulting concentration-over-time profiles (top) for the chosen proton resonances. While only the spectral regions of interest are included in this stack, not all of them have been used to follow the reaction. The two multiplets in the aromatic region (7.4–7.6 ppm) correspond to the protons in *meta* and *para* position of the phenyl ring of both substrate **5** and product **6**. The singlet at 6.07 ppm corresponds to the three aromatic protons of the internal standard of which the nine methoxy protons (3.75 ppm) have been chosen for a fixed concentration reference ([Internal standard] ≡ 6.0 mM). The olefinic protons of product **6** (4.38 and 4.81 ppm) have also not been integrated.

In the absence of any paramagnetic species (phase **IV**), the *ortho* phenyl resonances of **5** (light blue circles) give a significantly lower apparent concentration of about 43 mM than deduced from both the amide (golden circles) and the methylene (navy circles) resonances amounting to about 54 mM, despite a quite long delay time d = 10 s being used in the pulse sequence (a standard ^{1}H NMR experiment employs d = 2 s). Upon addition of oxidant **8** (phase **V**), the apparent concentration jumps to about 60 mM, in line with the initial theoretical concentration of $[\mathbf{5}]_0$ = 60 mM. Furthermore, the ferrocenylene resonances of 1,1'-diacetylferrocene (reduced **8**) appear in the ^{1}H NMR spectra (highlighted in bright red). Even though the effective concentration of paramagnetic species is not changed upon addition of reductant **9** (phase **VI**), a sudden drop in apparent concentration occurs for some resonances of both **5** and **6**. Notably, all signals of product **5** become significantly broadened and, presumably paramagnetically, shifted in phase VI, while all signals of substrate **5** remain unaltered in shape and position. Whether this is due to a selective interaction of the decamethylferrocenium cation ($[\mathbf{9}]^+$) with **6** or an interaction between (diamagnetic) **[1a(Au)$_3$]** and **6** is unclear. Gratifyingly, the resonances of **[1a(Au)$_3$]** re-appear after reduction (highlighted in yellow).

The signal broadening and shift of **6** is again reversed when the reaction mixture is re-oxidised with another equivalent of **8** (phase **VII**), again causing a discontinuity in the apparent concentrations back to more "expectable" values, thus suggesting the oxidised catalyst (in this case, **[1a(Au)$_3$]$^+$**) to be a paramagnetic relaxation agent. Further oxidation with two additional equivalents of **8** (phase **VIII**) does not result in another concentration discontinuity but is indeed mirrored in an increase in signal intensity of 1,1'-diacetylferrocene.

Taking everything into account, the *ortho* phenyl protons H_o of **6** (C2, dark green circles) provide the best indicators of the reaction progress when oxidised species are involved, given their separated position, especially from signals of **[1a(Au)$_3$]** and reduced **8**, and comparatively weak discontinuities upon addition of **8** and **9**.

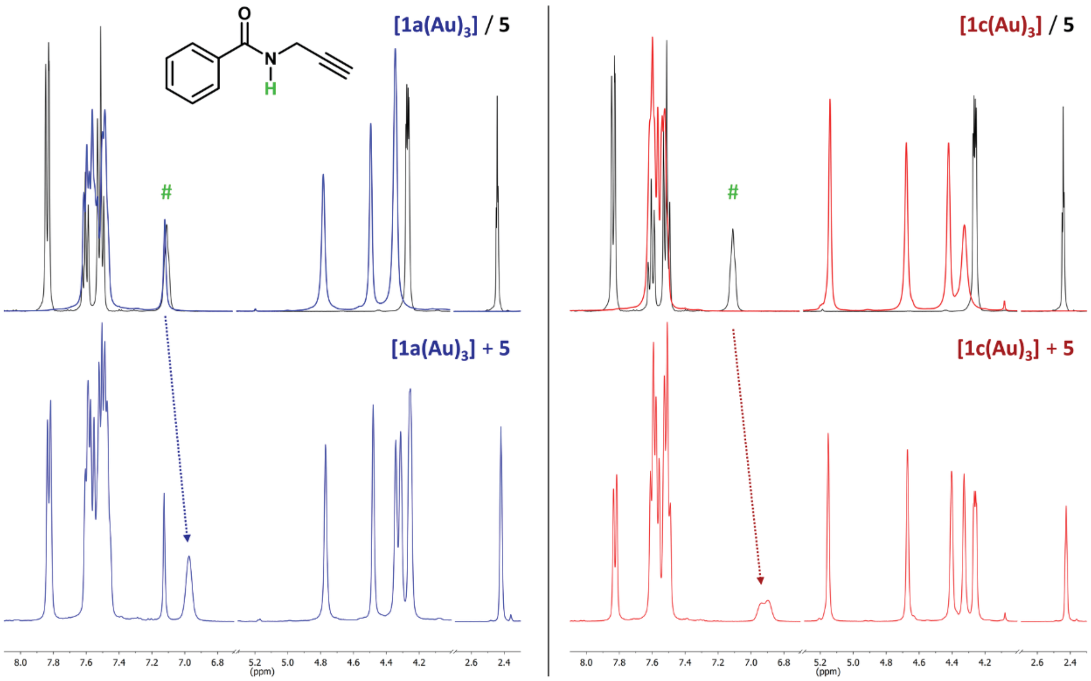

Fig. S23 ^{1}H NMR spectroscopic study of substrate-catalyst interaction (all spectra recorded at −60 °C in CD_2Cl_2, 12 scans, aq = 5 s, d1 = 15 s). Top: Superposition of spectra of **5** (black) and complexes **[1a(Au)$_3$]** (left, blue) and **[1c(Au)$_3$]** (right, red), respectively. Bottom: Spectra of complexes (**[1a(Au)$_3$]** – left, blue / **[1c(Au)$_3$]** – right, red) and **5** in a 1:3 ratio. The hashtag symbol (#) denotes the signal corresponding to the amide N-H proton of **5**.

In order to investigate the unexpected catalytic inactivity of **[1c(Au)$_3$]** towards the conversion of **5** to **6**, the interaction of complex and substrate was studied using ^{1}H NMR spectroscopy at −60 °C (Fig. S23). For this experiment, spectra of both complexes

and the substrate in CD_2Cl_2 were first recorded separately (Fig. S23, top) and superimposed. Following, a Young's NMR tube was charged with a defined amount of **[1a(Au)₃]** or **[1c(Au)₃]** by evaporating a stock solution *in vacuo*. The NMR tube was cooled to −100 °C (ethanol/$N_{2(l)}$). A stock solution of **5** in CD_2Cl_2 was added to the pre-cooled tube, so that a stoichiometric ratio complex-to-substrate of 1:3 was achieved. The NMR tubes containing the frozen mixture were evacuated, closed, and kept at −100 °C prior to the measurement. Each sample was put into the pre-cooled NMR spectrometer (−60 °C) as quickly as possible and left to equilibrate at this temperature for several minutes before locking, shimming, and measuring.

While both **[1a(Au)₃]** and **[1c(Au)₃]** lead to a significant shielding of the amide proton resonance of **5** this very resonance splits into a broad, doublet-like structure only for **[1c(Au)₃]** (Fig. S23, bottom right). The other resonances of **5** are hardly affected by the presence of either **[1a(Au)₃]** or **[1c(Au)₃]**. It is, however, interesting to note that the presence of **5** leads to a splitting (**[1a(Au)₃]**) or narrowing (**[1c(Au)₃]**) of the ferrocenylene resonances associated with the P-substituted C_5H_4 ring in both cases. This observation might hint at desymmetrisation of the trinuclear complexes upon (reversible and fast) coordination of **5** to the gold atoms; however, no changes in the corresponding ^{31}P NMR signals have been observed. As a side observation, cooling to −60 °C does not slow down the rotation about the C_{arene}–C_5H_4 bonds to observe decoalescence.

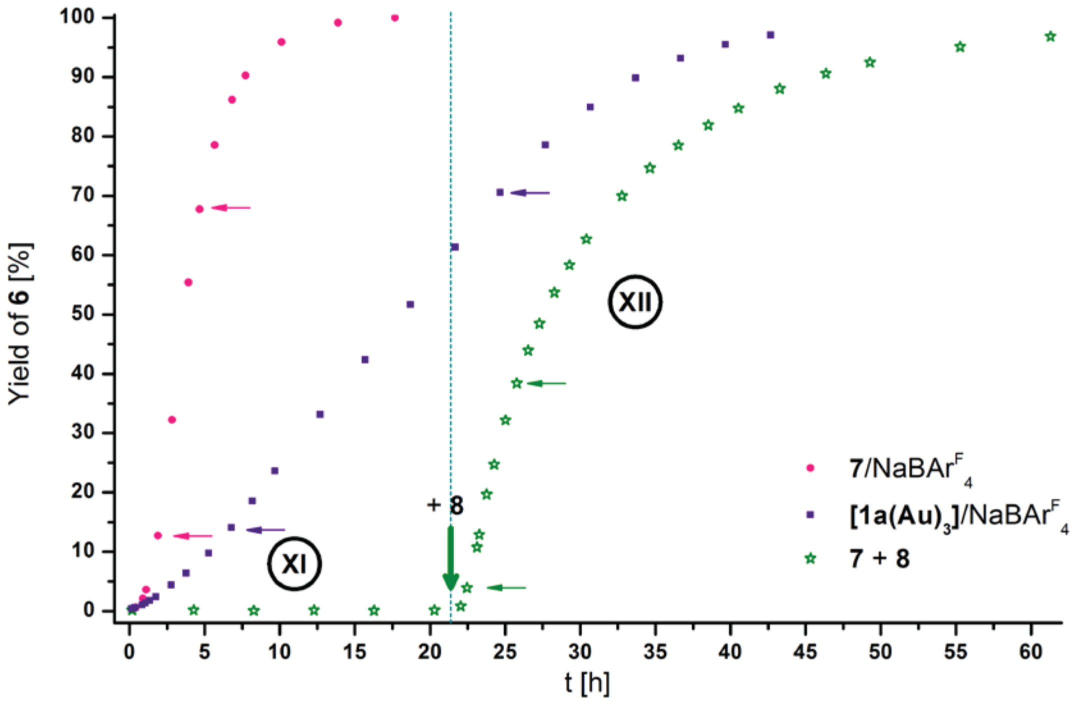

Fig. S24 Yield-over-time graph for gold(I)-catalysed cyclisation of **5** to **6** (3 mol% Au as **[1a(Au)₃]** and **7**, $[\mathbf{5}]_0$ = 60 mmol·L^{-1}, CD_2Cl_2, r.t.; yield determined *vs.* internal standard 1,3,5-trimethoxybenzene for protons H_o of **6** for "**7+8**" [green stars] and for ring-methylene protons of **6** H_m for NaBAr$^F_4$-activated reactions [pink dots, purple squares]). The vertical arrow indicates the addition of **8** for reaction "**7+8**" (green stars); **XI**: before oxidation of **7**, **XII**: after oxidation of **7**. Thin horizontal arrows denote the time region used for linear regression; for corresponding regression parameters a (TOF), b, and R_{corr}^2, cf. Table S13.

As Fig. S24 shows, both **[1a(Au)₃]** (purple squares) and **7** (pink dots) can be activated for the ring-closing isomerisation of propargyl amide **5** to oxazoline **6** by chloride abstraction, using a stoichiometric amount of NaBAr$^F_4$ prior to addition of substrate **5**. In contrast to directly employing native **[1a(Au)₃]** as a catalyst – where this tri-gold complex shows superior performance when compared to mono-gold analogue **7** (cf. Fig. 4, main article) – an anti-cooperative effect can be deduced from the yield-over-time plot and the correspondingly derived linear regressions (Fig. S31 and S32). In fact, **7/NaBAr$^F_4$** operates with a 6.4-fold higher TOF of 20.1±0.6 h^{-1} *vs.* 3.15±0.01 h^{-1} determined for **[1a(Au)₃]/NaBAr$^F_4$**.

To better understand the inferior performance of **[1a(Au)₃]/NaBAr$^F_4$** when compared to **7/NaBAr$^F_4$**, an NMR experiment was conducted where **[1a(Au)₃]**, in a higher concentration suitable for the acquisition of ^{1}H and ^{31}P{^{1}H} NMR spectra, was treated with a stoichiometric amount of NaBAr$^F_4$ in the absence of both substrate **5** and the internal standard 1,3,5-trimethoxybenzene, otherwise following the same activation protocol. The results of this experiment are shown in Fig. S25. Right after the abstraction process (dark slate blue, middle), almost no signal attributable to native **[1a(Au)₃]** (green, top) can be discerned, and severe line broadening is apparent from both the ^{1}H and the ^{31}P{^{1}H} NMR spectra. The latter indicates the presence of two major species, with the resonance at 42.9 ppm suggesting a cationic, *P,P'*-dicoordinate gold(I) species.[65,66] The sample was left overnight (prepared under inert conditions, sealed under an argon atmosphere and protected from light), and another set of spectra was recorded (bottom, dark purple), clearly showing a change with time, in line with our previous observation of a change in rate

constant over time. Employing the inverse-gated pulse sequence $z_g i_g$ (spectrum not shown), the integrals for the three main phosphorus-containing species were determined to approx. 2:1:2. Apparently, some **[1a(Au)₃]** had re-formed, its ^{1}H NMR signals significantly broadened and falling together into just three signals. A further upfield shift for the broad ^{31}P resonance is associated with a loss of intensity; the extremely broad signals previously observed in the ^{1}H NMR spectrum have almost completely vanished, leaving a much simpler pattern in the spectral region characteristic for ferrocene (4–5 ppm), the four major resonances suggesting a highly (C_3-)symmetric species. At this point, we can but speculate about the nature of the catalytically active species in solution. Despite the cationic dicoordinate gold(I) species being an obvious candidate, similar cationic, *P*,*P*'-dicoordinate gold(I) complexes that we have tested in the same transformation did – by far – perform worse; furthermore, the formation of a very similar species upon reduction of **[1a(Au)$_3$]$^{3+}$** (*vide infra*) casts further doubt on this hypothesis, leaving the species linked to the broadened resonance at about 20 ppm as a likely suspect. Yet, the presence of substrate might yield a different picture altogether.

In addition, the oxidation-induced activation of **7** using one equivalent of oxidant **8** was investigated, and the obtained results are also depicted in Fig. S24 (green stars). In its native state at 1 mol% Au catalyst loading, **7** displays no appreciable catalytic activity, unsurprising given its lack of activity at 3 mol% Au loading. Upon addition of **8**, notably without any discernible activation period in contrast to the oxidation-induced activation of **[1a(Au)$_3$]**, a sharp increase of catalytic activity can be witnessed. The TOF of **[7]$^+$** was determined to 10.6±0.4 h^{-1}, that is, higher than *in situ* generated **[1a(Au)$_3$]$^{3+}$**, isolated **[1a(Au)$_3$](TEF)$_3$** or **[1a(Au)$_3$]/NaBAr$^F_4$**, yet significantly smaller than for **7/NaBAr$^F_4$**. It can thus be concluded that the chloride abstraction provides a better means of activation for **7** than the oxidation, yet without the added benefit of comparatively easy reversibility. That is even more so to case for **[1a(Au)$_3$]** where (threefold) oxidation and chloride abstraction yield similarly active species, with the former allowing for mutual transformation of activity states into one another.

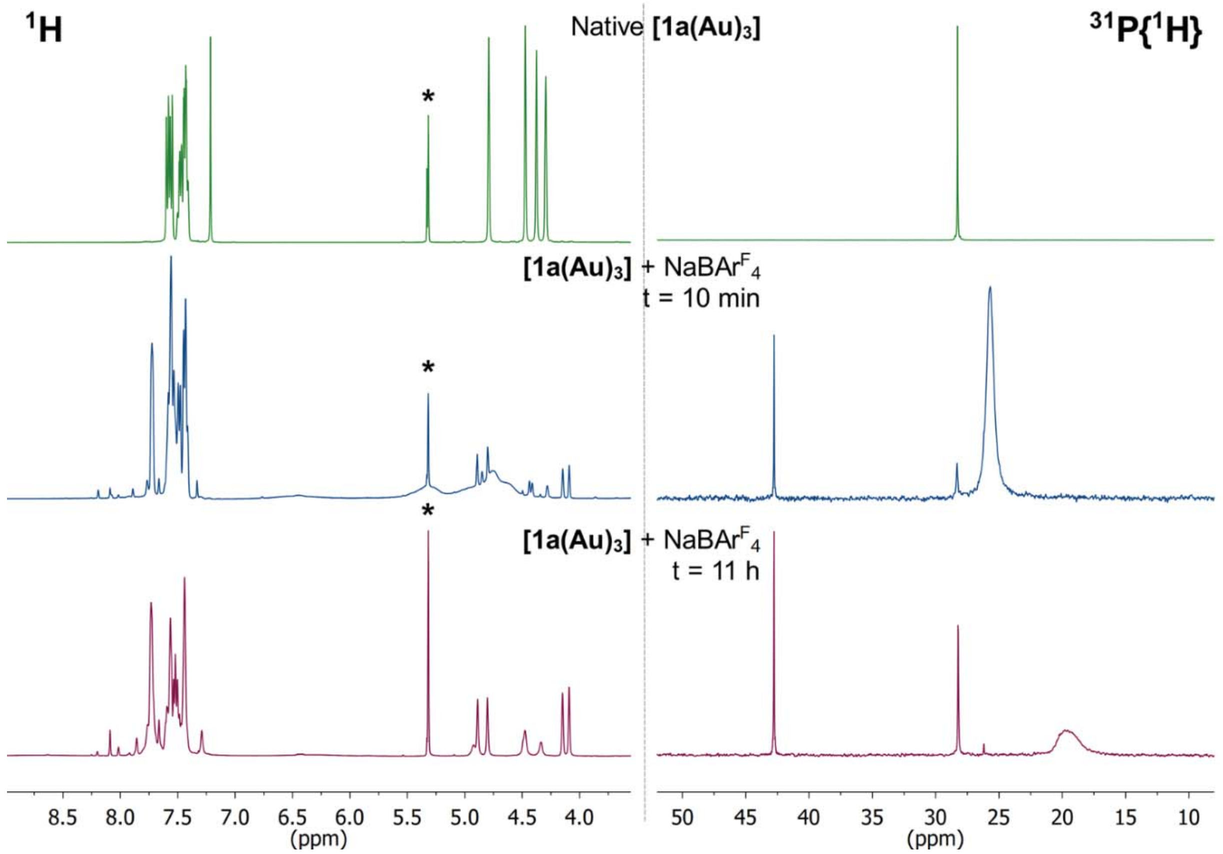

Fig. S25 Stacked ^{1}H (left) and ^{31}P{^{1}H} NMR (right) spectra acquired before (top, dark green), right after (middle, dark slate blue), and long after (bottom, dark purple) the halide abstraction using 1.0 eq. of NaBAr$^F_4$. Spectra have been acquired in CD_2Cl_2, the corresponding ^{1}H NMR signals for $CHDCl_2$ and CH_2Cl_2 are marked with asterisks (*). Only the relevant spectral region is shown. ^{31}P{^{1}H} NMR spectra after the halide abstraction have been acquired using the $z_g p_g$ pulse sequence for fast-relaxing nuclei and can hence not be integrated properly for concentration determination. For the graphic depiction, an exponential line broadening of 5 Hz has been applied to the ^{31}P{^{1}H} NMR spectra.

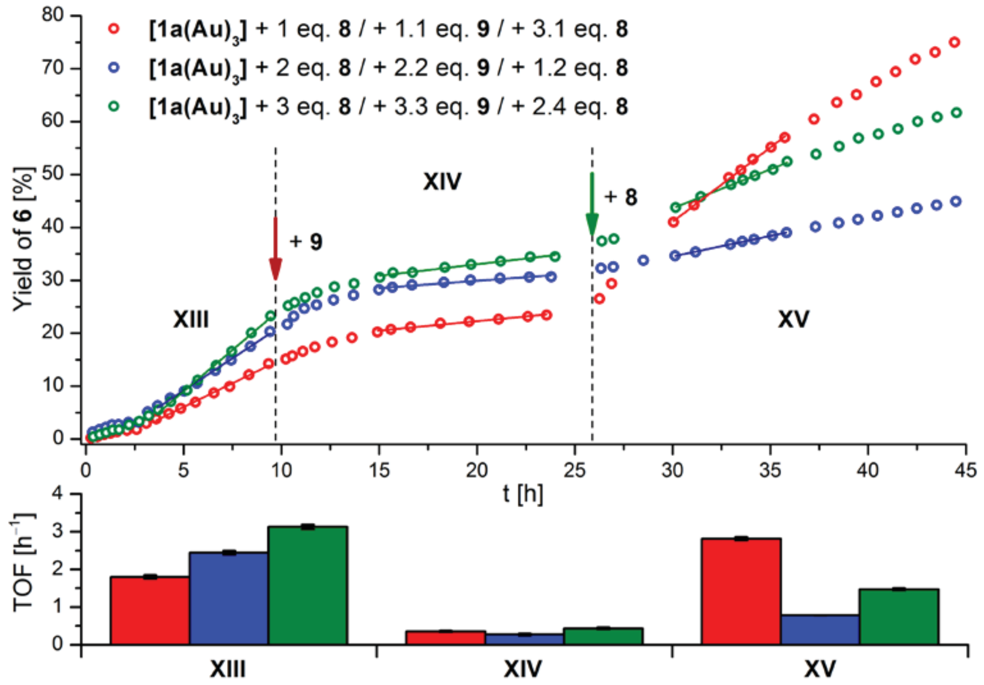

Fig. S26 Top: Yield-over-time graph for gold(I)-catalysed cyclisation of **5** to **6** (1 mol% Au as **[1a(Au)₃]**, $[\mathbf{5}]_0$ = 60 mmol·L^{-1}, CD_2Cl_2, r. t.; yield determined *vs*. internal standard 1,3,5-trimethoxybenzene for protons H_o of **6**). Complex **[1a(Au)₃]** was oxidised by 1 (red), 2 (blue), or 3 (green) equivalents of **8** prior to addition of substrate **5** (t = 0). The vertical arrows indicate the addition of **9** (red arrow) and 3.1 (red), 1.2 (blue), or 2.4 (green) equivalents of **8** (green arrow). Straight lines represent the linear fits during the three different phases: **XIII**: after oxidation; **XIV**: after reduction; **XV** after re-oxidation. For corresponding regression parameters a (TOF), b, and R_{corr}^2, cf. Table S13. Bottom: Turn-over-frequencies (TOF) obtained by linear regression for the three different reaction phases (colour code as above).

In order to test whether the presence of substrate during the oxidation would (negatively) influence the performance of the catalytically active oxidised complex during the conversion of **5** to **6**,[67] the oxidation of **[1a(Au)₃]** with one, two, or three equivalents prior to substrate addition was carried out (Fig. S26, TOF cf. bottom of Fig. S26 and Table S13). Oxidant **8** (20, 40 or 60 µL of a stock solution) and pre-catalyst **[1a(Au)₃]** (0.1 mL of a stock solution) were, under argon, mixed in a Young's NMR tube and left to stand for 10 minutes. Afterwards, the reaction was initiated by addition of the substrate stock solution (0.5 mL) and the reaction was followed by NMR spectroscopy as usual.

In all three cases, the TOF obtained by linear fits of the yield-over-time plots are in good agreement with those obtained by oxidising **[1a(Au)₃]** in the presence of substrate **5** with the exception of the mono-oxidised reaction (red trace in Fig. S26 which is slightly faster than its equivalent 1.8 h^{-1} *vs.* 1.2 h^{-1}). Induction periods are observed, too, and are thus most likely not a direct result of oxidation in the presence of **5**, but more likely resulting from the interaction of oxidised complexes and substrate **5** itself. Furthermore and in agreement with the oxidation in the presence of substrate, addition of reductant **9** leads, after some delay, to a halting of the conversion. A second addition of oxidant, as for the other reactions, did lead to renewed catalytic activity, although the observed activities are somewhat lower than for the initial species, particularly in the case of going from the twofold to the mono-oxidised species (blue trace in Fig. S26).

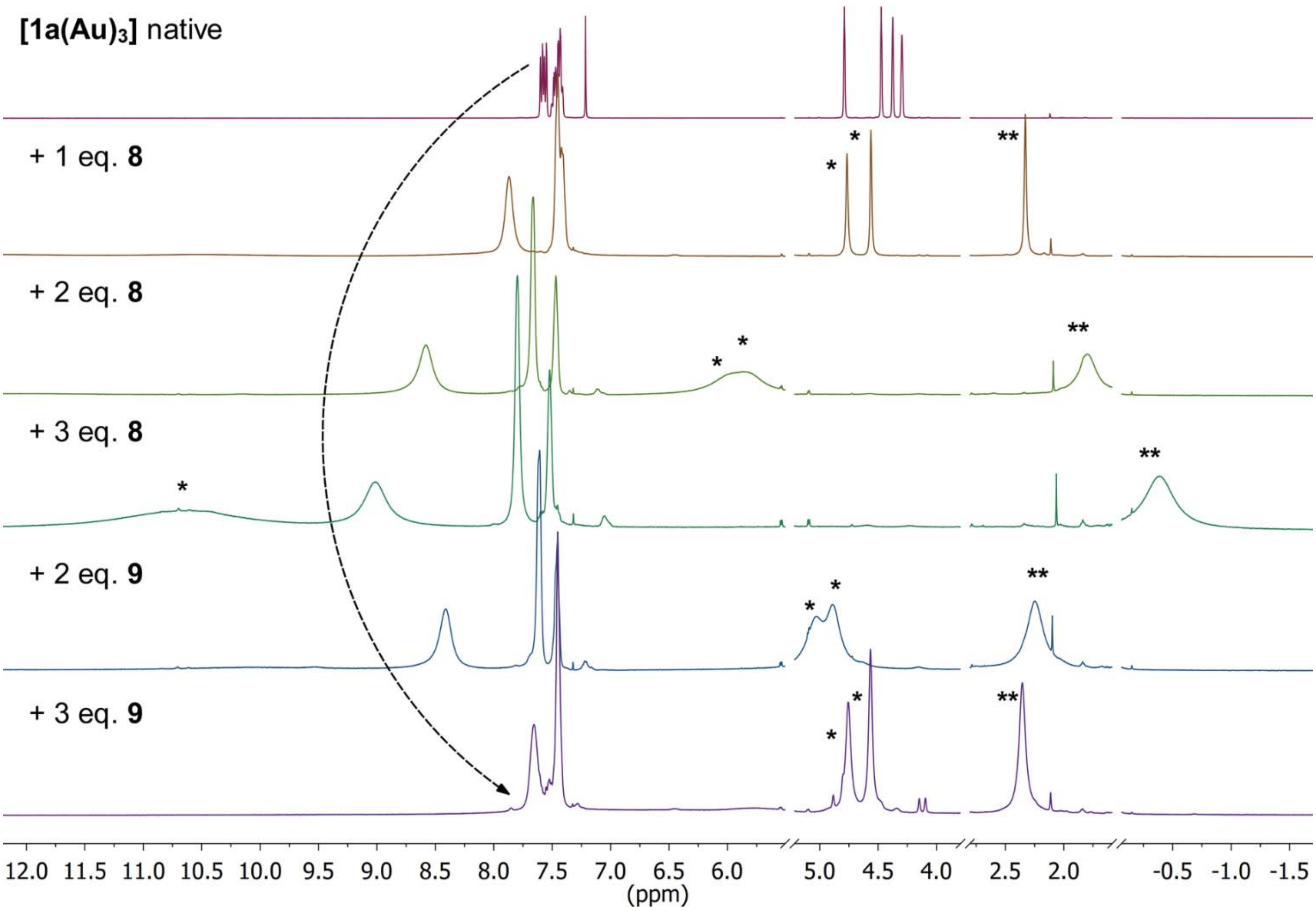

Fig. S27 Stacked, individually scaled ^{1}H NMR spectra before (red, top) and after the successive addition of one (orange), two (green), and three (teal) equivalents of oxidant **8**, followed by the successive addition of two (slate blue) and three (purple, bottom) equivalents of reductant **9**. Only regions of interest are shown, and residual solvent signals have been excised from the spectra as indicated by the broken lines. All spectra belong to the same sample prepared and kept under an argon atmosphere, and have been acquired in CD_2Cl_2. The sample was protected from light and maintained at room temperature in between measurements. Asterisks denote the signal attributable to the ferrocenyl (*) and acetyl (**) resonances of reduced **8** (*i.e.*, 1,1'-diacetylferrocene). The dashed arrow highlights the paramagnetic shift experienced by the phenyl protons of **[1a(Au)₃]** upon oxidation and reduction.

In an attempt to elucidate the behaviour of **[1a(Au)$_3$]** upon stepwise oxidation/reduction using **8**/**9**, a corresponding NMR experiment was conducted (Fig. S45 and S46) in CD_2Cl_2. Upon the addition of the first equivalent of oxidant **8**, the ferrocenylene signals of **[1a(Au)$_3$]** completely disappear from the standard spectral region of ^{1}H NMR spectroscopic measurements (Fig. S45), indicating either a mixed-valent nature of **[1a(Au)$_3$]$^{+}$** or fast intermolecular electron(-hole) transfer on the NMR time scale. Both would lead to, statistically speaking, all ferrocenylene moieties appearing (partly) oxidised on the NMR time scale, thus rendering the ^{1}H signals very broad and shifted to unusual resonance frequencies (cf. ^{1}H NMR shifts of the 1,1'-diacetylferrocenium ion in **8**: δ = 32.5 [$\omega_{1/2}$ = 1400 Hz], 3.95 [$\omega_{1/2}$ = 360 Hz], −10.1 ppm [$\omega_{1/2}$ = 320 Hz]). On the contrary, the ^{1}H NMR signals attributable to the *P*-bound phenyl rings of **[1a(Au)$_3$]$^{+}$** are less afflicted, experiencing minor shifts and broadening given their greater distances to the presumed loci of oxidation (cf. DFT calculations in section 4). However, only one set of resonances is visible, in line with fast electron(-hole) transfer. The signals attributable to 1,1'-diacetylferrocene (reduced **8**, * and ** in Fig. S45) appear at their literature-described chemical shifts.[68] Upon further addition of up to three equivalents of **8**, those signals shift to higher (*) and lower (**) field, indicative of either being strongly affected by the paramagnetic species present in solution or partaking in fast electron-transfer events between different oxidised species. The latter is more likely, given that the oxidation potential of the 1,1'-diacetylferrocenium cation in CH_2Cl_2 (E^0 = 490 mV *vs*. FcH/[FcH]$^{+}$)[69] might not be sufficient for complete two- and threefold oxidation, resulting in equilibria between **[1a(Au)$_3$]$^{2+}$**, **[1a(Au)$_3$]$^{3+}$**, 1,1'-diacetylferrocene and **8**. The ^{1}H NMR signals of the *P*-phenyl groups become deshielded upon further oxidation, experiencing the paramagnetism of the ferrocenylene units in **[1a(Au)$_3$]$^{n+}$** to different degrees. Similarly, the ^{31}P NMR resonance (Fig. S46) of **[1a(Au)$_3$]**, narrow in its uncharged state ($\omega_{1/2}$ = 3.2 Hz), becomes ever more shielded and broadened upon stepwise addition of up to three equivalents of **8** (**[1a(Au)$_3$]$^{n+}$**; n = 1: δ = 12.9 ppm, $\Delta\delta$ = −15.4 ppm, $\omega_{1/2}$ = 220 Hz; n = 2: δ = 6.2 ppm, $\Delta\delta$ = −22.1 ppm, $\omega_{1/2}$ = 325 Hz; n = 3: δ = 3.0 ppm, $\Delta\delta$ =−25.3 ppm, $\omega_{1/2}$ = 1000 Hz). Although the reaction seems to proceed quite selectively, a second species with a ^{31}P NMR chemical shift between 45 and 40 ppm is formed in a small quantity. Upon stepwise addition of up to three equivalents of reductant **9**, the ^{1}H

NMR spectral features characteristic of 1,1'-diacetylferrocene are recovered (Fig. S45), while those of the *P*-bound phenyl ring protons of **[1a(Au)₃]** remain broadened even after a prolonged waiting time which had been chosen to understand why the addition of **9** in the RSC experiments resulted in a delayed attenuation of catalytic activity. The ^{1}H NMR signals of the ferrocenylene moiety might re-appear but are hard to discern buried beneath the signals of 1,1'-diacetylferrocene. Similarly, although the addition of **9** also leads to a reversal of the upfield shift for the ^{31}P NMR resonance of **[1a(Au)$_3$]$^{n+}$**, no full but only partial recovery of **[1a(Au)$_3$]** can be observed, accompanied by the presence of a probably *P*,*P'*-dicoordinate gold(I) species (δ = 42.8 ppm, *vide supra*)[65,66] indicative of chloride loss and a broadened signal at δ = 18.6 ppm, maybe linked to incomplete reduction and electron-transfer equilibria.

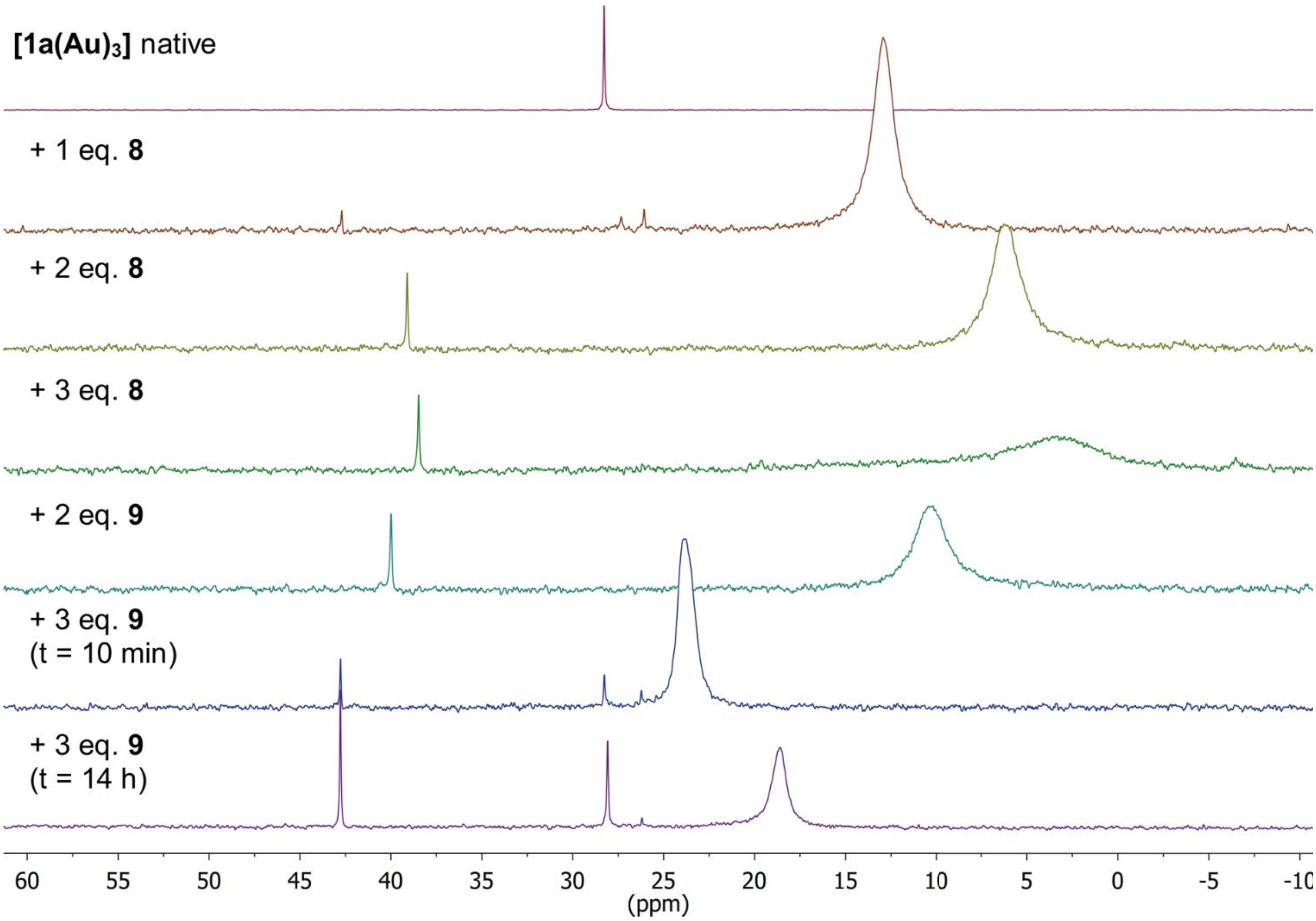

Fig. S28 Stacked, individually scaled $^{31}P\{^{1}H\}$ NMR spectra before (red, top) and after the successive addition of one (orange), two (mustard), and three (green) equivalents of oxidant **8**, followed by the successive addition of two (light blue) and three (navy) equivalents of reductant **9** as well as 14 h after the addition of **9** (purple, bottom). All spectra belong to the same sample prepared and kept under an argon atmosphere, and have been acquired in CD_2Cl_2 using the z_gp_g pulse sequence for fast-relaxing nuclei for all spectra except for native **[1a(Au)$_3$]** (top). They can hence not be integrated properly for concentration determination. For the graphic depiction, an exponential line broadening of 10 Hz was applied. The sample was protected from light and maintained at room temperature in between measurements.

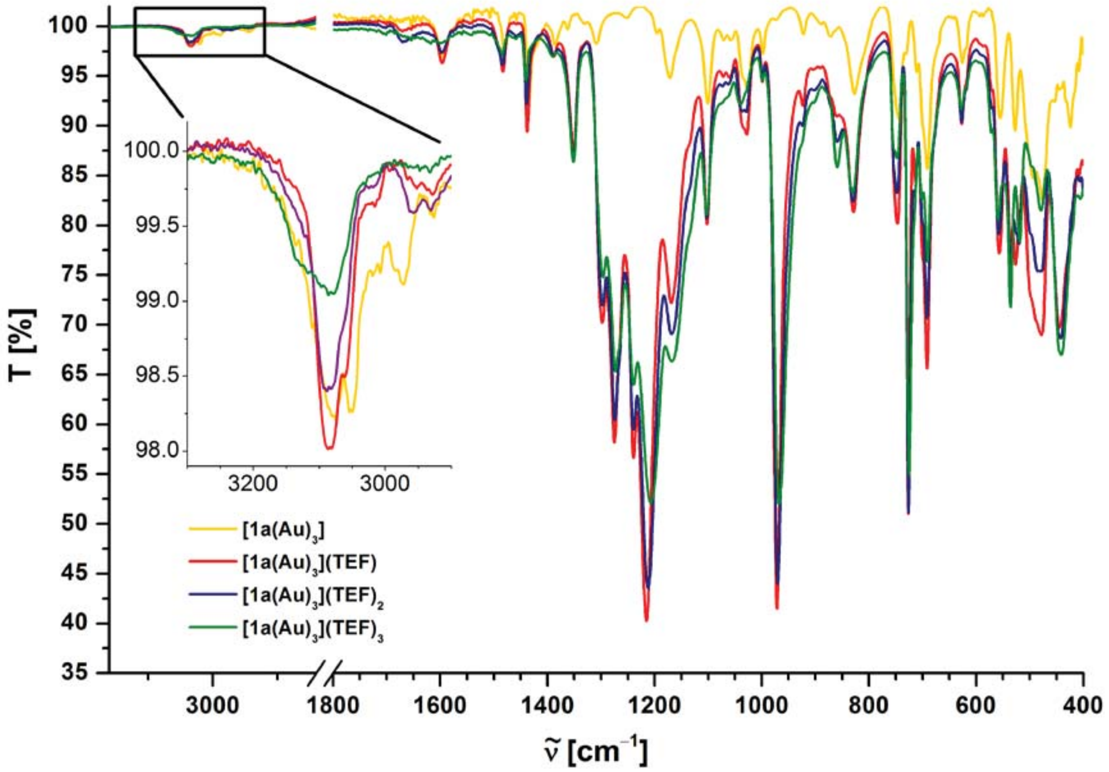

Fig. S29 Superposition of IR spectra (obtained by ATR IR spectroscopy of solid samples) of native **[1a(Au)$_3$]** (yellow) and oxidised complexes **[1a(Au)$_3$](TEF)$_n$** (n = 1 red, n = 2 blue, n = 3 green). The insert shows the magnified region containing the ν(C–H) stretching bands. The spectral region between 2800 and 1800 cm^{-1} does not contain identifiable signals.

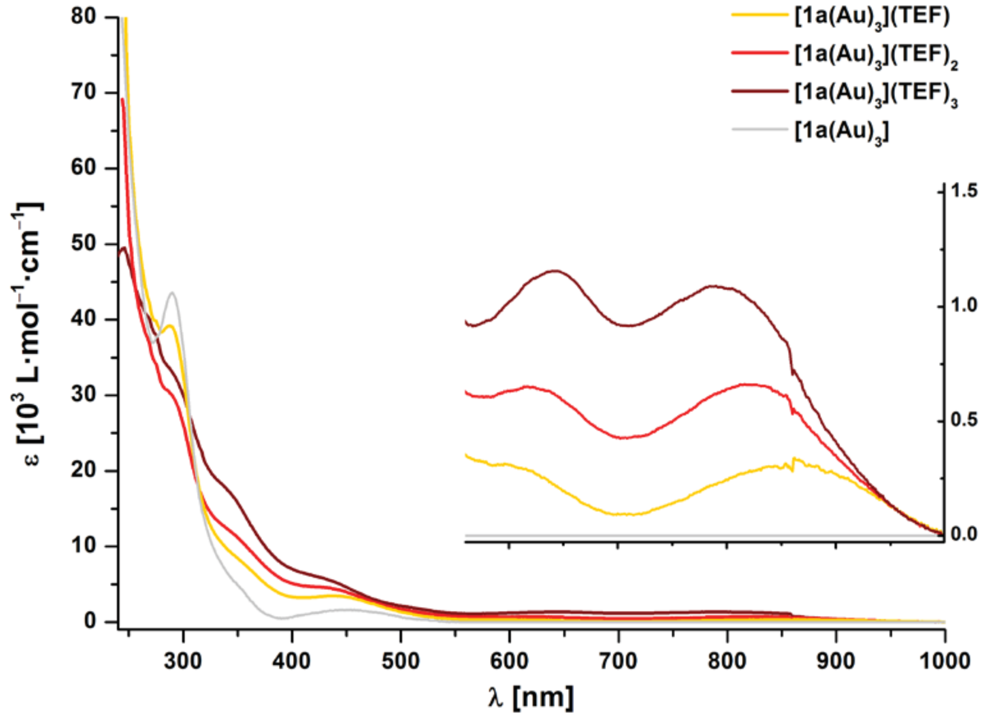

Fig. S30 Superposition of UV/Vis spectra of native **[1a(Au)$_3$]** (grey) and oxidised complexes **[1a(Au)$_3$](TEF)$_n$** (n = 1 yellow, n = 2 red, n = 3 brown) in CH_2Cl_2. The insert shows the magnified spectral region containing absorptions at long wavelength associated with the ferrocenium moieties.

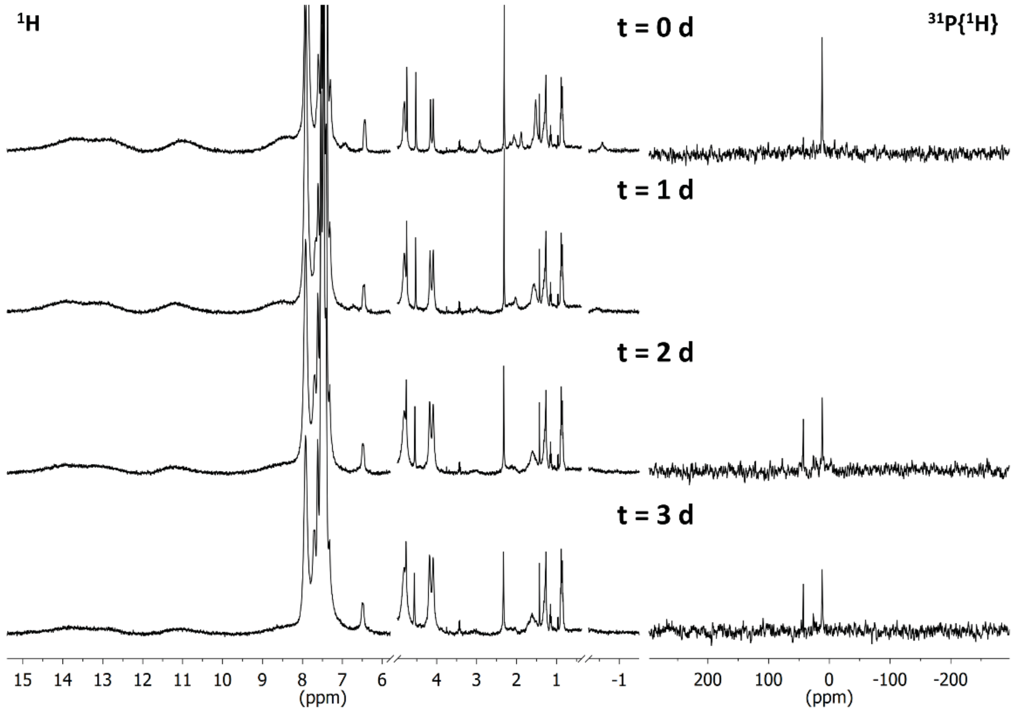

Fig. S31 Stacked ^{1}H (left) and ^{31}P{^{1}H} NMR spectra of **[1a(Au)$_3$](TEF)** in CD_2Cl_2 measured direct after sample preparation (top) and after one, two, and three days. On day one after sample preparation, no ^{31}P{^{1}H} NMR spectrum was recorded. For clarity, the signals attributable to $CHDCl_2$/CH_2Cl_2 and TMS have been cut out from the ^{1}H NMR spectra. ^{31}P{^{1}H} NMR spectra are shown with 100 Hz exponential line broadening.

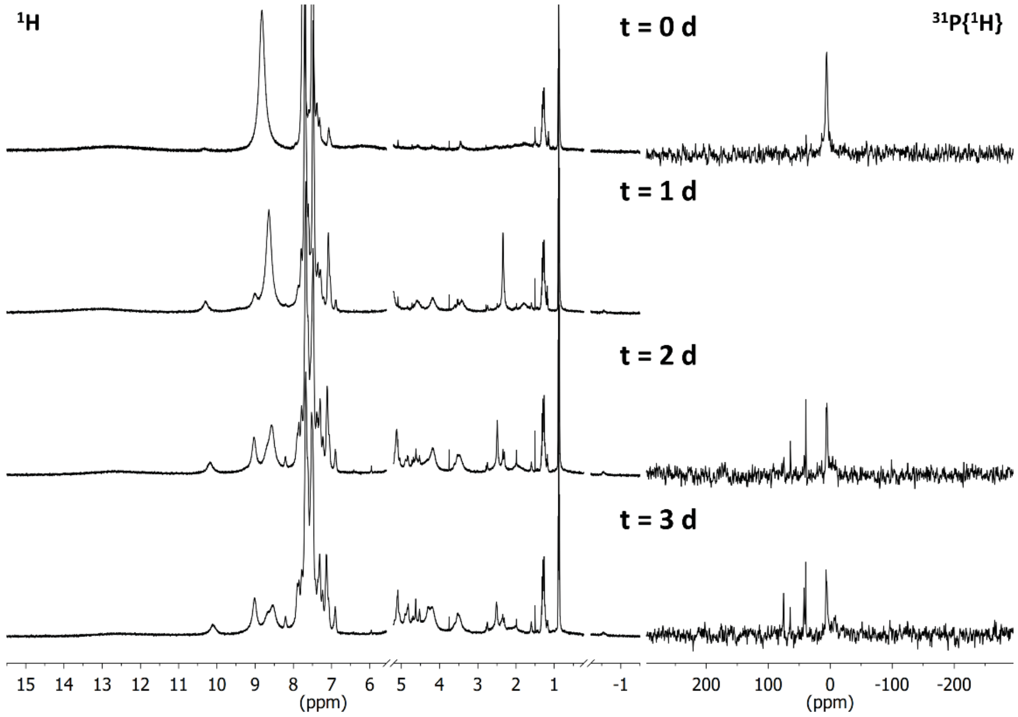

Fig. S32 Stacked ^{1}H (left) and ^{31}P{^{1}H} NMR spectra of **[1a(Au)$_3$](TEF)$_2$** in CD_2Cl_2 measured direct after sample preparation (top) and after one, two, and three days. On day one after sample preparation, no ^{31}P{^{1}H} NMR spectrum was recorded. For clarity, the signals attributable to $CHDCl_2$/CH_2Cl_2 and TMS have been cut out from the ^{1}H NMR spectra. ^{31}P{^{1}H} NMR spectra are shown with 100 Hz exponential line broadening.

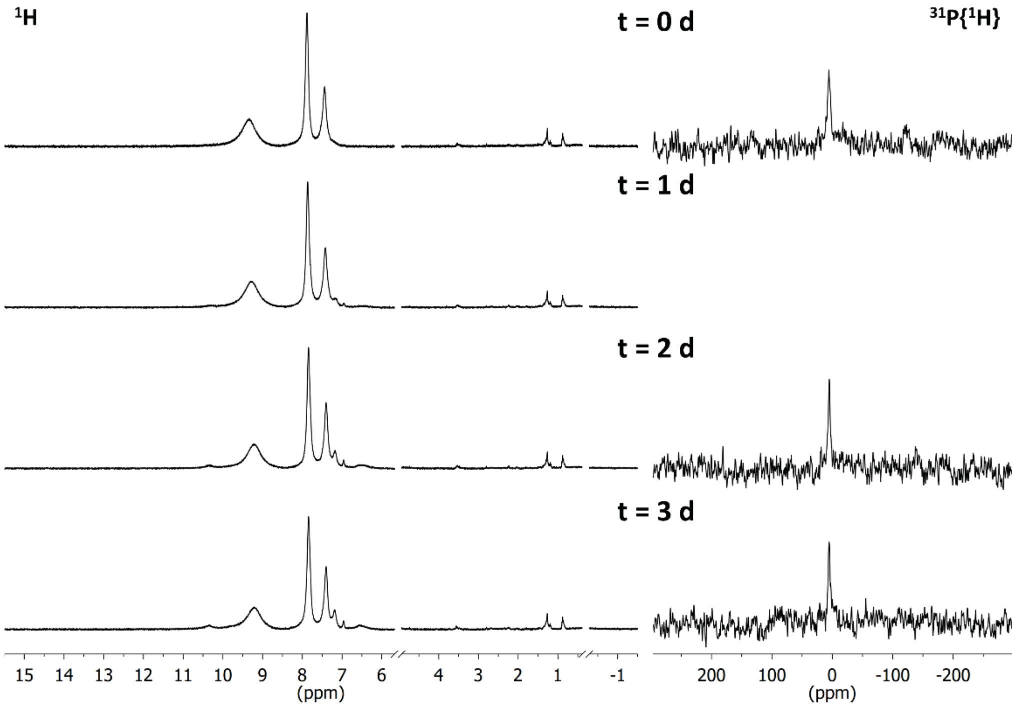

Fig. S33 Stacked ^{1}H (left) and ^{31}P{^{1}H} NMR spectra of **[1a(Au)$_3$](TEF)$_3$** in CD_2Cl_2 measured direct after sample preparation (top) and after one, two, and three days. On day one after sample preparation, no ^{31}P{^{1}H} NMR spectrum was recorded. For clarity, the signals attributable to $CHDCl_2$/CH_2Cl_2 and TMS have been cut out from the ^{1}H NMR spectra. ^{31}P{^{1}H} NMR spectra are shown with 150 Hz exponential line broadening.

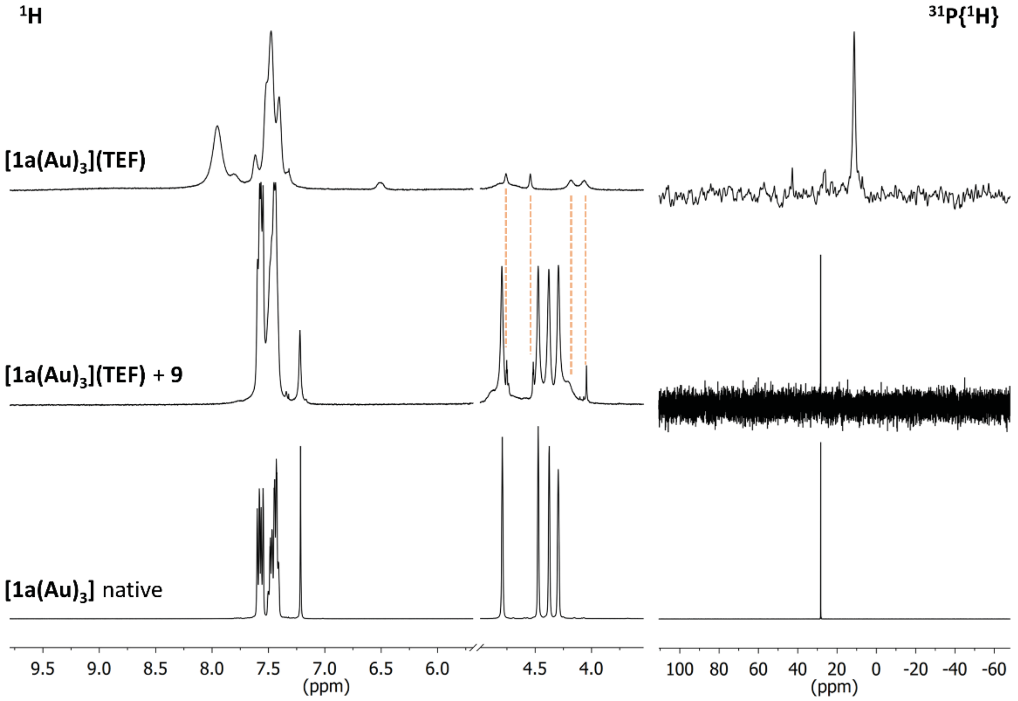

Fig. S34 Stacked ^{1}H (left) and ^{31}P{^{1}H} NMR spectra showing the reduction of **[1a(Au)$_3$](TEF)** (top) with one equivalent of **9** (centre) in CD_2Cl_2, compared to the respective NMR spectra of native **[1a(Au)$_3$]** (bottom). For clarity, the signals attributable to $CHDCl_2$/CH_2Cl_2 have been excised from the ^{1}H NMR spectra and only the relevant spectral region is shown. The ^{31}P{^{1}H} NMR spectrum of **[1a(Au)$_3$](TEF)** is shown with 100 Hz exponential line broadening. Salmon-coloured dashed lines show a persistent impurity in the ^{1}H NMR spectra of **[1a(Au)$_3$](TEF)** (+**9**) which might be related to the minority species with ^{31}P NMR resonances of about 40 ppm.

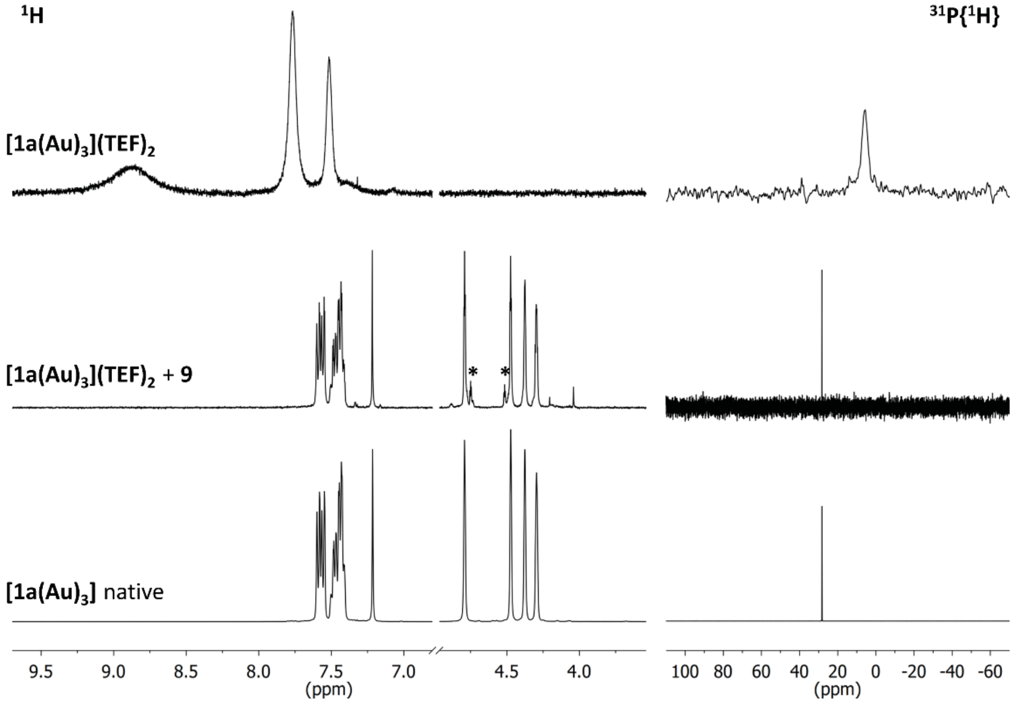

Fig. S35 Stacked ^{1}H (left) and ^{31}P{^{1}H} NMR spectra showing the reduction of **[1a(Au)$_3$](TEF)$_2$** (top) with two equivalents of **9** (centre) in CD_2Cl_2, compared to the respective NMR spectra of native **[1a(Au)$_3$]** (bottom). For clarity, the signals attributable to $CHDCl_2/CH_2Cl_2$ have been excised from the ^{1}H NMR spectra and only the relevant spectral region is shown. The ^{31}P{^{1}H} NMR spectrum of **[1a(Au)$_3$](TEF)$_2$** is shown with 100 Hz exponential line broadening. Asterisks denote ^{1}H NMR resonances attributable to residual 1,1'-diacetylferrocene.

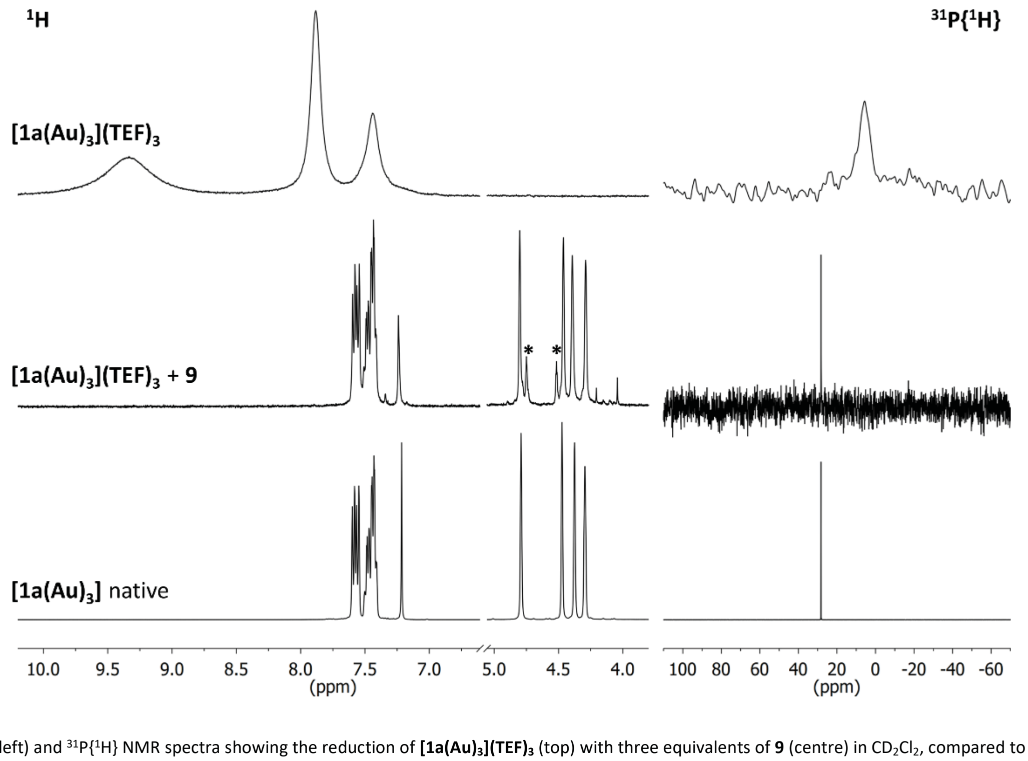

Fig. S36 Stacked ^{1}H (left) and ^{31}P{^{1}H} NMR spectra showing the reduction of **[1a(Au)$_3$](TEF)$_3$** (top) with three equivalents of **9** (centre) in CD_2Cl_2, compared to the respective NMR spectra of native **[1a(Au)$_3$]** (bottom). For clarity, the signals attributable to $CHDCl_2/CH_2Cl_2$ have been excised from the ^{1}H NMR spectra and only the relevant spectral region is shown. The ^{31}P{^{1}H} NMR spectrum of **[1a(Au)$_3$](TEF)$_3$** is shown with 150 Hz exponential line broadening. Asterisks denote ^{1}H NMR resonances attributable to residual 1,1'-diacetylferrocene.

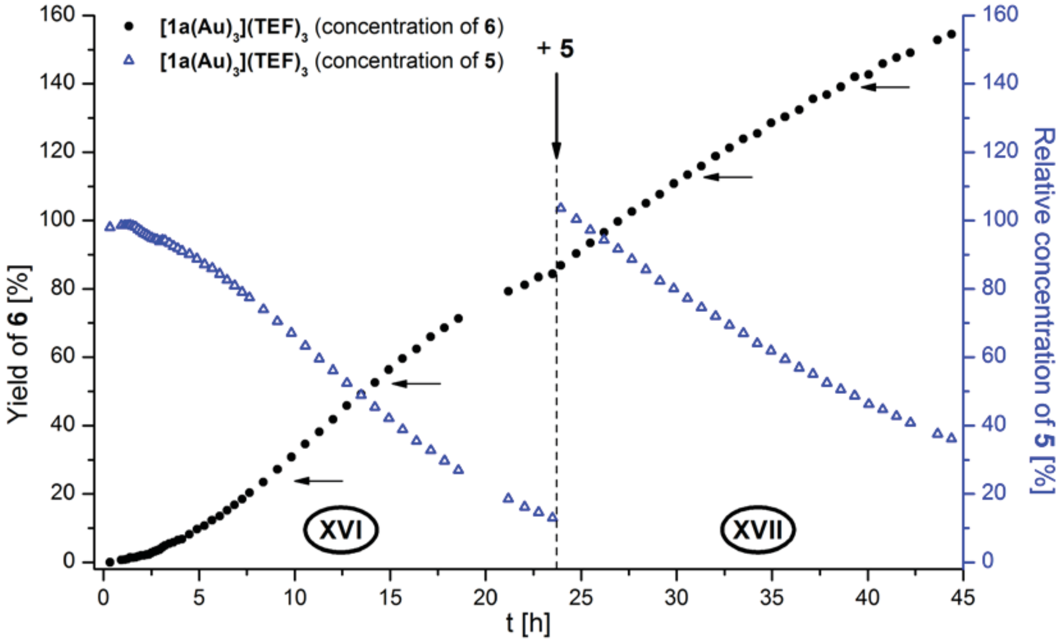

Fig. S37 Yield-over-time graph for gold(I)-catalysed cyclisation of **5** to **6** (1 mol% Au as **[1a(Au)₃](TEF)₃**, $[\mathbf{5}]_0$ = 60 mmol·L^{-1}, CD_2Cl_2, r. t.; yield determined *vs*. internal standard 1,3,5-trimethoxybenzene for protons H_o of **6** (solid black circles) and **5** (hollow blue triangles)). The vertical arrow indicates the addition of a second batch of **5** to arrive at the same catalyst:substrate ratio than for t = 0. Horizontal arrows mark the time periods chosen for linear regression. For corresponding regression parameters a (TOF), b, and R_{corr}^2, cf. Table S13.

Aiming to study the catalytic stability of **[1a(Au)$_3$](TEF)$_3$** (representative for all other oxidised species) and to, in the same way, shed light on the observed induction periods, a second batch of **5** was added to an almost-finished catalytic run (Fig. S37). The catalytic activity, determined over the same relative increase of product concentration, drops from 5.0±0.1 h^{-1} to 3.2±0.1 h^{-1}. No visible catalyst degradation (*e.g.*, precipitation of solids, formation of a gold mirror or colour changes) took place; potentially, at these relatively high concentrations of oxazoline, product inhibition of the catalyst might already operate. Most strikingly and similar to the *in situ* re-oxidation of **[1a(Au)$_3$]**, no second induction period is observed. Taken together with the independence of the order of addition of oxidant and substrate (*vide supra*), these findings indicate that the formation of the catalytically active species takes place only after oxidation but involves the substrate. Such amide-assisted activation has already been suggested by Heinze and co-workers, supported through DFT calculations, in their study of valence tautomerism from Fe^{III}/Au^{I} to Fe^{II}/Au^{II}.[70] Time-resolved UV/Vis spectroscopy of mixing 1 eq. of **[1a(Au)$_3$](TEF)$_3$** and 60 eq. of **5** in CH_2Cl_2 (Fig. S38) supports this hypothesis in that the iron(III)-associated absorptions of **[1a(Au)$_3$](TEF)$_3$** at long wavelength vanish over the course of approximately 1.5 h. The presence of an isosbestic point at about 312 nm indicates a clean conversion of **[1a(Au)$_3$](TEF)$_3$** to a different, most likely iron(II)-based, species. Valency tautomerism leading to (still paramagnetic) Fe^{II}/Au^{II} species might also be the reason for the delayed switching-off behaviour observed after addition of reductant **9**.

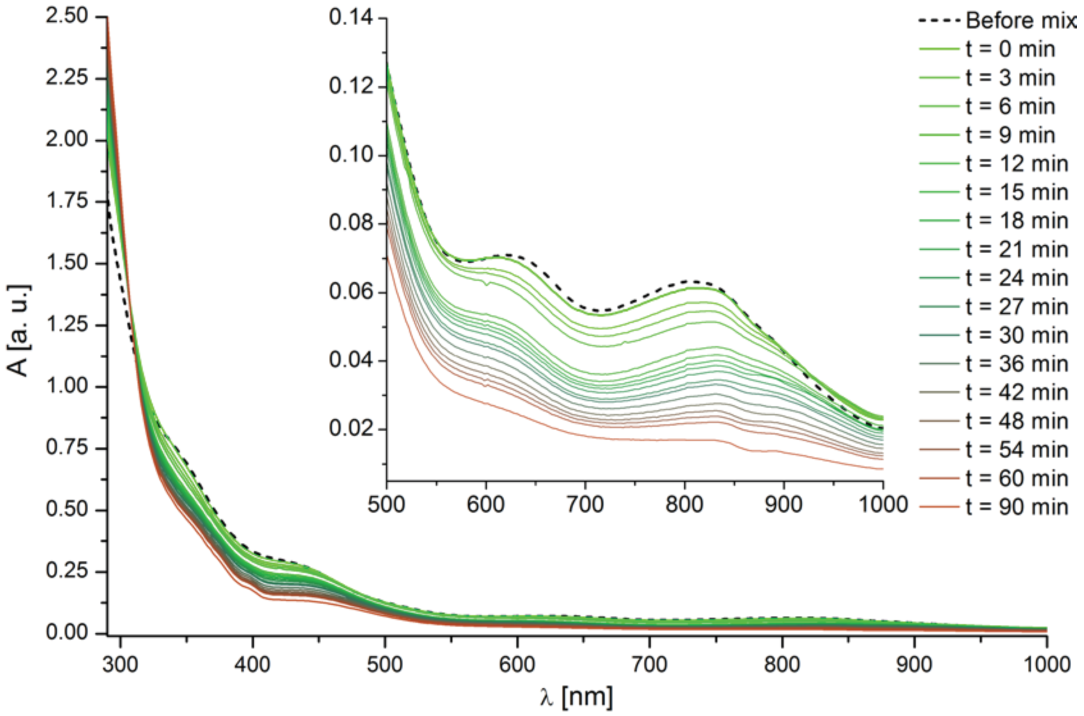

Fig. S38 Time-resolved UV/Vis plot of the reaction between 1 eq. **[1a(Au)$_3$](TEF)$_3$** and 60 eq. substrate **5** in CH_2Cl_2 during an interval of 90 min. The dashed black line signifies the superposition of the spectra of both **[1a(Au)$_3$](TEF)$_3$** and **5**, not yet mixed, in a two-compartment UV/Vis cuvette (path length 2x 0.476 cm). After this spectrum had been recorded, the cuvette was vigorously shaken (t = 0 min) and successively measured at the indicated times.

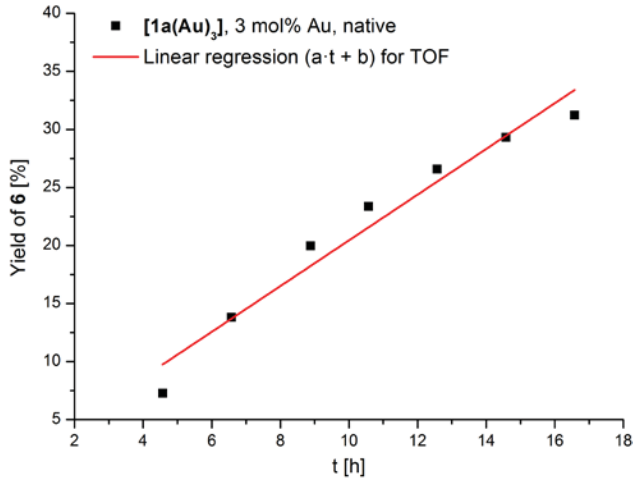

Fig. S39 Yield-over-time plot, including linear regression (red), for 3 mol% Au catalyst loading of native **[1a(Au)$_3$]** in the catalytic conversion of **5** to **6**. Concentrations were determined from protons H_m *vs*. internal standard 1,3,5-trimethoxybenzene (^{1}H NMR). For resulting a (TOF), b, and R_{corr}^2, cf. Table S13.

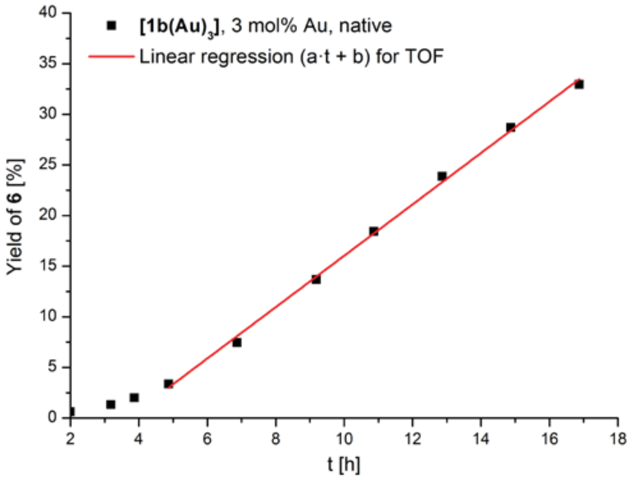

Fig. S40 Yield-over-time plot, including linear regression (red), for 3 mol% Au catalyst loading of native **[1b(Au)$_3$]** in the catalytic conversion of **5** to **6**. Concentrations were determined from protons H_m *vs*. internal standard 1,3,5-trimethoxybenzene (^{1}H NMR). For resulting a (TOF), b, and R_{corr}^2, cf. Table S13.

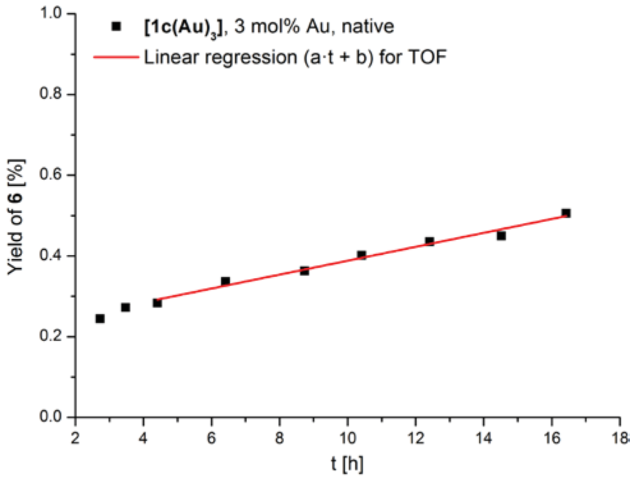

Fig. S41 Yield-over-time plot, including linear regression (red), for 3 mol% Au catalyst loading of native **[1c(Au)$_3$]** in the catalytic conversion of **5** to **6**. Concentrations were determined from protons H_m *vs*. internal standard 1,3,5-trimethoxybenzene (^{1}H NMR). For resulting a (TOF), b, and R_{corr}^2, cf. Table S13.

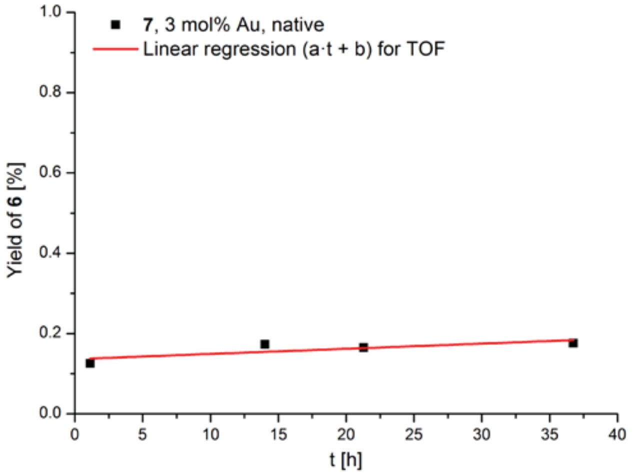

Fig. S42 Yield-over-time plot, including linear regression (red), for 3 mol% Au catalyst loading of native **7** in the catalytic conversion of **5** to **6**. Concentrations were determined from protons H_m *vs*. internal standard 1,3,5-trimethoxybenzene (^{1}H NMR). For resulting a (TOF), b, and R_{corr}^2, cf. Table S13.

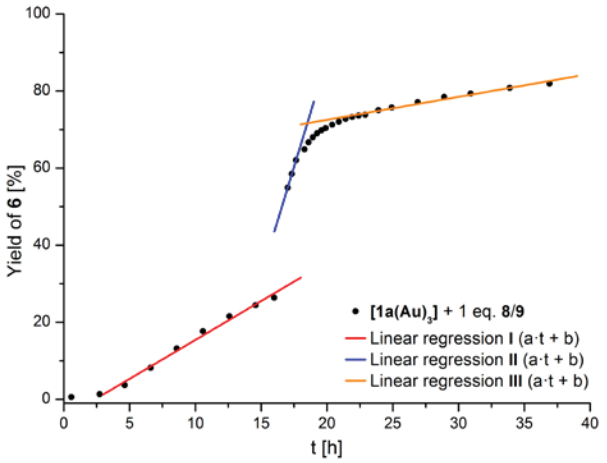

Fig. S43 Yield-over-time plot, including linear regression (red), for 3 mol% Au catalyst loading of **[1a(Au)$_3$]** and 1 eq. **8**/**9** before (**I**, red) and after (**II**, blue) oxidation and reduction (**III**, orange). Phases refer to Fig. 4, main article. Concentrations were determined from protons H_o *vs*. internal standard 1,3,5-trimethoxybenzene (^{1}H NMR). For resulting a (TOF), b, and R_{corr}^2, cf. Table S13.

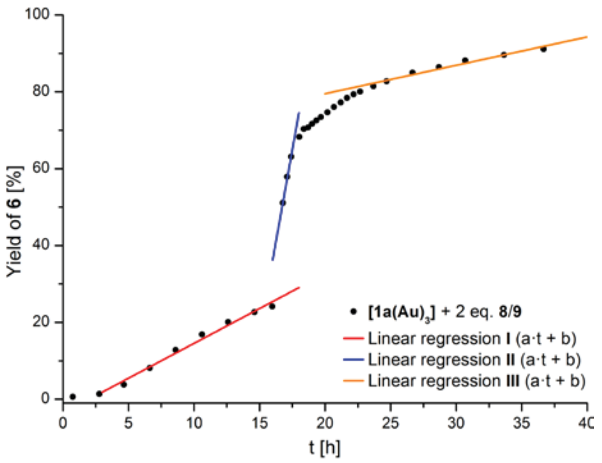

Fig. S44 Yield-over-time plot, including linear regression (red), for 3 mol% Au catalyst loading of **[1a(Au)$_3$]** and 2 eq. **8**/**9** before (**I**, red) and after (**II**, blue) oxidation and reduction (**III**, orange). Phases refer to Fig. 4, main article. Concentrations were determined from protons H_o *vs*. internal standard 1,3,5-trimethoxybenzene (^{1}H NMR). For resulting a (TOF), b, and R_{corr}^2, cf. Table S13.

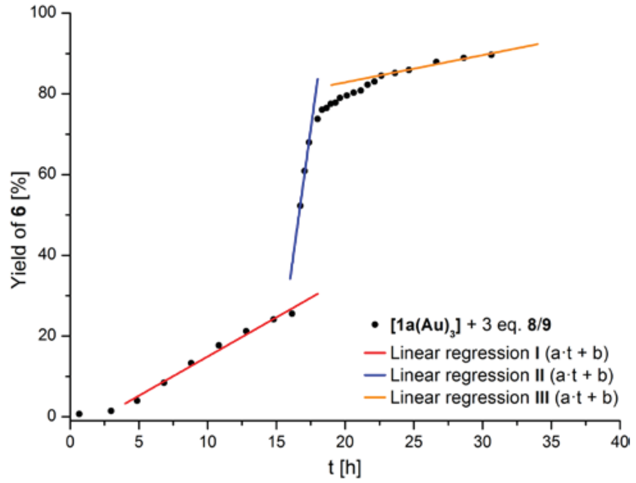

Fig. S45 Yield-over-time plot, including linear regression (red), for 3 mol% Au catalyst loading of **[1a(Au)₃]** and 3 eq. **8**/**9** before (**I**, red) and after (**II**, blue) oxidation and reduction (**III**, orange). Phases refer to Fig. 4, main article. Concentrations were determined from protons H_o *vs*. internal standard 1,3,5-trimethoxybenzene (^{1}H NMR). For resulting a (TOF), b, and R_{corr}^2, cf. Table S13.

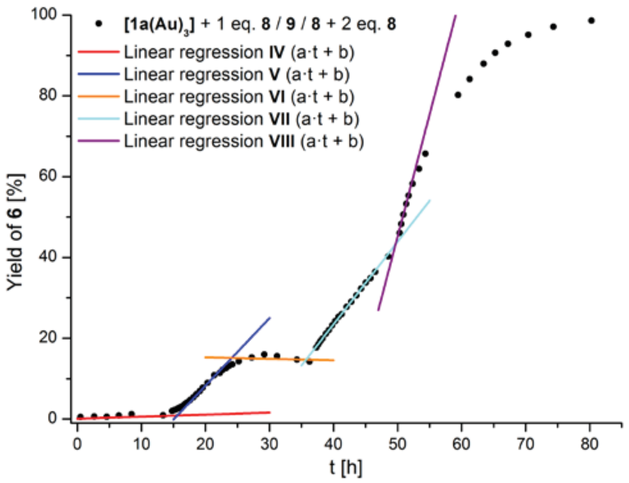

Fig. S46 Yield-over-time plot, including linear regressions, for 1 mol% Au catalyst loading of **[1a(Au)₃]** and 1 eq. **8**/**9** before (**IV**, red) and after (**V**, blue) the first oxidation, reduction (**VI**, orange), second (**VII**, light blue), and third oxidation (**VIII**, purple). Phases refer to Fig. 5, main article. Concentrations were determined from protons H_o *vs*. internal standard 1,3,5-trimethoxybenzene (^{1}H NMR). For resulting a (TOF), b, and R_{corr}^2, cf. Table S13.

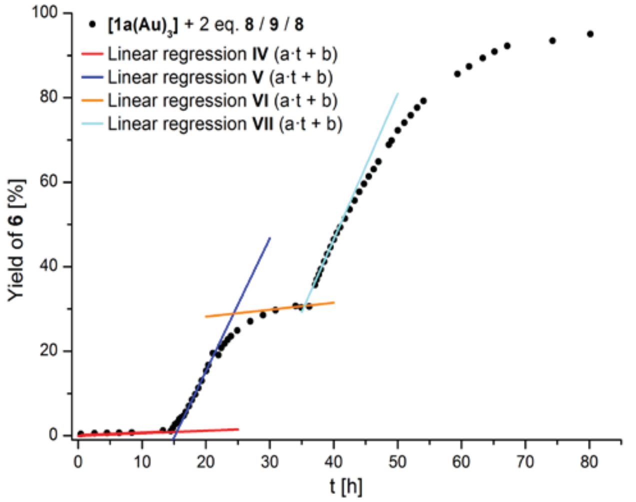

Fig. S47 Yield-over-time plot, including linear regressions, for 1 mol% Au catalyst loading of **[1a(Au)₃]** and 2 eq. **8**/**9** before (**IV**, red) and after (**V**, blue) the first oxidation, reduction (**VI**, orange), and second (**VII**, light blue) oxidation. Phases refer to Fig. 5, main article. Concentrations were determined from protons H_o *vs*. internal standard 1,3,5-trimethoxybenzene (^{1}H NMR). For resulting a (TOF), b, and R_{corr}^2, cf. Table S13

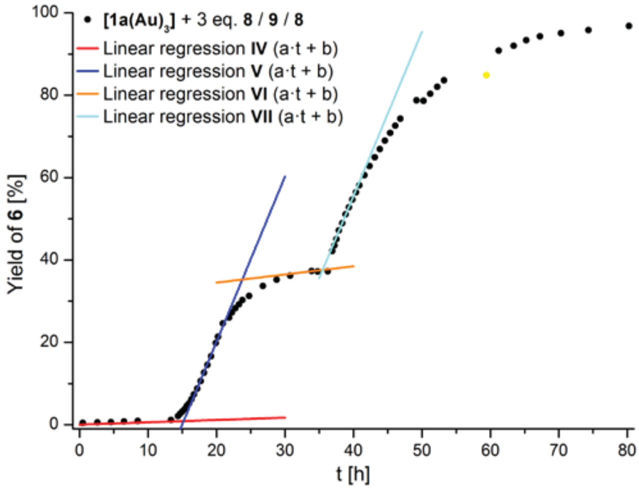

Fig. S48 Yield-over-time plot, including linear regressions, for 1 mol% Au catalyst loading of **[1a(Au)₃]** and 3 eq. **8**/**9** before (**IV**, red) and after (**V**, blue) the first oxidation, reduction (**VI**, orange), and second (**VII**, light blue) oxidation. The data point indicated in yellow has been excluded from further analyses. Phases refer to Fig. 5, main article. Concentrations were determined from protons H_o *vs*. internal standard 1,3,5-trimethoxybenzene (^{1}H NMR). For resulting a (TOF), b, and R_{corr}^2, cf. Table S13.

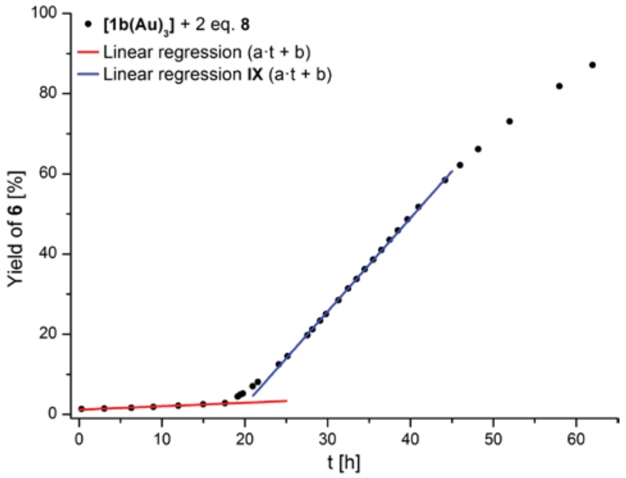

Fig. S49 Yield-over-time plot, including linear regressions, for 1 mol% Au catalyst loading of **[1b(Au)$_3$]** and 2 eq. **8** before (red) and after (**IX**, blue) the oxidation. Phases refer to Fig. 5, main article. Concentrations were determined from protons H_o *vs*. internal standard 1,3,5-trimethoxybenzene (^{1}H NMR). For resulting a (TOF), b, and R_{corr}^2, cf. Table S13.

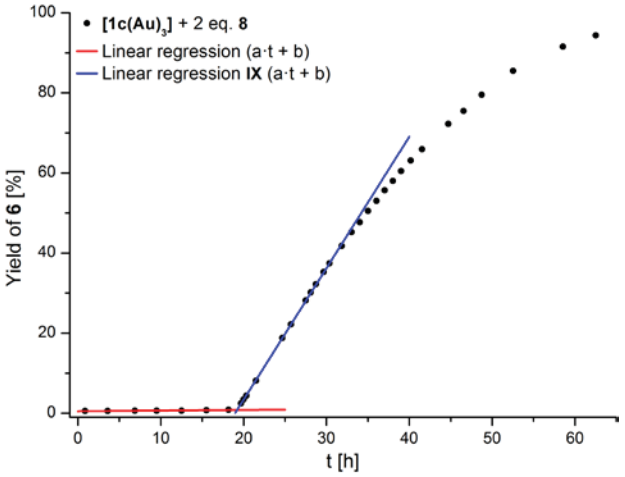

Fig. S50 Yield-over-time plot, including linear regressions, for 1 mol% Au catalyst loading of **[1c(Au)$_3$]** and 2 eq. **8** before (red) and after (**IX**, blue) the oxidation. Phases refer to Fig. 5, main article. Concentrations were determined from protons H_o *vs*. internal standard 1,3,5-trimethoxybenzene (^{1}H NMR). For resulting a (TOF), b, and R_{corr}^2, cf. Table S13.

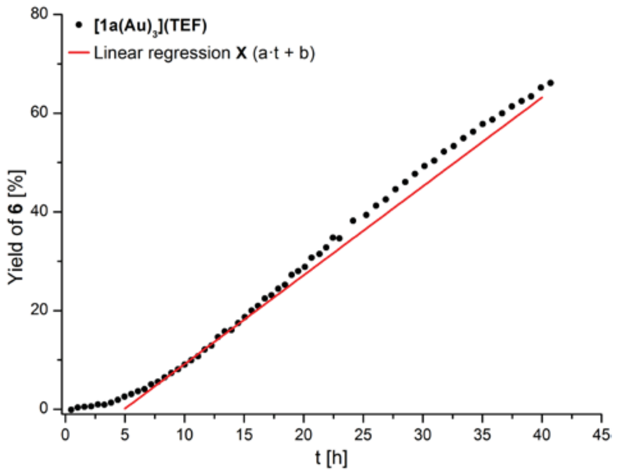

Fig. S51 Yield-over-time plot, including linear regression, for 1 mol% Au catalyst loading of **[1a(Au)$_3$](TEF)**. Concentrations were determined from protons H_o *vs*. internal standard 1,3,5-trimethoxybenzene (^{1}H NMR). For resulting a (TOF), b, and R_{corr}^2, cf. Table S13.

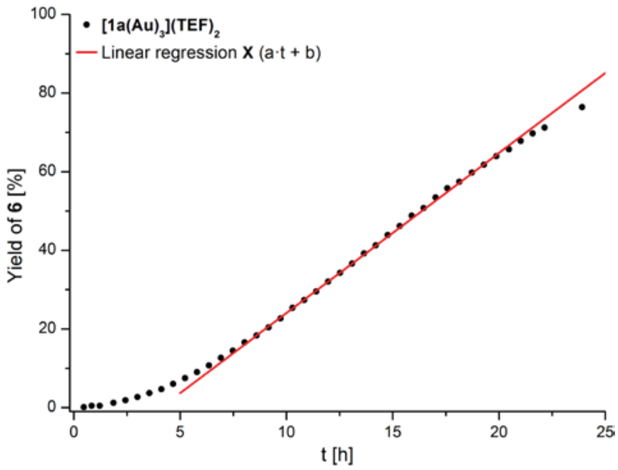

Fig. S52 Yield-over-time plot, including linear regression, for 1 mol% Au catalyst loading of **[1a(Au)$_3$](TEF)$_2$**. Concentrations were determined from protons H_o *vs*. internal standard 1,3,5-trimethoxybenzene (^{1}H NMR). For resulting a (TOF), b, and R_{corr}^2, cf. Table S13.

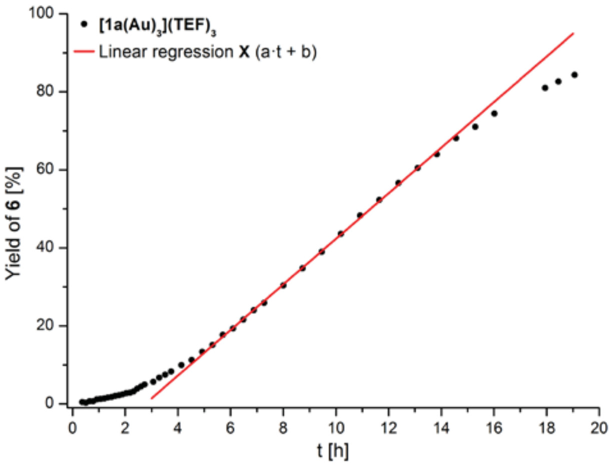

Fig. S53 Yield-over-time plot, including linear regression, for 1 mol% Au catalyst loading of **[1a(Au)$_3$](TEF)$_3$**. Concentrations were determined from protons H_o *vs*. internal standard 1,3,5-trimethoxybenzene (^{1}H NMR). For resulting a (TOF), b, and R_{corr}^2, cf. Table S13.

Table S13 Turn-over-frequencies TOF (parameter a of linear fit), intersections b**,** and coefficients of determination R_{corr}^2 derived from linear regressions of conversion-over-time plots employing **[1a-c(Au)$_3$]**, **[1a(Au)$_3$](TEF)$_n$** (n = 1–3), and **7** as (pre-)catalysts for the gold(I)-catalysed ring-closing isomerisation of **5** to **6**. TOF are listed with three significant digits except for TOF < 0.1 h^{-1}.

(Pre-)Catalyst / Loading Au	Additives[a]	Phase[b]	TOF (±error) [h^{-1}]	b (±error)	R_{corr}^2	Time span [h]
**[1a(Au)$_3$]** / 3 mol%	-	-	1.97 (±0.18)	0.75 (±2.05)	0.951	4.6 – 16.6
**[1b(Au)$_3$]** / 3 mol%	-	-	2.54 (±0.05)	−9.34 (±0.57)	0.998	4.9 – 16.9
**[1c(Au)$_3$]** / 3 mol%	-	-	0.017 (±0.001)	0.22 (±0.01)	0.980	4.4 – 16.4
**7** / 3 mol%	-	-	0.001 (±0.001)	0.14 (±0.01)	0.488 [c]	1.1 – 36.7
**[1a(Au)$_3$]** / 3 mol% Au	-	**I**	2.02 (±0.10)	−4.80 (±1.07)	0.987	4.6 – 16.0
	1.0 eq. **8**	**II**	11.2 (±0.1)	−136 (±1)	0.9999	17.0 – 17.7
	1.1 eq. **9**	**III**	0.597 (±0.029)	60.6 (±0.8)	0.979	21.9 – 36.9
	-	**I**	1.81 (±0.10)	−3.59 (±1.16)	0.981	4.7 – 16.0
	2.0 eq. **8**	**II**	19.1 (±1.1)	−270 (±18)	0.994	16.8 – 17.4
	2.2 eq. **9**	**III**	0.739 (±0.057)	64.7 (±1.7)	0.965	23.7 – 36.7
	-	**I**	1.94 (±0.10)	−4.49 (±1.15)	0.984	4.9 – 16.1
	3.0 eq. **8**	**II**	24.7 (±1.3)	−361 (±22)	0.995	17.1 – 18.0
	3.3 eq. **9**	**III**	0.674 (±0.054)	69.3 (±1.4)	0.969	22.6 – 28.6
**[1a(Au)$_3$]** / 1 mol% Au	-	**IV**	0.035 (±0.012)	0.339 (±0.083)	0.544 [c]	0.5 – 13.4
	1.0 eq. **8**	**V**	1.18 (±0.02)	−17.6 (±0.4)	0.998	17.6 – 21.4
	1.1 eq. **9**	**VI**	−0.025 (±0.059) [d]	11.6 (±1.8)	−0.195 [c]	25.2 – 36.2
	1.2 eq. **8**	**VII**	1.44 (±0.01)	−40.9 (±0.5)	0.9992	37.3 – 48.6
	2.4 eq. **8**	**VIII**	4.28 (±0.23)	−182 (±11.9)	0.985	50.3 – 52.3
	-	**IV**	0.043 (±0.004)	0.254 (±0.028)	0.947	0.4 – 13.3
	2.0 eq. **8**	**V**	2.22 (±0.06)	−33.4 (±1.1)	0.992	16.2 – 21.1
	2.2 eq. **9**	**VI**	0.117 (±0.055)	17.9 (±1.9)	0.540 [c]	30.9 – 36.2
	2.4 eq. **8**	**VII**	2.44 (±0.05)	−64.3 (±1.8)	0.997	37.1 – 41.5
	-	**IV**	0.040 (±0.003)	0.294 (±0.017)	0.977	0.5 – 13.4
	3.0 eq. **8**	**V**	2.84 (±0.07)	−42.2 (±1.3)	0.994	16.0 – 21.0
	3.3 eq. **9**	**VI**	0.140 (±0.053)	21.9 (±1.8)	0.667 [c]	30.8 – 36.3
	3.6 eq. **8**	**VII**	2.82 (±0.07)	−73.3 (±2.9)	0.993	36.9 – 41.6
	1.0 eq. **8**	**XIII**	1.80 (±0.05)	−2.94 (±0.33)	0.994	3.6 – 9.3
	1.1 eq. **9**	**XIV**	0.357 (±0.020)	15.1 (±0.4)	0.978	14.9 – 23.6
	3.6 eq. **8**	**XV**	2.81 (±0.05)	−43.3 (±1.5)	0.998	30.0 – 35.7
	2.0 eq. **8**	**XIII**	2.44 (±0.05)	−3.01 (±0.35)	0.997	3.6 – 9.4
	2.2 eq. **9**	**XIV**	0.276 (±0.026)	24.4 (±0.5)	0.941	15.0 – 23.8
	1.2 eq. **8**	**XV**	0.779 (±0.007)	11.1 (±0.2)	0.9996	28.5 – 35.1

	3.0 eq. **8**	**XIII**	3.13 (±0.06)	−6.61 (±0.41)	0.997	3.7 – 9.5
	3.3 eq. **9**	**XIV**	0.436 (±0.027)	24.3 (±0.5)	0.973	15.0 – 24.0
	2.4 eq. **8**	**XV**	1.48 (±0.02)	−0.670 (±0.944)	0.998	30.2 – 35.9
**[1b(Au)$_3$]** / 1 mol% Au	-	-	0.086 (±0.006)	1.17 (±0.06)	0.971	0.3 – 17.6
	2.0 eq. **8**	**IX**	2.33 (±0.02)	−44.3 (±0.6)	0.999	24.1 – 36.5
**[1c(Au)$_3$]** / 1 mol% Au	-	-	0.016 (±0.003)	0.508 (±0.031)	0.847	0.9 – 18.2
	2.0 eq. **8**	**IX**	3.28 (±0.02)	−62.2 (±0.4)	0.9998	19.7 – 31.9
**[1a(Au)$_3$](TEF)** / 1 mol% Au	-	**X**	1.80 (±0.06)	−8.9 (±0.6)	0.990	8.3 – 13.9
**[1a(Au)$_3$](TEF)$_2$** / 1 mol% Au	-	**X**	4.07 (±0.04)	−16.7 (±0.4)	0.9992	8.0 – 14.2
**[1a(Au)$_3$](TEF)$_3$** / 1 mol% Au	-	**X**	5.84 (±0.08)	−16.1 (±0.9)	0.999	8.0 – 13.8
	-	**XVI**	5.02 (±0.03)	-18.5 (±0.3)	0.9998	8.4 – 14.2
	-	**XVII**	3.23 (±0.05)	14.9 (±1.7)	0.997	29.8 – 38.6
**7** / 1 mol% Au	-	**XI**	0.001 (±0.001)	0.035 (±0.007)	0.250 [c]	0.2 – 20.3
	1.0 eq. **8**	**XII**	10.6 (±0.4)	-234 (±9)	0.993	22.5 – 25.8
**7** / 1 mol% Au	1.0 eq. $NaBArF_4$	-	20.1 (±0.6)	−25.0 (±2.1)	0.997	1.9 – 4.7
**[1a(Au)$_3$]** / 1 mol% Au	3.0 eq. $NaBArF_4$	-	3.15 (±0.01)	−7.08 (±0.18)	0.9999	6.8 – 24.7

[a] Equivalents are given with respect to the amount of the (pre-)catalyst used in the corresponding run; [b] With respect to Fig. 4 (main article) and S43–45 (**I**,**II**,**III**), to Fig. 5 (main article) and S46–54 (**IV**–**VIII**, **IX**, **X**), to Fig. S24 (**XI**, **XII**), to Fig. S26 (**XIII**–**XV**), and to Fig. S37 (**XVI**, **XVII**). [c] Bad linear fit due to (close-to) catalytic inactivity. [d] Apparent loss of product due to change in relaxation time of protons H_o of **6** during evolution of different paramagnetic species in solution.

Author Contributions

AS has carried out the syntheses and characterisation of the compounds (except for diacetylferrocenium teflonate **8**), including the electrochemical, the catalysis, and the NMR experiments, and has written the original draft. PC has acquired and solved the solid-state structures of all compounds but **4a** and conducted and evaluated the computational calculations. LD has prepared and characterised diacetylferrocenium teflonate **8**. EHH has supervised and administered the project, helped in acquiring funding for AS and PC and supported AS in the writing of the draft.

References

1 H. C. Clark, A. B. Goel, R. G. Goel, S. Goel and W. O. Ogini, *Inorg. Chim. Acta*, 1978, **31**, L441-L442.
2 S. O. Mihigo, W. Mammo, M. Bezabih, K. Andrae-Marobela and B. M. Abegaz, *Bioorg. Med. Chem.*, 2010, **18**, 2464.
3 M. Beinhoff, B. Karakaya and A. D. Schlüter, *Synthesis*, 2003, 79.
4 US 6310243 B1, 2001.
5 A. M. Kenwright, G. Sandford, A. J. Tadeusiak, D. S. Yufit, J. A.K. Howard, P. Kilickiran and G. Nelles, *Tetrahedron*, 2010, **66**, 9819.
6 J. Zoń, D. Kong, K. Gagnon, H. Perry, L. Holliness and A. Clearfield, *Dalton Trans.*, 2010, **39**, 11008.
7 M. S. Inkpen, S. Du, M. Driver, T. Albrecht and N. J. Long, *Dalton Trans.*, 2013, **42**, 2813.
8 N. A. Yakelis and R. G. Bergman, *Organometallics*, 2005, **24**, 3579.
9 R. Taube and S. Wache, *J. Organomet. Chem.*, 1992, **428**, 431.
10 R. Uson, A. Laguna, M. Laguna, D. A. Briggs, H. H. Murray and J. P. Fackler, Jr., in *Inorganic Syntheses*, ed. H. D. Kaesz, John Wiley & Sons, Inc, Hoboken, NJ, USA, 1989, vol. 26, pp. 85–91.
11 I. Krossing, *Chem. Eur. J.*, 2001, **7**, 490.
12 P. Wipf, Y. Aoyama and T. E. Benedum, *Org. Lett.*, 2004, **6**, 3593.
13 A. Straube, P. Coburger, M. R. Ringenberg and E. Hey-Hawkins, *Chem. Eur. J.*, 26, **2020**, 5758.
14 R. K. Harris, E. D. Becker, S. M. Cabral de Menezes, R. Goodfellow and P. Granger, *Pure Appl. Chem.*, 2001, **73**, 1795.
15 D. F. Evans, *J. Chem. Soc.*, 1959, 2003.
16 G. A. Bain and J. F. Berry, *J. Chem. Educ.*, 2008, **85**, 532.
17 CrysAlis Pro, Agilent Technologies.
18 SCALE3 ABSPACK, Oxford Diffraction, 2006.
19 G. M. Sheldrick, *Acta Crystallogr. Sect. A*, 2008, **64**, 112.
20 G. M. Sheldrick, *Acta Crystallogr. Sect. A*, 2015, **71**, 3.
21 C. F. Macrae, I. J. Bruno, J. A. Chisholm, P. R. Edgington, P. McCabe, E. Pidcock, L. Rodriguez-Monge, R. Taylor, J. van de Streek and P. A. Wood, *J. Appl. Crystallogr.*, 2008, **41**, 466.
22 I. Noviandri, K. N. Brown, D. S. Fleming, P. T. Gulyas, P. A. Lay, A. F. Masters and L. Phillips, *J. Phys. Chem. B*, 1999, **103**, 6713.
23 G. Gritzner and J. Kuta, *Pure Appl. Chem.*, 1984, **56**, 461.
24 F. Neese, *Wiley Interdiscip. Rev. Comput. Mol. Sci.*, 2012, **2**, 73.
25 F. Neese, *Wiley Interdiscip. Rev. Comput. Mol. Sci.*, 2018, **8**, e1327.
26 S. Grimme, S. Ehrlich and L. Goerigk, *J. Comput. Chem.*, 2011, **32**, 1456.
27 F. Weigend and R. Ahlrichs, *Phys. Chem. Chem. Phys.*, 2005, **7**, 3297.
28 S. Grimme, J. Antony, S. Ehrlich and H. Krieg, *J. Chem. Phys.*, 2010, **132**, 154104.
29 A. D. Becke, *Phys. Rev. A*, 1988, **38**, 3098.
30 J. P. Perdew, *Phys. Rev. B*, 1986, **33**, 8822.
31 D. Andrae, U. Häußermann, M. Dolg, H. Stoll and H. Preuß, *Theor. Chim. Acta*, 1990, **77**, 123.
32 T.-Y. Dong and D. N. Hendrickson, *Bull. Inst. Chem., Acad. Sin.*, 1987, **34**, 67.
33 A. Dolmella and G. Bandoli, *Coord. Chem. Rev.*, 2000, **209**, 161.

34 P. Štěpnička, I. Císařová, J. Sedláček, J. Vohlídal and M. Polášek, *Collect. Czech. Chem. Commun.*, 1997, **62**, 1577.
35 U. Pfaff, A. Hildebrandt, D. Schaarschmidt, T. Hahn, S. Liebing, J. Kortus and H. Lang, *Organometallics*, 2012, **31**, 6761.
36 H. Horn, F. Rudolph, R. Ahlrichs and K. Merzweiler, *Z. Naturforsch., B.: Chem. Sci.*, 1992, **47**, 1.
37 S. Steffens, M. H. Prosenc, J. Heck, I. Asselberghs and K. Clays, *Eur. J. Inorg. Chem.*, 2008, 1999.
38 S. Alvarez, *Dalton Trans.*, 2013, **42**, 8617.
39 D. O'Hagan, *Chem. Soc. Rev.*, 2008, **37**, 308.
40 G. Cavallo, P. Metrangolo, R. Milani, T. Pilati, A. Priimagi, G. Resnati and G. Terraneo, *Chem. Rev.*, 2016, **116**, 2478.
41 F. H. Allen, *Acta Crystallogr. Sect. B*, 2002, **58**, 380.
42 H.-K. Norheim, J. Capar, R. F. Einrem, K. J. Gagnon, C. M. Beavers, H. Vazquez-Lima and A. Ghosh, *Dalton Trans.*, 2016, **45**, 681.
43 E. Rabinovich, I. Goldberg and Z. Gross, *Chem. Eur. J.*, 2011, **17**, 12294.
44 G. C. Welch, R. Coffin, J. Peet and G. C. Bazan, *J. Am. Chem. Soc.*, 2009, **131**, 10802.
45 J. Capar, J. Conradie, C. M. Beavers and A. Ghosh, *J. Phys. Chem. A*, 2015, **119**, 3452.
46 B. Hu, M. He, Z. Yao, C. E. Schulz and J. Li, *Inorg. Chem.*, 2016, **55**, 9632.
47 D. Mandon, P. Ochenbein, J. Fischer, R. Weiss, K. Jayaraj, R. N. Austin, A. Gold, P. S. White and O. Brigaud, *Inorg. Chem.*, 1992, **31**, 2044.
48 M. Malischewski, K. Seppelt, J. Sutter, F. W. Heinemann, B. Dittrich and K. Meyer, *Angew. Chem. Int. Ed.*, 2017, **56**, 13372.
49 T.-Y. Dong, K. Chen, M.-C. Lin and L. Lee, *Organometallics*, 2005, **24**, 4198.
50 T.-Y. Dong, W.-Y. Lee, P.-T. Su, L.-S. Chang and K.-J. Lin, *Organometallics*, 1998, **17**, 3323.
51 H. Schmidbaur and A. Schier, *Chem. Soc. Rev.*, 2012, **41**, 370.
52 A. J. Blake, W. Clegg, J. M. Cole, J. S. O. Evans, P. Main, S. Parsons and D. J. Watkin, *Crystal Structure Analysis. Principles and Practice*, Oxford University Press, Oxford, 2nd edn., 2009, vol. 13.
53 J. P. Coleman, M. Fleischmann and D. Pletcher, *Electrochim. Acta*, 1973, **18**, 331.
54 V. P. Vysotsky, G. E. Salnikov and L. N. Shchegoleva, *Int. J. Quantum Chem.*, 2004, **100**, 469.
55 N. V. Vasilieva, I. G. Irtegova, N. P. Gritsan, A. V. Lonchakov, A. Y. Makarov, L. A. Shundrin and A. V. Zibarev, *J. Phys. Org. Chem.*, 2010, **23**, 536.
56 F. Barrière and W. E. Geiger, *J. Am. Chem. Soc.*, 2006, **128**, 3980.
57 M. J. Verschoor-Kirss, O. Hendricks, C. M. Verschoor, R. Conry and R. U. Kirss, *Inorg. Chim. Acta*, 2016, **450**, 30.
58 F. Barrière, R. U. Kirss and W. E. Geiger, *Organometallics*, 2005, **24**, 48.
59 D. A. Durfey, R. U. Kirss, C. Frommen and W. Feighery, *Inorg. Chem.*, 2000, **39**, 3506.
60 K. Rößler, T. Rüffer, B. Walfort, R. Packheiser, R. Holze, M. Zharnikov and H. Lang, *J. Organomet. Chem.*, 2007, **692**, 1530.
61 P. H. Rieger, *Electrochemistry*, Springer Netherlands, Dordrecht, 1994.
62 J. de Heer, *Rev. Mod. Phys.*, 1963, **35**, 631.
63 S. Klenk, S. Rupf, L. Suntrup, M. van der Meer and B. Sarkar, *Organometallics*, 2017, **36**, 2026.
64 T. A. Michaels, O. F. Pritchard, J. S. Dell, M. W. Bezpalko, W. S. Kassel and C. Nataro, *J. Organomet. Chem.*, 2019, **889**, 1.
65 J. E. Aguado, S. Canales, M. C. Gimeno, P. G. Jones, A. Laguna and M. D. Villacampa, *Dalton Trans.*, 2005, 3005.
66 M. C. Gimeno, A. Laguna, C. Sarroca and P. G. Jones, *Inorg. Chem.*, 1993, **32**, 5926.
67 L. A. Brown, J. L. Rhinehart and B. K. Long, *ACS Catal.*, 2015, **5**, 6057.
68 H.-K. Kim, J.-A. Park, K. M. Kim, S. M. Nasiruzzaman, D.-S. Kang, J. Lee, Y. Chang and T.-J. Kim, *Chem. Commun.*, 2010, **46**, 8442.
69 N. G. Connelly and W. E. Geiger, *Chem. Rev.*, 1996, **96**, 877.
70 P. Veit, C. Volkert, C. Förster, V. Ksenofontov, S. Schlicher, M. Bauer and K. Heinze, *Chem. Commun.*, 2019, **55**, 4615.

SUPPORTING INFORMATION

NMR Spectra

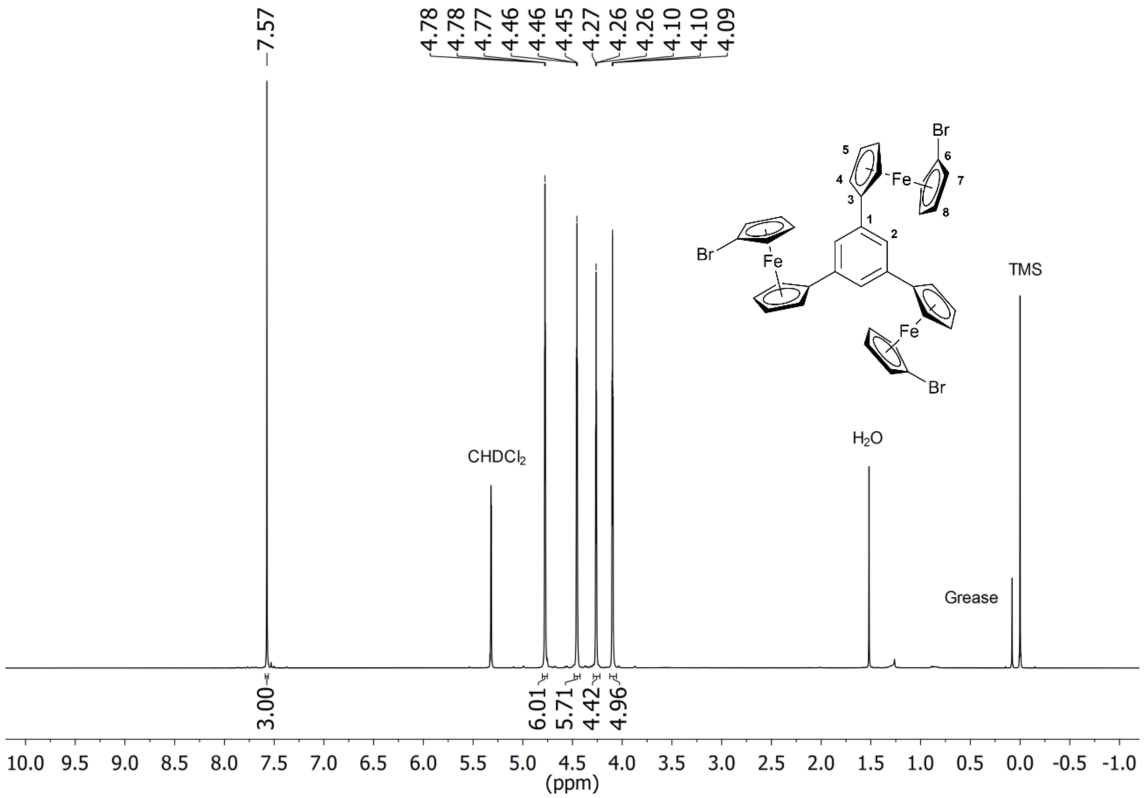

Fig. **S54** ^{1}H NMR spectrum of **4a** in CD_2Cl_2 (Assignment: δ = 7.57 (H2), 4.78 (H4/5), 4.46 (H4/5), 4.26 (H7/8), 4.10 (H7/8) ppm).

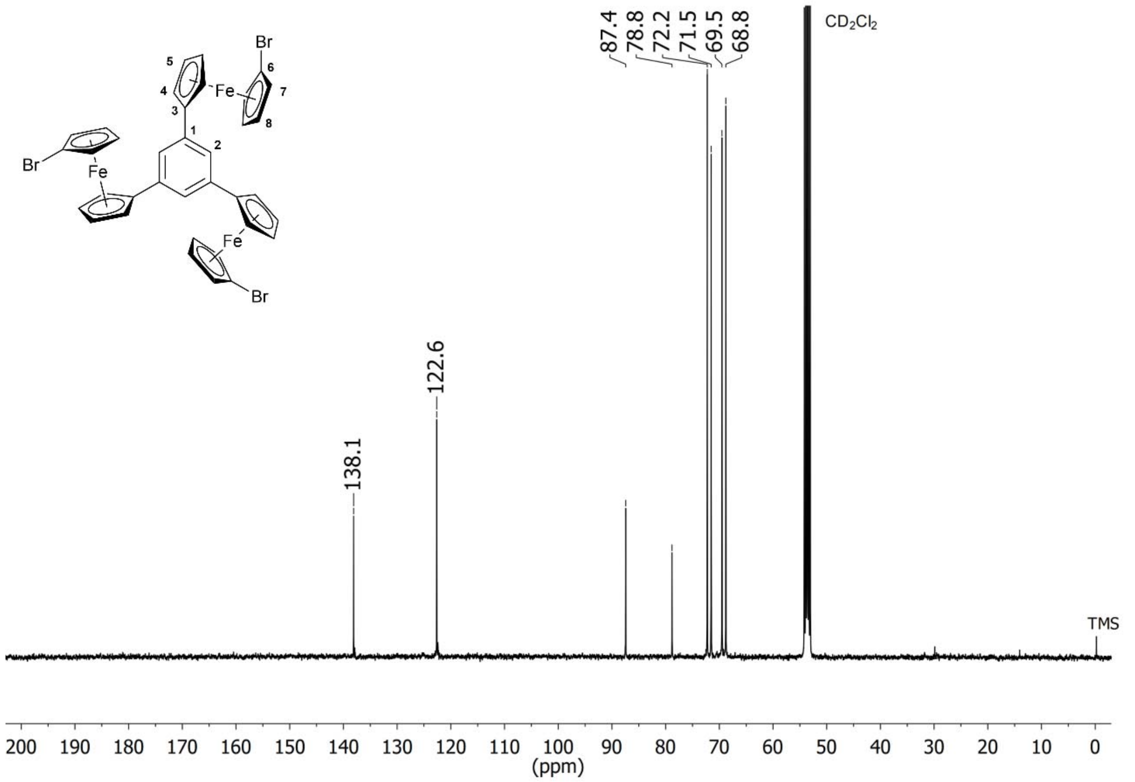

Fig. **S55** $^{13}C\{^1H\}$ NMR spectrum of **4a** in CD_2Cl_2 (Assignment: δ = 138.1 (C1), 122.6 (C2), 87.4 (C3), 78.8 (C6), 72.2 (C7/8), 71.5 (C4/5), 69.5 (C4/5), 68.8 (C7/8) ppm).

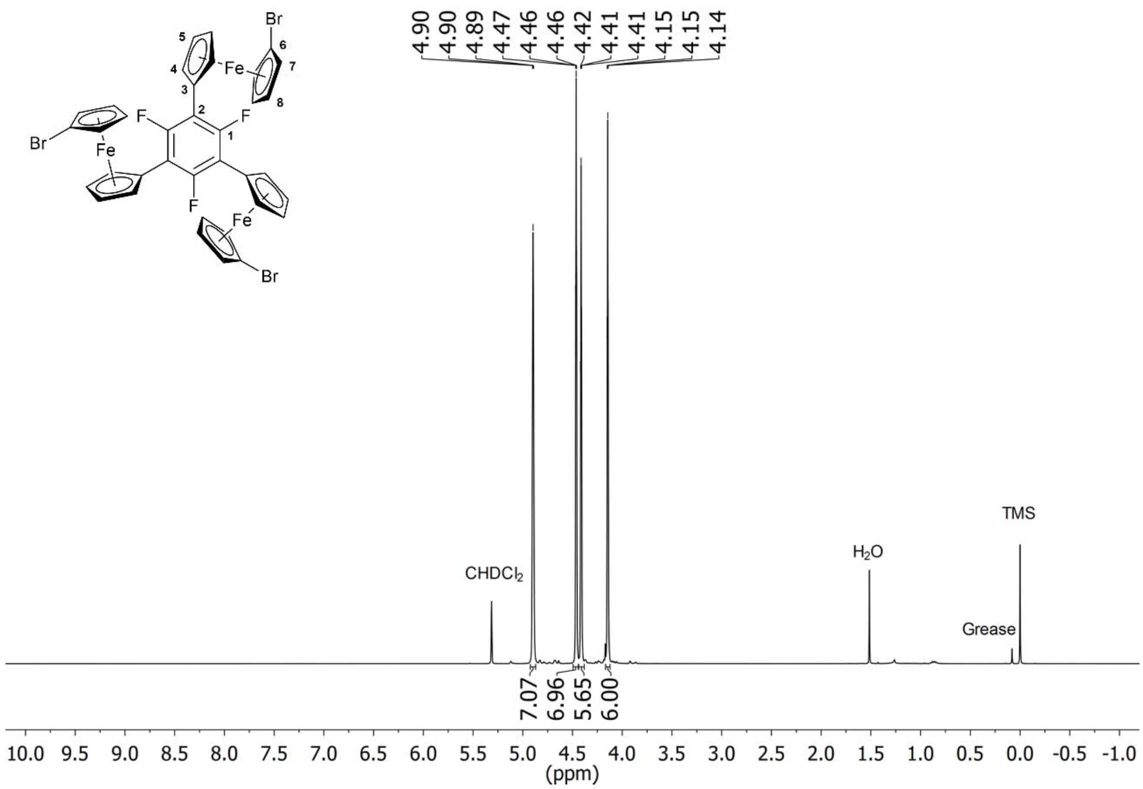

Fig. **S56** ^{1}H NMR spectrum of **4b** in CD_2Cl_2 (Assignment: δ = 4.90 (H4/5), 4.47 (H4/5), 4.41 (H7/8), 4.15 (H7/8) ppm).

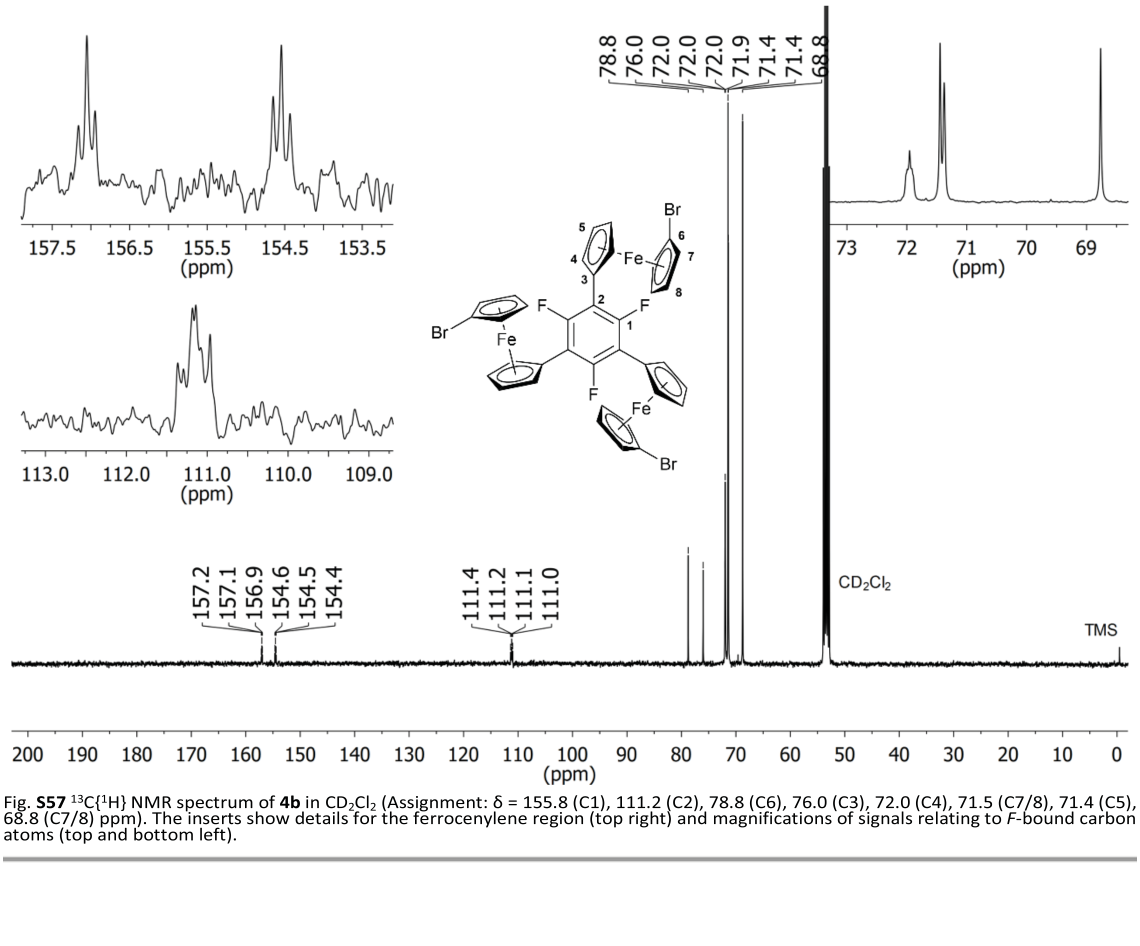

Fig. **S57** $^{13}C\{^{1}H\}$ NMR spectrum of **4b** in CD_2Cl_2 (Assignment: δ = 155.8 (C1), 111.2 (C2), 78.8 (C6), 76.0 (C3), 72.0 (C4), 71.5 (C7/8), 71.4 (C5), 68.8 (C7/8) ppm). The inserts show details for the ferrocenylene region (top right) and magnifications of signals relating to *F*-bound carbon atoms (top and bottom left).

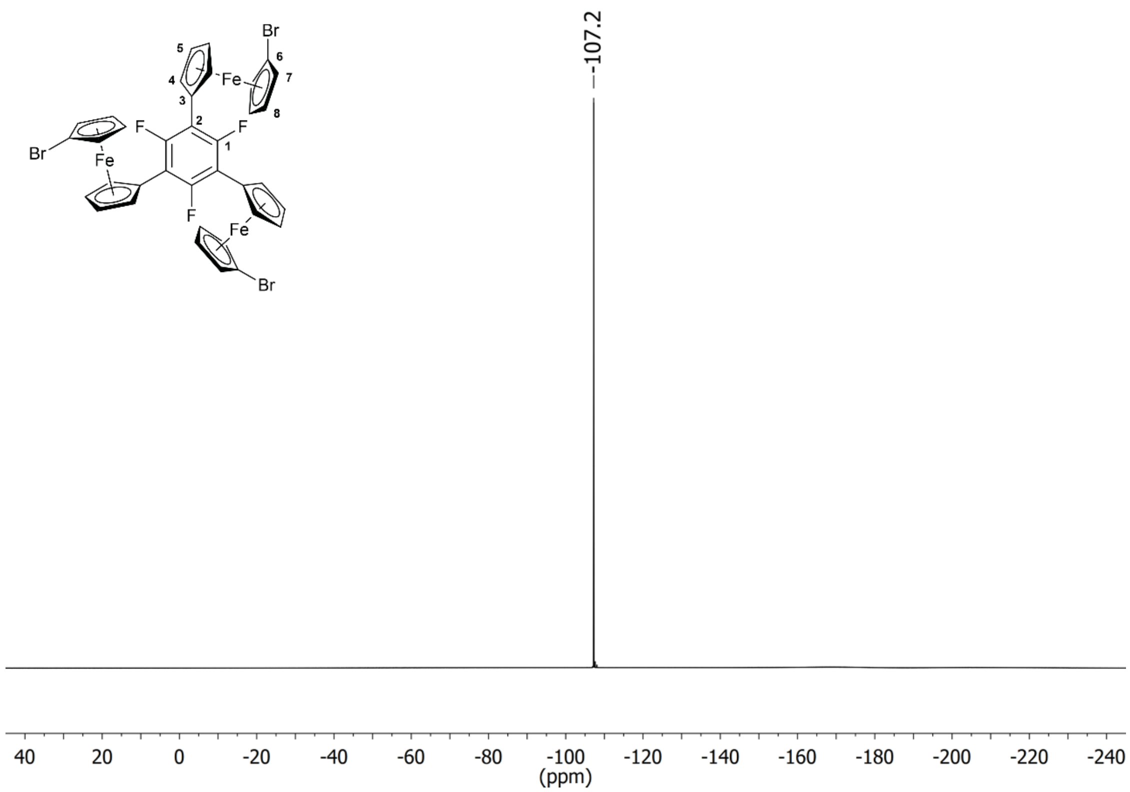

Fig. **S58** $^{19}F\{^1H\}$ NMR spectrum of **4b** in CD_2Cl_2.

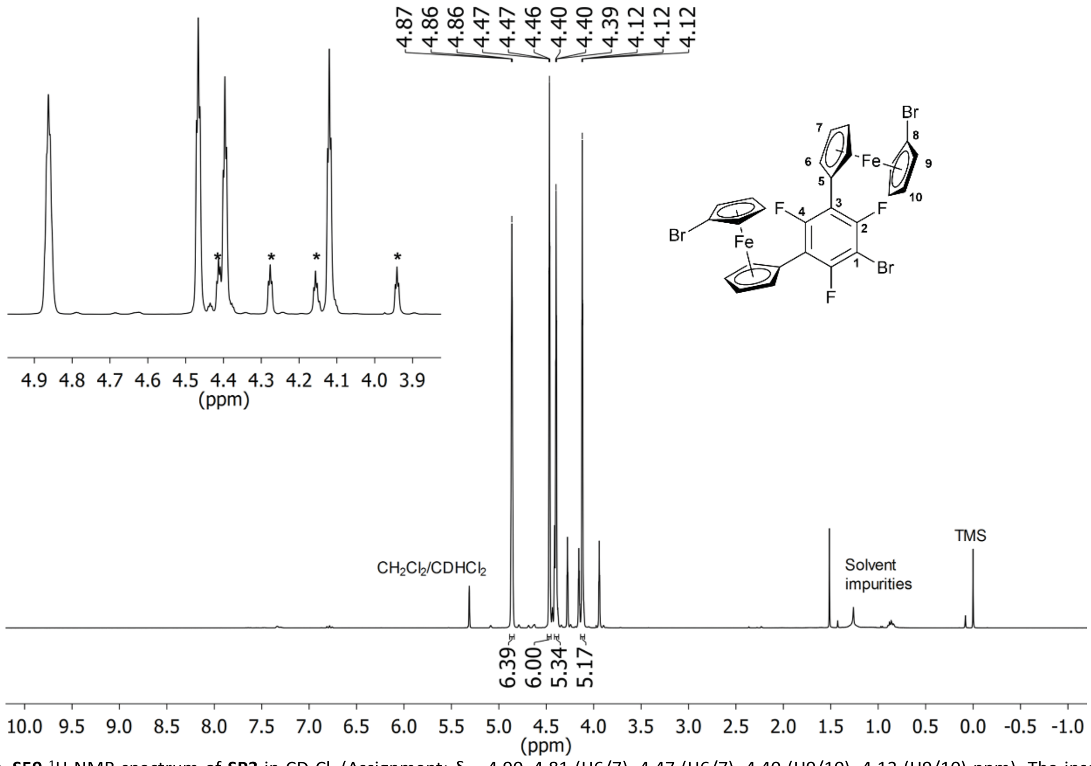

Fig. **S59** ^1H NMR spectrum of **SP3** in CD_2Cl_2 (Assignment: δ = 4.90–4.81 (H6/7), 4.47 (H6/7), 4.40 (H9/10), 4.12 (H9/10) ppm). The insert shows the ferrocenylene region in greater detail; signals marked with an asterisk (*) are linked to an impurity.

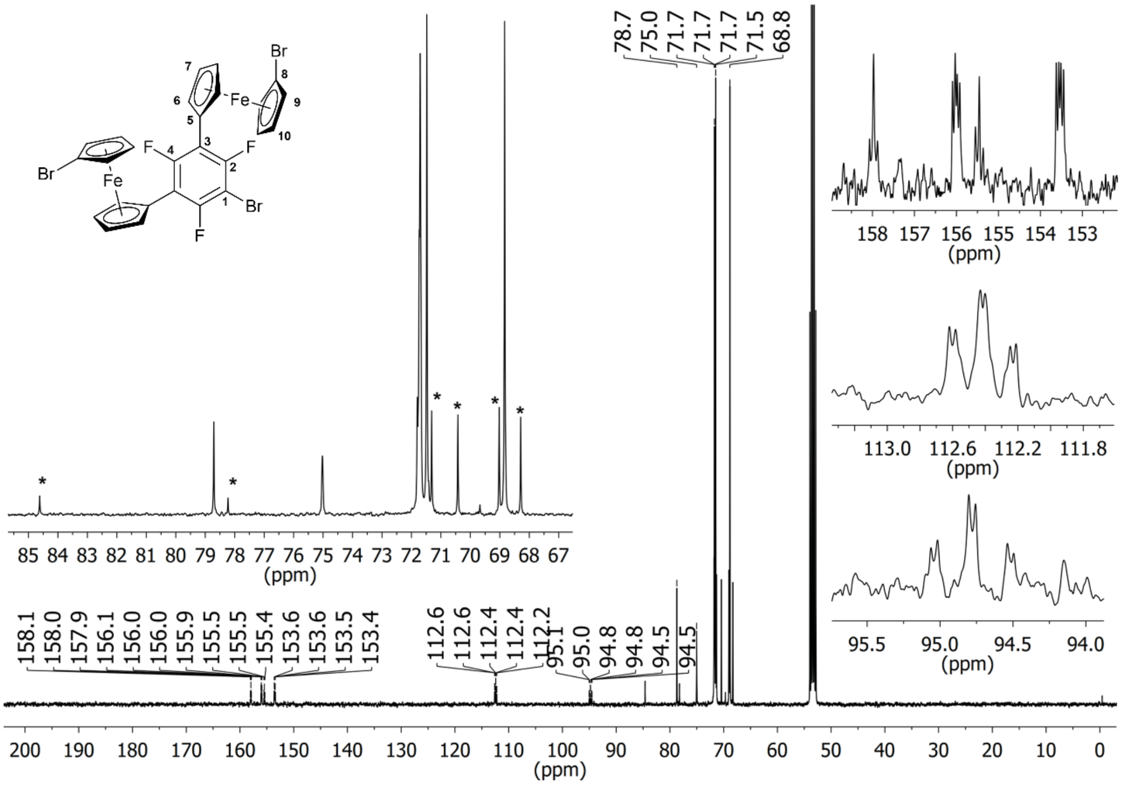

Fig. **S60** $^{13}C\{^1H\}$ NMR spectrum of **SP3** in CD_2Cl_2 (Assignment: δ = 156.7 (C4), 154.8 (C2), 112.4 (C3), 94.8 (C1), 78.7 (C8), 75.0 (C5), 71.7 (2x C6/7/9/10), 71.5 (C6/7), 68.8 (C9/10) ppm). The inserts show the ferrocenylene region in greater detail (left) as well as magnifications of the *F*-bound carbon atoms (right); signals marked with an asterisk (*) are linked to an impurity.

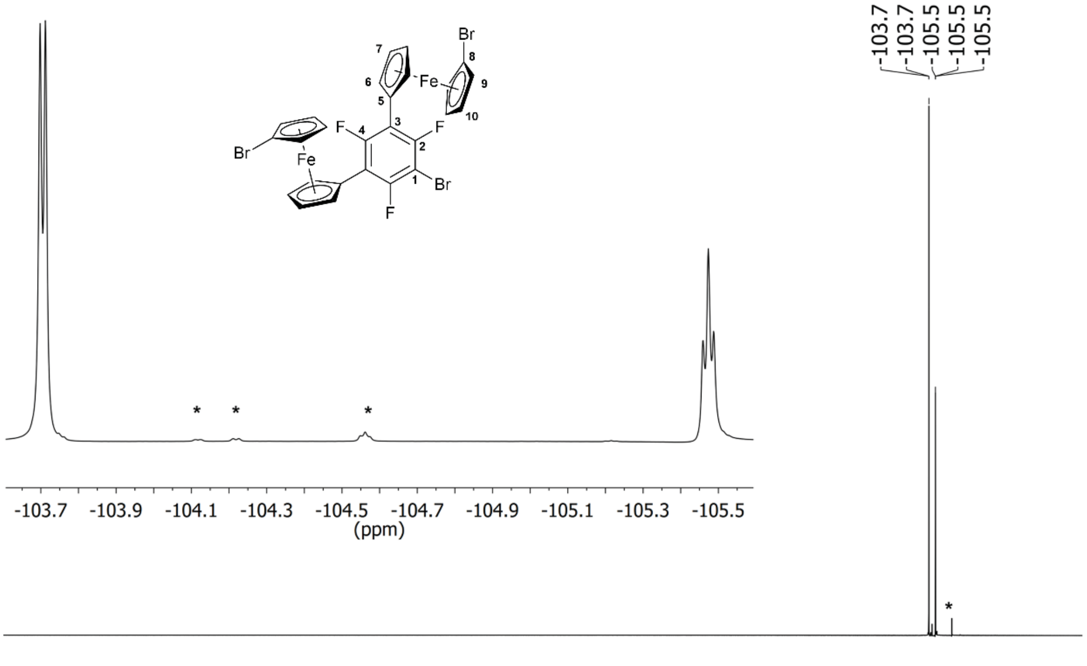

Fig. **S61** $^{19}F\{^1H\}$ NMR spectrum of **SP3** in CD_2Cl_2. The insert shows the relevant region in detail. Signals marked with an asterisk (*) are linked to an impurity.

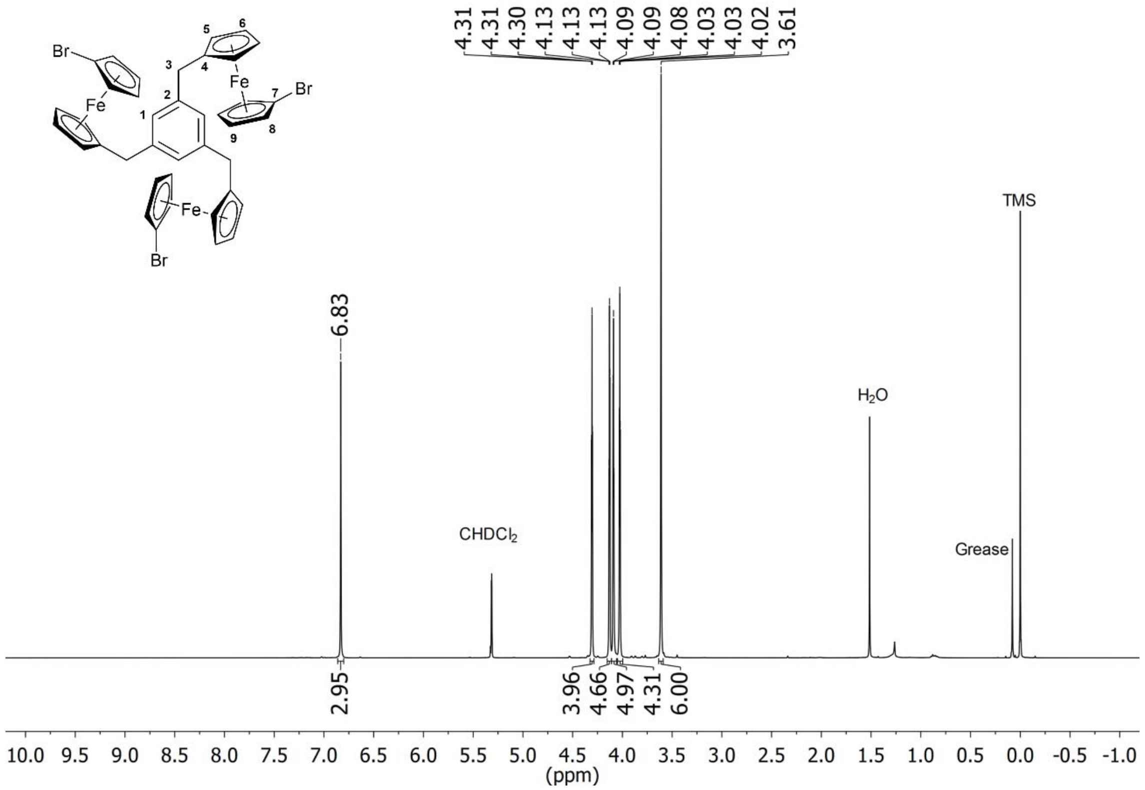

Fig. **S62** ^{1}H NMR spectrum of **4d** in CD_2Cl_2 (Assignment: δ = 6.83 (H1) 4.31 (H8/9), 4.13 (H5/6), 4.09 (H5/6), 4.03 (H8/9), 3.61 (H3) ppm).

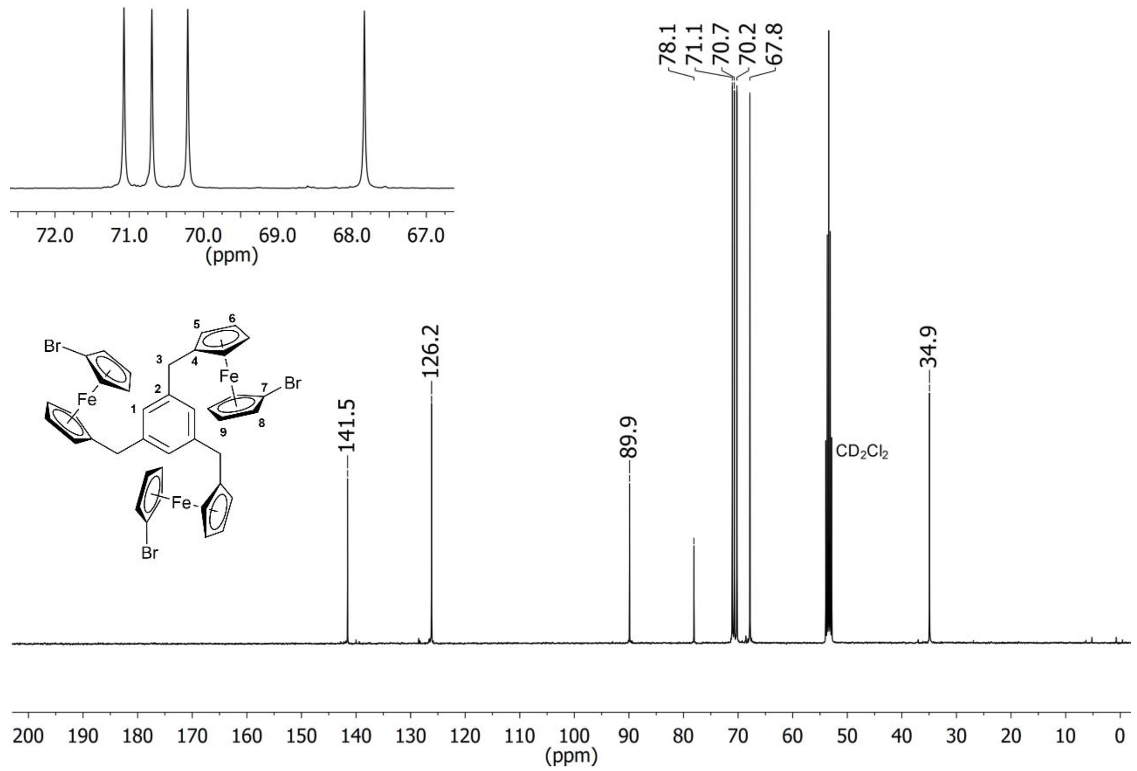

Fig. **S63** ^{13}C{^{1}H} NMR spectrum of **4d** in CD_2Cl_2 (Assignment: δ = 141.5 (C2), 126.2 (C1), 89.9 (C4), 78.1 (C7), 71.1 (C5/6), 70.7 (C8/9), 70.2 (C5/6), 67.8 (C8/9), 34.9 (C3) ppm). The insert shows the region of the ferrocenylene proton signals in detail.

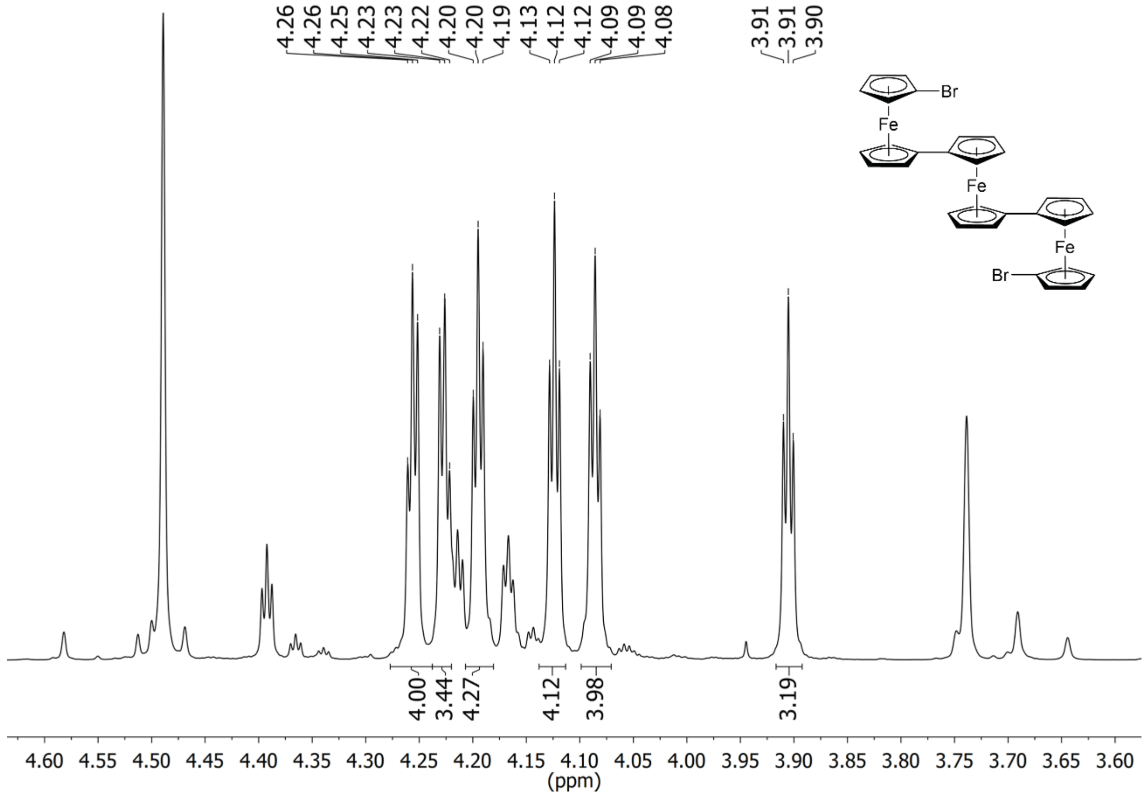

Fig. **S64** Section of ^{1}H NMR spectrum of **SP1** in CD_2Cl_2, only signals attributable to **SP1** are labelled and integrated.

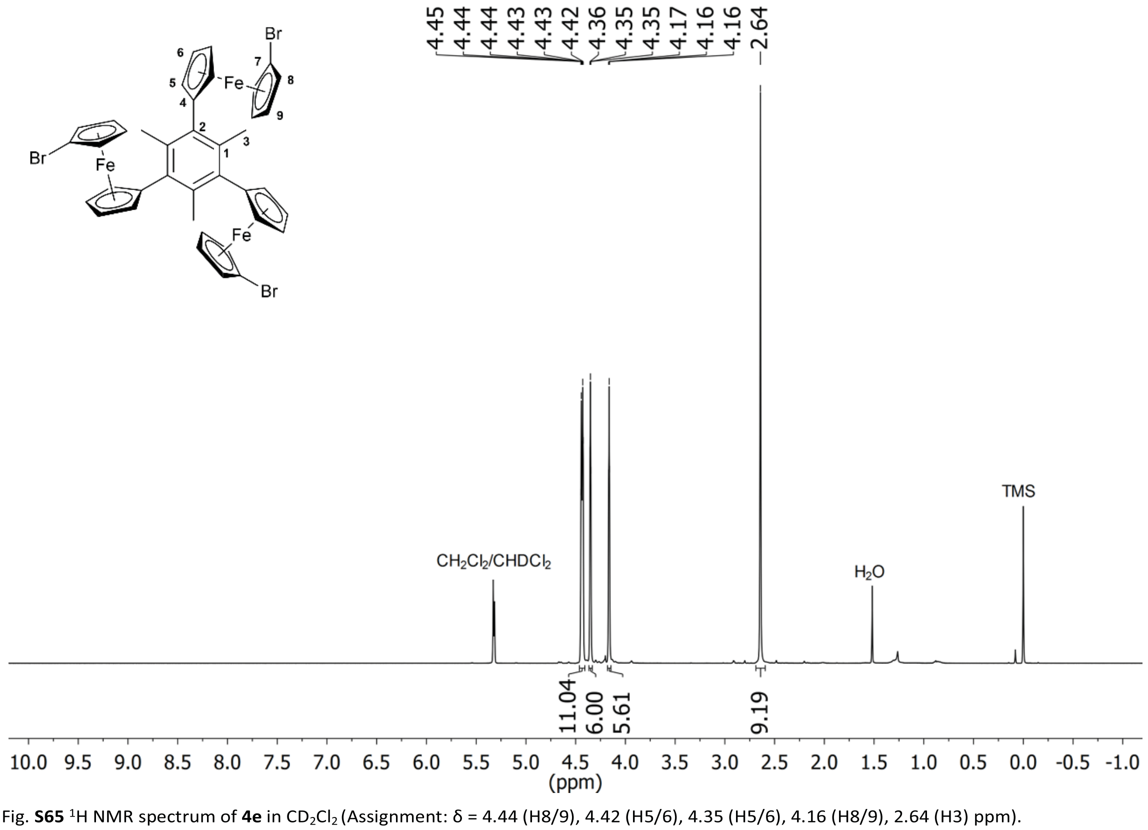

Fig. **S65** ^{1}H NMR spectrum of **4e** in CD_2Cl_2 (Assignment: δ = 4.44 (H8/9), 4.42 (H5/6), 4.35 (H5/6), 4.16 (H8/9), 2.64 (H3) ppm).

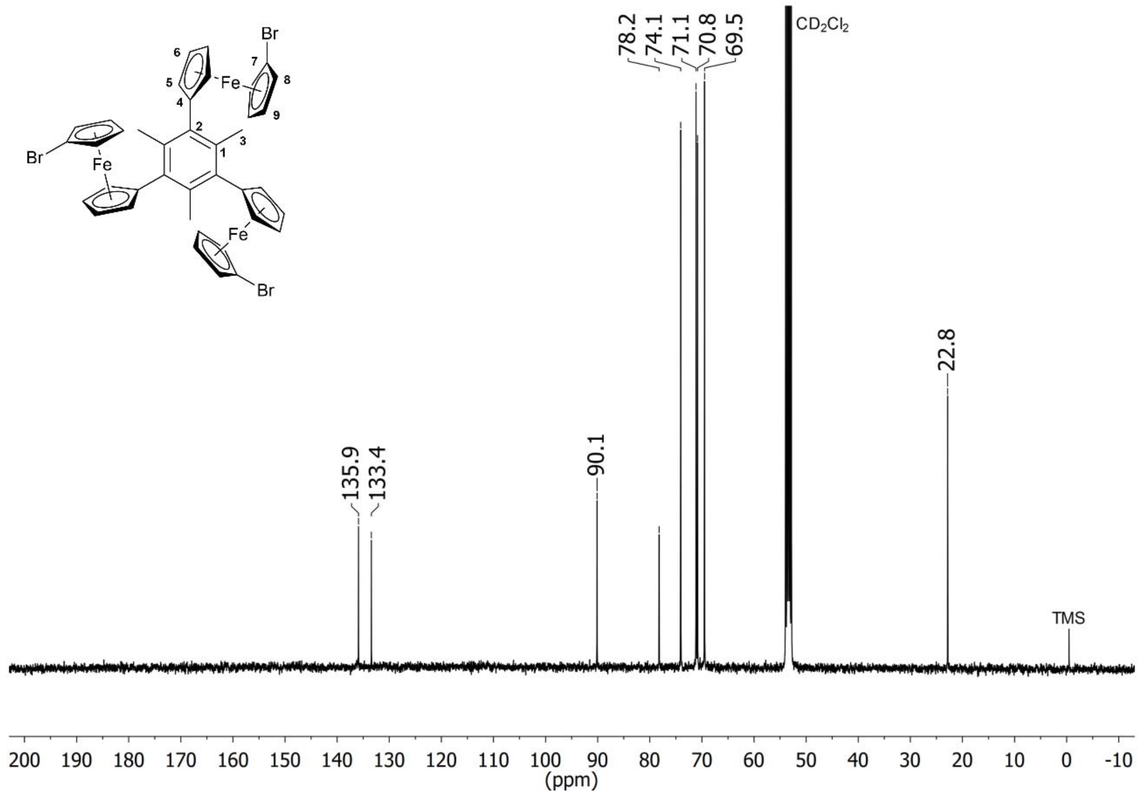

Fig. **S66** $^{13}C\{^1H\}$ NMR spectrum of **4e** in CD_2Cl_2 (Assignment: δ = 135.9 (C1), 133.4 (C2), 90.1 (C4), 78.2 (C7), 74.1 (C5/6), 71.1 (C8/9), 70.8 (C5/6), 69.5 (C8/9), 22.8 (C3) ppm).

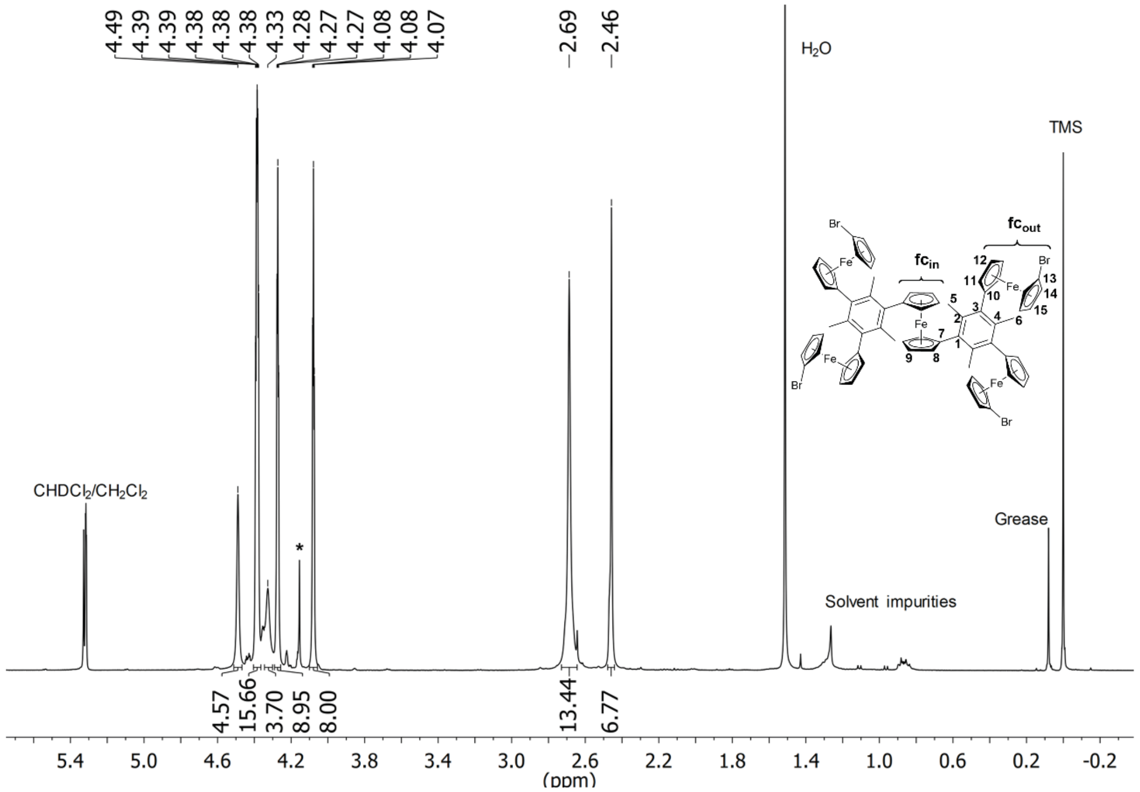

Fig. **S67** Section of the ^1H NMR spectrum of **SP2** in CD_2Cl_2 (Assignment: δ = 4.48–4.50 (fc_{in}), 4.39 (fc_{out}), 4.38 (fc_{out}), 4.32–4.34 (fc_{in}), 4.27 (fc_{in}), 4.08 (fc_{in}), 2.69 (H5), 2.46 (H6) ppm). Signals attributable to the inner ferrocenylene group are broadened. The asterisk marks a clearly discernible impurity; other signals relating to impurities are present beneath and merged with the signals of **SP2**.

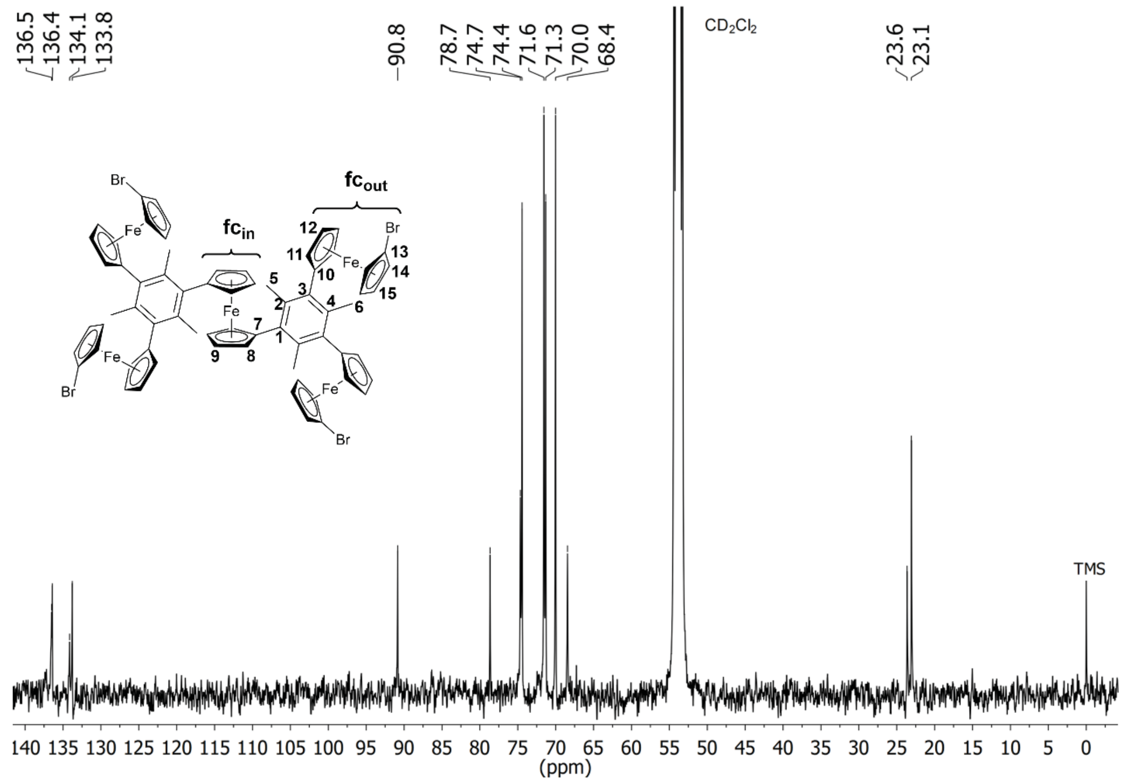

Fig. **S68** Section of the $^{13}C\{^{1}H\}$ NMR spectrum of **SP2** in CD_2Cl_2 (Assignment: δ = 136.5 (C1–4), 136.4 (C1–4), 134.1 (C1–4), 133.8 (C1–4), 90.8 (C10), 78.7 (C13), 74.7 (C8/9), 74.4 (fc_{out}), 71.5 (fc_{out}), 71.3 (fc_{out}), 70.0 (fc_{out}), 68.4 (C8/9), 23.6 (C6), 23.1 (C5) ppm).

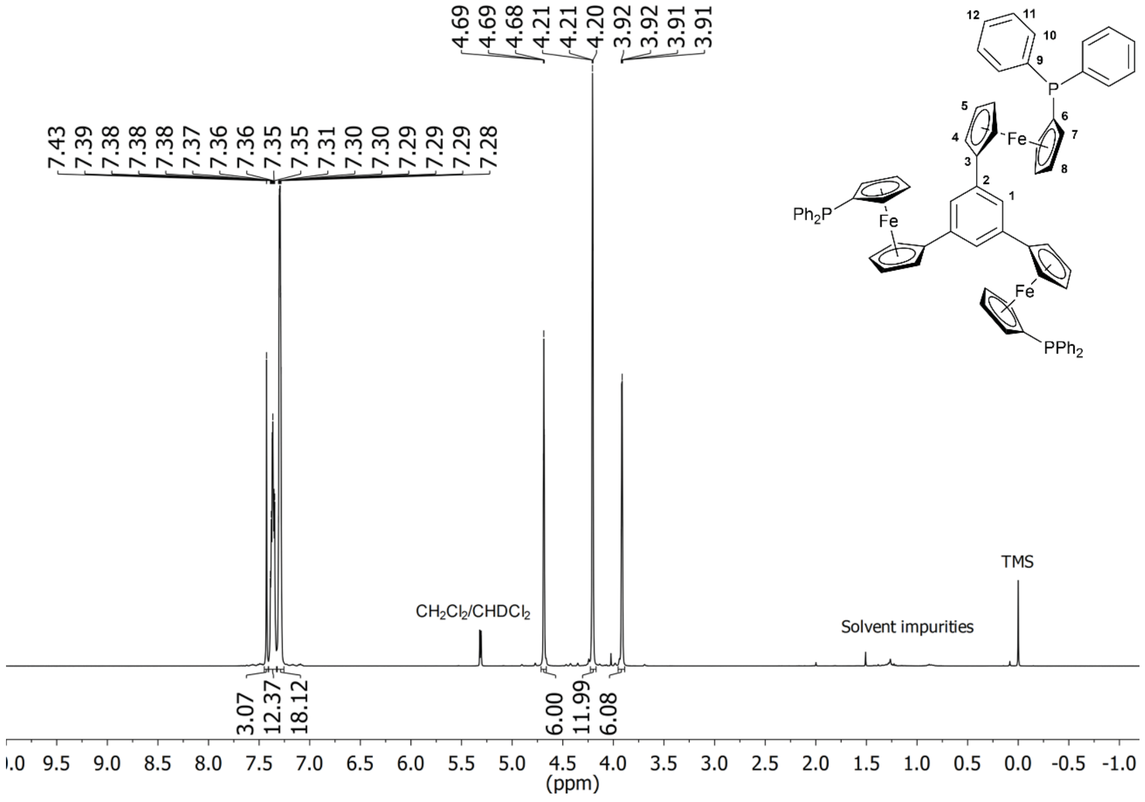

Fig. **S69** ^{1}H NMR spectrum of **1a** in CD_2Cl_2 (Assignment: δ = 7.43 (H1), 7.40–7.34 (H10), 7.33–7.27 (H11+12), 4.69 (H4/5), 4.20 (H4/5+ H8), 3.92 (H7) ppm).

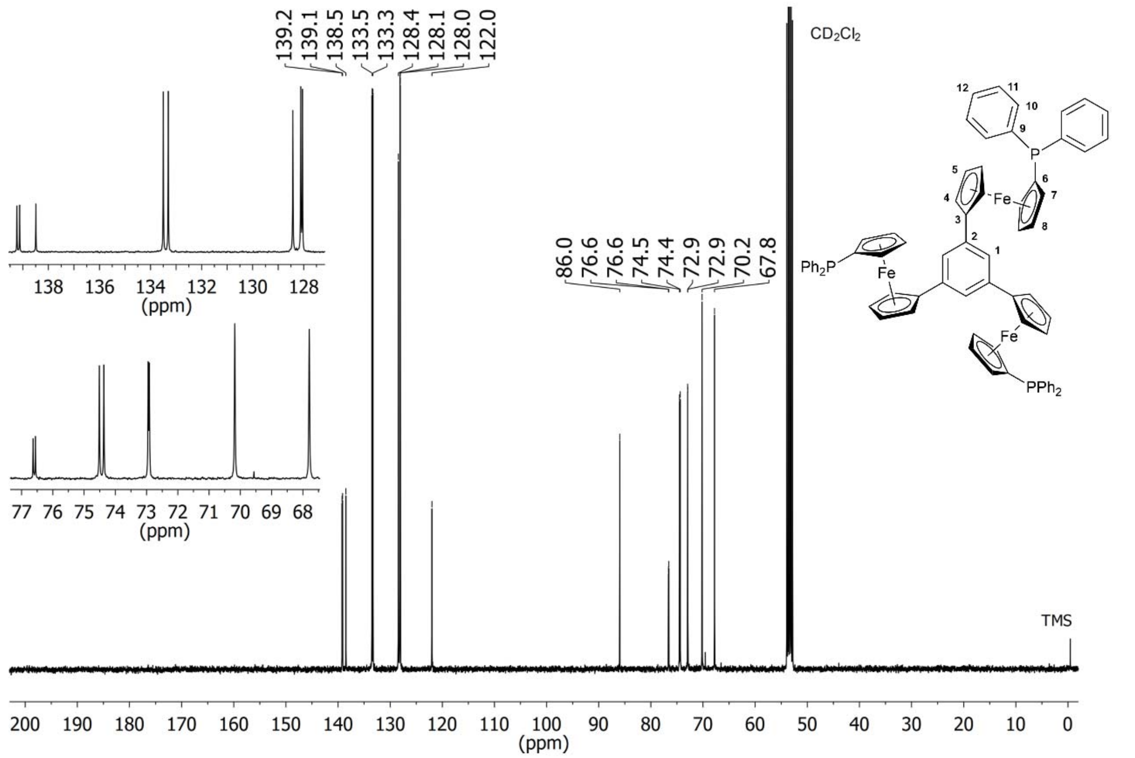

Fig. **S70** $^{13}C\{^1H\}$ NMR spectrum of **1a** in CD_2Cl_2 (Assignment: δ = 128.1 (C11), 122.0 (C1), 86.0 (C3), 76.6 (C6), 74.5 (C7), 72.9 (C8), 70.2 (C4/5), 67.8 (C4/5) ppm). The inserts show details of the phenyl (top) and ferrocenylene (bottom) spectral regions.

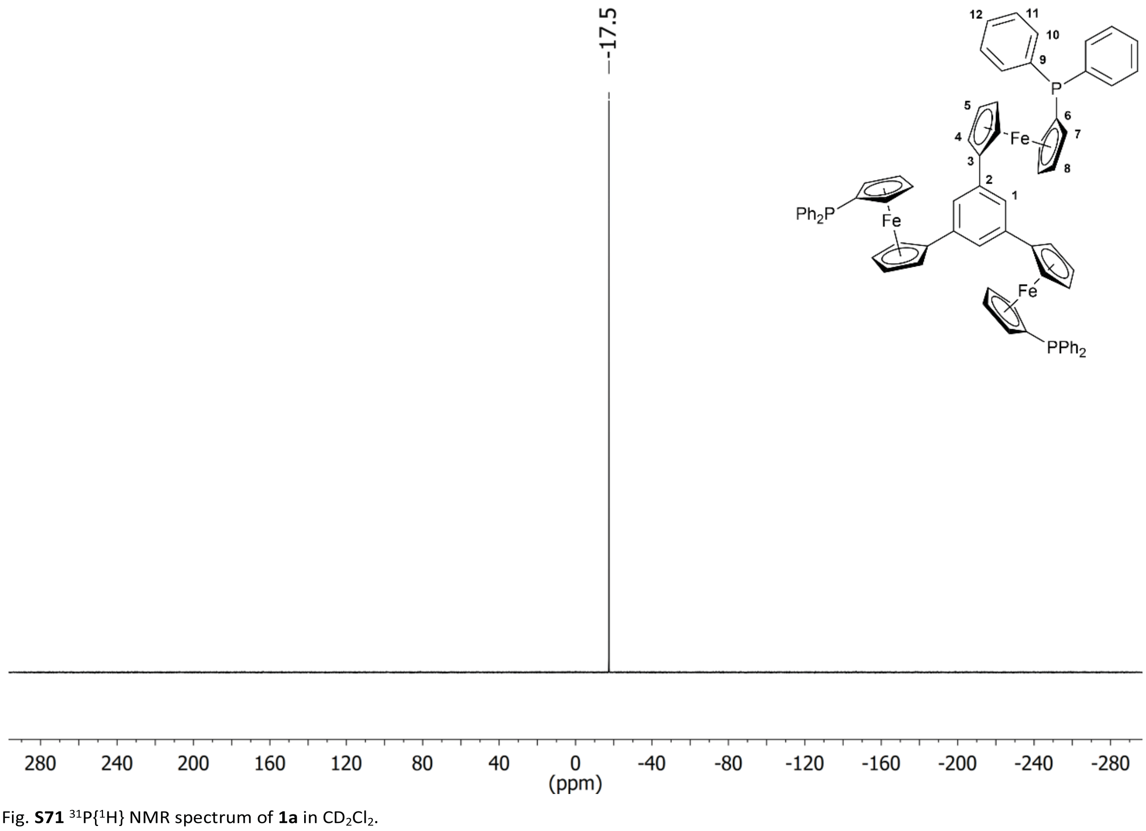

Fig. **S71** $^{31}P\{^1H\}$ NMR spectrum of **1a** in CD_2Cl_2.

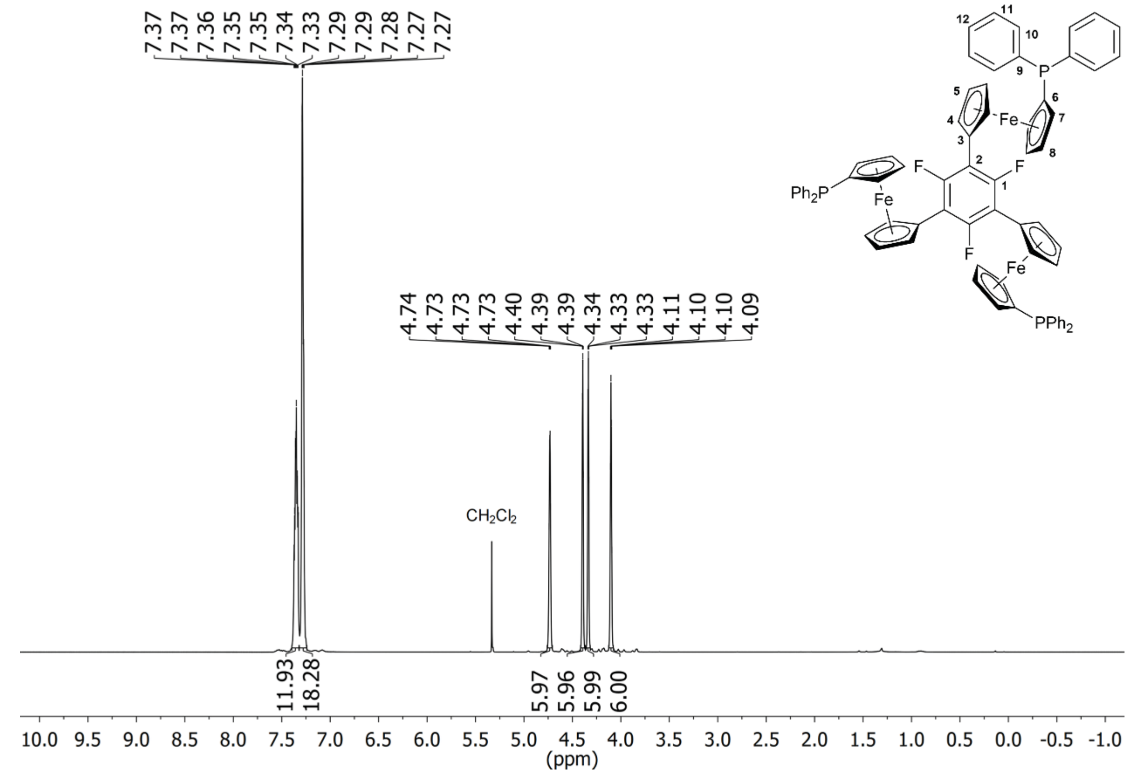

Fig. **S72** ^{1}H NMR spectrum of **1b** in CD_2Cl_2 (Assignment: δ = 7.39–7.31 (H10), 7.30–7.26 (H11+H12), 4.73 (H4), 4.39 (H8), 4.34 (H5), 4.10 (H7) ppm).

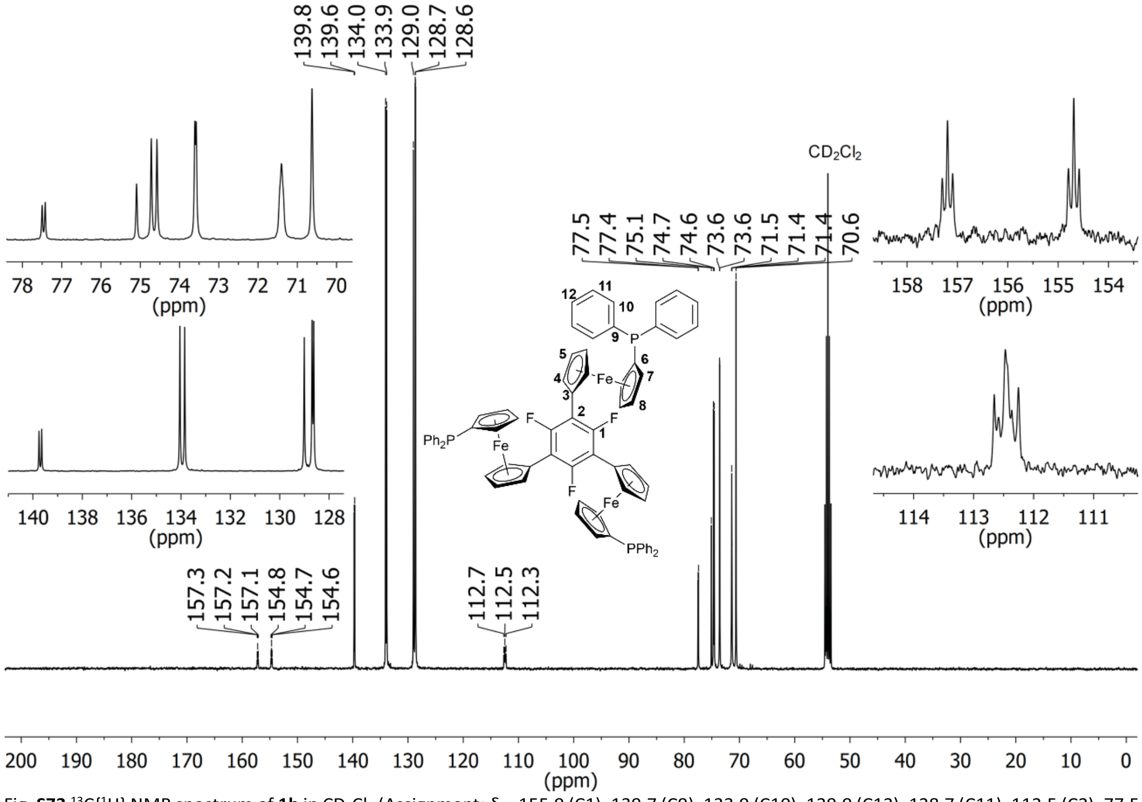

Fig. **S73** ^{13}C{^{1}H} NMR spectrum of **1b** in CD_2Cl_2 (Assignment: δ = 155.9 (C1), 139.7 (C9), 133.9 (C10), 129.0 (C12), 128.7 (C11), 112.5 (C2), 77.5 (C6), 75.1 (C3), 74.6 (C7), 73.6 (C8), 71.4 (C4), 70.6 (C5) ppm). The inserts show details of the phenyl (bottom left) and ferrocenylene (top left) spectral regions as well as magnifications of the *F*-bound arene carbon atoms (right).

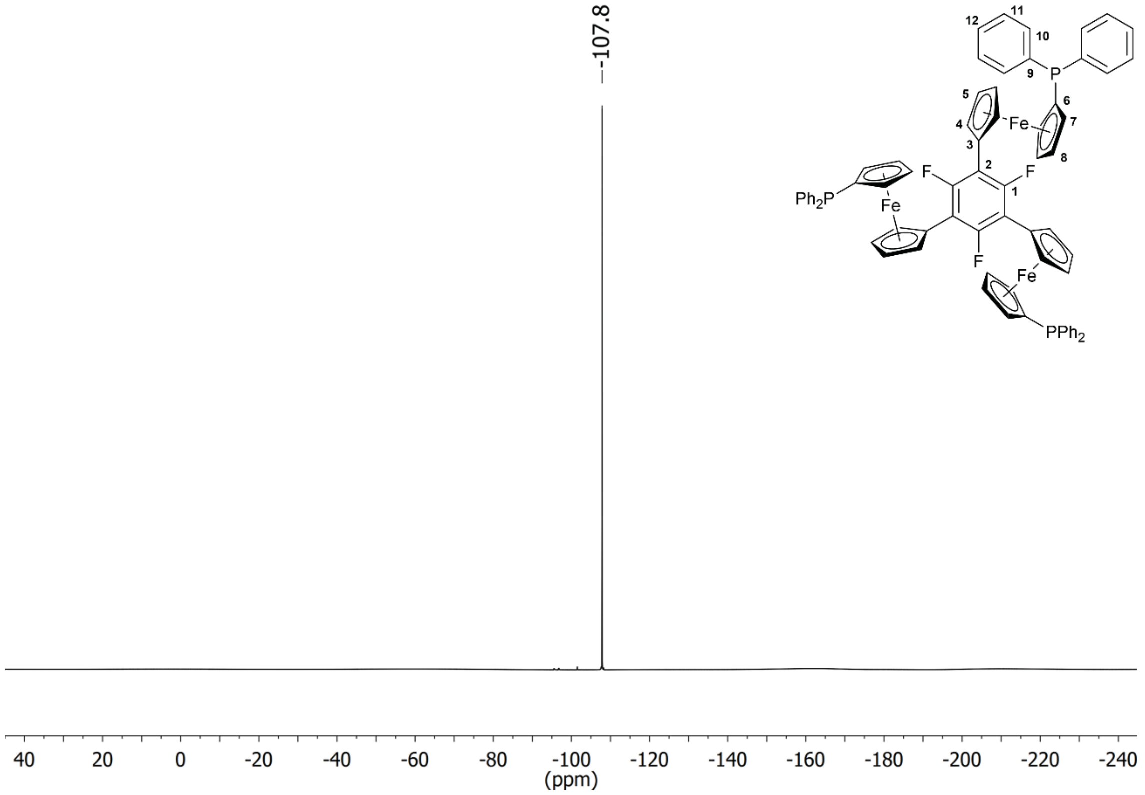

Fig. S74 $^{19}F\{^1H\}$ NMR spectrum of **1b** in CD_2Cl_2.

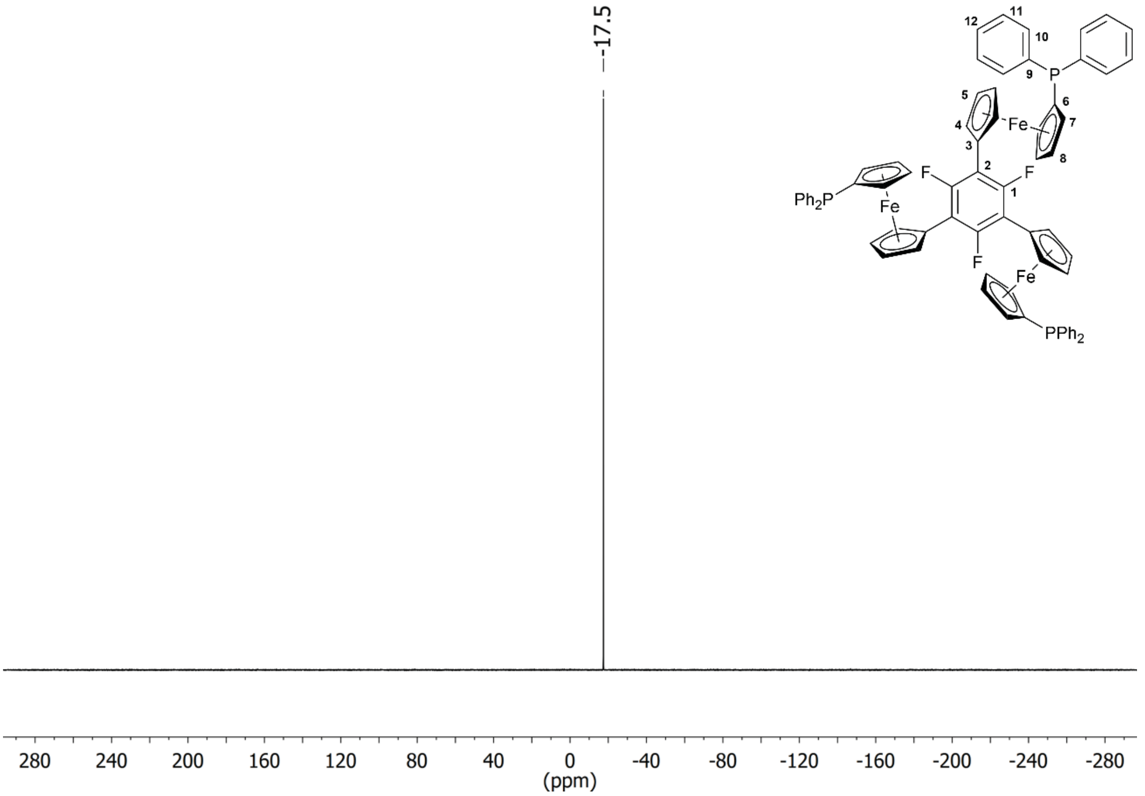

Fig. **S75** $^{31}P\{^1H\}$ NMR spectrum of **1b** in CD_2Cl_2.

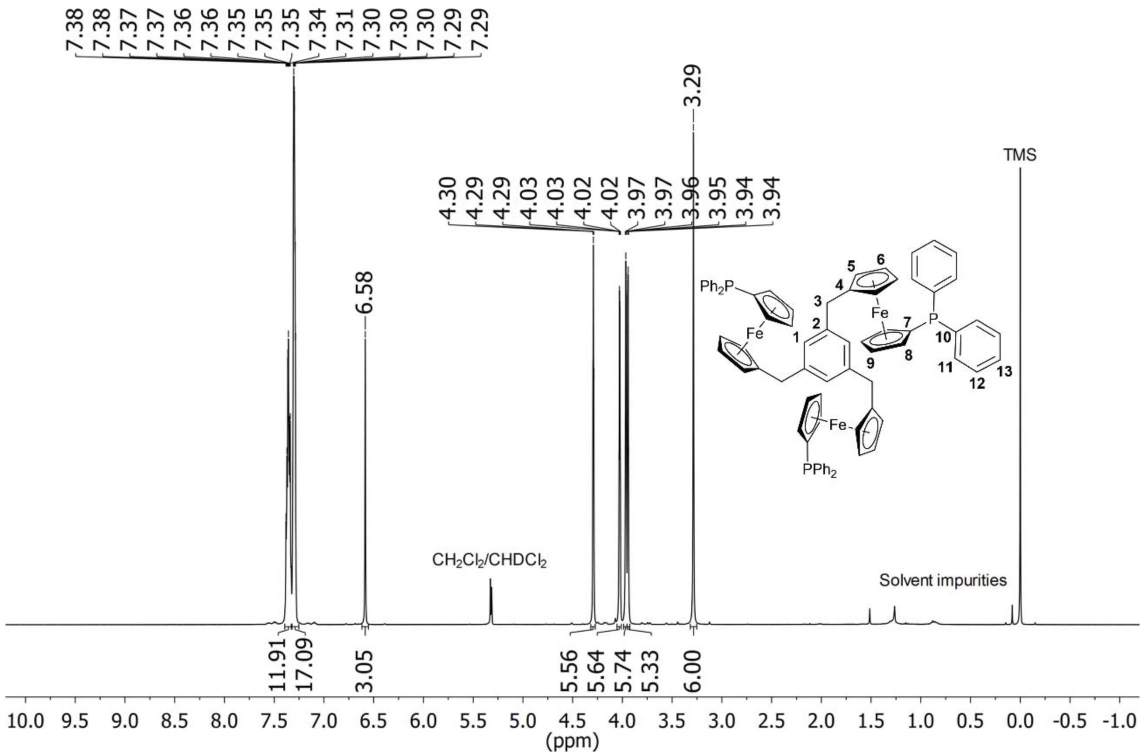

Fig. **S76** ^{1}H NMR spectrum of **1d** in CD_2Cl_2 (Assignment: δ = 7.40–7.33 (H11), 7.33–7.27 (H12+H13), 6.58 (H1), 4.29 (H8/9), 4.03 (H8/9), 3.97 (H5/6), 3.94 (H5/6), 3.29 (H3) ppm).

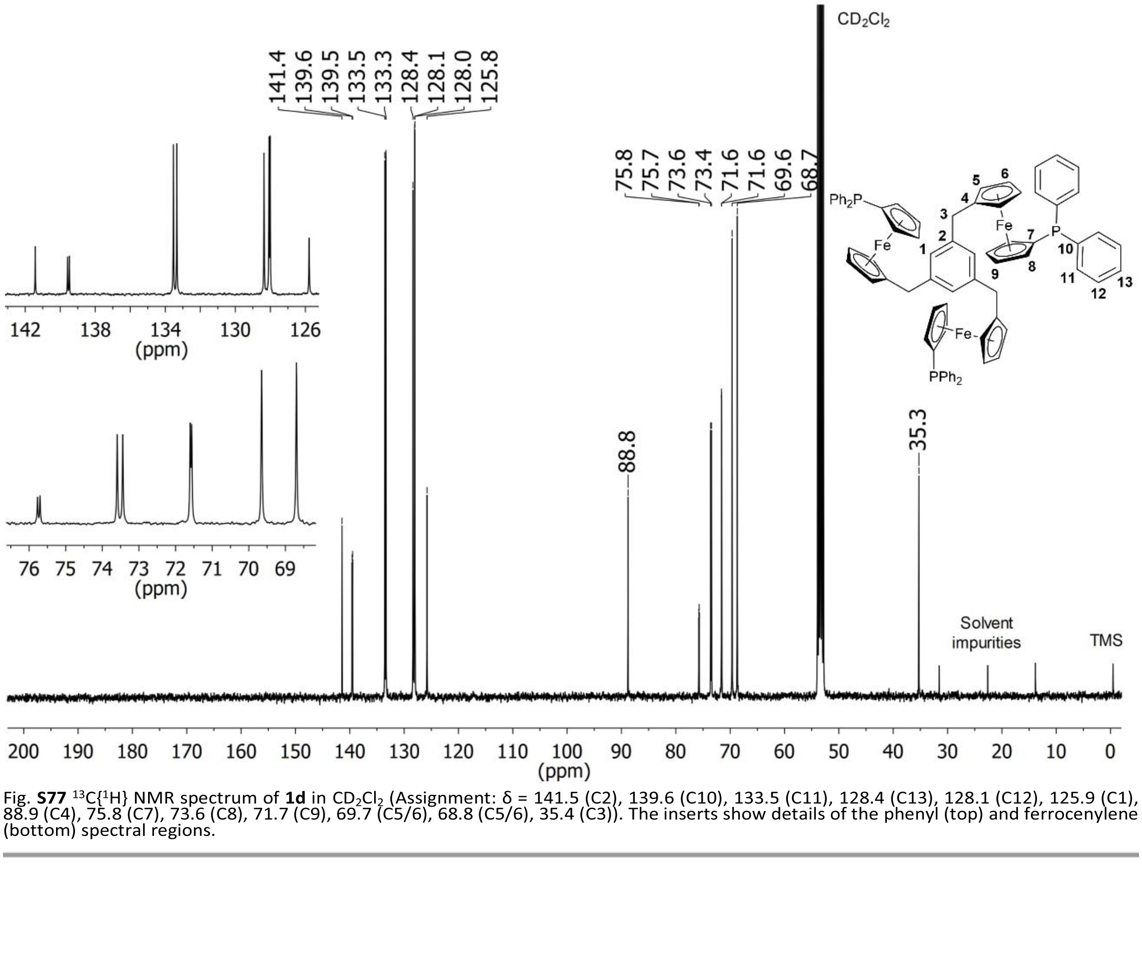

Fig. **S77** $^{13}C\{^{1}H\}$ NMR spectrum of **1d** in CD_2Cl_2 (Assignment: δ = 141.5 (C2), 139.6 (C10), 133.5 (C11), 128.4 (C13), 128.1 (C12), 125.9 (C1), 88.9 (C4), 75.8 (C7), 73.6 (C8), 71.7 (C9), 69.7 (C5/6), 68.8 (C5/6), 35.4 (C3)). The inserts show details of the phenyl (top) and ferrocenylene (bottom) spectral regions.

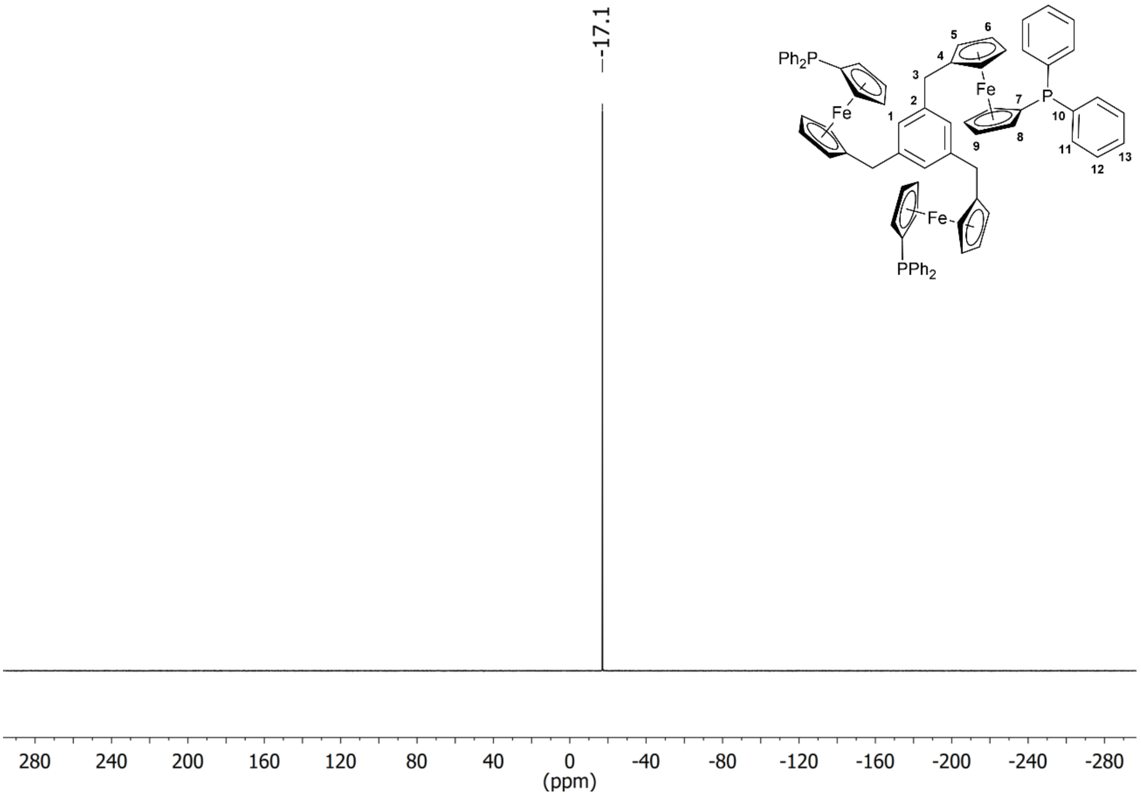

Fig. **S78** ^{31}P{^{1}H} NMR spectrum of **1d** in CD_2Cl_2.

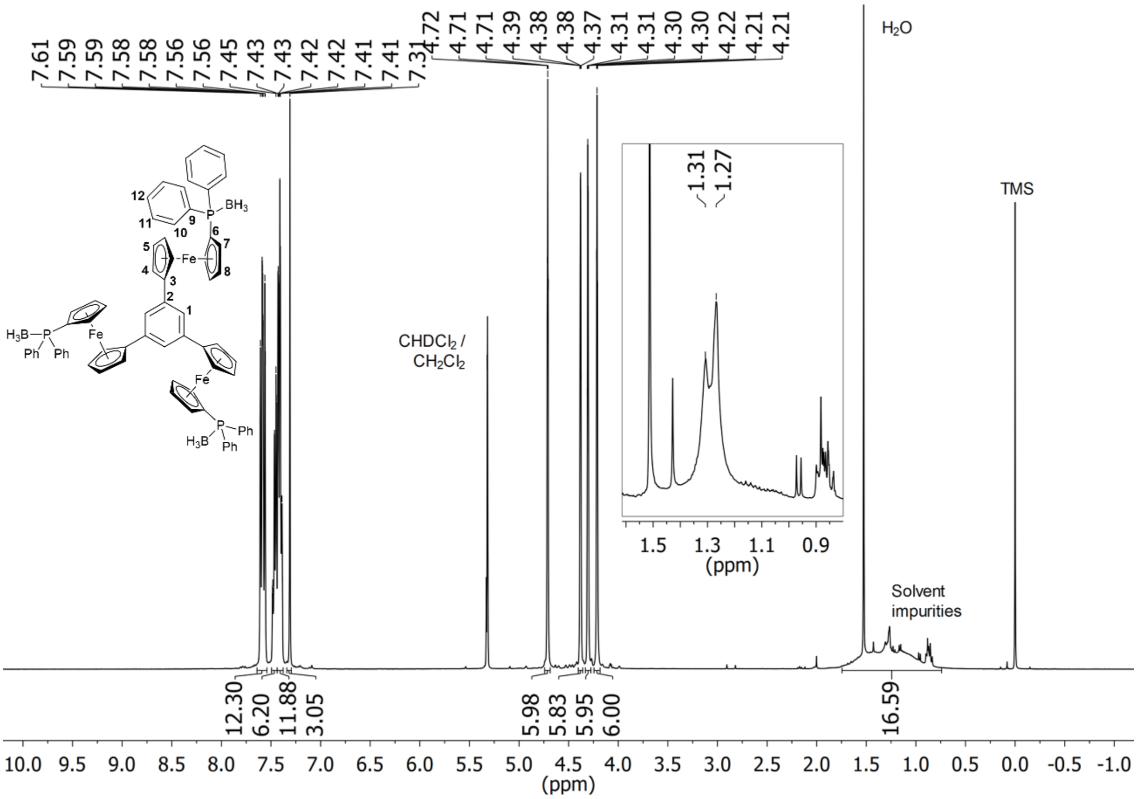

Fig. **S79** ^{1}H NMR spectrum of **[1a(BH$_3$)$_3$]** in CD_2Cl_2 (Assignment: δ = 7.64–7.53 (H10), 7.51–7.44 (H12), 7.44–7.37 (H11), 7.31 (H1), 4.71 (H4), 4.38 (H7), 4.31 (H8), 4.21 (H5), 1.72–0.77 (BH_3) ppm. The integral of the borane region centred around 1.3 pm is overestimated due to NMR solvent impurities. The framed insert shows the corresponding region in a ^{1}H{^{11}B} NMR experiment in the same solvent.

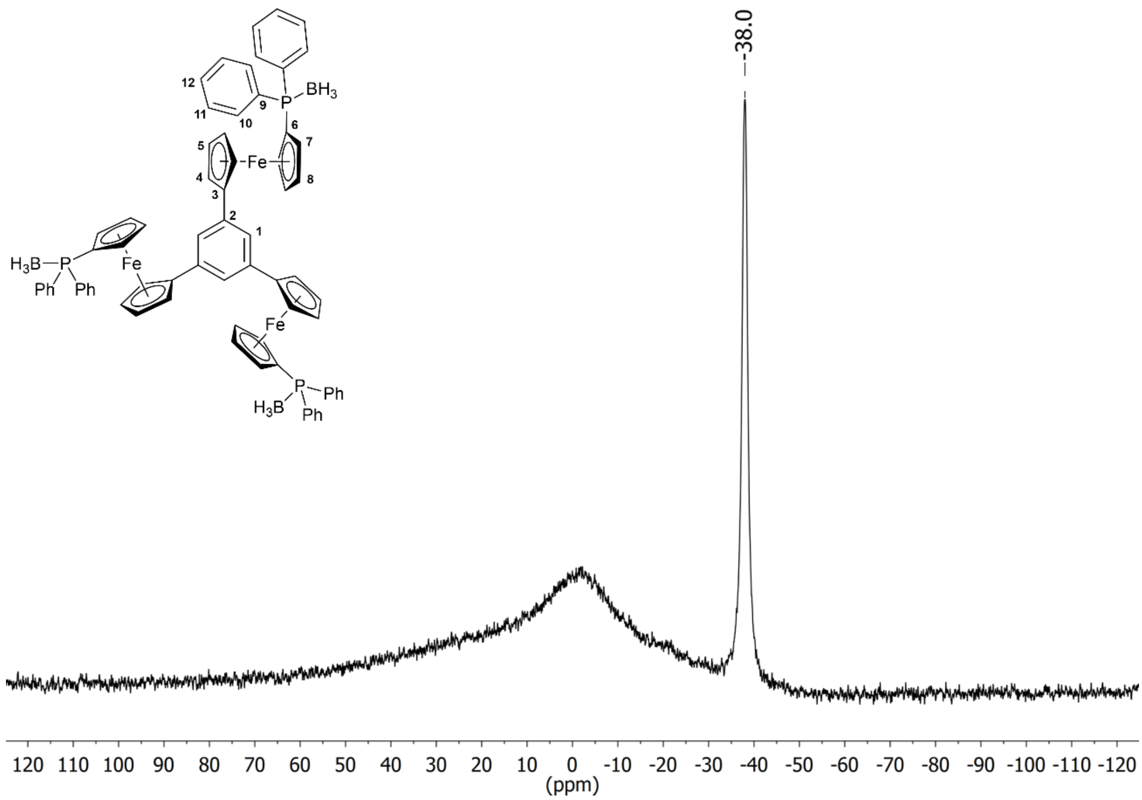

Fig. **S80**. $^{11}B\{^{1}H\}$ NMR spectrum of **[1a(BH$_3$)$_3$]** in CD_2Cl_2. The broad baseline signal originates from the boron-containing components of the NMR tube glass; for the graphic depiction, exponential line broadening (lb = 10 Hz) was applied.

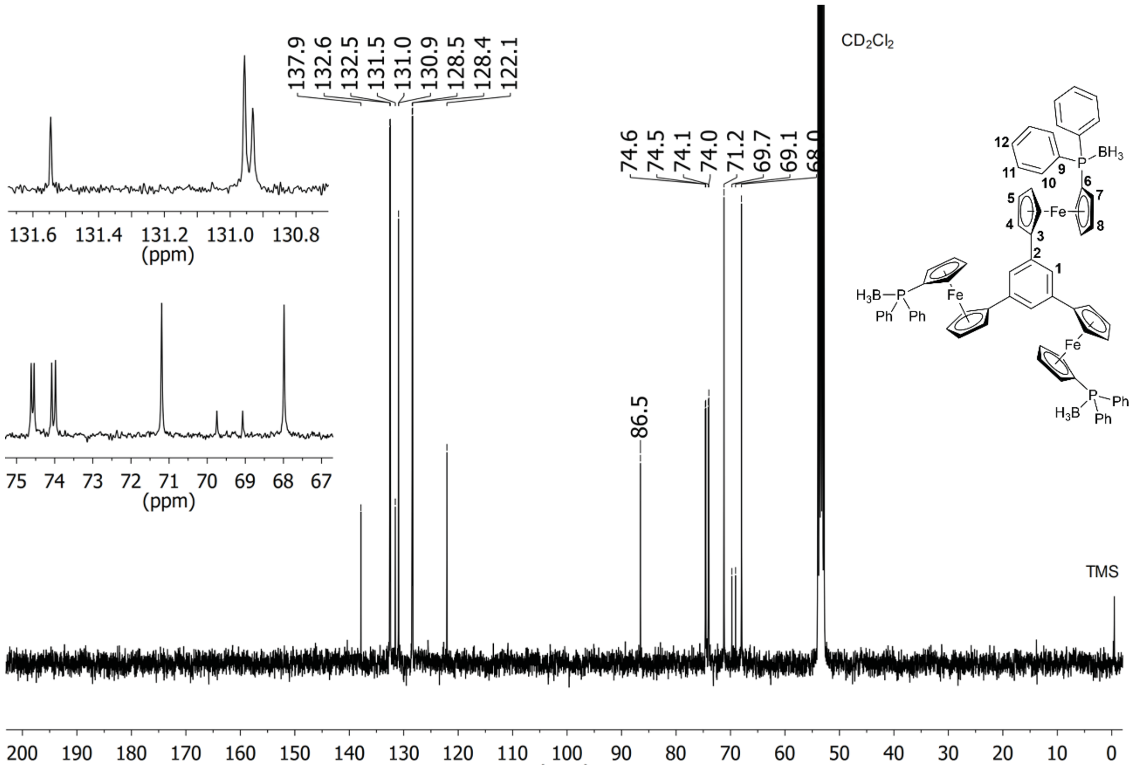

Fig. **S81** $^{13}C\{^{1}H\}$ NMR spectrum of **[1a(BH$_3$)$_3$]** in CD_2Cl_2 (Assignment: δ = 137.9 (C2), 132.5 (C10), 131.2 (C9), 131.0 (C12), 128.4 (C11), 122.1 (C1), 86.5 (C3), 74.6 (C7), 74.0 (C8), 71.2 (C4/5), 69.4 (C6), 68.0 (C4/5) ppm). The inserts show details of the phenyl (top) and ferrocenylene (bottom) spectral regions.

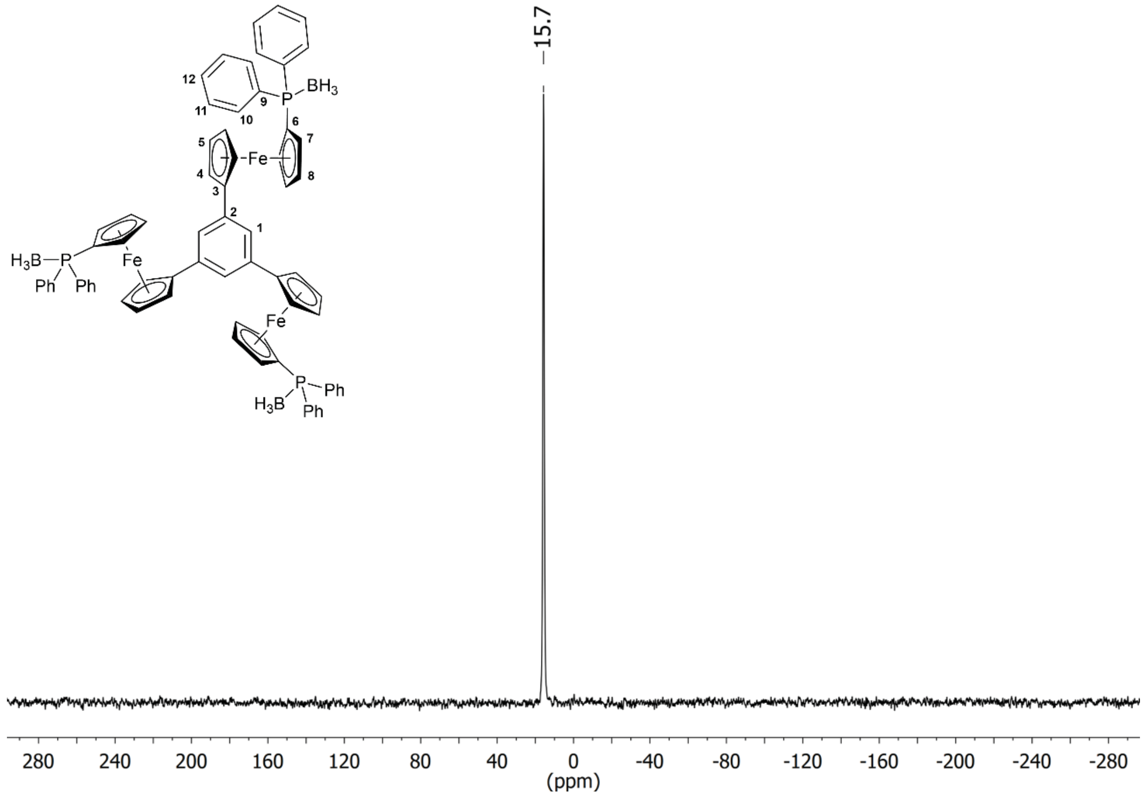

Fig. **S82** $^{31}P\{^{1}H\}$ NMR spectrum of **[1a(BH₃)₃]** in CD_2Cl_2. For the graphic depiction, exponential line broadening (lb = 30 Hz) was applied.

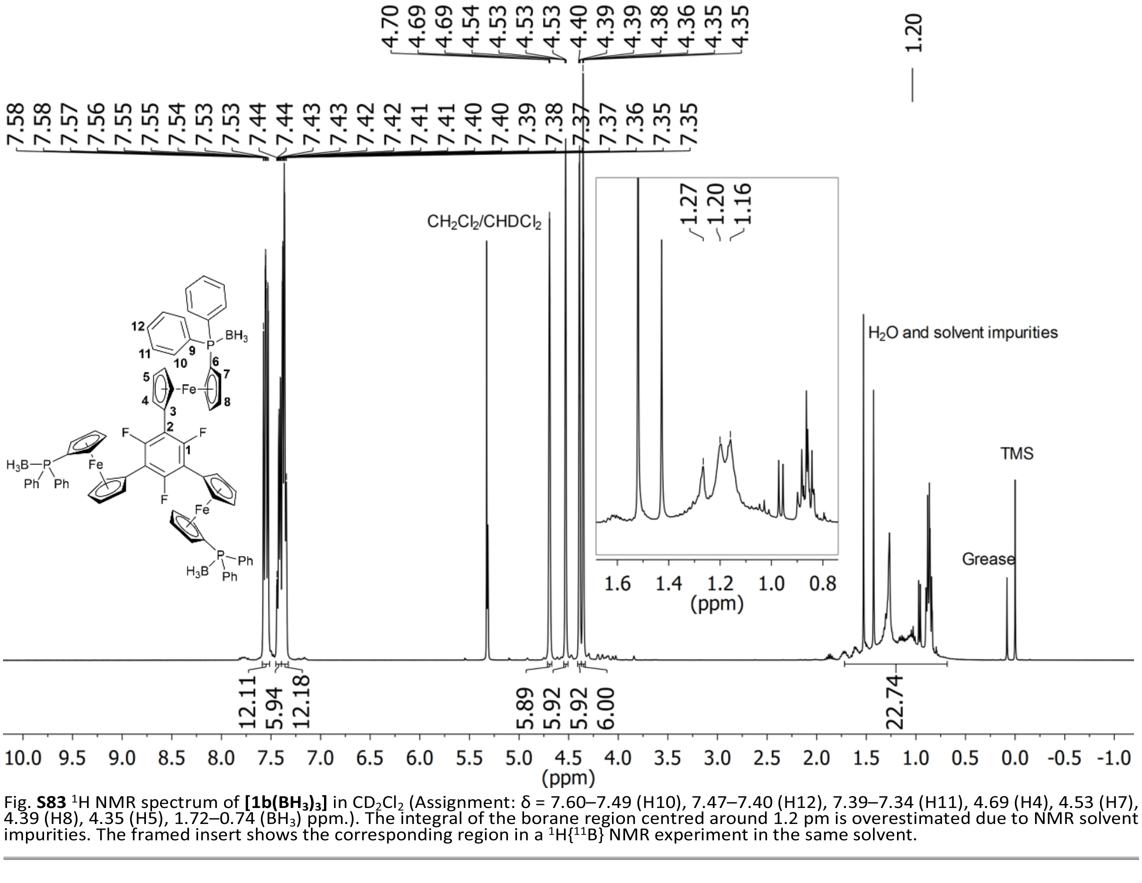

Fig. **S83** ^{1}H NMR spectrum of **[1b(BH₃)₃]** in CD_2Cl_2 (Assignment: δ = 7.60–7.49 (H10), 7.47–7.40 (H12), 7.39–7.34 (H11), 4.69 (H4), 4.53 (H7), 4.39 (H8), 4.35 (H5), 1.72–0.74 (BH_3) ppm.). The integral of the borane region centred around 1.2 pm is overestimated due to NMR solvent impurities. The framed insert shows the corresponding region in a $^{1}H\{^{11}B\}$ NMR experiment in the same solvent.

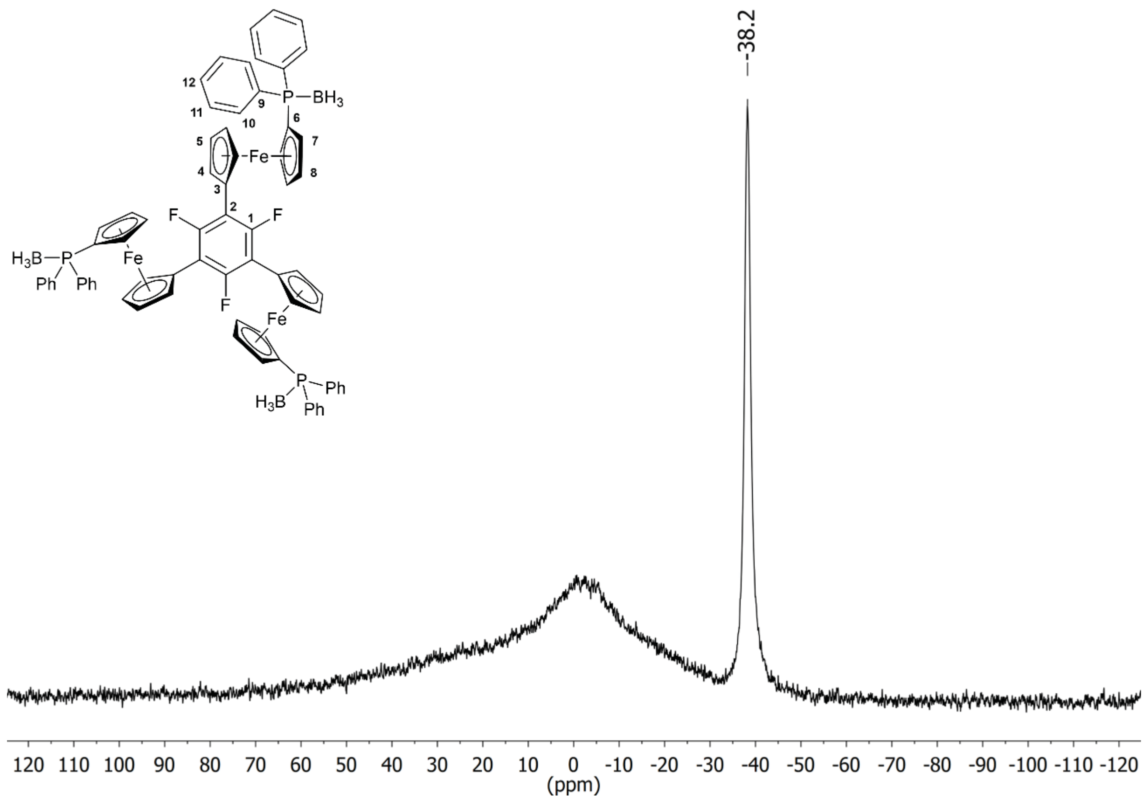

Fig. **S84**. $^{11}B\{^{1}H\}$ NMR spectrum of **[1b(BH$_3$)$_3$]** in CD_2Cl_2. The broad baseline signal originates from the boron-containing components of the NMR tube glass; for the graphic depiction, exponential line broadening (lb = 10 Hz) was applied.

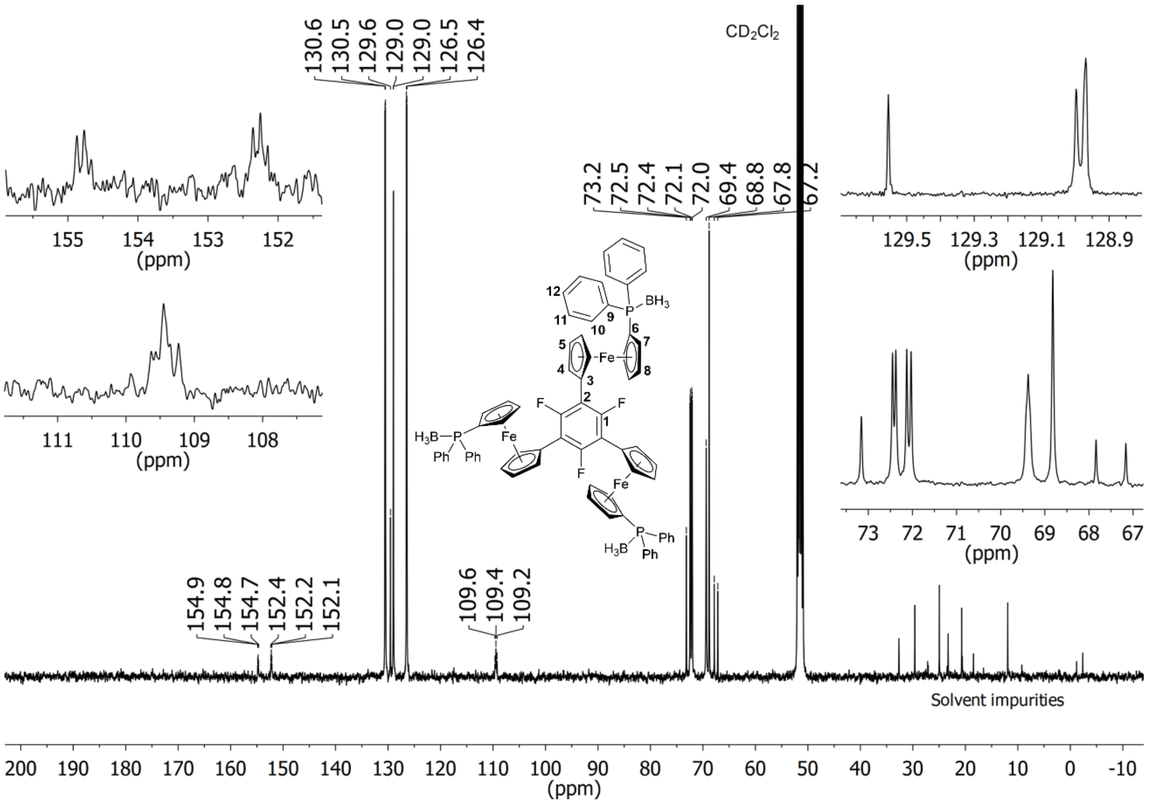

Fig. **S85** $^{13}C\{^{1}H\}$ NMR spectrum of **[1b(BH$_3$)$_3$]** in CD_2Cl_2 (Assignment: δ = 153.5 (C1), 130.5 (C10), 129.3 (C9), 129.0 (C12), 126.4 (C11), 109.5 (C2), 73.1 (C3), 72.4 (C7), 72.1 (C8), 69.4 (C4), 68.8 (C5), 67.5 (C6) ppm). The inserts show details of the phenyl (top right) and ferrocenylene (bottom right) spectral regions as well as magnifications of the *F*-bound arene carbon atoms (left).

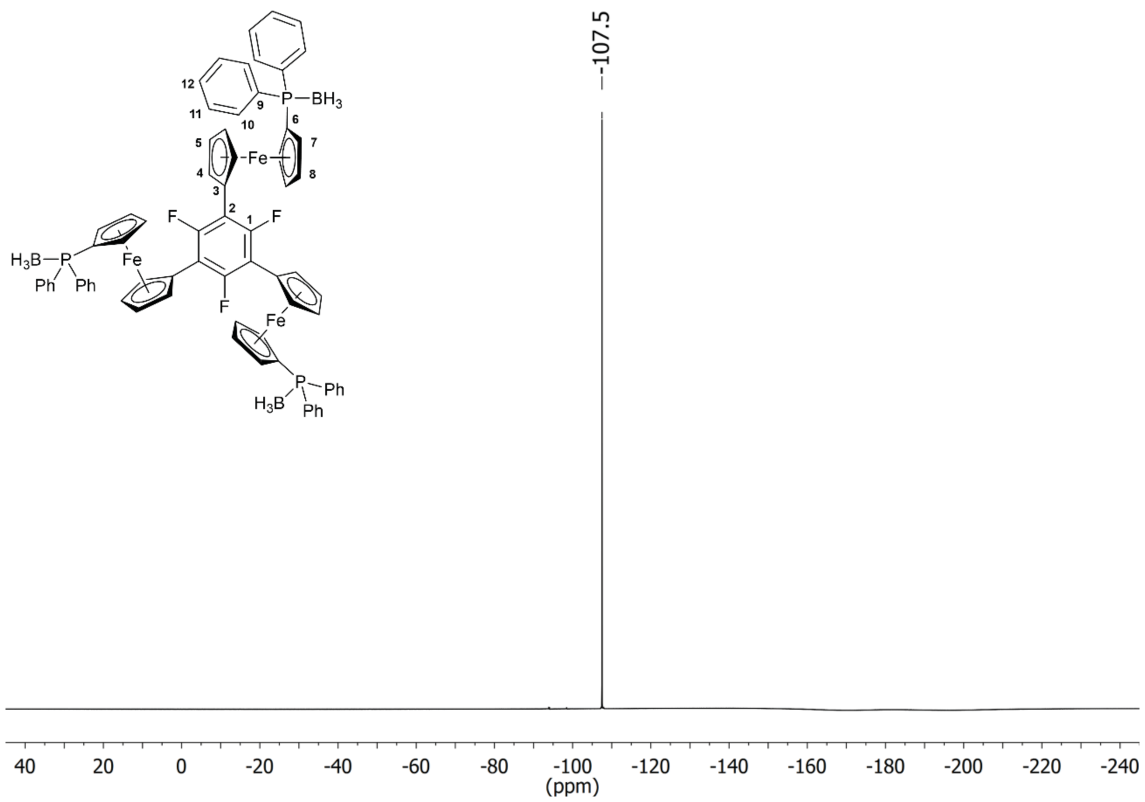

Fig. **S86** $^{19}F\{^1H\}$ NMR spectrum of **[1b(BH₃)₃]** in CD_2Cl_2.

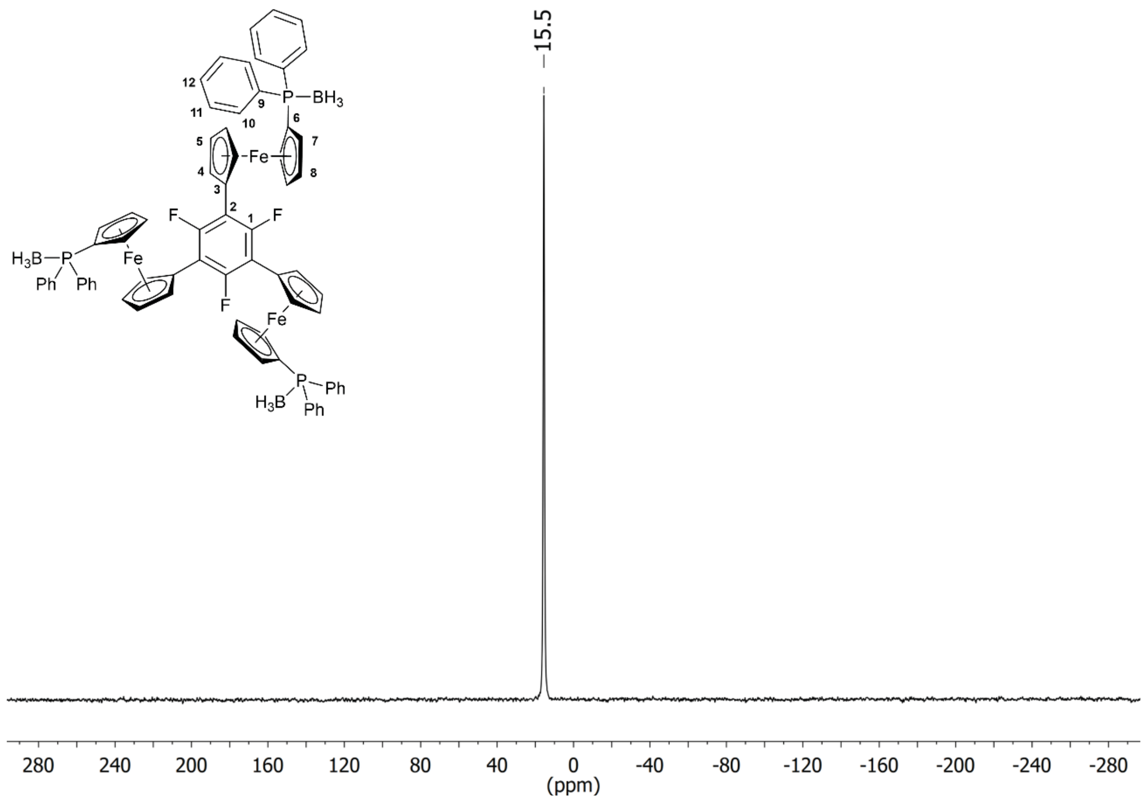

Fig. **S87** $^{31}P\{^1H\}$ NMR spectrum of **[1b(BH$_3$)$_3$]** in CD_2Cl_2. For the graphic depiction, exponential line broadening (lb = 30 Hz) was applied.

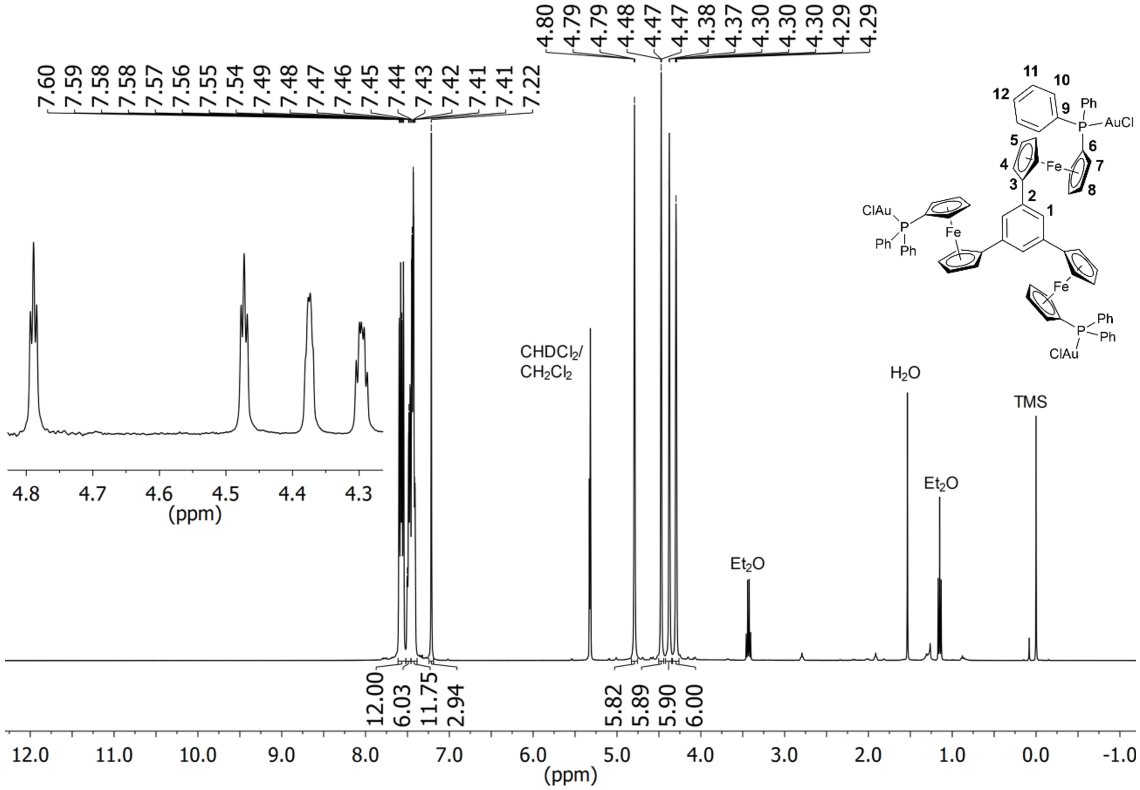

Fig. **S88** ^{1}H NMR spectrum of **[1a(Au)$_3$]** in CD_2Cl_2 (Assignment: δ = 7.62–7.53 (H11), 7.51–7.46 (H12), 7.46–7.39 (H10), 7.22 (H1), 4.79 (H4), 4.47 (H5), 4.38 (H7), 4.30 (H8) ppm). The insert shows the ferrocenylene proton NMR signals in detail.

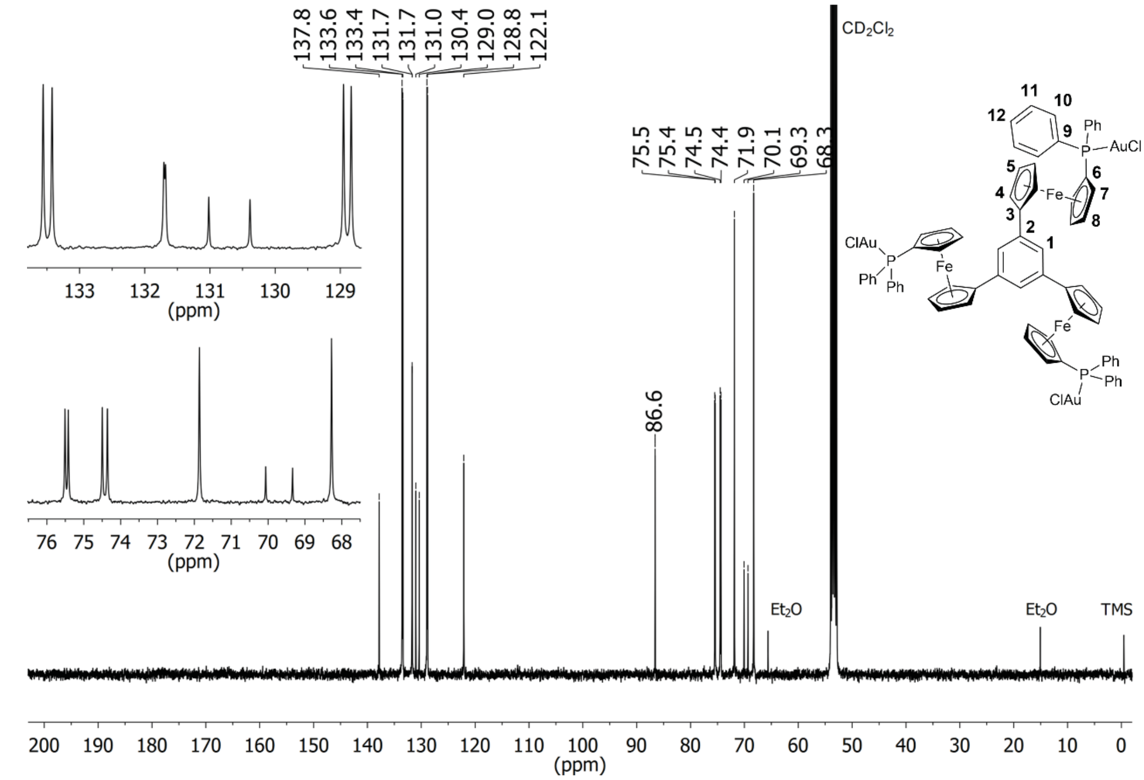

Fig. **S89**. $^{13}C\{^1H\}$ NMR spectrum of **[1a(Au)$_3$]** in CD_2Cl_2 (Assignment: δ = 137.8 (C2), 133.5 (C11), 131.7 (C12), 130.7 (C9), 128.9 (C10), 122.1 (C1), 86.6 (C3), 75.5 (C7), 74.4 (C8), 71.9 (C4/5), 69.7 (C6), 68.3 (C4/5) ppm) The inserts show details of the phenyl (top left) and ferrocenylene (bottom left) spectral regions.

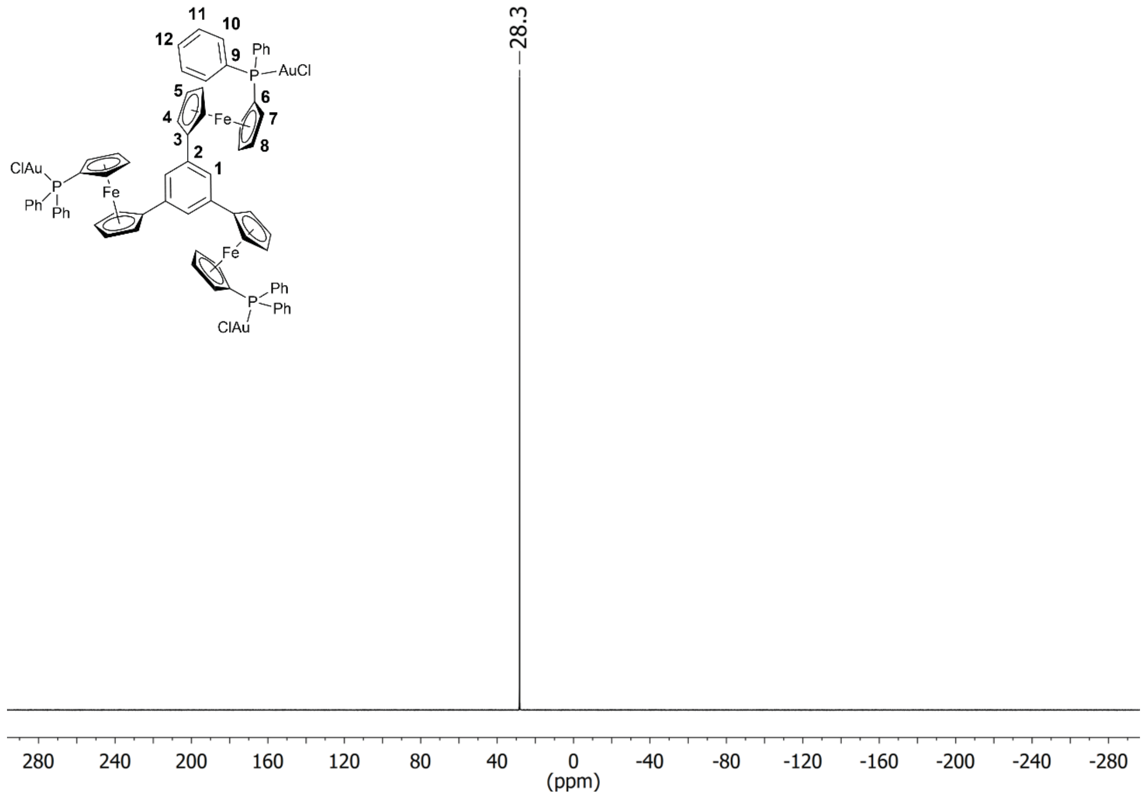

Fig. **S90** $^{31}P\{^{1}H\}$ NMR spectrum of **[1a(Au)$_3$]** in CD_2Cl_2.

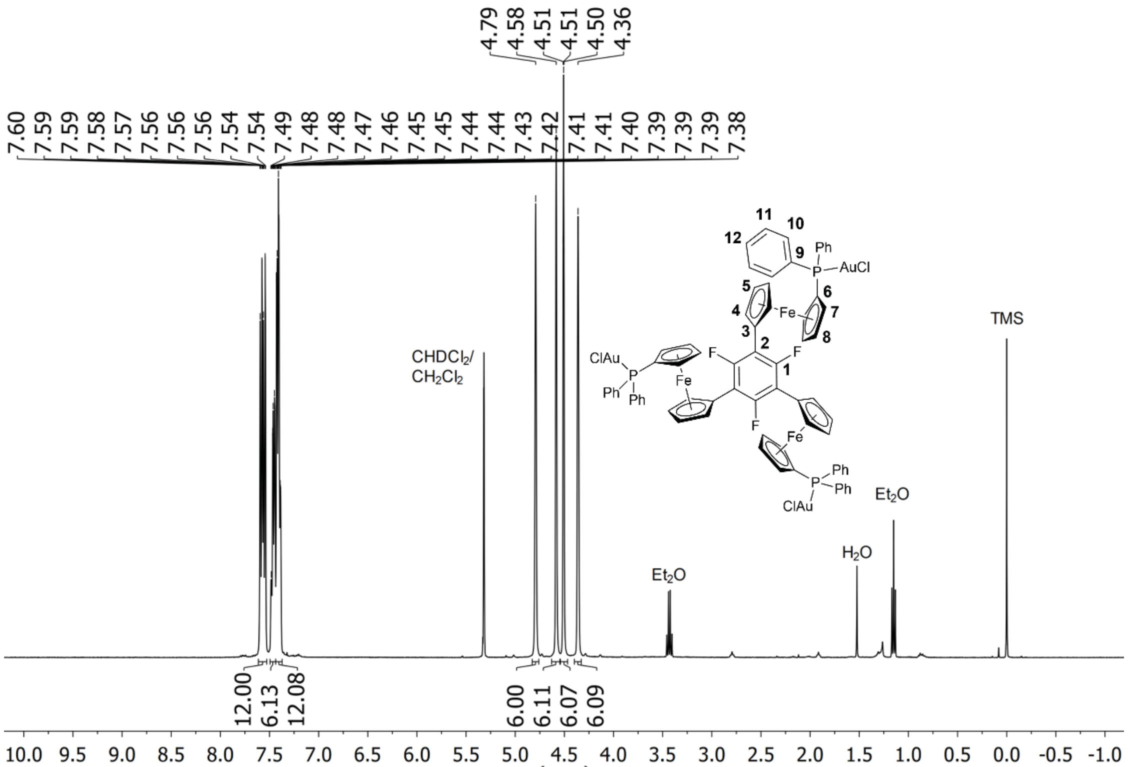

Fig. **S91** ^{1}H NMR spectrum of **[1b(Au)$_3$]** in CD_2Cl_2 (Assignment: δ = 7.61–7.53 (H11), 7.51–7.44 (H12), 7.44–7.33 (H10), 4.79 (H4), 4.58 (H7/8), 4.51 (H5), 4.36 (H7/8) ppm).

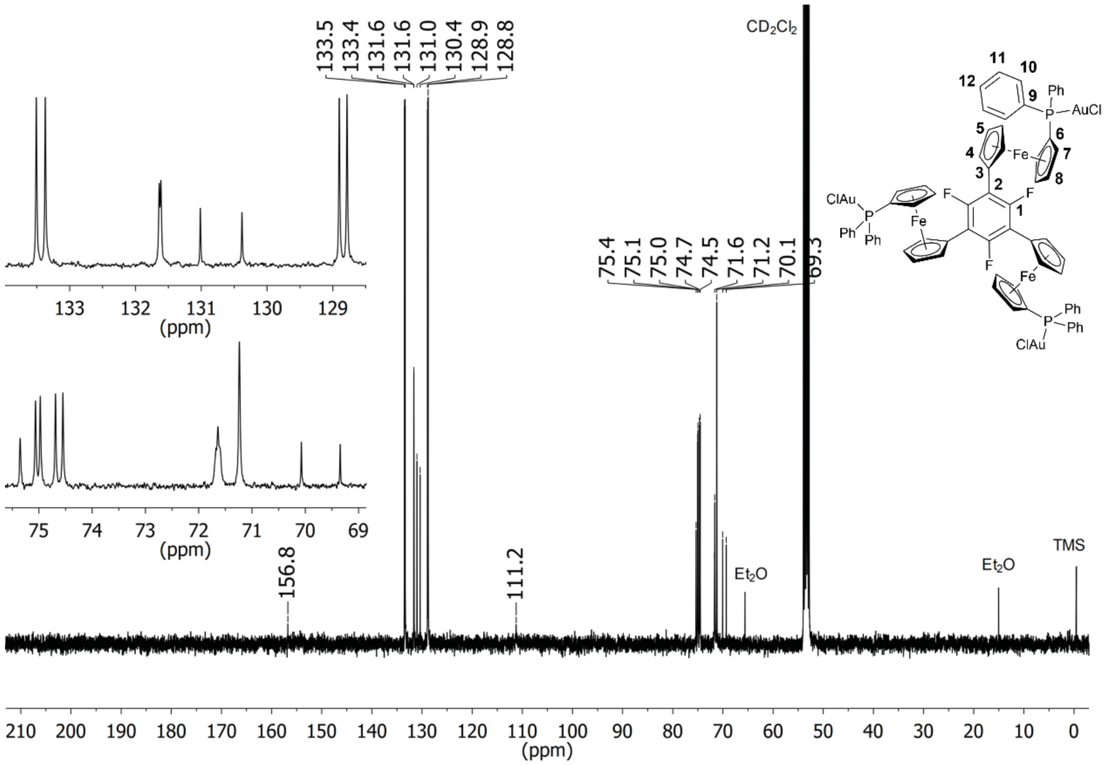

Fig. **S92** $^{13}C\{^{1}H\}$ NMR spectrum of **[1b(Au)$_3$]** in CD_2Cl_2 (Assignment: δ = 156.8 (C1), 133.4 (C11), 131.6 (C12), 130.7 (C9), 128.8 (C10), 111.2 (C2), 75.4 (C3), 75.0 (C7), 74.6 (C8), 71.6 (C4), 71.2 (C5), 69.7 (C6) ppm). The inserts show details of the phenyl (top left) and ferrocenylene (bottom left) spectral regions.

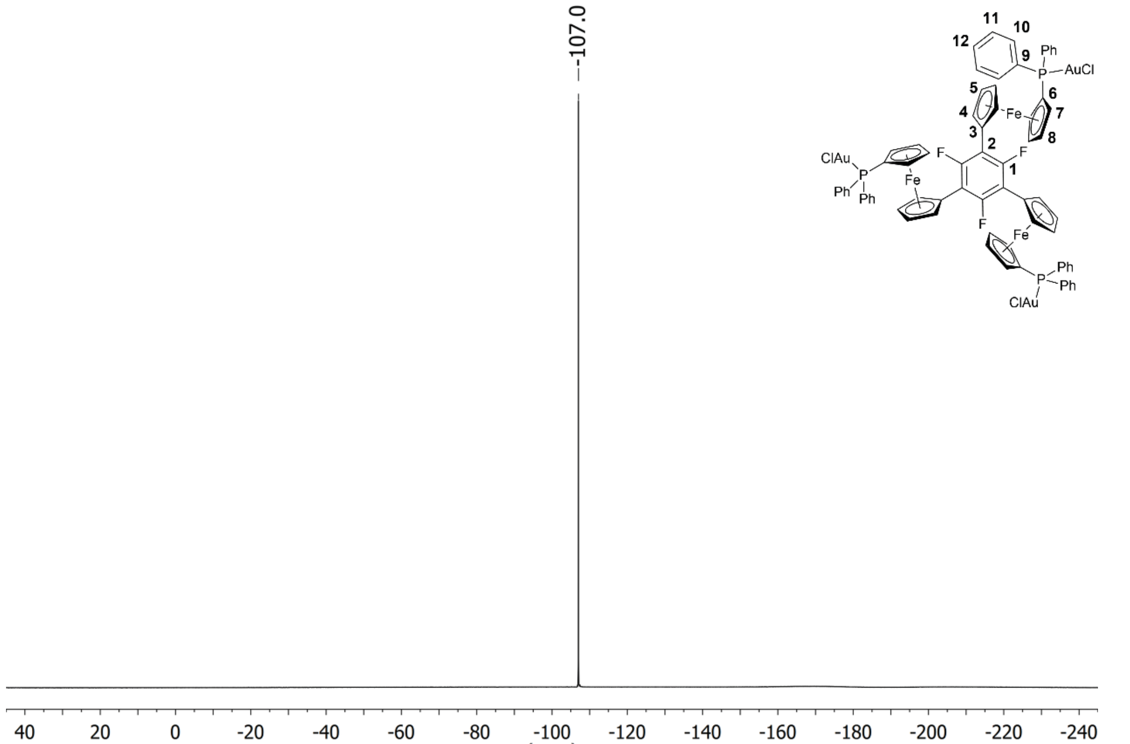

Fig. **S93**. $^{19}F\{^{1}H\}$ NMR spectrum of **[1b(Au)$_3$]** in CD_2Cl_2.

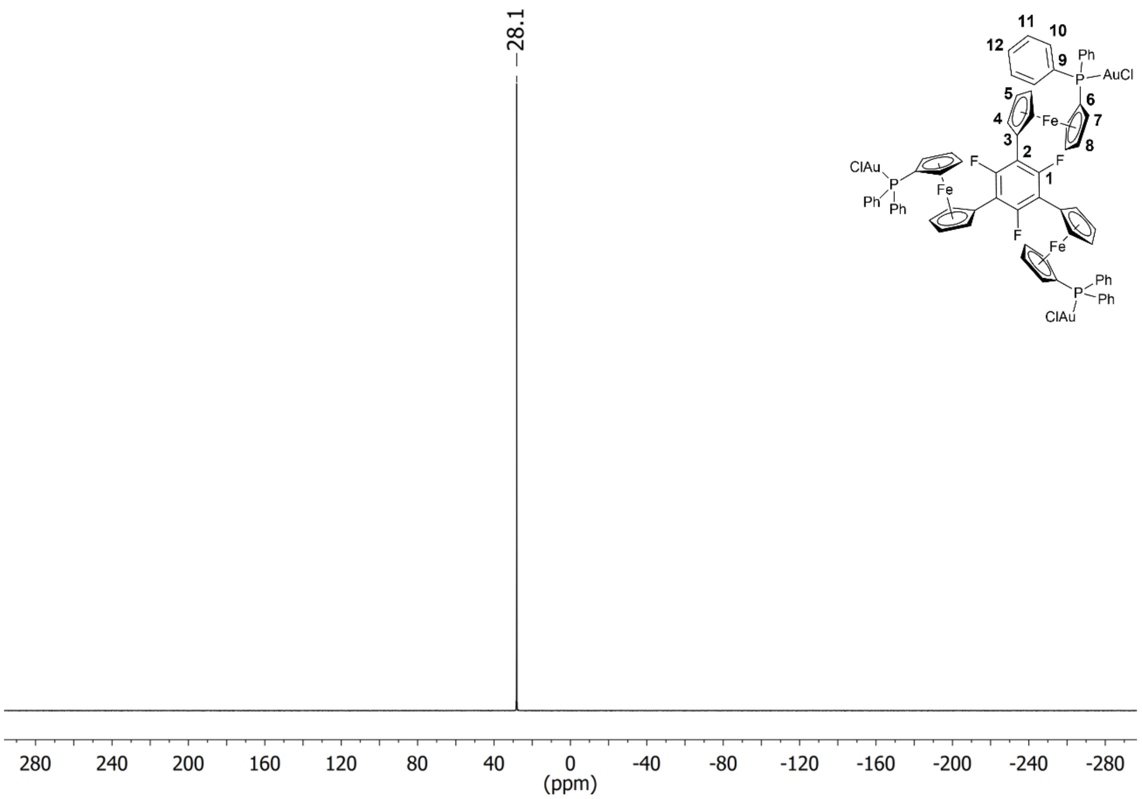

Fig. **S94** $^{31}P\{^{1}H\}$ NMR spectrum of **[1b(Au)$_3$]** in CD_2Cl_2.

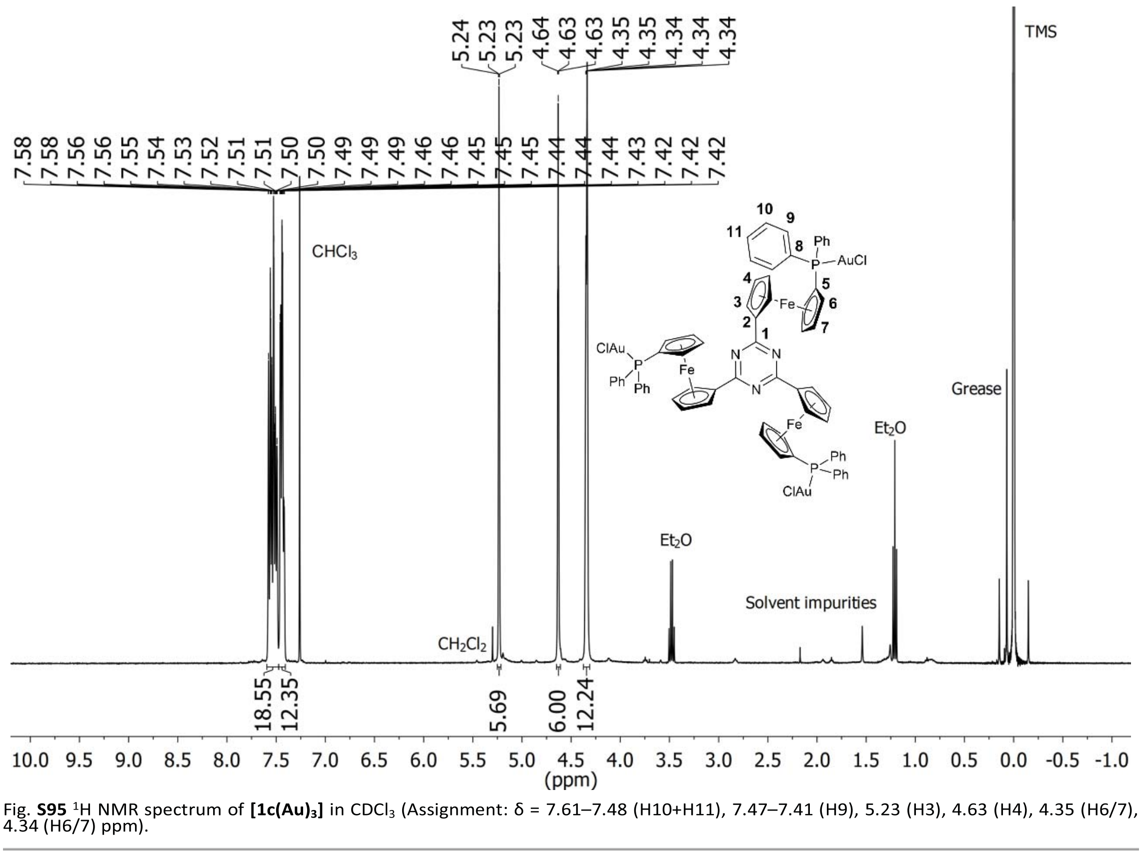

Fig. **S95** ^{1}H NMR spectrum of **[1c(Au)$_3$]** in $CDCl_3$ (Assignment: δ = 7.61–7.48 (H10+H11), 7.47–7.41 (H9), 5.23 (H3), 4.63 (H4), 4.35 (H6/7), 4.34 (H6/7) ppm).

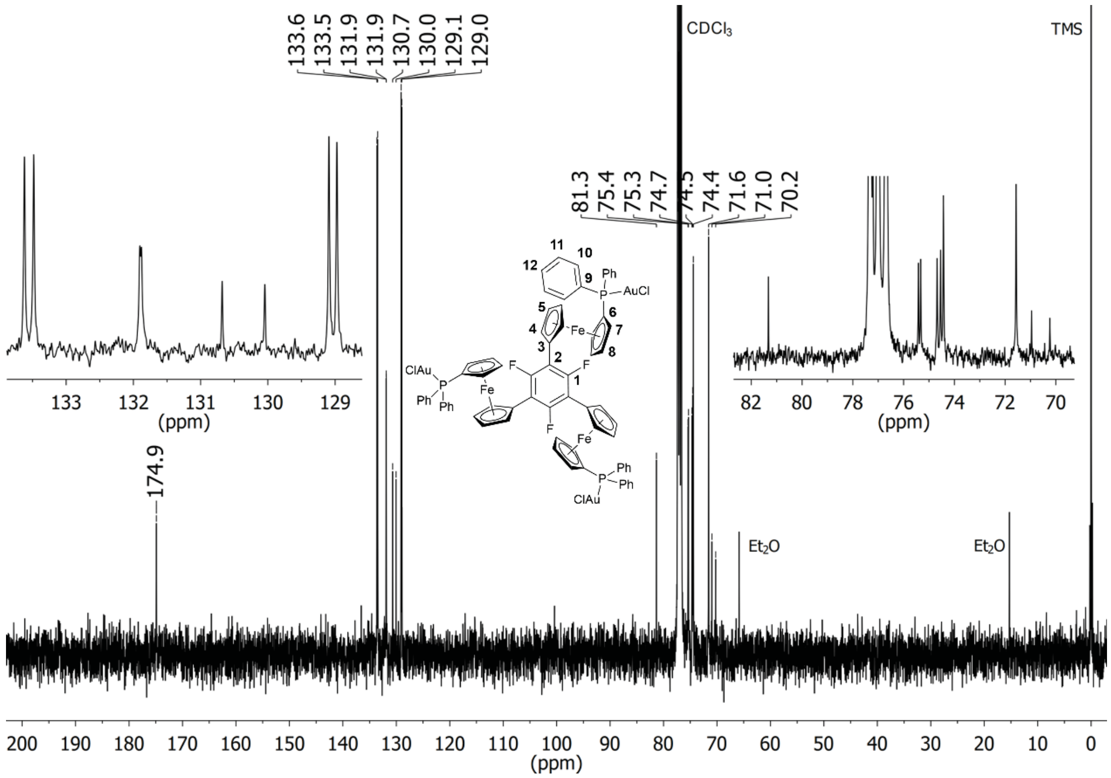

Fig. **S96** $^{13}C\{^{1}H\}$ NMR spectrum of **[1c(Au)$_3$]** in $CDCl_3$ (Assignment: δ = 174.9 (C1), 133.6 (C10), 131.9 (C11), 130.4 (C8), 129.0 (C3), 81.3 (C2), 75.4 (C6), 74.6 (C7), 74.4 (C3/4), 71.6 (C3/4), 70.6 (C5) ppm The inserts show details of the phenyl (left) and ferrocenylene (right) spectral regions.

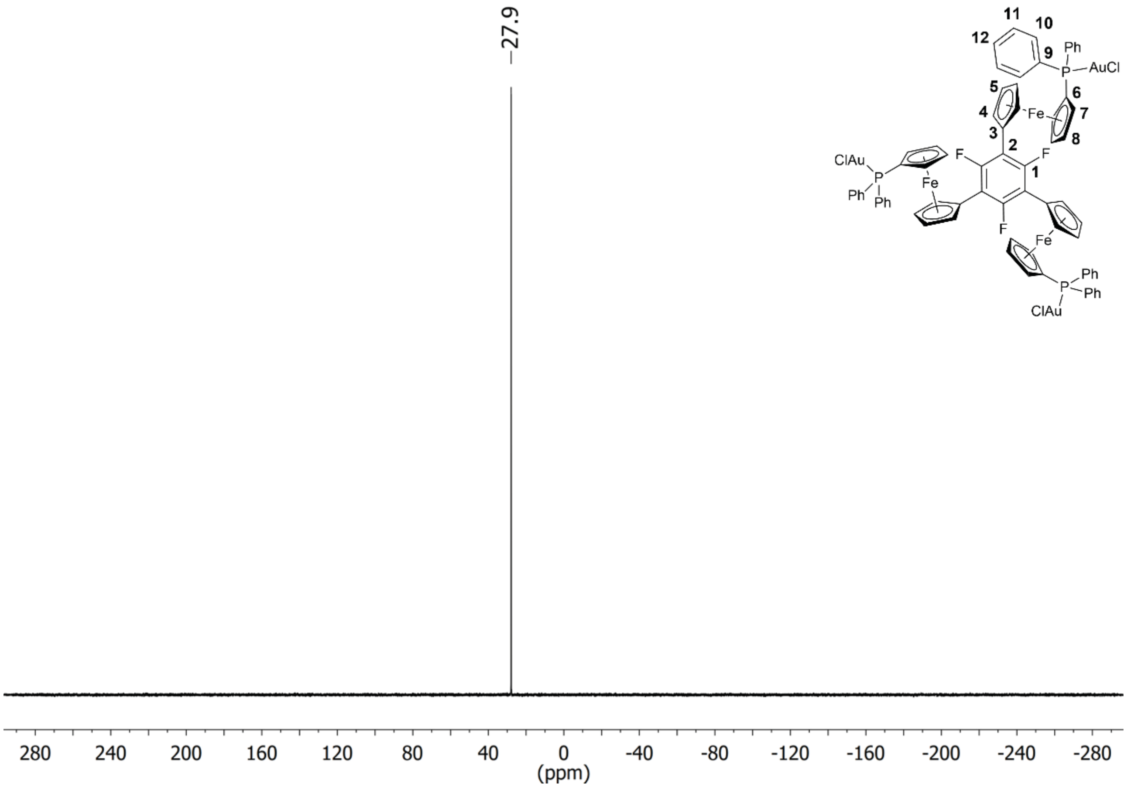

Fig. **S97** $^{31}P\{^{1}H\}$ NMR spectrum of **[1c(Au)$_3$]** in $CDCl_3$.

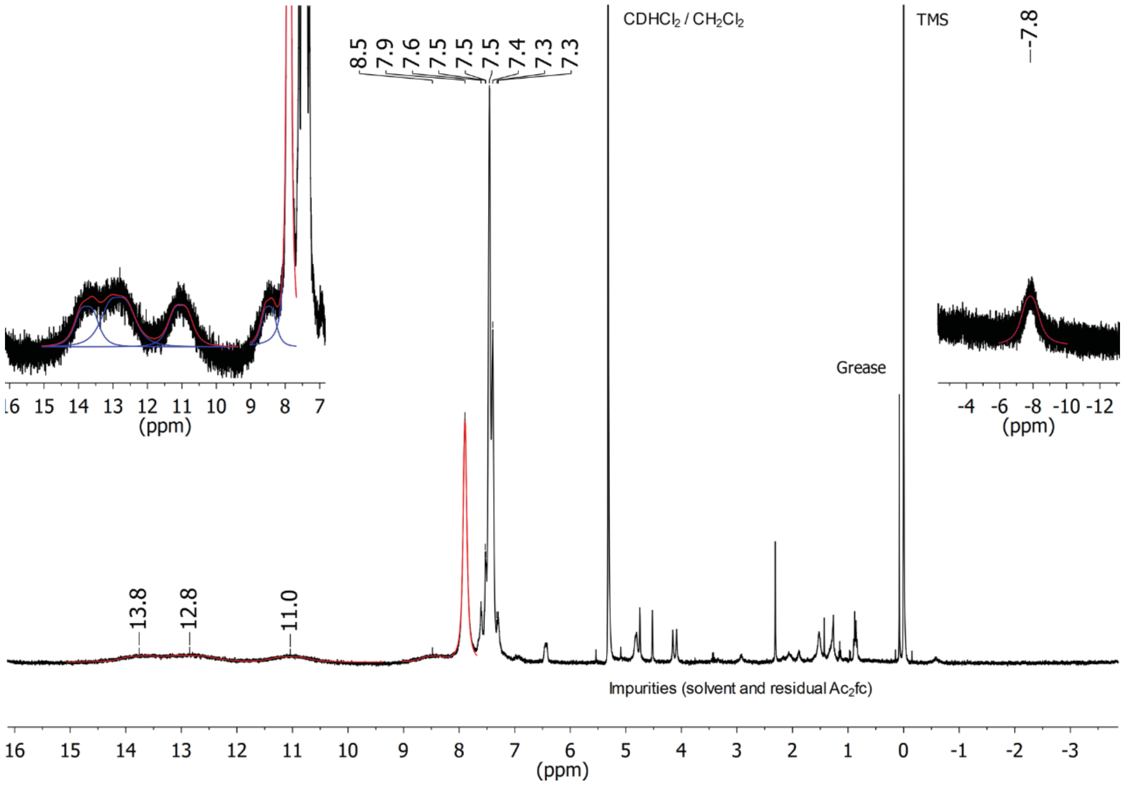

Fig. **S98** ^{1}H NMR spectrum of **[1a(Au)$_3$](TEF)** in CD_2Cl_2. The inserts show the relevant far-positive and far-negative spectral regions containing broad, most likely ferrocenium-centred resonances which have been fitted using the line-fitting tool implemented in MestReNova (red: sum, blue: individual peak contribution). Integration is not reliably possible for these spectra.

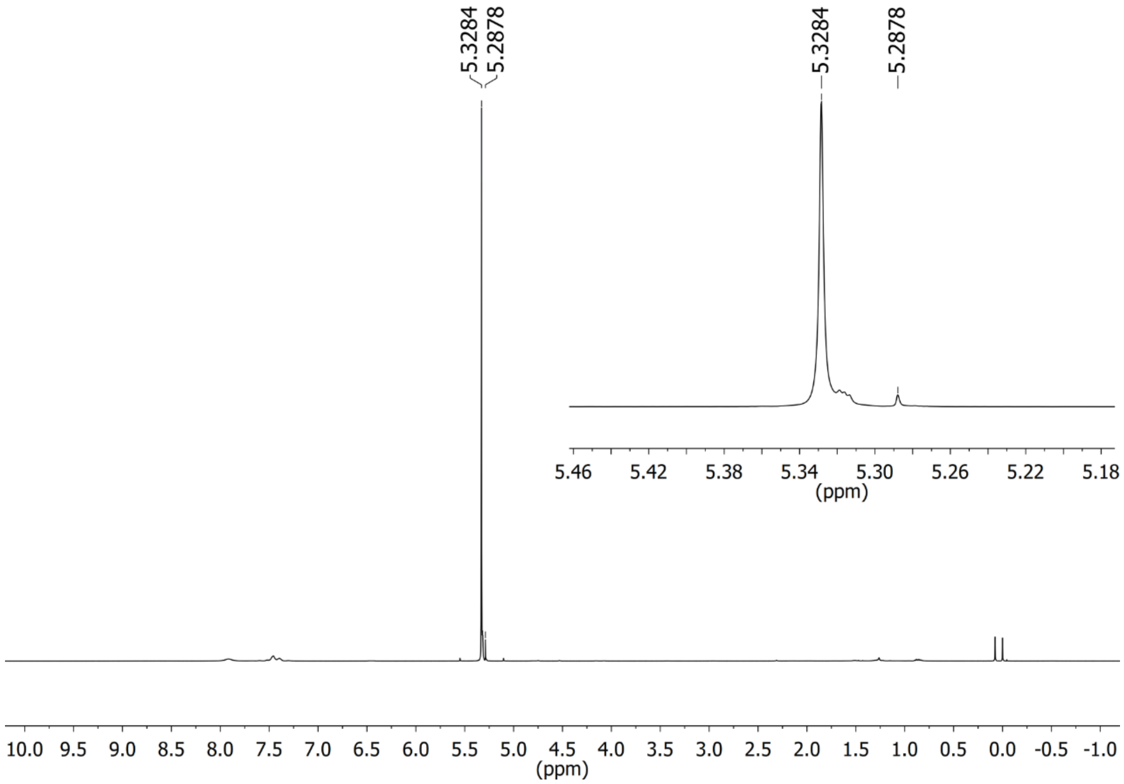

Fig. **S99** Evans' (^{1}H) NMR spectrum for determination of effective magnetic moment of **[1a(Au)$_3$](TEF)** (4.74 mmol·L^{-1}, 25 °C) in CD_2Cl_2/CH_2Cl_2 50:1 v/v. The insert shows the shift between CH_2Cl_2 (sample) and CH_2Cl_2 (reference).

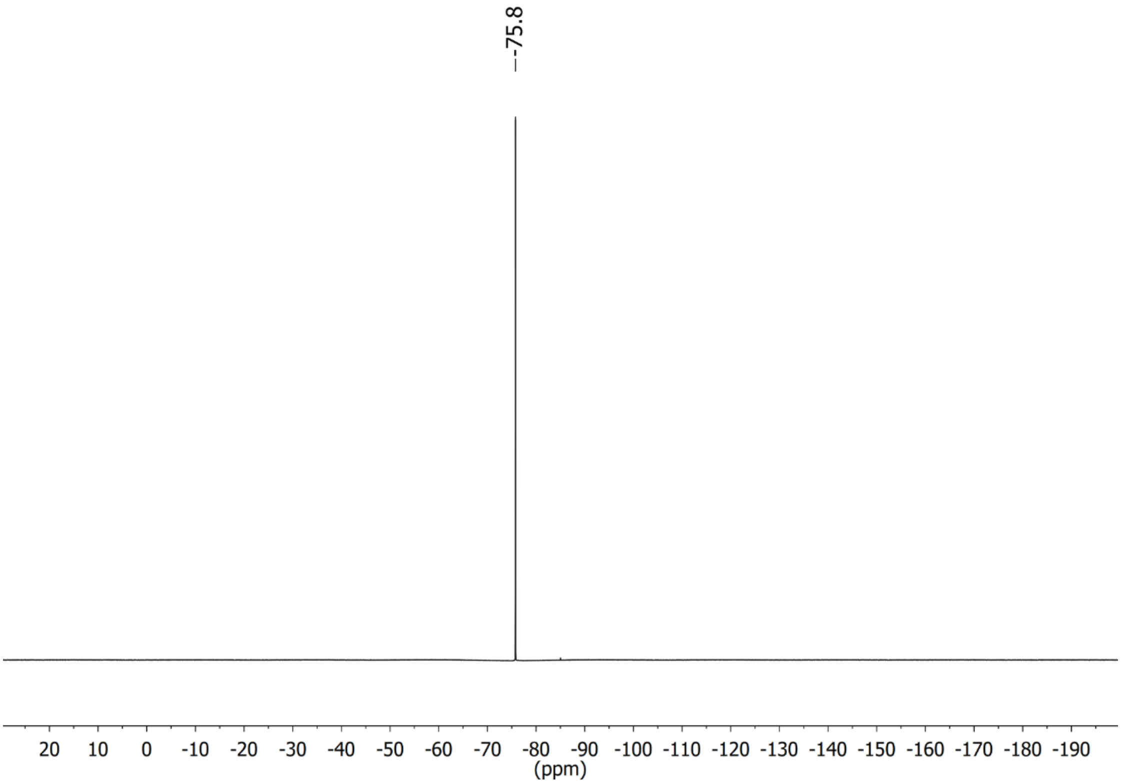

Fig. **S100** ^{19}F NMR spectrum of **[1a(Au)$_3$](TEF)** in CD_2Cl_2.

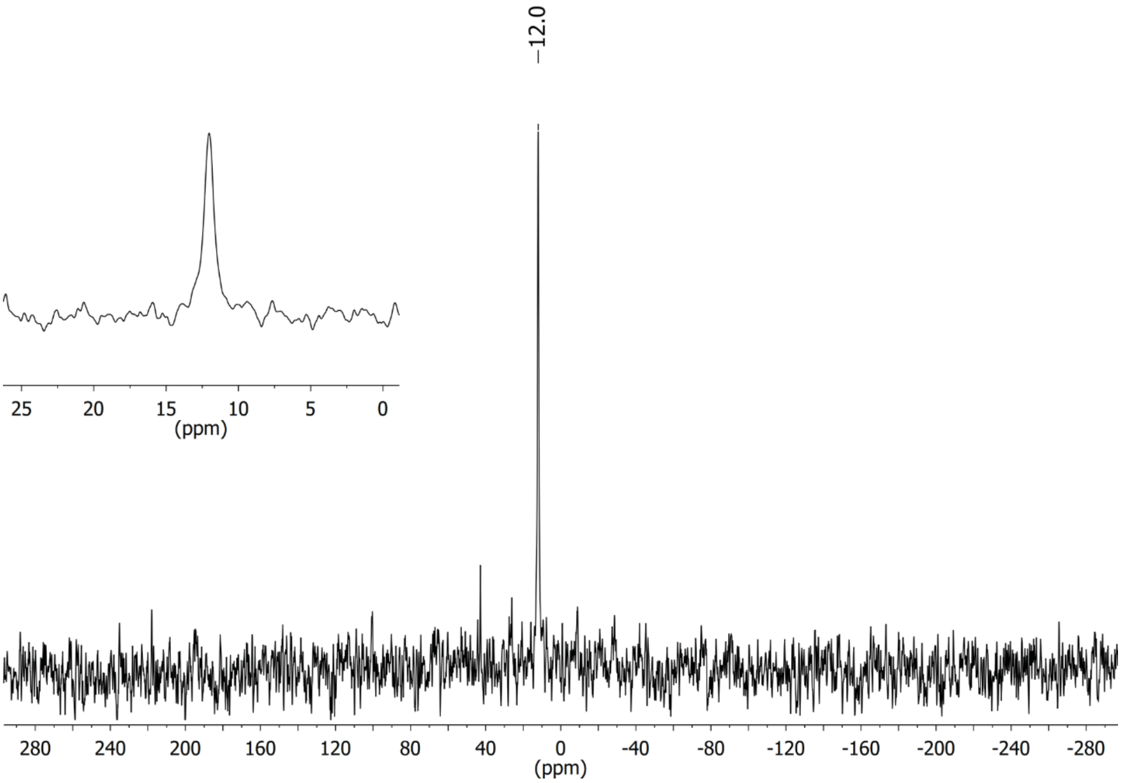

Fig. **S101** $^{31}P\{^1H\}$ NMR spectrum of **[1a(Au)$_3$](TEF)** in CD_2Cl_2, determined using the $z_g p_g$ pulse sequence for fast-relaxing nuclei. For the graphical representation, an experimental line broadening of 50 Hz has been used. The insert shows the relevant signal in detail.

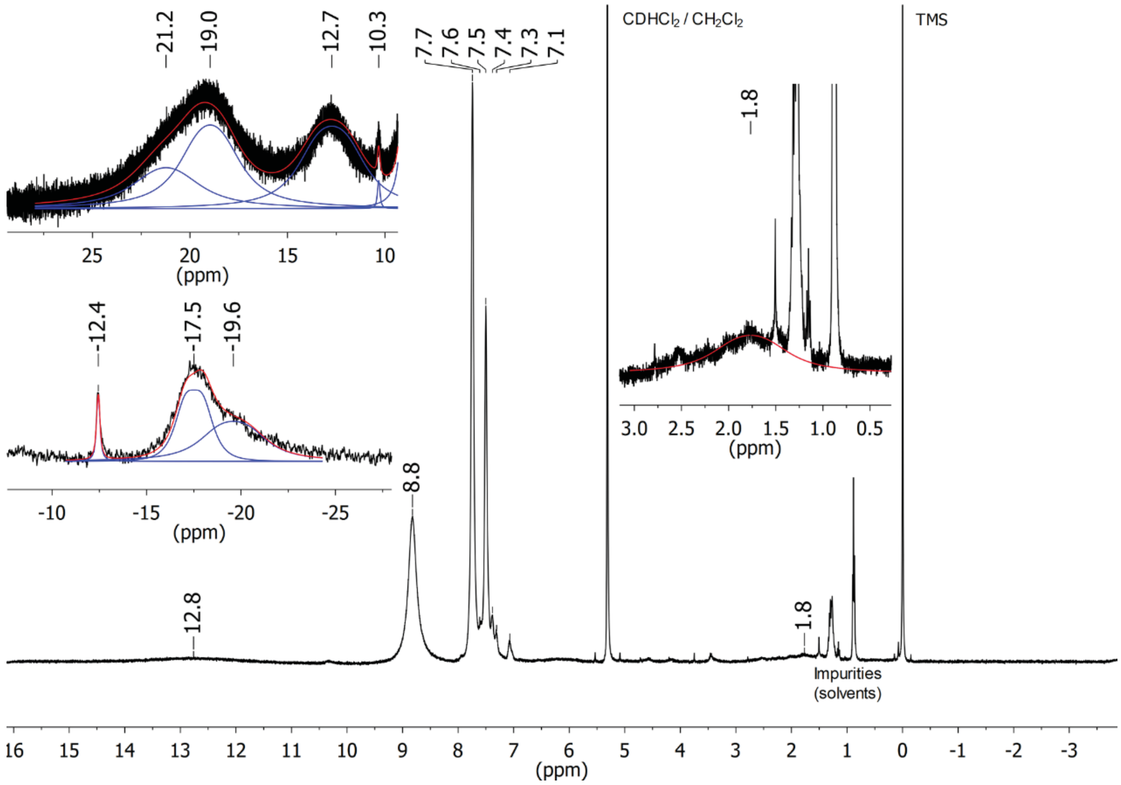

Fig. **S102** ^{1}H NMR spectrum of **[1a(Au)$_3$](TEF)$_2$** in CD_2Cl_2. The inserts show the relevant spectral regions containing broad, most likely ferrocenium-centred resonances which have been fitted using the line-fitting tool implemented in MestReNova (red: sum, blue: individual peak contribution). Integration is not reliably possible for these spectra.

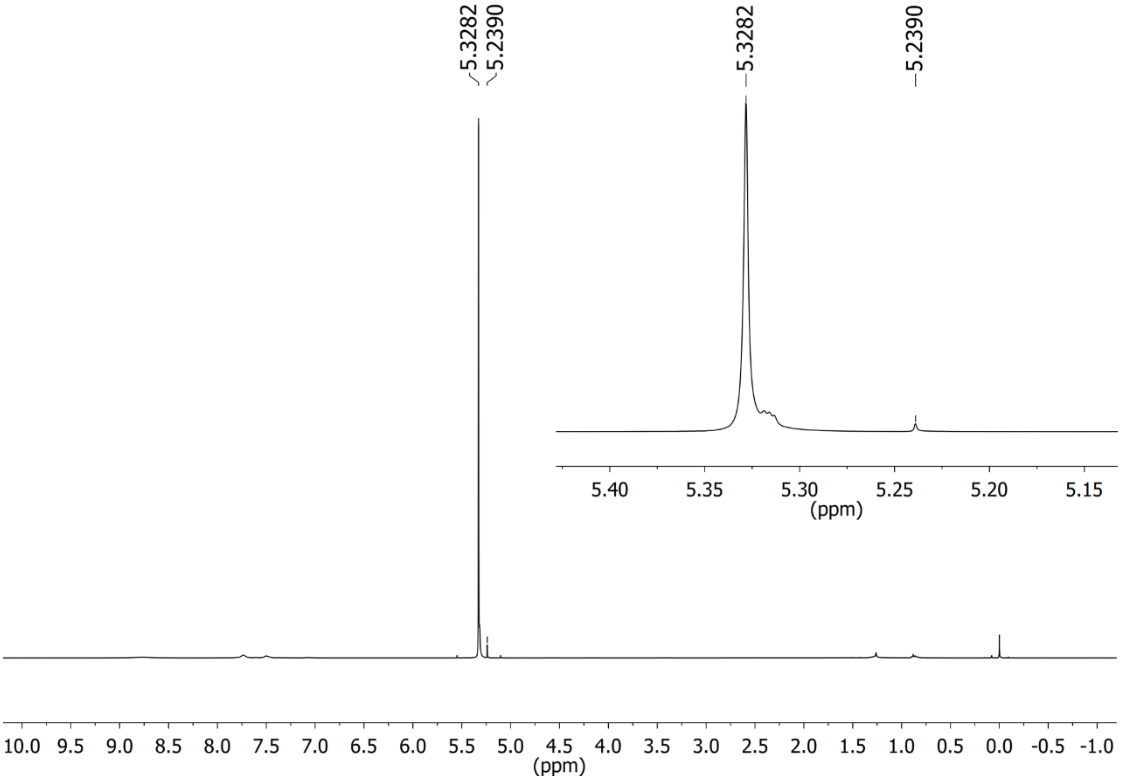

Fig. **S103** Evans' (^{1}H) NMR spectrum for determination of effective magnetic moment of **[1a(Au)$_3$](TEF)$_2$** (4.72 mmol·L^{-1}, 25 °C) in CD_2Cl_2/CH_2Cl_2 50:1 v/v. The insert shows the shift between CH_2Cl_2 (sample) and CH_2Cl_2 (reference).

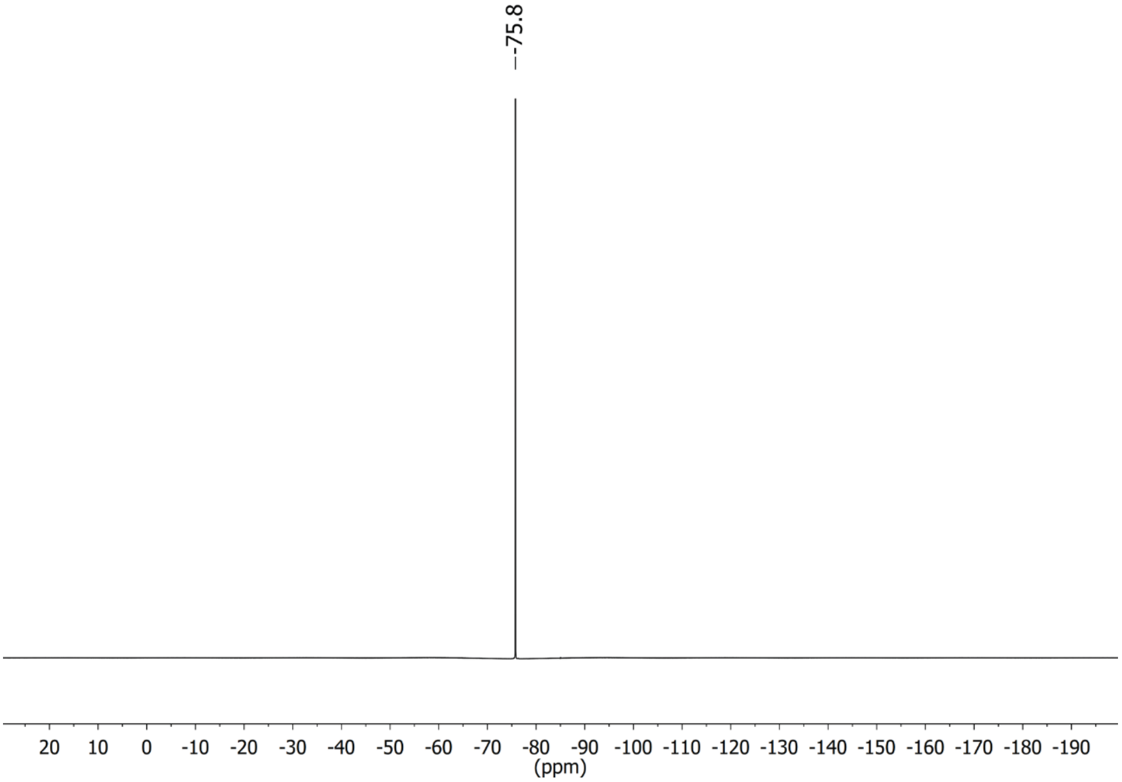

Fig. **S104** ^{19}F NMR spectrum of **[1a(Au)$_3$](TEF)$_2$** in CD_2Cl_2.

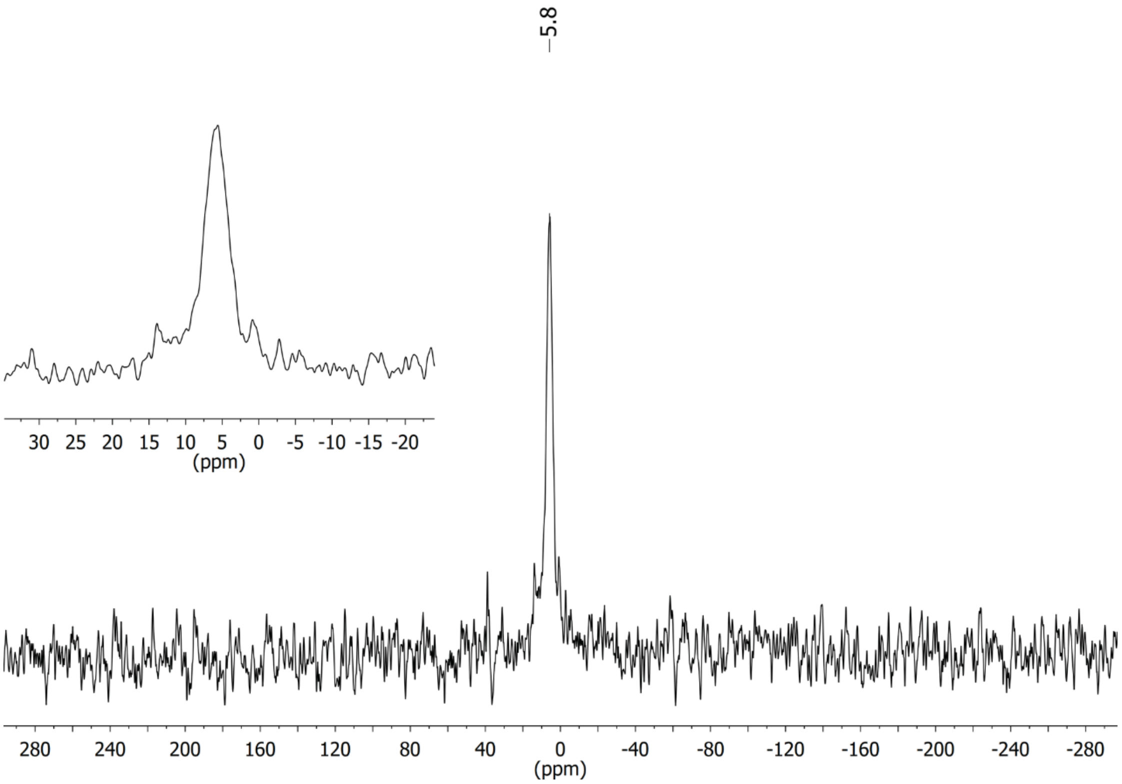

Fig. **S105** $^{31}P\{^1H\}$ NMR spectrum of **[1a(Au)$_3$](TEF)$_2$** in CD_2Cl_2, determined using the $z_g p_g$ pulse sequence for fast-relaxing nuclei. For the graphical representation, an experimental line broadening of 100 Hz has been used. The insert shows the relevant signal in detail.

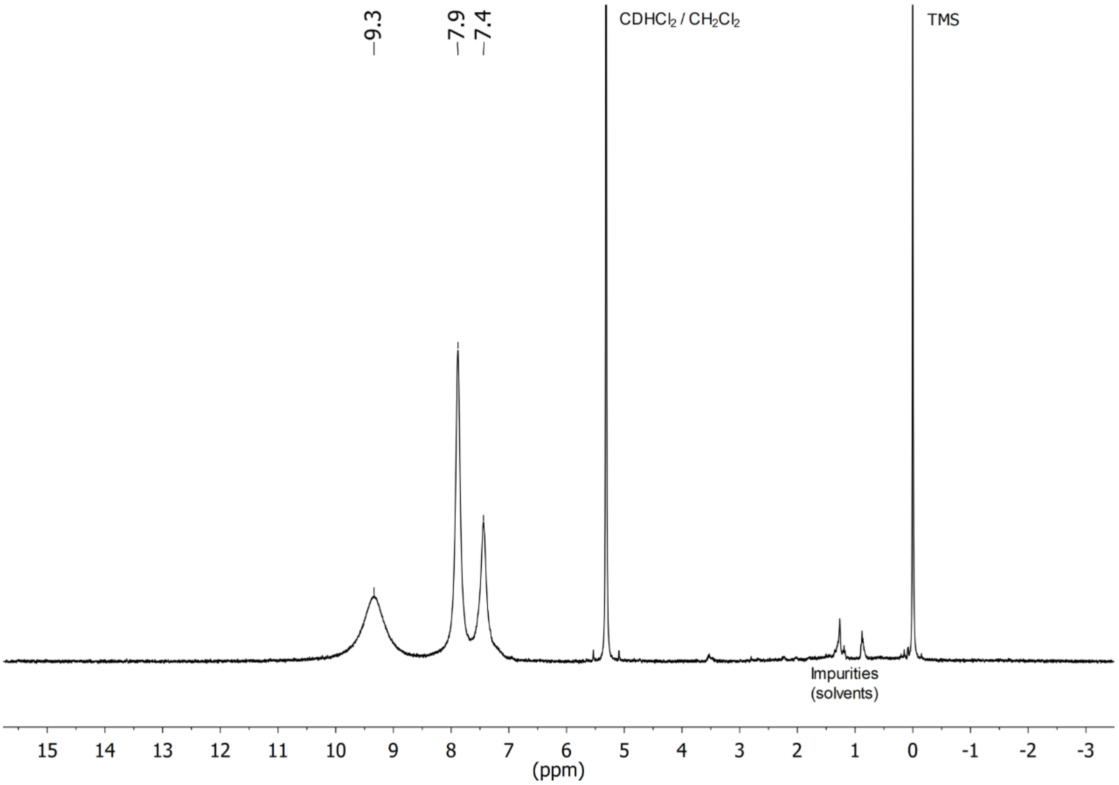

Fig. **S106** ^{1}H NMR spectrum of **[1a(Au)$_3$](TEF)$_3$** in CD_2Cl_2. No other resonances have been identified in the range of +50 to −45 ppm.

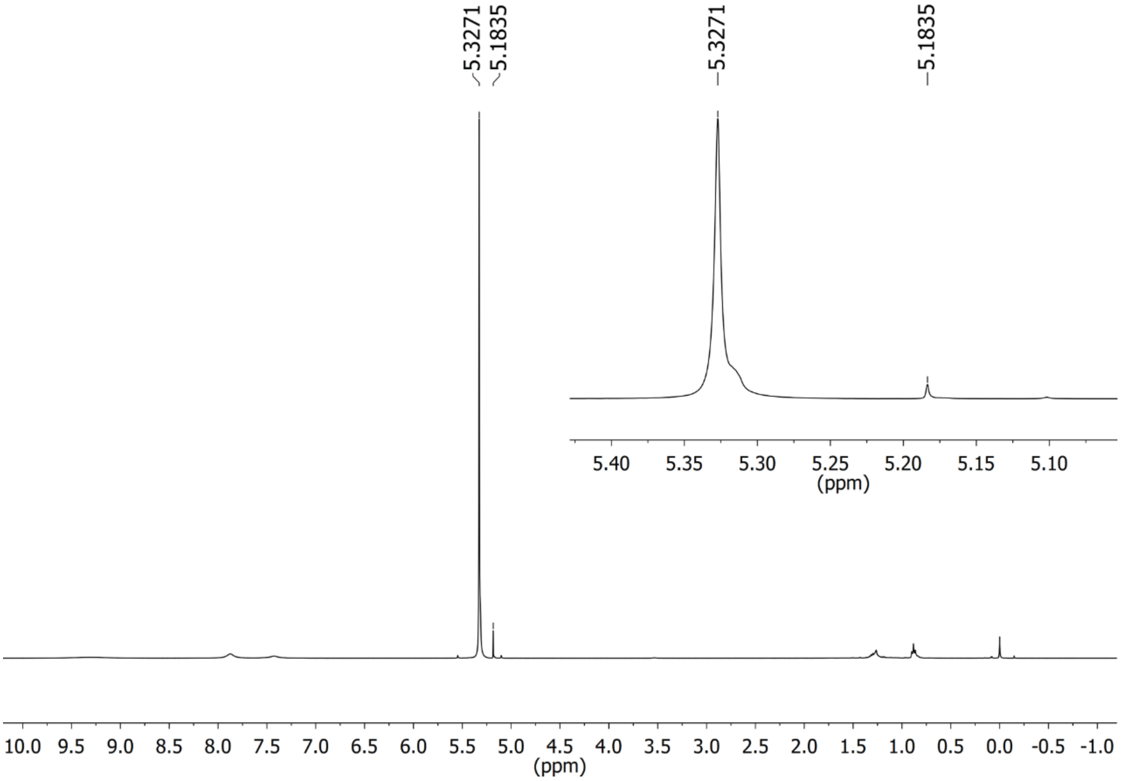

Fig. **S107** Evans' (^{1}H) NMR spectrum for determination of effective magnetic moment of **[1a(Au)$_3$](TEF)$_3$** (5.30 mmol·L^{-1}, 25 °C) in CD_2Cl_2/CH_2Cl_2 50:1 v/v. The insert shows the shift between CH_2Cl_2 (sample) and CH_2Cl_2 (reference).

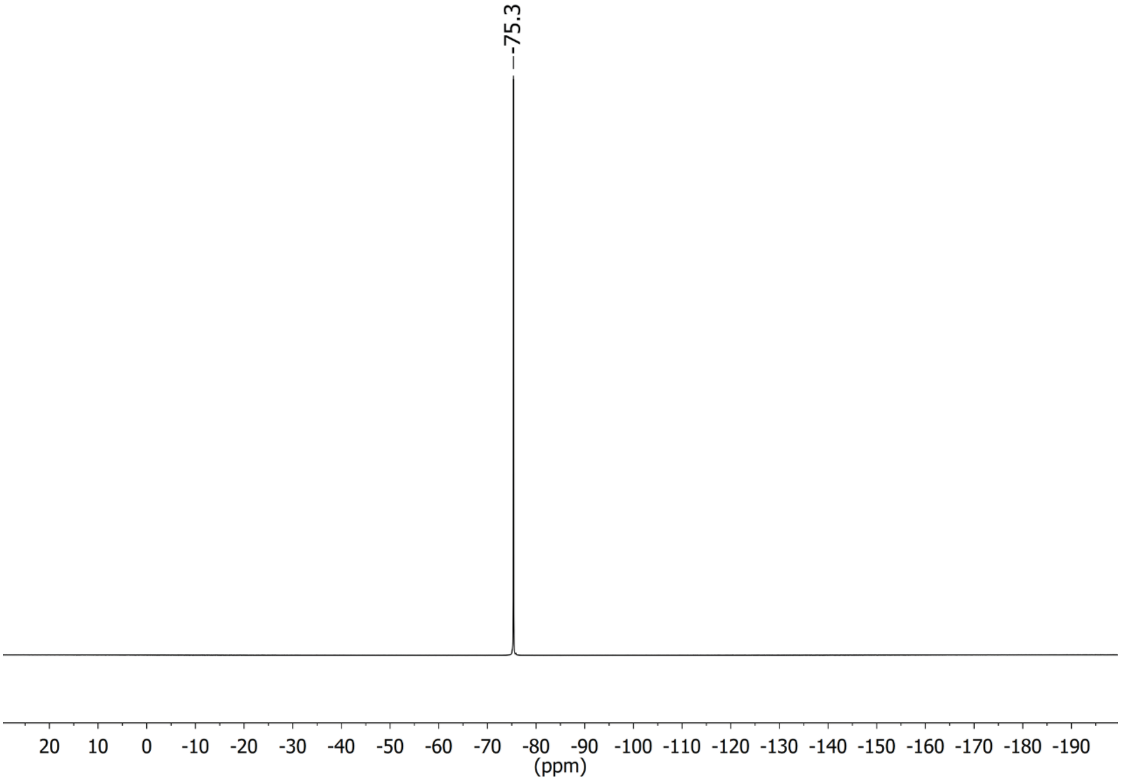

Fig. **S108** ^{19}F NMR spectrum of **[1a(Au)$_3$](TEF)$_3$** in CD_2Cl_2.

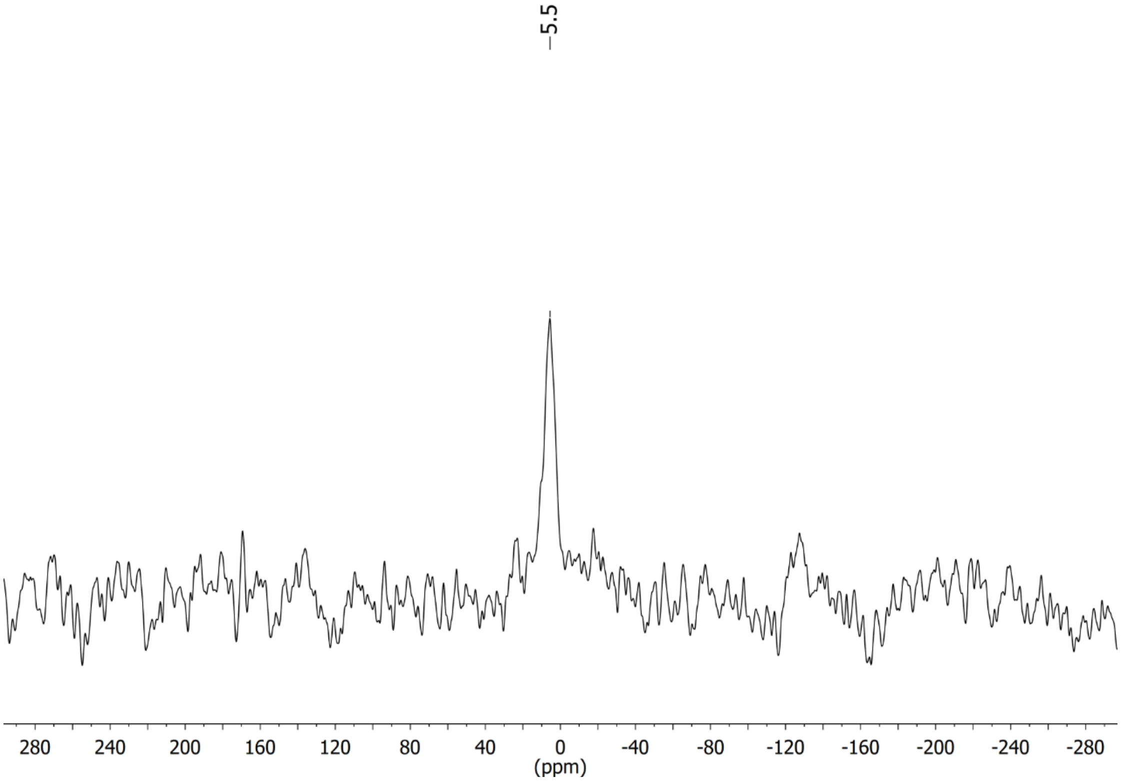

Fig. **S109** $^{31}P\{^1H\}$ NMR spectrum of **[1a(Au)$_3$](TEF)$_3$** in CD_2Cl_2, determined using the z_gp_g pulse sequence for fast-relaxing nuclei. For the graphical representation, an experimental line broadening of 300 Hz has been used.

SUPPORTING INFORMATION

DFT Coordinates for **[1a(Au)$_3$]/[1a(Au)$_3$]$^+$**

**C**	0.516695191	9.136613613	4.720476736
**C**	0.362428991	9.006771179	2.288539717
**C**	2.513137564	9.45337471	3.349688325
**C**	-0.132774581	9.0674739	6.033993703
**C**	0.520578362	8.903405851	7.310676467
**H**	1.591588808	8.816830297	7.46871279
**C**	-0.481266676	8.865465627	8.331928176
**H**	-0.306960912	8.771974583	9.39991664
**C**	-1.757272731	9.00827245	7.697407549
**H**	-2.726223489	9.056820239	8.186094595
**C**	-1.548007352	9.146331	6.292779036
**H**	-2.322382565	9.34145809	5.557357445
**C**	-1.406179059	12.25743314	8.154936602
**C**	-1.444890511	12.37093931	6.716824149
**H**	-2.333284446	12.43466165	6.096030127
**C**	-0.100655868	12.33196109	6.242738996
**H**	0.172791959	12.34641329	5.190339755
**C**	0.775764802	12.18569426	7.365615064
**H**	1.857570931	12.09431261	7.326230261
**C**	-0.022792048	12.1376534	8.55390809
**H**	0.33402002	12.00604976	9.570446419
**C**	-2.261090472	11.45168099	10.75205174
**C**	-1.451481718	12.17865618	11.63741277
**H**	-1.231500009	13.22801793	11.43458426
**C**	-0.940088301	11.56479998	12.78156705
**H**	-0.312050347	12.13496471	13.46753119
**C**	-1.238844344	10.22587494	13.05197246
**H**	-0.841172067	9.748876482	13.94880176
**C**	-2.062477088	9.505069107	12.18322307
**C**	-2.576264096	10.11705269	11.03894017
**H**	-3.237633141	9.568219699	10.36685379
**C**	-3.18365702	13.94120115	9.658145882
**C**	-4.36790666	14.24104136	10.34862427
**H**	-5.080630806	13.44371684	10.56920807
**C**	-4.635995794	15.55251182	10.73684504
**H**	-5.558307137	15.78155788	11.2721328
**C**	-3.731136741	16.57386553	10.42732533
**H**	-3.946995751	17.60118909	10.72382264
**C**	-2.558486937	16.28009513	9.728129028
**H**	-1.8570388	17.07653502	9.475754202
**C**	-2.281469883	14.96558364	9.343613274
**H**	-1.370171129	14.73355499	8.791045567
**C**	-0.437855865	8.892844669	1.067193673

C	-1.864083185	9.066952988	0.952314549
H	-2.532307671	9.327094321	1.766392379
C	-2.230347135	8.885490291	-0.416363274
H	-3.237881144	8.937486953	-0.818070001
C	-1.04227002	8.597515153	-1.159097579
H	-0.985136598	8.385090291	-2.221238385
C	0.061283945	8.592460522	-0.250426741
H	1.088160731	8.353569614	-0.510442688
C	-1.155564806	5.356283135	-0.4915523
C	-0.25239418	5.493348697	0.628199857
H	0.829535031	5.413247224	0.577589486
C	-1.034470216	5.7896757	1.786765802
H	-0.640755612	6.004857052	2.776431701
C	-2.412584265	5.845507007	1.399094615
H	-3.247569442	6.088424184	2.049875611
C	-2.497855175	5.579907187	-0.005330205
H	-3.399619768	5.574658116	-0.608843393
C	-2.040808975	5.434132066	-3.219453807
C	-3.178957581	4.614701479	-3.172760982
H	-3.183295366	3.724436701	-2.541891879
C	-4.298521969	4.930723162	-3.942308121
H	-5.181927939	4.29228414	-3.901210676
C	-4.285453756	6.058526345	-4.769337766
H	-5.161514822	6.302393833	-5.37164927
C	-3.145932264	6.863706966	-4.835274177
H	-3.126150514	7.734469505	-5.491610785
C	-2.024613903	6.550545181	-4.065260642
H	-1.120869135	7.160090702	-4.125976055
C	-0.396411159	3.262444159	-2.312082337
C	0.256149324	2.744711459	-3.441033048
H	0.663639661	3.427543585	-4.18944835
C	0.39179297	1.366105614	-3.596431066
H	0.900546679	0.966919204	-4.474736676
C	-0.112295836	0.49828751	-2.621720428
H	0.001537775	-0.579940515	-2.741151373
C	-0.750945122	1.011937423	-1.490730289
H	-1.135886142	0.337110945	-0.725025499
C	-0.894961831	2.39264143	-1.333279422
H	-1.385490087	2.798351389	-0.447383765
C	3.930065482	9.810989094	3.214327814
C	4.56013685	10.2997563	2.012728163
H	4.077821107	10.43339406	1.050600964
C	5.913206333	10.63813309	2.310660373
H	6.630373328	11.05789285	1.611632137
C	6.144039048	10.36031164	3.696655525

**H**	7.071001124	10.52688965	4.237843458
**C**	4.927440271	9.851435055	4.254122661
**H**	4.77828254	9.560765021	5.289738205
**C**	3.30285707	13.19094439	3.054310479
**C**	4.658513615	13.6873628	2.965579208
**H**	5.127225492	14.12133282	2.089274373
**C**	5.275361966	13.52081073	4.244212953
**H**	6.302785415	13.77383221	4.489944841
**C**	4.319879758	12.92329088	5.129061164
**H**	4.500090037	12.63682135	6.161168692
**C**	3.107281055	12.7199179	4.402896764
**H**	2.202486435	12.24247476	4.763250143
**C**	2.603195746	12.5006485	0.277213853
**C**	3.823223119	12.90420601	-0.289148809
**H**	4.393236896	13.71733919	0.158255157
**C**	4.310091076	12.26383926	-1.427334671
**H**	5.266918891	12.56989583	-1.852647967
**C**	3.575955058	11.22993374	-2.019940314
**H**	3.960200883	10.70702894	-2.896048851
**C**	2.340125454	10.85893742	-1.488782148
**H**	1.772371733	10.05849546	-1.961746012
**C**	1.853594793	11.49069084	-0.341789531
**H**	0.89606772	11.19273399	0.088671167
**C**	1.847551049	15.01705763	1.436605045
**C**	2.079575778	15.97357715	2.43515082
**H**	2.404327955	15.65568901	3.426638433
**C**	1.899972181	17.32934344	2.157547271
**H**	2.084967999	18.06962103	2.937069157
**C**	1.485676922	17.73721704	0.886636402
**H**	1.346604831	18.79745722	0.671593128
**C**	1.248856676	16.7845992	-0.108383048
**H**	0.92436097	17.09860666	-1.101285534
**C**	1.425971847	15.4277755	0.16384307
**H**	1.242099662	14.68477794	-0.613367364
**C**	-0.235727957	8.946034157	3.554484257
**C**	1.74072988	9.236690863	2.200020988
**C**	1.89132284	9.395687353	4.603783648
**P**	-2.858445916	12.20668924	9.201432762
**P**	-0.597416842	5.067608372	-2.16889044
**P**	1.990592447	13.23834313	1.821006417
**Cl**	-6.308747702	9.951231067	7.328910243
**Cl**	-2.08309571	11.77835089	3.233139956
**Cl**	3.028876208	7.648501228	-3.069064972
**Fe**	-0.558960199	10.63110767	7.297258451
**Fe**	-1.306143837	7.199429528	0.321992202

**Fe**	4.620386764	11.71659601	3.497894831
**Au**	-4.591670085	11.13156082	8.280875461
**Au**	0.02221591	12.41957691	2.528382419
**Au**	1.223305692	6.278554613	-2.668650185
**H**	2.213770532	9.282654694	1.22110816
**H**	-1.304243328	8.757780356	3.631329625
**H**	2.472354543	9.604658403	5.502174106
**H**	-2.314000185	8.466178497	12.40027319
